# Influence of Head‐to‐Tail Cyclization of Lanmodulin‐Inspired Peptides on Lanthanide Affinity and Structure

**DOI:** 10.1002/chem.71438

**Published:** 2026-07-25

**Authors:** Sophie M. Gutenthaler‐Tietze, Jerome Kretzschmar, Satoru Tsushima, Patrick Weis, Robin Steudtner, Björn Drobot, Lena J. Daumann

**Affiliations:** ^1^ Lehrstuhl für Bioanorganische Chemie Heinrich‐Heine‐Universität Düsseldorf Düsseldorf Germany; ^2^ Institute of Resource Ecology Helmholtz‐Zentrum Dresden‐Rossendorf e.V. Dresden Germany; ^3^ Institute of Integrated Research Institute of Science Tokyo Tokyo Japan; ^4^ Institute For Physical Chemistry II Karlsruher Institut für Technologie Karlsruhe Germany

**Keywords:** cyclic peptides, head‐to‐tail, lanmodulin, lanthanides, molecular dynamics, spectroscopy

## Abstract

Over the recent years, lanthanide (Ln)‐binding proteins such as lanmodulin, a naturally Ln‐binding EF‐hand protein from *Methylorubrum extorquens* AM1, as well as protein‐inspired short Ln‐binding peptides, have become of increasing interest for the development of separation and recycling methods for rare earth elements. Here, we comprehensively examined a set of head‐to‐tail cyclized lanmodulin‐EF‐hand sequence‐inspired peptides and their complexation behavior toward Lns using time‐resolved laser‐induced fluorescence spectroscopy (TRLFS), isothermal titration calorimetry (ITC), nuclear magnetic resonance (NMR), and circular dichroism (CD) spectroscopies, complemented by molecular dynamic (MD) simulations and advanced mass spectrometry methods. Results obtained from this multi‐method approach evidence that the head‐to‐tail cyclization can indeed increase the Ln affinity in some cases and positively impact the pre‐organization of the metal‐free peptide while predominantly forming 1:1 complexes in comparison to linear analogues, which can form both 1:1 and 1:2 species. Furthermore, the impact of a sequence alteration by inserting glycine at the head‐to‐tail junction is shown to be useful in NMR experiments for probing alterations in the local environment of neighboring amino acids (AA) potentially involved in coordination. Lastly, *K_D_* values for the peptides with the whole Ln series (except Pm) and the actinide curium are presented.

## Graphical abstract

The influence of head‐to‐tail cyclization, as well as the effect of ring expansion with an additional glycine on lanmodulin‐inspired peptides on the structure and lanthanide (Ln)‐binding behavior was investigated. While the decrease of structural flexibility can indeed increase the Ln affinity and peptide pre‐organization, it does not lead to an affinity boost in all cases.

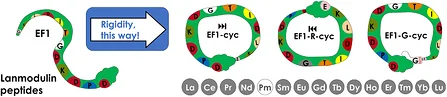

## 1 Introduction

Over the last few decades, the demand for rare earth elements (REEs) has drastically increased as a wide range of our technological advances rely on this group of metals [1]. The group of REEs consists of the f‐block elements lanthanides (Lns), lanthanum to lutetium, as well as scandium and yttrium. While their spectroscopic and magnetic properties made them popular in a wide range of applications, their extraction and separation remain to this day a tedious and environmentally harmful process [2, 3]. Especially in recent years, the research on peptides and proteins for the design of greener solutions for precisely that—REE extraction and separation—has gained popularity [4]. A key factor for this development was the discovery of the bio‐relevance of some Lns for methylotrophic bacteria [5, 6, 7] as well as the subsequent discovery of Ln‐binding proteins in these bacteria, such as lanmodulins [8, 9, 10, 11], lanpepsy [12], and landiscernin [13]. In particular, the isolation of the EF‐hand protein lanmodulin (LanM) from *Methylorubrum extorquens* AM1 by Cotruvo and coworkers in 2018 [8] prompted the investigation of LanM‐based short peptides by multiple groups. While in some studies the focus was on gaining fundamental understanding of the metal‐binding properties of the LanM metal‐binding loop sequences [14], others already focused successfully on proof‐of‐concept studies, for example, Ln‐sensing or separation. For example, Renner and coworkers have recently immobilized amino acid (AA) sequences based on LanM on a gold sensor for selective REE‐binding [15], functionalized membrane fibers with LanM‐inspired peptides for successful REE recovery [16, 17], and showed a potential application of such peptides in Ln‐biosensors [18]. Cao and coworkers, on the other hand, focused on using modified LanM sequences for the selective recovery of scandium [19], and Sree et al. successfully immobilized a 12‐AA long peptide derived from LanM for the separation of Eu^3+^ from some transition metals, Al^3+^, as well as some alkali and alkaline earth metals [20]. Other studies thereby focus on developing screening platforms to find new potent Ln‐binding peptides and proteins [21, 22]. The research on LanM‐derived peptides for multiple applications thereby strongly benefited from the extensive research that had been done by multiple groups in the past decades, designing Ln‐binding peptides based on the EF‐hand loops of the Ca^2+^‐binding calmodulin proteins [23, 24]. Especially noteworthy is the pioneering work by Szabo and coworkers [25, 26] as well as the work of Imperiali and coworkers on Ln‐binding tags (LBTs), which were originally primarily optimized for a high Tb^3+^ affinity [27, 28, 29, 30]. LBTs have nowadays found multiple applications, ranging from their original use as luminescent tags in proteins to the more recent use for the recovery of REEs [24, 31, 32]. Recently, Berthomieu et al. replaced an EF‐hand binding site in a calmodulin protein with a slightly modified LBT‐sequence based on Nitz et al. [30] to obtain CaMLBT for which they observed a picomolar Ln‐affinity and thereby approaching the binding range of lanmodulins [33]. Interestingly, the majority of Ln‐binding peptide research focuses on linear peptides, while studies on cyclic peptides are scarce, even though there have been very promising results on Ln‐binding cyclic peptides in the past. For example, Nitz et al. observed a remarkable 30‐fold increase in Tb^3+^ affinity (*K_D_* = 2 ± 1 nM) for a LBT that was side chain‐to‐side chain‐cyclized due to the introduction of a disulfide bond in comparison to the linear version [29]. Ishida and coworkers also used the formation of a disulfide bond to obtain short (13–15 AA long) cyclic lanthanide ion mineralization peptides (Lamps), which can selectively extract precipitated REE hydroxide species for REE recovery [34, 35]. Chiniforoush et al. very recently designed Ln‐binding cyclic peptides (LBCPs) using a computing library with the focus on peptide sequences with less than 30 AAs, three to four carboxylate side chains, and again cyclization upon the formation of an intramolecular disulfide bond [36]. Studies focusing on head‐to‐tail cyclized Ln‐binding peptides are even more scare in the literature. Delangle and coworkers designed a head‐to‐tail cyclized decapeptide termed PA, inspired by the work on LBTs with Ln‐affinities in the nanomolar range and a controlled conformation with two *β*‐turns [37]. In general, cyclic peptides are associated with increased rigidity, which can reduce the entropic cost of binding and lead to a higher affinity. Furthermore, cyclic peptides are of particular interest for the design of peptide drugs due to their inherently higher stability against enzymatic breakdown and membrane permeability [38, 39]. However, a key challenge is the often poorly understood sequence–structure relationship in this class of peptides, which makes their design for specific applications especially challenging [40]. So far, to the best of our knowledge, the potential of LanM binding sequences for the development of cyclic Ln‐binding peptides has not yet been thoroughly explored. Only Chiniforoush et al. used a via disulfide bond cyclized peptide based on LanM's EF1 binding loop as a comparison to test the resilience of their molecular dynamics (MD) simulations [36]. Herein, we investigate two short head‐to‐tail cyclized LanM‐inspired peptides (EF1‐cyc and EF1‐R‐cyc) in detail by isothermal titration calorimetry (ITC), time‐resolved laser‐induced fluorescence spectroscopy (TRLFS), nuclear magnetic resonance (NMR), and circular dichroism (CD) spectroscopies, as well as MD simulations and advanced mass spectrometry methods to test whether the introduced rigidity due to the cyclization can increase the Ln‐binding affinity of the previously examined linear LanM‐inspired 12‐AA‐long peptides. Furthermore, we also study their binding to the actinide analogue Cm^3+^, as well as Ln‐selectivity, and investigate the effect of a sequence modification involving the insertion of a glycine at the head–tail junction.

## 2 Results and Discussion

We previously studied linear, uncapped peptides based on the 12‐AA‐long metal‐binding loops of lanmodulin, which were synthesized according to the natural sequence (= “forward” peptides) as well as by reading the sequence backwards (‐R, “reverse” peptides). For these peptides, we were able to observe the formation of 1:1 and 1:2 complexes with Lns^3+^, low micromolar Ln‐affinity for the forward peptides, and a nanomolar Ln‐affinity for some of the reverse peptides [14, 41]. We hypothesized that one key factor for the formation of multiple complexes in such short peptides is the high structural flexibility due to the linearity, and suspected that the affinity of the EF‐hand peptides might be further improved by increasing the rigidity of the structure due to, for example, cyclization. To test this, we obtained both a “forward” as well as a “reverse” peptide originally based on the same sequence (EF1 metal‐binding loop in lanmodulin) as their head‐to‐tail cyclized variants EF1‐cyc and EF1‐R‐cyc (Figure 1) and investigated their binding behavior toward Lns in detail.

**FIGURE 1:**
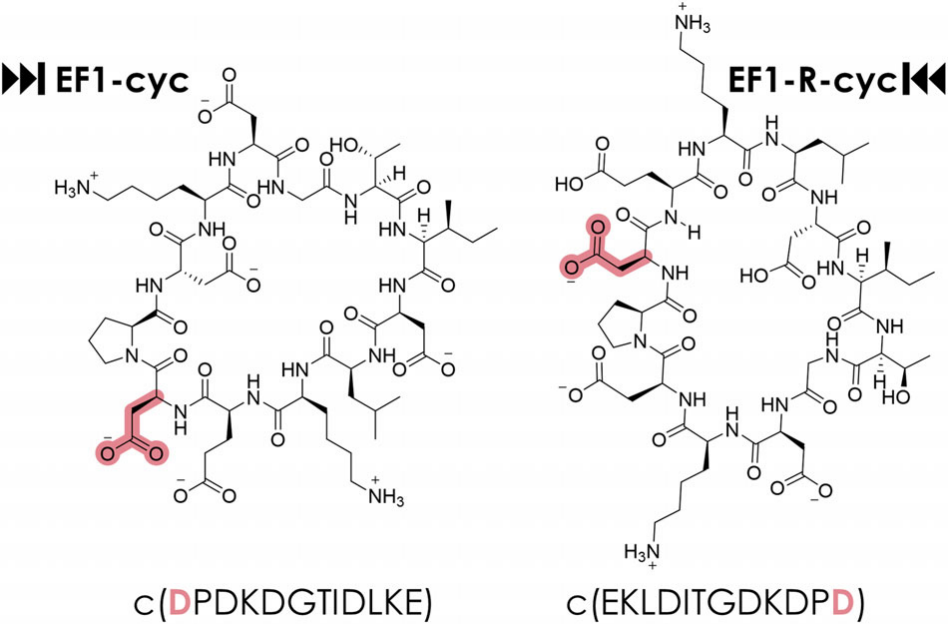
Comparison of the head‐to‐tail cyclized peptides EF1‐cyc and EF1‐R‐cyc. For orientation, the N‐ or C‐terminal aspartic acid residue previously present in the corresponding linear peptides is highlighted in red in EF1‐cyc and EF1‐R‐cyc, respectively.

### 2.1 Thermodynamic Investigation of EF1‐cyc and EF1‐R‐cyc

To study the thermodynamics of the isomeric cyclic peptides EF1‐cyc and EF1‐R‐cyc with Lns and to compare their binding properties, we used a combination of TRLFS and ITC. For consistency and comparability between the methods, the experiments were performed with Eu^3+^ owing to its excellent luminescence properties, which are exploited in TRLFS investigation. TRLFS data were analyzed using modified parallel factor analysis (PARAFAC) [42, 43]. ITC experiments were performed using a setup in which the same Eu^3+^ concentration was titrated to in total three different peptide concentrations. The data was then analyzed globally with the advantage to obtain a better overview over the tested concentration range in comparison to an ordinary setup using triplicates [41]. Both methods show the formation of only a 1:1 complex in contrast to the analogous linear peptides for which an additional 1:2 peptide:Eu^3+^ was observed [14, 41]. The obtained affinity values (*K*_D_) and luminescence lifetimes (*τ*) for both peptides with Eu^3+^ are shown in Table 1. Obtained single‐component spectra and associated luminescence lifetimes, as well as correspondingly derived species distribution together with matching ITC data, are shown for EF1‐cyc in Figure 2 (cf. Figure S37 for EF1‐R‐cyc). As seen from Table 1 and Figure 2, both methods are in excellent agreement, and the affinity values for Eu^3+^ are identical within the error range.

**TABLE 1:** Binding affinity values *K_D_* for EF1‐cyc and EF1‐R‐cyc with Eu^3+^ obtained by ITC (global analysis of three independent replicates with varying peptide concentrations in the cell; cf. Table S3 and Figures S2‐S5) and by TRLFS experiments (global analysis of independently measured duplicates; cf. Figures S36 and S37), along with the luminescence lifetime (τ) of the Eu^3+^‐EF complex. Experimental conditions: 10 mM MOPSO, 100 mM KCl, pH 6.6. All errors were obtained by a Monte Carlo (MC) approach.

	ITC	TRLFS	
EF‐Eu^3+^	*K*_D_ (µM)	*K_D_* (µM)	τ (µs)^a^
EF1‐cyc	3.2 ± 0.1	3.3 ± 0.2	247 ± 5
EF1‐R‐cyc	8.2 ± 0.2	8.1 ± 0.5	180 ± 2

^a^ Luminescence lifetimes of the Eu^3+^‐aquo ions are listed in Table S5.

**FIGURE 2:**
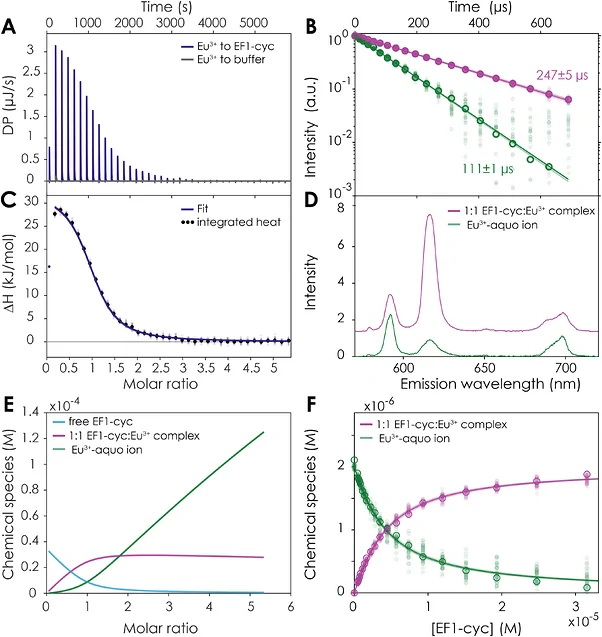
ITC and TRLFS data of titration experiments with EF1‐cyc and EuCl_3_. (A) ITC thermogram, (C) integrated heat and corresponding fit as well as (E) chemical species distribution for a EuCl_3_ (900 µM) to EF1‐cyc (30 µM) titration. The dataset was analyzed globally with titrations using 45 or 60 µM in the ITC cell. Luminescence lifetimes (B), stacked single‐component spectra (D), and chemical speciation (F) derived from two globally analyzed EF1‐cyc to EuCl_3_ TRLFS titration experiments (*λ*_ex_(Eu) = 394 nm; 0–22.7 µM peptide, 2 µM EuCl_3_). Subfigures B, D, and F share the same color code. Overlaid transparent lines/ symbols always represent the used Monte Carlo (MC) runs for error estimation. Experimental conditions: 10 mM MOPSO, 100 mM KCl, pH 6.6). The results of the full ITC data set are shown in Figures S2 and S3; additional spectra to the TRLFS experiment are shown in Figure S36.

For EF1‐cyc, we observe an Eu^3+^‐affinity (*K_D_*) around 3 µM, while the reverse peptide EF1‐R‐cyc binds somewhat more loosely (*K_D_* ∼ 8 µM). With these binding affinities, the peptides are within the expected range of normal EF‐hand‐based peptides [24]. Therefore, the cyclization did not lead to a significant affinity increase, as for example, observed for an LBT cyclized via an intramolecular disulfide bond, afterwards reaching a Tb^3+^ affinity of 2 nM [29]. However, when compared with the linear analogues, we only observe a slight affinity increase for the cyclized forward sequence EF1‐cyc (ITC: EF1: 8.6 ± 0.2 µM [14] vs. EF1‐cyc: 3.2 ± 0.1 µM (this work)). We explain this with the decrease in flexibility (reduction of conformational degrees of freedom) and thus higher rigidity and potential pre‐structuring. For EF1‐R‐cyc, however, the cyclization causes a decrease in affinity by a factor of 16 when compared to linear EF1‐R, for which an Eu^3+^‐affinity in the 1:1 complex of 520 ± 30 nM [41] was observed by ITC. We previously showed that one key feature responsible for the nanomolar affinity of EF1‐R is the combination of a C‐terminal aspartic acid and a free C‐terminal carboxylate, forming a succinate‐like motif [41]. This free C‐terminal carboxylate is eliminated upon head‐to‐tail cyclization. To show the importance of the free C‐terminal carboxylate for the nanomolar affinity, we previously investigated a linear analogue of EF1‐R in which the C‐terminal carboxylate was blocked by the introduction of a methyl ester group. The thus obtained peptide, termed EF1‐R‐OMe, showed a decreased affinity of 31.8 ± 2.1 µM via ITC, impressively showing the significant impact of the succinate‐like motif on the binding affinity in the reversed sequences [41]. Despite the blocked succinate‐like motif, EF1‐R‐cyc binds four times more tightly (*K_D_* ∼ 8 µM) as compared to EF1‐R‐OMe. This is consistent with the aforementioned pre‐structuring by cyclization and the associated increase in affinity relative to the flexible linear peptides. Therefore, head‐to‐tail cyclization can represent a useful strategy to enhance the affinity of short peptides, as long as important binding motifs are not blocked.

Both peptides were also investigated with Cm^3+^, as we previously observed a slightly higher affinity for EF1 and its analogues for this actinide in comparison to the Ln Eu^3+^ [14]. This was in line with the higher affinity of LanM for actinides than for lanthanides [44, 45], and is of particular interest in view of optimizing and designing peptide‐ligands for the separation of lanthanides and actinides. To ensure that the Cm^3+^‐aquo ion is the exclusive metal ion species at the beginning of our titration experiments, the pH was lowered from 6.6 to 6.0, as compared with Eu^3+^. The protonation state of the peptide remains unaffected and, consequently, so is the comparability of the Eu^3+^ and Cm^3+^ experiments. The analysis of the titration experiments of EF1‐cyc and EF1‐R‐cyc to Cm^3+^ is shown in Figure 3.

**FIGURE 3:**
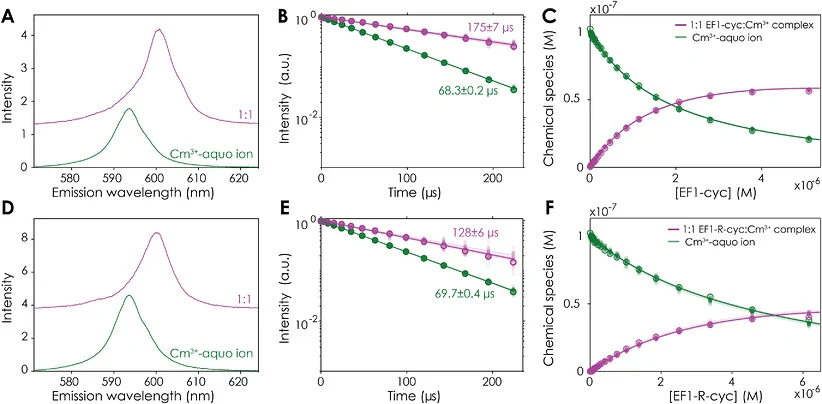
TRLFS data of peptide to Cm^3+^ titration experiments. Stacked single‐component spectra (A), luminescence lifetimes (B), and chemical species distribution (C) of a EF1‐cyc to Cm^3+^ titration experiment; the color code given in C also applies to A and B. Stacked single‐component spectra (D), luminescence lifetimes (E), and chemical species distribution (F) of a EF1‐R‐cyc to Cm^3+^ titration experiment; the color code given in (F) also applies to (D) and (E); Overlaid transparent lines/ symbols always represent the used Monte Carlo (MC) runs for error estimation. Experimental conditions: 10 mM MOPSO, 100 mM KCl, pH 6.0, λ_ex_(Cm) = 396 nm. Additional spectra are shown in Figures S39 and S40.

From the experiments, we found that both peptides, EF1‐cyc and EF1‐R‐cyc, exhibit an affinity around factor 2 higher for Cm^3+^ than for Eu^3+^ (EF1‐cyc: 1.9 ± 0.03 µM, EF1‐R‐cyc: 5.1 ± 0.16 µM), which is in the same range as previously observed, for e.g. EF1 [14]. Therefore, the cyclization had no effect on the selectivity for Cm^3+^ over Eu^3+^ beyond the expected level. In the literature, it has been shown for LBTs that their affinity for the actinide Am^3+^ can be enhanced by selectively introducing softer binding motifs. For example, by exchanging a single AA for cysteine functionalized with a 2‐methylenepyridine group Özçubukçu et al. could increase the Am^3+^ selectivity over the Ln Nd^3+^ by a factor of 10 [46].

### 2.2 Structural Investigation of EF1‐cyc and EF1‐R‐cyc

As we have previously successfully used NMR titration experiments with different Lns to obtain structural insights into the formed peptide‐Ln^3+^ complexes [41], we investigated both peptides with three different Lns. To cover the extremes of the Ln series, we chose the two diamagnetic Lns La^3+^ and Lu^3+^, as well as the paramagnetic Ln Sm^3+^. In our previous study, we had used Eu^3+^ in such experiments to remain consistent among the different methods [41]. However, due to Eu's paramagnetism, resonances vanished very rapidly during titration experiments at the expense of gaining structural information. Therefore, we decided to switch to the neighboring Ln Sm^3+^ for the NMR experiments as it is known for its weaker paramagnetism [47].

By using a combination of ^1^H NMR spectroscopy and 2D NMR experiments (TOCSY, HSQC, and HMBC), we characterized all peptides in the absence of Lns via assignment of all ^1^H and ^13^C resonances. The full assignment for EF1‐cyc and EF1‐R‐cyc can be found in the Supporting Information (EF1‐cyc: Figure S47, and Tables S8 and S9, EF1‐R‐cyc: Figure S49, and Tables S10 and S11). This allowed us to not only determine metal ion complexation‐induced changes (see below) but also to complement CD spectroscopic studies on the peptides’ secondary structure characteristics.

The observed chemical shifts of the metal‐free peptide samples were examined regarding the indication of secondary structure features (Figures S52 and S53). In proteins, secondary chemical shifts (SCS) of essentially all AA ^13^Cα (^1^Hα) signals exhibit a clear downfield (upfield) trend on helix formation and a clear upfield (downfield) trend on β‐strand formation [48, 49]. Since the protein‐associated terms helix and strand are hardly applicable to such short cyclic peptides as studied in this work, we prefer to express deviation from random coil characteristics as local order. As inferred from a CD‐by‐NMR plot (Figure S52) of the NH/Hα fingerprint region, normally clustering signals by helices, strands, or coils, no matter what the cyclic peptide, a significant number of NH signals are outside the chemical shift region (*δ*_H_ 9.0‐8.2 ppm) characteristic for random coil [49, 50]. Additionally, we determined SCS as deviations from reported, nearest neighbor effects‐corrected random coil ^1^Hα and ^13^Cα chemical shift values [49] (Figures S53), which show two major results. First, the SCS shows deviation from flexible peptide chain random coil values, mirroring that upon cyclization, the conformational variability is reduced and pre‐structuring causes local order, perfectly fitting the measured CD data (see the metal‐free peptide spectra in Figures 4, S43, and S44). Second, these local order domains, that is, a train of at least four consecutive AAs showing the same positive/negative deviation, in EF1‐cyc are distinct from those in its reversed‐sequence counterpart EF1‐R‐cyc. That is, ^1^Hα and ^13^Cα SCS combined are significant for Asp9 – Glu12 in EF1‐cyc, whereas in EF1‐R‐cyc they are for Leu3 – Thr6 (excluding Gly on purpose). Another observation when comparing the NMR spectra of the linear peptides with the cyclic forms is that the Gly Hα resonances are more distinct as the conformational variability is reduced.

**FIGURE 4:**
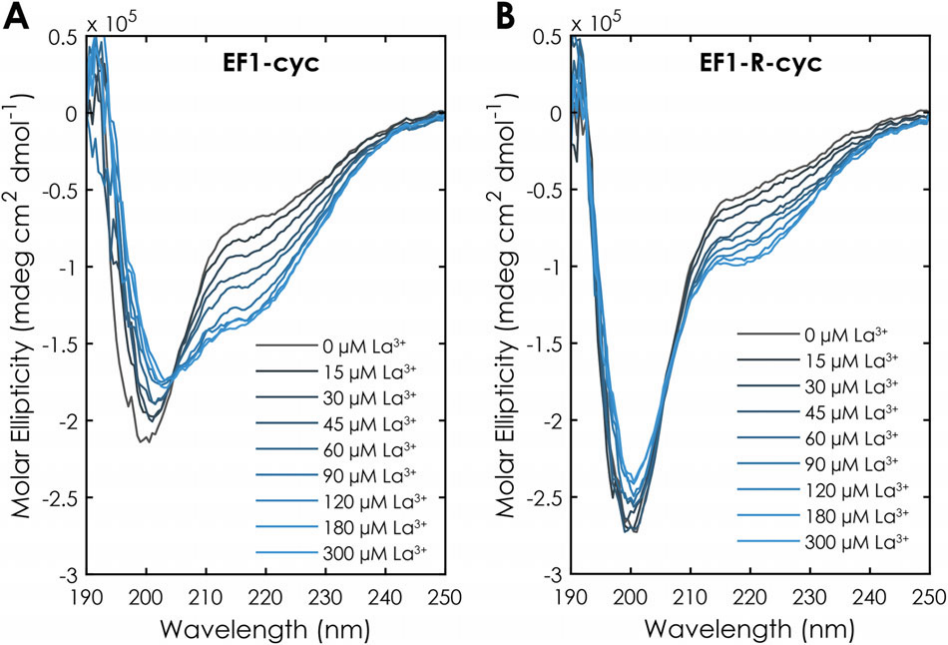
CD spectra of LaCl_3_ to (A) EF1‐cyc and (B) EF1‐R‐cyc titration experiments. Conditions: 60 µM peptide, 25°C, pH 6.6 (10 mM MOPSO, 100 mM KCl).

When adding LnCl_3_ to both peptides, especially for the NH resonances, significant shifts and signal broadening can be observed, suggesting a structural change in the peptide backbone (Figures S54–S59), accompanied with exposure of NH to solvent, facilitating exchange of sites formerly shielded from solvent. This fits the measured CD data (see Figures 4, S43, and S44), which also showed an increase in organization upon Ln addition.

In comparison to the linear peptides (see literature [41]), both EF1‐cyc and EF1‐R‐cyc show already in the metal‐free state a slightly more pronounced band at 222 nm, deviating from the classic random‐coil spectrum [51]. This already suggests a greater degree of pre‐organization before the addition of Lns and is in line with the expectations for more constrained cyclic peptides in comparison to highly flexible linear peptides. In general, after the addition of all tested Lns (La^3+^, Sm^3+^, Eu^3+^, and Lu^3+^), the CD spectra show for both peptides a well‐pronounced decrease of the band at 222 nm and an increase of the band at around 200 nm. The only deviation from this pattern is the titration experiment of Lu^3+^ with EF1‐cyc, for which the band around 200 nm stays relatively unchanged. From La^3+^ to Lu^3+^, one can observe that for both peptides, the greatest structural change is obtained for Sm^3+^ and Eu^3+^, the Lns in the middle of the series. Notably, for EF1‐cyc the addition of La^3+^, Sm^3+^, and Eu^3+^ not only leads to the increase of the band around 200 nm, but also to a shift toward 210 nm, starting to resemble the CD spectra of a *α*‐helix [51, 52]. While we do not expect a 12‐AA cyclic peptide to be able to form a α‐helix, the CD spectra definitely suggest a higher grade of organization upon Ln addition. This is also in line with MD simulations (see Figure S86) performed for EF1‐cyc with Ln^3+^ (Ln = La, Eu, and Lu). Interestingly, Delangle and coworkers observed for a head‐to‐tail cyclic decapeptide including two prolylglycine sequences based on ROESY‐NMR and CD data, evidence for two β‐turns at pD 3.4, while at pD 6.5 and pH 7 the spectroscopic data indicated predominating random coil conformation in the metal‐free state, but persisting β‐turns for the 1:1 Ln complexes [37].

One interesting point to confirm from the NMR perspective was the formation of only a 1:1 complex, as hinted by the ITC and TRLFS data. This can be clearly seen from the course of the shifts within the titration series (see Figures S54–S59). In general, a continuous shift up to a peptide‐to‐metal ratio of 1:1 can be observed for affected resonances, which eventually approaches saturation with an excess of Lns^3+^, and no new resonances appear at higher Ln^3+^ concentrations. This is particularly evident for the Thr Hγ resonance for EF1‐cyc, shown in Figure 5A. The only deviation from this trend is the titration series of Lu^3+^ to EF1‐cyc, for which an additional set of signals (♣) besides the resonance of the 1:1 complex (*) seems to appear at an excess of Lu^3+^. Since the second set of signals emerges in the metal excess regime, we ascribe it to a 2:1 Lu^3+^‐EF1‐cyc complex, likely provoked by the rather high concentrations (2 mM Lu^3+^ and 200 µM EF1‐cyc at the end of the titration) used for NMR. Furthermore, it also suggests that the Thr OH group might be indirectly involved in metal‐binding via a hydrogen bond in EF1‐cyc to a coordinating water molecule, while the steady resonance in EF1‐R‐cyc (Figure 5B) suggests no such feature. Keeping in mind that in case of EF1‐R‐cyc, the Thr OH is not involved, the Thr methyl signal responds to effects elsewhere in the peptide molecules. Additionally, the lower affinity—especially for Lu^3+^—is likely accompanied by reduced kinetic stability, hence metal excess conditions cause metal exchange reactions. According to common trends related to Ln contraction, for La^3+^, the exchange regime is very fast on the NMR time scale (600 MHz, 25°C), while it is somewhat slower for Lu^3+^, hence causing line broadening. For high excess of Sm^3+^, additionally, for both peptides, the increased bulk susceptibility affects more or less all signals, as seen also for other residues; for instance, the Leu and Ile methyl signals (cf. also Figure S58).

**FIGURE 5:**
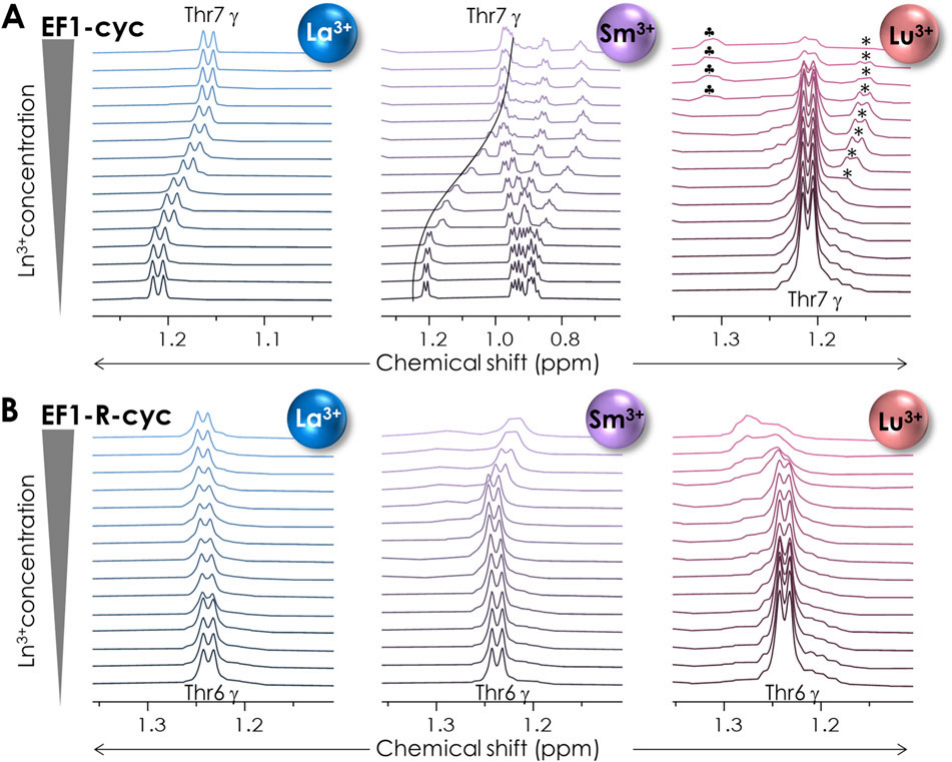
Selected region of the LnCl_3_ to EF1‐cyc (A) and EF1‐R‐cyc (B) NMR titration series (Figures S54–S59) showing the Thr γ resonance. ♣: Indicates the resonance assigned to the potential formation of a 1:2 EF1‐cyc:Lu^3+^ complex; *: Indicates the resonance assigned to the 1:1 EF1‐cyc:Lu^3+^ complex. The steady signal at 1.21 ppm is assigned to the free peptide. *Note*: The Ile8 γ2 (EF1‐cyc) / Ile5 γ2 (EF1‐R‐cyc) multiplet resonates closely. Experimental conditions: 600 MHz, H_2_O/D_2_O (9:1, *v/v*) + 0.003% TMSP‐*d*_4_, 30 mM MES‐*d*_13_, 100 mM KCl, pH 6.6; the exact Ln:peptide ratios were omitted from the figure for clarity, but are the same as stated with Figure 6.

We also employed MD simulations to get structural insights of the Ln^3+^‐bound state of EF1‐cyc. As the investigated short cyclic peptides have a significantly reduced structural flexibility compared to their linear analogues, we performed simulations on EF1‐cyc in the presence of Ln^3+^ ions, without prior relaxation of the peptide structure. The inherent rigidity of the cyclic peptide is a critical factor in determining the optimal location for metal ions at the beginning of the simulations. At first, we performed a series of simulations in which five carboxylic groups were initially forced to be bound to Ln^3+^. These simulations always ended up with all five carboxylates still bound to Ln^3+^, even after simulation times of 500 ns. As we were not able to confirm the involvement of all five carboxylates in Ln^3+^ coordination by NMR, we took an alternative approach and performed another set of simulations in which metal ions are initially distantly located from the peptide, allowing them to freely ascertain their optimal positions. Again, we employed a long simulation time (500 ns) due to the rigid nature of EF1‐cyc, which eventually facilitated the Ln^3+^ ions to slowly find their optimal coordination patterns. These simulations were performed using two distinct sets of 12–6–4 type LJ parameters on Ln^3+^, one set originally developed by Merz and coworkers [53], and the other modified by Hay and coworkers [54]. The latter is better adapted for the investigation of metal‐binding peptides. The simulations employing Merz's parameters converged to the coordination patterns involving Asp1, Asp3, and Glu12, as well as several backbone oxygens. In contrast, those employing Hay's potentials converged to the coordination patterns involving exclusively Asp3, Asp5, and Asp9 (cf. Table S32). As the latter results matched significantly better with our NMR data, we hereafter focused on using Hay's parameters. However, as already pointed out by Slough et al. [40], *φ* and *ψ* dihedrals in cyclic peptides do not frequently change within a 500 ns simulation time, which in general imposes a clear limitation of using classical MD simulations for this type of peptide. Thus, MD results were solely used as support for our experimental data.

To address experimentally which residues are involved in binding, we focused our NMR studies on the Asp *β* as well as Glu γ resonances, as they are close to the carboxylate residues which tend to bind Lns. When looking at the spectral region of the Asp *β* and Glu *γ* resonances as shown in Figure 6A for EF1‐cyc, it can be seen that for Asp1 and Glu12 only negligible shifts are observed, strongly suggesting that neither of these side chains is involved in the Ln^3+^ coordination. The Asp1 β2 signal is well separated from the other Asps’ signals, allowing assigning that residue throughout the titration series. In contrast to that, the *β* resonances of Asp3, Asp5, and Asp9 show significant shifts to a lower field with magnitudes depending on the Ln^3+^, suggesting differences in the binding modes between La^3+^, Sm^3+^, and Lu^3+^. Despite severe signal overlap, by means of TOCSY spectra of the metal‐free and metal‐bound peptide, as exemplarily shown in Figure 6B for EF1‐cyc with La^3+^, all Asp residues were assigned. For La^3+^, only two out of the four Asp seem to be involved in binding, as only Asp3 *β*1 and Asp5 *β*2 signals show significant displacements of around 230–320 ppb. For the Asp9 *β* signals, only minor shifts (−30–−50 ppb) to higher field can be observed, suggesting no involvement in the coordination (see Tables S14–S16 for a full list of Asp and Glu signals with excess Lns^3+^). In presence of the paramagnetic Sm^3+^, the spin systems of Asp3, Asp5, and Asp9 cannot be unambiguously assigned, and the spin system of one Asp cannot be detected at all. The latter is assumed to be indicative of paramagnetic effects from Sm^3+^ proximity. However, no matter how the observable signal sets get permutated for calculating the complexation‐induced signal displacements, values in the 300–500 ppb range are obtained, clearly suggesting an involvement of these three residues in the coordination. For Lu^3+^ and EF1‐cyc, all aspartates can be unambiguously assigned, and as for all three *β*1 signals, significant displacements (+120–+260 ppb) downfield can be observed; we again conclude that Asp3, Asp5, and Asp9 are involved in binding. This also fits the suggested coordination from the MD simulation shown in Figure 6C.

**FIGURE 6:**
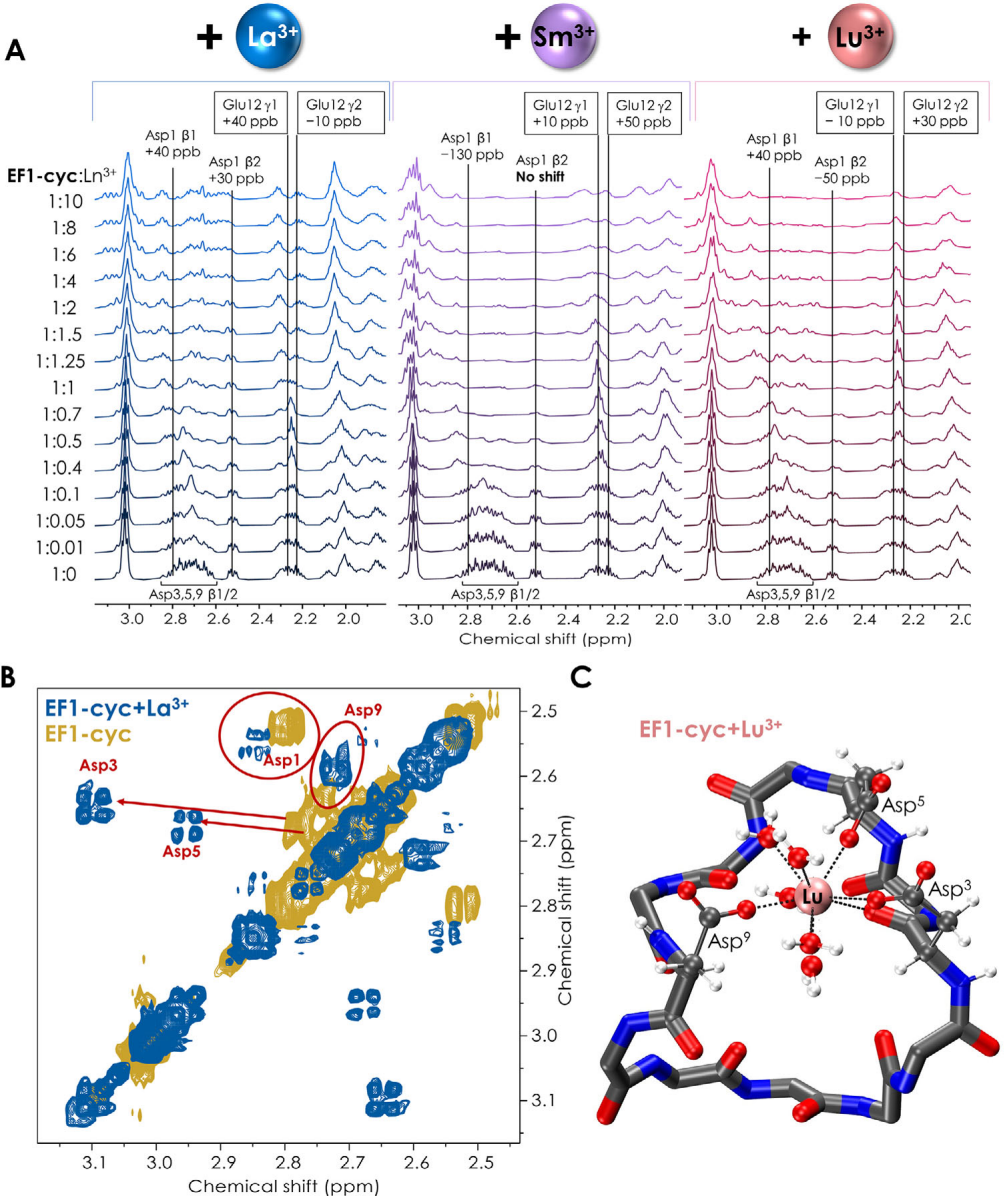
NMR and MD investigations of the binding behavior of EF1‐cyc. (A) Selected spectral region of ^1^H NMR LnCl_3_ (Ln  =  La^3+^, Sm^3+^, and Lu^3+^) to EF1‐cyc titration series (cf. Figures S54–S56) with Glu12*γ* and Asp1*β* resonances highlighted. (B) Selected region of superimposed TOCSY spectra of EF1‐cyc without and with excess of LaCl_3_. Experimental conditions for the NMR spectra: 600 MHz, H_2_O/D_2_O (9:1 *v/v*) + 0.003% TMSP‐*d_4_*, 30 mM MES‐*d_13_*, 100 mM KCl, pH 6.6. (C) A snapshot from the MD trajectory of EF1‐cyc with Lu^3+^ after 500 ns simulation time using the 12–6–4 LJ potential by Hay and coworkers [54] on Lu^3+^. The peptide backbone is shown in grey with the nitrogen (blue) and carbonyl (red) of the peptide bond highlighted.

For EF1‐R‐cyc, the observed shifts for Glu1 and the four Asp residues are listed in Tables S17–S19. Owing to severe signal overlap, tracking the complexation‐induced Asp signal displacements in the titration spectra is hardly feasible. Only the displacement of Asp8 β2, the most upfield‐shifted and best resolved among all Asp Hβ signals during the first five titration spectra, can be traced quite well in order to tell that Asp8 is likely involved in coordination (see discussion on neighboring Gly7 below). Although we cannot unambiguously assign the observed Asp spin systems to specific Asp residues, for La^3+^ and Lu^3+^, we can conclude from the chemical shift values found for Ln‐bound peptide (Tables S16–S18) that in either case, again two Asps are involved in Ln‐coordination. As inferred from the chemical shift values of the carboxymethylene ^1^H, the La^3+^ and Lu^3+^ complexes form without participation of Glu1, while in the titration with Sm^3+^, we observe notable downfield shifts for both Glu1 γ resonances (+260 ppb), indicating that in this case, Glu1 seems to be involved. In the Sm^3+^ complex, the Asp Hβ chemical shifts, however, are inconclusive as they all more or less indicate some complexation‐induced and/or paramagnetic shifts. As inferred from the Eu^3+^ luminescence lifetime (Table 1) as well as from the evidently reduced affinity of EF1‐R‐cyc, coordination by all five carboxylic groups can be ruled out. These findings could be explained by intramolecular dynamics, where the various carboxylic groups associate and dissociate rapidly on the NMR timescale and a maximum of three rather than four carboxylates are coordinated simultaneously. In this context of dynamic, that is, labile, coordination, it is also conceivable that some carboxylates are part of a second coordination sphere instead, for instance, upon hydrogen bonding to metal ion‐coordinating water molecules.

### 2.3 Investigation of the Lanthanide Selectivity of EF1‐cyc and EF1‐R‐cyc

While we did not observe a significant affinity boost from the micromolar range to, for example, the nanomolar range when going from the linear version of EF1 to its cyclic analogue EF1‐cyc, we were interested in finding out whether the cyclization might change the selectivity for a certain Ln. For EF1 and also for EF1‐R, we previously observed an affinity trend within the Ln series similar to the full protein LanM under similar experimental conditions, with the highest affinity for Nd^3+^, Sm^3+^, and Eu^3+^ [41, 44]. To evaluate this, we used a TRLFS competition experiment between Eu^3+^ and the respective Ln^3+^ (see Section S2.3). In contrast to the previously investigated linear peptides [41], the formation of only a 1:1 complex facilitated data analysis and allowed the extraction of conditional binding affinities rather than relative affinities. We also employed ITC to determine the Ln^3+^ preference of EF1‐cyc (all Lns, except Pm), reiterating the robustness of our TRLFS setup as we not only obtained exactly the same affinity trend (cf. Figure 7A,C), but also the individual values are in very good agreement (cf. Tables S4 and S7). As the TRLFS competition setup does require less peptide sample and is more time efficient compared to the ITC setup, for the investigation of EF1‐R‐cyc, solely this method was used.

**FIGURE 7:**
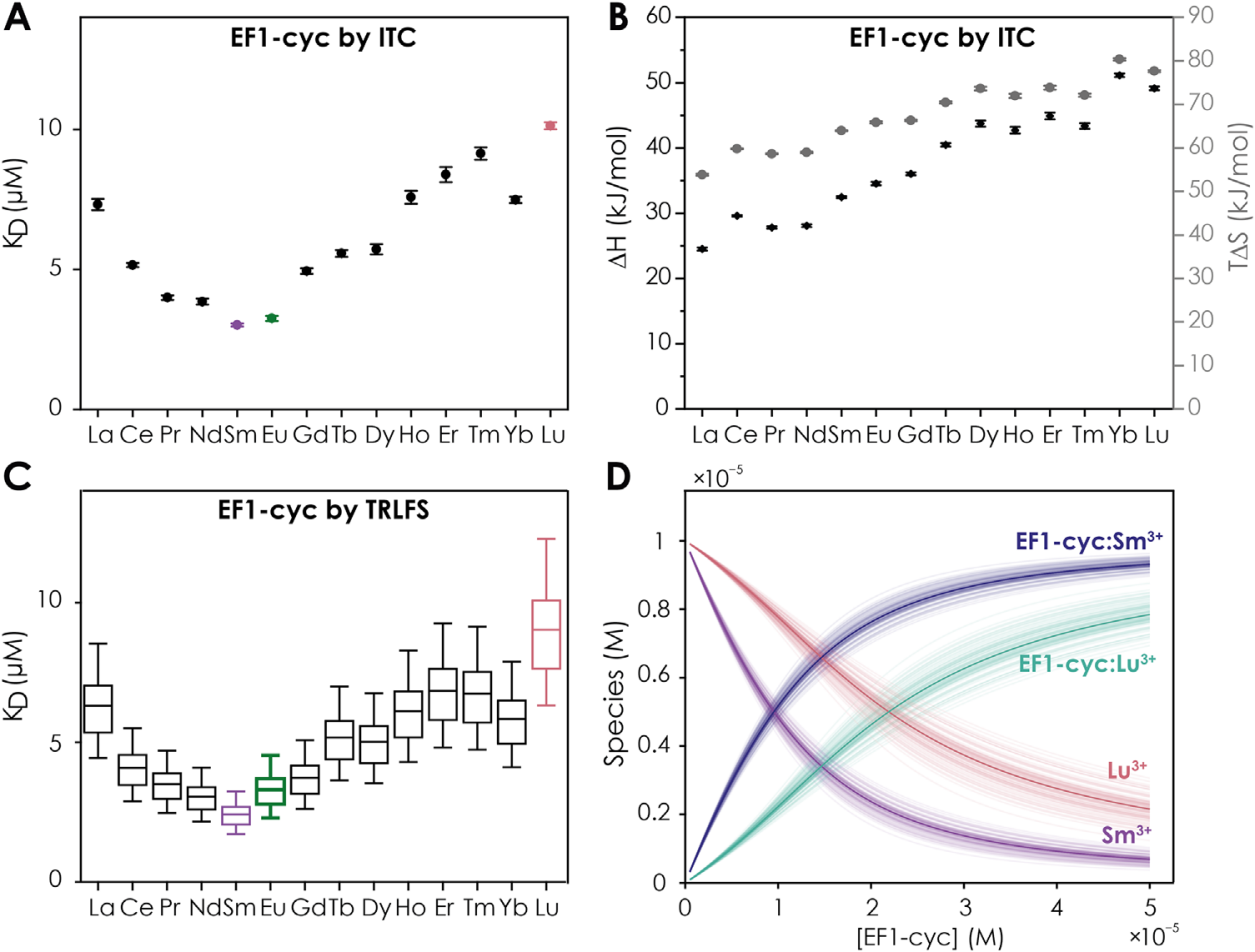
Lanthanide selectivity of EF1‐cyc. (A) Ln affinity of EF1‐cyc determined by ITC titrating 900 µM LnCl_3_ to 30 µM EF1‐cyc and (B) determined *∆H* (black symbols) and *T∆S* (gray symbols) values plotted in one graph with two *y*‐axes (cf. Tables S4 and Figures S8–S35). Titrations were performed in triplicates and evaluated globally; the uncertainties were obtained by 100 MC runs, and are shown as error bars. (C) Ln selectivity of EF1‐cyc determined by a TRLFS (*λ*_ex_(Eu) = 394 nm) competition experiment between the respective Ln^3+^ and Eu^3+^(cf. Table S7). The binding affinities are shown as a box plot (median and quartiles) and were obtained by PARAFAC in combination with an MC approach as described in Section S2.3. The corresponding MC distribution is given in Figure S41A. Experimental conditions for ITC and TRLFS: 10 mM MOPSO, 100 mM KCl, pH 6.6. (D) Modeled speciations [55] for the pair Sm^3+^ (10 µM) and Lu^3+^ (10 µM) and their complex formation with EF1‐cyc using the determined *K_D_* values from the competition experiment. At a 1:1:2 Sm^3+^:Lu^3+^:EF1‐cyc ratio (cf. Figure above at  20 µM EF1‐cyc), twice as much Sm^3+^ is peptide‐bound as Lu^3+^.

For both EF1‐cyc (Figure 7) and EF1‐R‐cyc (see Figure S41), we obtained a very similar trend over the Ln series, with the highest affinity in the middle of the series for Nd^3+^, Sm^3+^, and Eu^3+^ and the more or less pronounced outlier Yb^3+^, reflecting the observed trends for the linear analogues. However, the Yb^3+^ peculiarity seems to be more pronounced in the cyclic peptides than in the linear analogues [41]. Interestingly, in the literature, a deviation of the experimentally determined Gibbs free energy of hydration for Yb^3+^ from the overall trend within the Ln series is reported, which might explain the here observed dip [56, 57]. While the exact Lns with the highest affinity in the middle of the series often vary between different Ln‐binding peptides, the observation that the affinity does not follow the Ln contraction can be generally observed in different peptide systems [36, 58, 59]. While for LBTs it was shown that the alteration of an asparagine residue to an aspartate residue can increase the affinity for the heavier Lns, the highest affinity still remained for the Lns in the middle of the series [59]. Chiniforoush et al. explained the observed higher affinity in the middle of the series of their tested LBCPs by the following: A decrease in ionic radii for the Lns^3+^ following the Ln contraction leads to an already shorter Ln–O distance for the middle Lns and thus increased electrostatic attraction. For the heavier Lns, the lower affinity is then explained by the further reduction of the ionic radii with an increase in steric repulsion between the coordinating peptide residues, and therefore results in a weaker binding [36]. The *∆G* values determined by ITC (−29 to −32 kJ/mol) are consistent with the observed affinity trend, as expected from the relationship *∆G* = −*RT *ln *K* (see Table S4). The enthalpy and entropy values measured over the whole series show that the Ln‐binding is always endothermic and entropy driven (Figure 7B); however, the extent to which the respective effect comes into play varies within the series and roughly follows the Ln contraction. In a first approximation, both trends might be explained with the stripping of the hydration shell, as from La^3+^ to Lu^3+^, increasingly more energy is required for this step [57, 60, 61]. All in all, taking together our results and those from the literature, tuning the Ln selectivity of short peptides—especially cyclic ones—for a specific Ln selectivity remains a promising though challenging task and requires more work toward structural understanding. However, by determining the *K_D_* values for all Lns^3+^ (except Pm^3+^), speciation modeling for different separation scenarios becomes possible, allowing an assessment of whether the observed affinity differences are sufficient for effective separation. Figure 7D exemplarily shows such a modeled speciation for EF1‐cyc with the pair Sm^3^^+^/Lu^3^^+^. Although all *K_D_* values fall within one order of magnitude, the differences are sufficient to bind approximately twice as much Sm^3^^+^ as Lu^3^^+^ at a 10 µM:10 µM:20 µM Sm^3^^+^:Lu^3^^+^:EF1‐cyc ratio, providing an illustrative example of how even modest affinity differences could translate into measurable separation effects.

### 2.4 Influence of a Glycine Insertion Between Aspartic and Glutamic Acid in EF1‐cyc

The NMR investigations showed that the formally N‐ and C‐terminal AAs usually do not coordinate Ln^3+^, despite their carboxylate side chains (cf. Tables S14–S19). This, together with some hints from MD simulations, prompted us to separate those AAs by the insertion of an additional Gly, yielding the peptide EF1‐G‐cyc. MD simulations showed for the comparison between EF1‐cyc and EF1‐G‐cyc with La^3+^ and Eu^3+^ the involvement of the formally N‐terminal Asp1 after the insertion of Gly0 (see Tables S32 and S33). The idea was that by increasing the distance between the negatively charged Glu and Asp side chains with the inserted Gly, the electrostatic repulsion would decrease and, in turn, increase the number of coordinating residues and boost the affinity in comparison to EF1‐cyc. A further benefit of the insertion of Gly next to potentially binding AAs is that the Gly Hα resonances can be easily traced in the NMR in order to qualitatively probe changes in their local environment, addressing involvement of the neighboring AAs or to mirror conformational alterations. The full signal assignment for EF1‐G‐cyc (Figure S51, and Tables S12 and S13) and the titration series (Tables S60–S62), as well as a direct comparison of TOCSY spectra of EF1‐cyc and EF1‐G‐cyc with and without La^3+^, along with a more detailed discussion (stated with Figure S63), can be found in the Supporting Information. Against our initial hypothesis, the data indicate that introducing Gly0 did not facilitate the involvement of Asp1, nor Glu12 in Ln binding. Instead, the insertion of Gly0 seems to have more of an influence on the peptide backbone. Gly6, as the common feature in both peptides, correspondingly displays similar spectroscopic signatures, with signal displacements of identical direction (sign) and comparable magnitude (respecting the individual Ln's Lewis acidities), mirroring resemblance in binding motif and thermodynamic properties. Reversing the peptide sequence evidently alters the complexation behavior more or less in all aspects, that is, affinity, binding motif, and therefore in spatial relationships and inter‐AA interactions influencing NMR signatures as mirrored by the distinct local order domains (see above). Hα‐signal displacements of EF1‐R‐cyc's Gly7 are notably small (Table S26). Interconversion among various structures (see discussion on carboxylate dynamics above, and results from mass spectrometry below) and thus contributions of individual conformations likely averages or even cancels out, resulting in (observable) spectral effects.

CD titrations with EF1‐G‐cyc (see Figure S45) corroborate the findings from NMR. First, the metal‐free peptides EF1‐cyc and EF1‐G‐cyc are more similar to each other than either of them is to EF1‐R‐cyc. Second, the additional Gly0 causes the spectral characteristics associated with local order to diminish (Figures S52 and S53). This is in great agreement with the MD simulations of EF1‐G‐cyc in the absence of Lns (Figure S87, SI), which also show less organization than EF1‐cyc (Figure S86). Additionally, the observed changes upon addition of Ln^3+^ are much smaller. The band at around 222 nm still decreases with increasing Ln^3+^ concentration, but the band around 200 nm remains almost unchanged, except for Lu^3+^, where it decreases. Also, the ^1^H NMR NH region indicates alterations in the backbone just by simply adding one glycine, that is, for EF1‐cyc the NH resonances are almost evenly dispersed between *δ*_H_ 8.3 and 7.7 ppm, while for EF1‐G‐cyc that part of the spectrum is very different and more clustered.

To test for possible changes in affinity upon inserting an additional Gly, we performed ITC (Table S3, and Figures S6 and S7, SI) and TRLFS (Table S6 and Figure S38) experiments. We find a slightly higher affinity for EF1‐G‐cyc in comparison to EF1‐cyc (ITC: 2.4 ± 0.1 vs. 3.2 ± 0.1 µM; TRLFS: 1.5 ± 0.1 vs. 3.3 ± 0.2 µM). However, such minor differences should be interpreted with care, as both peptides still remain in the same affinity range. The observed range is comparable with that of the cyclic peptide LBCP2 recently designed by Chiniforoush et al., which was cyclized by the introduction of an intramolecular disulfide bond [36]. Interestingly, the fluorescence of Eu^3+^‐EF1‐G‐cyc exhibits several spectral peculiarities (Figure S38) compared to the other peptides. While the emission spectra obtained for the other two cyclic peptides appear rather featureless, the spectrum of EF1‐G‐cyc shows pronounced splitting patterns in the ^5^D_0_→^7^F_2_ transition. In addition, the F_4_ band appears more structured by comparison. Since the F_1_ band lacks any splitting patterns, an interpretation of the F_2_ splitting in terms of complex symmetry is not meaningful. Combined with the longer lifetime of the Eu^3+^–EF1‐G‐cyc complex, these observations indicate a more defined (i.e., less dynamic) complex that displaces a larger number of water molecules from the hydration shell of the Ln.

Lastly, the TRLFS competition experiment (Figure S42) demonstrated that the inserted Gly did not significantly alter the overall selectivity trend observed for EF1‐cyc. It follows that, while the structure seems to have changed in comparison to EF1‐cyc, the modification did not yield a worthwhile affinity or selectivity improvement.

### 2.5 Investigation of Cyclic Peptides in the Gas Phase With Advanced Mass Spectrometry Methods

Metal‐binding cyclic peptides are also found in nature, and their structural and isomeric forms upon metal binding are an ongoing field of research [62, 63]. Advanced mass spectrometry methods such as ion mobility spectrometry (IMS) can help us learn about structural changes of metal‐binding peptides without the need of great sample amounts or measuring time [64]. First, we investigated all peptides by classical electrospray ionization techniques using a LTQ XL Orbitrap mass spectrometer. An exemplary mass spectrum of EF1‐cyc in the presence of an excess of EuCl_3_ is shown in Figure S82A (see Figures S83A and S84A for the respective mass spectra of EF1‐R‐cyc and EF1‐G‐cyc). Here we observe in different intensities, depending on the instrument parameters (e.g., spray voltage and tube lens voltage), mono‐, di‐, and trications. As the observed effects are for all three types of cations very similar, for the sake of clarity, we focus in this study on the evaluation of the formed monocations. For all three peptides, the Eu^3+^‐complexes can be easily identified in reasonable intensities. After successful discrimination of the different species in the mass spectrometer, we performed collision‐induced dissociation (CID) experiments on isolated species to gain more insights into the structure, stability, and fragmentation channels. In this type of experiment, the respective ions are mass‐selected in the ion trap of the instrument and then excited in order to initiate fragmentation. The so obtained fragment ions are then analyzed in the orbitrap cell. Figure S82B shows the fragmentation of [EF1‐cyc+H]^+^, that is, *m/z* 1326.6376, isolated resonantly excited in the ion trap. Besides (multiple) water loss, we observe the typical fragmentation pattern for peptides dominated by the cleavage of peptide bonds (*b* and *y*‐type fragment ions) [65]. While we observed the same typical fragmentation pattern for the previously investigated peptides EF1 and EF1‐R [64], the *b*‐ and y‐type fragmentation of cyclic peptides is somewhat more complicated than for linear peptides. For the observation of fragments of cyclic peptides, at least two peptide bond cleavages are necessary: The first cleavage transfers the ring into a linear chain with one water molecule less than the respective linear peptide, and therefore most likely an acylium ion at the C‐terminus with the same mass as the cyclic peptide. Statistically, for a ring composed of *n* different AAs, *n* different linear chains with the same mass as the precursor ring are possible. The observed fragments can emerge from each of these chains by a second peptide bond cleavage. We calculated the different fragment species with mMass 5.0.0, an open‐source software tool for the fragmentation of both linear and cyclic peptides [66]. The matched fragments are summarized in Table S29. Both EF1‐R‐cyc and EF1‐G‐cyc fragmentation follow the same scheme, and the results for the monocations are shown in Figures S83B and S84B, Tables S30 and S31. While fragmentation of the monocations of all three peptides without a bound metal follows the expected fragmentation patterns, the fragmentation pattern of the Eu^3+^‐complexes is completely different. The fragmentation for the formed monocation [EF1‐cyc+Eu−2H]^+^ is shown in Figure S82C. As can be easily seen, instead of a complex fragmentation distribution due to multiple peptide bond cleavages, solely the loss of neutral molecules such as water and C_2_H_4_O (based on literature [67, 68], tentatively assigned to the loss of acetaldehyde from the threonine sidechain) can be observed. Thus, due to Eu^3+^ complexation, the formation of fragments due to backbone‐cleavage gets suppressed. Again, all three peptides show a very similar behavior, and respective mass spectra are shown in Figures S83C and S84C. All in all, the observations for the cyclic peptides match our previous results on the related linear peptides and can be explained with the stabilizing effect of the metal ion on the peptides in the gas phase [64].

As IMS is a powerful tool to identify and separate isomeric ions by differences in their collision cross sections (CCS) and can thereby help to gain an understanding of different species present in the gas phase, we performed such measurements for the two isomeric peptides EF1‐cyc and EF1‐R‐cyc. When comparing the same charge state, qualitative structural observations can be made as larger CCS are usually obtained for open, unfolded structures, while compact, folded structures have smaller CCS. In Figure 8A, the obtained mobilograms for the monocations [EF1‐cyc+H]^+^ and [EF1‐R‐cyc+H]^+^ are shown. Interestingly, we observe a slightly smaller CCS for the reversed sequence in comparison to the forward sequence—a result we also observed previously when investigating the di‐ and trications of the linear versions [64]. Furthermore, EF1‐R‐cyc shows at least three overlapping peaks and therefore multiple structural isomers. The resulting mobilograms of the monocations of the Eu^3+^‐complexes, [EF1‐cyc+Eu−2H]^+^ and [EF1‐R‐cyc+Eu−2H]^+^, are shown in Figure 8B (cf. Figure S85 for IMS of the dications). Two main observations can be derived from the obtained data. First, in contrast to the linear peptides EF1 and EF1‐R [64], the complexation of Eu^3+^, as expected due to the lower flexibility, does not yield a significantly smaller CCS and thus a more compact structure than the metal‐free peptide. Second, for EF1‐R‐cyc, the mobilogram shows the presence of at least six structural isomers with overlapping CCS, while for EF1‐cyc, only a slight shoulder in the mobilogram can be observed. As of now, we cannot explain the striking difference by IMS between EF1‐cyc and EF1‐R‐cyc when bound Eu^3+^. However, the NMR spectroscopic peculiarities discussed for the EF1‐R‐cyc complex of Sm^3+^ in solution (see above), speculating on pronounced intramolecular carboxylate exchange dynamics, may relate to the multiple peptide complex isomers inferred from IMS in the gas phase.

**FIGURE 8:**
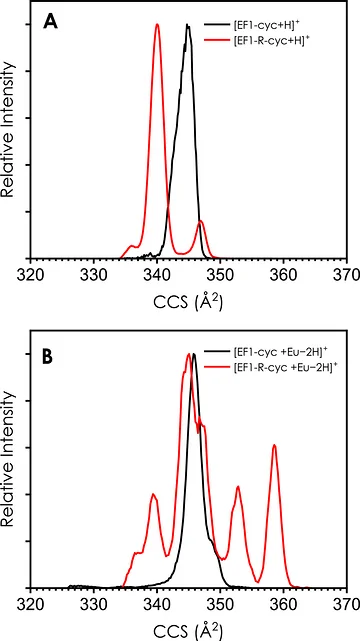
(A) Mobilograms of [EF1‐cyc+H]^+^ and [EF1‐R‐cyc+H]^+^. (B) Mobilograms of [EF1‐cyc+Eu−2H]^+^ and [EF1‐R‐cyc+Eu−2H]^+^.

## 3 Conclusion

In this study, head‐to‐tail cyclized LanM‐inspired peptides were investigated for the first time with respect to their Ln affinity, selectivity, and structural characteristics. Using CD and NMR spectroscopy accompanied by MD simulations, we demonstrated that head‐to‐tail cyclization leads to structural changes and, consequently, to enhanced pre‐organization of the peptide backbone compared to the open chain peptides.

Importantly, affinities toward the complete Ln series (excluding Pm^3+^) were determined for the first time for these cyclic systems, revealing small but significant differences across the series. Complementary ITC and TRLFS experiments demonstrated that cyclization does not significantly alter the established Ln selectivity trends known from LanM and linear LanM‐derived peptides.

In comparison to their linear C‐terminal capped analogues, the cyclic peptides exhibited substantially improved binding behavior, highlighting the beneficial effect of backbone cyclization. Additionally, by cyclization we also observed a slight affinity increase for the uncapped forward sequence EF1, while we observe a drastic decrease in affinity for the reversed sequence EF1‐R, highlighting the crucial role of the C‐terminal succinate‐like binding motif in open chain peptides for effective Ln‐coordination. For the actinide Cm^3+^, we found a factor 2 higher affinity in comparison to Eu^3+^.

Furthermore, the insertion of an additional glycine residue to separate two negatively charged side chains was shown to markedly influence the peptide‐Ln^3+^‐complex structure, while not significantly enhancing the Ln affinity or changing the selectivity. Notably, our NMR data clearly demonstrated the usefulness of such glycine insertions next to potentially coordinating AAs to probe the complexation via ^1^H NMR spectroscopy. Finally, this work highlights the potential of advanced mass spectrometry techniques as powerful complementary tools for the investigation of cyclic Ln‐binding peptides.

## Author Contributions

**S.M.G.T**., **L.J.D.,** and **B.D**. conceptualized the idea, **S.M.G.T**. wrote the initial draft of the manuscript. **L.J.D., B.D.,** and **P.W**. acquired funding. **S.M.G.T**, **J.K**., **B.D**., and **P.W**. performed experiments and analyzed data. **S.T**. performed molecular dynamic simulations and provided the interpretation. The necessary resources and infrastructure for this work were provided by **L.J.D**., **B.D**., and **R.S**. All authors were involved in reviewing and editing this manuscript.

## Funding

European Research Council (ERC), ERC Starting Grant LANTHANOPHOR (945846); Deutsche Forschungsgemeinschaft (DFG, German Research Foundation), Collaborative Research Centre “4f for Future” (CRC 1573, project number 471424360, project A02); P.W. is grateful to KIT, Land B.‐W. and DFG for the funding of a Waters Cyclic IMS system under Art. 91b GG. Bundesministerium für Bildung und Forschung (BMFTR, formerly BMBF), PepTight project (031B1122A).

## Conflicts of Interest

The authors declare no conflicts of interest.

## Supporting information




**Supporting File 1**: chem71438‐sup‐0001‐SuppMat.pdf

## Data Availability

Data supporting the findings of this study are available within the paper and its Supporting Information. Additionally, raw data files from NMR (.fid), TRLFS (.sif), ITC (.itc), CD (.jws) and MS (.raw) experiments, along with .xyz and .pdb files of the molecular dynamics simulations are available in the RODARE repository (DOI: 10.14278/rodare.4287).

## References

[1] S. M. Jowitt , “Mineral Economics of the Rare‐Earth Elements,” MRS Bulletin 47 (2022): 276–282, 10.1557/s43577-022-00289-3.

[2] V. Balaram , “Rare Earth Elements: A Review of Applications, Occurrence, Exploration, Analysis, Recycling, and Environmental Impact,” Geoscience Frontiers 10 (2019): 1285–1303, 10.1016/j.gsf.2018.12.005.

[3] T. McNulty , N. Hazen , and S. Park , “Processing the Ores of Rare‐Earth Elements,” MRS Bulletin 47 (2022): 258–266, 10.1557/s43577-022-00288-4.

[4] Q. Ye , D. Wang , and N. Wei , “Engineering Biomaterials for the Recovery of Rare Earth Elements,” Trends in Biotechnology 42 (2024): 575–590, 10.1016/j.tibtech.2023.10.011. 37985335

[5] A. Pol , T. R. M. Barends , A. Dietl , et al., “Rare Earth Metals Are Essential for Methanotrophic Life in Volcanic Mudpots,” Environmental Microbiology 16 (2014): 255–264, 10.1111/1462-2920.12249. 24034209

[6] Y. Hibi , K. Asai , H. Arafuka , M. Hamajima , T. Iwama , and K. Kawai , “Molecular Structure of La^3+^‐Induced Methanol Dehydrogenase‐Like Protein in Methylobacterium Radiotolerans,” Journal of Bioscience and Bioengineering 111 (2011): 547–549, 10.1016/j.jbiosc.2010.12.017. 21256798

[7] T. Nakagawa , R. Mitsui , A. Tani , et al., “A Catalytic Role of XoxF1 as La^3+^‐Dependent Methanol Dehydrogenase in Methylobacterium extorquens Strain AM1,” PLoS ONE 7 (2012): e50480, 10.1371/journal.pone.0050480. PMC3507691 23209751

[8] J. A. Cotruvo Jr. , E. R. Featherston , J. A. Mattocks , J. V. Ho , and T. N. Laremore , “Lanmodulin: A Highly Selective Lanthanide‐Binding Protein From a Lanthanide‐Utilizing Bacterium,” Journal of the American Chemical Society 140 (2018): 15056–15061, 10.1021/jacs.8b09842. 30351021

[9] J. A. Mattocks , J. J. Jung , C.‐Y. Lin , et al., “Enhanced Rare‐Earth Separation With a Metal‐Sensitive Lanmodulin Dimer,” Nature 618 (2023): 87–93, 10.1038/s41586-023-05945-5. PMC10232371 37259003

[10] C.‐E. Wegner , L. Gorniak , S. Riedel , M. Westermann , and K. Küsel , “Lanthanide‐Dependent Methylotrophs of the Family Beijerinckiaceae: Physiological and Genomic Insights,” Applied and Environmental Microbiology 86 (2019): e01830, 10.1128/AEM.01830-19. PMC6912076 31604774

[11] Y. Fujitani , T. Shibata , and A. Tani , “A Periplasmic Lanthanide Mediator, Lanmodulin, in Methylobacterium Aquaticum Strain 22A,” Frontiers in Microbiology 13 (2022): 921636, 10.3389/fmicb.2022.921636. PMC9260416 35814700

[12] J. L. Hemmann , P. Keller , L. Hemmerle , et al., “Lanpepsy is a Novel Lanthanide‐Binding Protein Involved in the Lanthanide Response of the Obligate Methylotroph Methylobacillus flagellatus,” Journal of Biological Chemistry 299 (2023): 102940, 10.1016/j.jbc.2023.102940. PMC9988556 36702252

[13] W. B. Larrinaga , J. J. Jung , C.‐Y. Lin , A. K. Boal , and J. A. Cotruvo , “Modulating Metal‐Centered Dimerization of a Lanthanide Chaperone Protein for Separation of Light Lanthanides,” Proceedings of the National Academy of Sciences 121 (2024): e2410926121, 10.1073/pnas.2410926121. PMC11551332 39467132

[14] S. M. Gutenthaler , S. Tsushima , R. Steudtner , et al., “Lanmodulin Peptides – Unravelling the Binding of the EF‐Hand Loop Sequences Stripped From the Structural Corset,” Inorganic Chemistry Frontiers 9 (2022): 4009–4021. 10.1039/d2qi00933a PMC9362731 36091973

[15] G. Verma , J. Hostert , A. A. Summerville , et al., “Investigation of Rare Earth Element Binding to a Surface‐Bound Affinity Peptide Derived From EF‐Hand Loop I of Lanmodulin,” ACS Applied Materials & Interfaces 16 (2024): 16912–16926, 10.1021/acsami.3c17565. PMC10995902 38527460

[16] L. Johnson , B. L. Schneider , H. Mithaiwala , M. D. Green , J. N. Renner , and C. E. Duval , “Electrospun Membranes Modified With Lanmodulin‐Derived Peptides for Lanthanide Adsorption,” ACS Applied Engineering Materials 2 (2024): 2442–2453, 10.1021/acsaenm.4c00510.

[17] L. Johnson and C. E. Duval , “Rare‐Earth Element Adsorption to Membranes Functionalized With Lanmodulin‐Derived Peptides,” Langmuir 41 (2025): 9581–9589, 10.1021/acs.langmuir.5c00969. 40171965

[18] S. Asaei , G. Verma , N. S. Sinclair , and J. N. Renner , “Electrochemical Biosensing of Cerium With a Tyrosine‐Functionalized EF ‐Hand Loop Peptide,” AIChE Journal (2024): e18620, 10.1002/aic.18620.

[19] M. Zhang , L. Tian , D. Villa‐Gomez , et al., “Bio‐Inspired Metal Extraction: Tailoring Peptides for Highly Selective Scandium Recovery,” Separation and Purification Technology 354 (2025): 128640, 10.1016/j.seppur.2024.128640.

[20] H. Sree , G. Swarup , S. Gupta , and K. Pushpavanam , “Gravity‐Driven Separation for Enrichment of Rare Earth Elements Using Lanthanide Binding Peptide‐Immobilized Resin,” ACS Applied Bio Materials 7 (2024): 7828–7837, 10.1021/acsabm.3c01280. 38685483

[21] G. Techert , B. Drobot , R. Braun , et al., “Application of Phage Surface Display for the Identification of Eu^3+^‐Binding Peptides,” Frontiers in Bioengineering and Biotechnology 13 (2025): 1508018, 10.3389/fbioe.2025.1508018. PMC11882594 40051836

[22] R. Klassen , A. Heider , H. Kugler , M. Groll , and C. Zeymer , “A Luminescence‐Based Screening Platform for Lanthanide‐Binding Peptides and Proteins,” ACS Chemical Biology 20 (2025): 2897–2906, 10.1021/acschembio.5c00670. PMC12723673 41248128

[23] L. Ancel , A. Niedźwiecka , C. Lebrun , C. Gateau , and P. Delangle , “Rational Design of Lanthanide Binding Peptides,” Comptes Rendus Chimie 16 (2013): 515–523, 10.1016/j.crci.2012.12.002.

[24] E. Falcone , E. Mathieu , and C. Hureau , “Lanthanide(iii)‐Binding Peptides and Proteins: Coordination Properties and Applications,” Chemical Society Reviews 54 (2025): 9245–9288, 10.1039/D4CS01148A. 40980867

[25] P. MacManus , G. Szabo , W. Hogue , and J. Marsden , “Terbium Luminescence in Synthetic Peptide Loops From Calcium‐binding Proteins With Different Energy Donors,” Journal of Biological Chemistry 265 (1990): 10358–10366, 10.1016/S0021-9258(18)86954-X. 2355005

[26] C. W. Hogue , J. P. MacManus , D. Banville , and A. G. Szabo , “Comparison of Terbium (III) Luminescence Enhancement in Mutants of EF Hand Calcium Binding Proteins,” Journal of Biological Chemistry 267 (1992): 13340–13347, 10.1016/S0021-9258(18)42216-8. 1618836

[27] K. J. Franz , M. Nitz , and B. Imperiali , “Lanthanide‐Binding Tags as Versatile Protein Coexpression Probes,” Chembiochem 4 (2003): 265–271, 10.1002/cbic.200390046. 12672105

[28] L. J. Martin , M. J. Hähnke , M. Nitz , et al., “Double‐Lanthanide‐Binding Tags: Design, Photophysical Properties, and NMR Applications, Photophysical Properties, and NMR Applications,” Journal of the American Chemical Society 129 (2007): 7106–7113, 10.1021/ja070480v. 17497862

[29] M. Nitz , K. J. Franz , R. L. Maglathlin , and B. Imperiali , “A Powerful Combinatorial Screen to Identify High‐Affinity Terbium(III)‐Binding Peptides,” Chembiochem 4 (2003): 272–276, 10.1002/cbic.200390047. 12672106

[30] M. Nitz , M. Sherawat , K. J. Franz , E. Peisach , K. N. Allen , and B. Imperiali , “Structural Origin of the High Affinity of a Chemically Evolved Lanthanide‐Binding Peptide,” Angewandte Chemie 116 (2004): 3768–3771, 10.1002/ange.200460028. 15248272

[31] P. K. R. Tay , A. Manjula‐Basavanna , and N. S. Joshi , “Repurposing Bacterial Extracellular Matrix for Selective and Differential Abstraction of Rare Earth Elements,” Green Chemistry 20 (2018): 3512–3520, 10.1039/C8GC01355A.

[32] R. A. Scheel , J. K. Sahoo , L. D. Morton , et al., “Reusable Silk‐Based Mesoporous Membranes for Recovery of Rare‐Earth Elements,” ACS ES&T Engineering 4 (2024): 2135–2144, 10.1021/acsestengg.4c00184.

[33] C. Berthomieu , M. Martin , J. Aupiais , et al., “Characterizing the Lanthanide‐Binding Tag Grafted at Calmodulin Site 1: Affinity, Selectivity, and Coordination Properties,” European Journal of Inorganic Chemistry 29 (2026): e202500468.

[34] T. Hatanaka , A. Matsugami , T. Nonaka , et al., “Rationally Designed Mineralization for Selective Recovery of the Rare Earth Elements,” Nature Communications 8 (2017): 15670, 10.1038/ncomms15670. PMC5458567 28548098

[35] Y. Hosokawa , A. Oshima , T. Hatanaka , and N. Ishida , “Improved Recovery and Selectivity of Lanthanide‐Ion‐Binding Cyclic Peptide Hosts by Changing the Position of Acidic Amino Acids,” Minerals 12 (2022): 148.

[36] S. Chiniforoush , O. Fizer , A. de Bettencourt‐Dias , and D. C. Cantu , “Cyclic Peptides for Lanthanide Binding,” Journal of Physical Chemistry B 129 (2025): 10690–10698, 10.1021/acs.jpcb.5c03650. 41055890

[37] C. S. Bonnet , P. H. Fries , S. Crouzy , et al., “A Gadolinium‐Binding Cyclodecapeptide With a Large High‐Field Relaxivity Involving Second‐Sphere Water,” Chemistry—A European Journal 15 (2009): 7083–7093, 10.1002/chem.200900636. 19533726

[38] A. Zorzi , K. Deyle , and C. Heinis , “Cyclic Peptide Therapeutics: Past, Present and Future,” Current Opinion in Chemical Biology 38 (2017): 24–29, 10.1016/j.cbpa.2017.02.006. 28249193

[39] L. Liu , L. Yang , S. Cao , et al., “CyclicPepedia: A Knowledge Base of Natural and Synthetic Cyclic Peptides,” Briefings in Bioinformatics 25 (2024): bbae190, 10.1093/bib/bbae190. PMC11056021 38678388

[40] D. P. Slough , S. M. McHugh , and Y.‐S. Lin , “Understanding and Designing Head‐to‐Tail Cyclic Peptides,” Biopolymers 109 (2018): e23113, 10.1002/bip.23113. PMC6135719 29528114

[41] S. M. Gutenthaler‐Tietze , J. Kretzschmar , S. Tsushima , R. Steudtner , B. Drobot , and L. J. Daumann , “Reversing Lanmodulin's Metal‐Binding Sequence in Short Peptides Surprisingly Increases the Lanthanide Affinity,” Angewandte Chemie International Edition 64 (2025): e202510453, 10.1002/anie.202510453. PMC12603986 40985823

[42] B. Drobot , R. Steudtner , J. Raff , G. Geipel , V. Brendler , and S. Tsushima , “Combining luminescence Spectroscopy, Parallel Factor Analysis and Quantum Chemistry To Reveal Metal Speciation—A Case Study of Uranyl( VI ) Hydrolysis,” Chemical Science 6 (2015): 964–972, 10.1039/C4SC02022G. PMC5811152 29560182

[43] B. Drobot , A. Bauer , R. Steudtner , et al., “Speciation Studies of Metals in Trace Concentrations: the Mononuclear Uranyl(VI) Hydroxo Complexes,” Analytical Chemistry 88 (2016): 3548–3555, 10.1021/acs.analchem.5b03958. 26977534

[44] H. Singer , B. Drobot , C. Zeymer , R. Steudtner , and L. J. Daumann , “Americium Preferred: Lanmodulin, a Natural Lanthanide‐Binding Protein Favors an Actinide Over Lanthanides,” Chemical Science (2021): 15581–15587, 10.1039/D1SC04827A. PMC8654097 35003587

[45] G. J.‐P. Deblonde , J. A. Mattocks , H. Wang , et al., “Characterization of Americium and Curium Complexes With the Protein Lanmodulin: A Potential Macromolecular Mechanism for Actinide Mobility in the Environment,” Journal of the American Chemical Society 143 (2021): 15769–15783, 10.1021/jacs.1c07103. 34542285

[46] S. Özçubukçu , K. Mandal , S. Wegner , M. P. Jensen , and C. He , “Selective Recognition of Americium by Peptide‐Based Reagents,” Inorganic Chemistry 50 (2011): 7937–7939. 10.1021/ic201094e 21819087

[47] T. Hatanaka , N. Kikkawa , A. Matsugami , Y. Hosokawa , F. Hayashi , and N. Ishida , “The Origins of Binding Specificity of a Lanthanide Ion Binding Peptide,” Scientific Reports 10 (2020): 19468, 10.1038/s41598-020-76527-y. PMC7656248 33173124

[48] M. Hellman , H. Tossavainen , P. Rappu , J. Heino , and P. Permi , “Characterization of Intrinsically Disordered Prostate Associated Gene (PAGE5) at Single Residue Resolution by NMR Spectroscopy,” PLoS ONE 6 (2011): e26633, 10.1371/journal.pone.0026633. PMC3206799 22073178

[49] D. S. Wishart , “Interpreting Protein Chemical Shift Data,” Progress in Nuclear Magnetic Resonance Spectroscopy 58 (2011): 62–87, 10.1016/j.pnmrs.2010.07.004. 21241884

[50] D. S. Wishart , B. D. Sykes , and F. M. Richards , “Simple Techniques for the Quantification of Protein Secondary Structure by ^1^H NMR Spectroscopy,” FEBS Letters 293 (1991): 72–80, 10.1016/0014-5793(91)81155-2. 1959674

[51] W. C. Johnson , “Secondary Structure of Proteins through Circular Dichroism Spectroscopy,” Annual Review of Biophysics and Biophysical Chemistry (1988): 145–166, 10.1146/annurev.bb.17.060188.001045. 3293583

[52] Y. Wei , A. A. Thyparambil , and R. A. Latour , “Protein Helical Structure Determination Using CD Spectroscopy for Solutions With Strong Background Absorbance From 190 to 230 nm,” Biochimica et Biophysica Acta (BBA)—Proteins and Proteomics 1844 (2014): 2331–2337, 10.1016/j.bbapap.2014.10.001. PMC4395514 25308773

[53] Z. Li , L. F. Song , P. Li , and K. M. Jr. Merz , “Parametrization of Trivalent and Tetravalent Metal Ions for the OPC3, OPC, TIP3P‐FB, and TIP4P‐FB Water Models,” Journal of Chemical Theory and Computation 17 (2021): 2342–2354, 10.1021/acs.jctc.0c01320. PMC8173366 33793233

[54] P. Kantakevičius , C. Mathiah , L. O. Johannissen , and S. Hay , “Chelator‐Based Parameterization of the 12‐6‐4 Lennard‐Jones Molecular Mechanics Potential for More Realistic Metal Ion–Protein Interactions,” Journal of Chemical Theory and Computation 18 (2022): 2367–2374. 10.1021/acs.jctc.1c00898 PMC9171819 35319190

[55] D. S. Smith , “Solution of Simultaneous Chemical Equilibria in Heterogeneous Systems: Implementation in Matlab,” Chemistry Faculty Publications 14 (2019), https://scholars.wlu.ca/chem_faculty/14 .

[56] Y. Marcus , “Thermodynamics of Solvation of Ions. Part 5.—Gibbs Free Energy of Hydration at 298.15 K,” Journal of the Chemical Society, Faraday Transactions 87 (1991): 2995–2999, 10.1039/FT9918702995.

[57] J. Ciupka , X. Cao‐Dolg , J. Wiebke , and M. Dolg , “Computational Study of Lanthanide(III) Hydration,” Physical Chemistry Chemical Physics 12 (2010): 13215, 10.1039/c0cp00639d. 20820581

[58] L. E. Ortuno Macias , F. Jiménez‐Ángeles , J. G. Marmorstein , et al., “Lanthanide Binding Peptide Surfactants at Air–Aqueous Interfaces for Interfacial Separation of Rare Earth Elements,” Proceedings of the National Academy of Sciences 121 (2024): e2411763121, 10.1073/pnas.2411763121. PMC11670062 39700142

[59] S. S. Kt , B. Qiao , J. G. Marmorstein , et al., “The Role of Asparagine as a Gatekeeper Residue in the Selective Binding of Rare Earth Elements by Lanthanide‐Binding Peptides,” Chemistry—A European Journal 31 (2025): e202501318, 10.1002/chem.202501318. PMC12160965 40312258

[60] M. Duvail , R. Spezia , and P. Vitorge , “A Dynamic Model to Explain Hydration Behaviour Along the Lanthanide Series,” Chemphyschem 9 (2008): 693–696, 10.1002/cphc.200700803. 18338343

[61] J. Zhang , N. Heinz , and M. Dolg , “Understanding Lanthanoid(III) Hydration Structure and Kinetics by Insights From Energies and Wave Functions,” Inorganic Chemistry 53 (2014): 7700–7708, 10.1021/ic500991x. 24960079

[62] P. Comba , A. Eisenschmidt , L. R. Gahan , et al., “Is CuII Coordinated to Patellamides Inside Prochloron Cells?,” Chemistry—A European Journal 23 (2017): 12264–12274, 10.1002/chem.201700895. 28339125

[63] Y. Li , Y. Ma , Y. Xia , et al., “Discovery and Biosynthesis of Tricyclic Copper‐Binding Ribosomal Peptides Containing Histidine‐to‐Butyrine Crosslinks,” Nature Communications 14 (2023): 2944, 10.1038/s41467-023-38517-2. PMC10206099 37221219

[64] S. M. Gutenthaler‐Tietze , L. J. Daumann , and P. Weis , “Lanthanide‐Binding Lanmodulin‐Based Peptides: Insights From Advanced Mass Spectrometry Techniques,” European Journal of Inorganic Chemistry 28 (2025): e202500258, 10.1002/ejic.202500258.

[65] P. Roepstorff and J. Fohlman , “Letter to the Editors,” Biological Mass Spectrometry 11 (1984): 601, 10.1002/bms.1200111109.

[66] T. H. J. Niedermeyer and M. Strohalm , “mMass as a Software Tool for the Annotation of Cyclic Peptide Tandem Mass Spectra,” PLoS ONE 7 (2012): e44913, 10.1371/journal.pone.0044913. PMC3441486 23028676

[67] N. G. Rumachik , G. C. McAlister , J. D. Russell , D. J. Bailey , C. D. Wenger , and J. J. Coon , “Characterizing Peptide Neutral Losses Induced by Negative Electron‐Transfer Dissociation (NETD),” Journal of the American Society for Mass Spectrometry 23 (2012): 718–727, 10.1007/s13361-011-0331-5. PMC3371390 22290482

[68] Y. Liang , P. Neta , X. Yang , and S. E. Stein , “Collision‐Induced Dissociation of Deprotonated Peptides. Relative Abundance of Side‐Chain Neutral Losses, Residue‐Specific Product Ions, and Comparison With Protonated Peptides,” Journal of the American Society for Mass Spectrometry 29 (2018): 463–469, 10.1007/s13361-017-1842-5. 29143271

