# Supplementary material for: Influence of Head‐to‐Tail Cyclization of Lanmodulin‐Inspired Peptides on Lanthanide Affinity and Structure
Source: Chemistry. 2026 Jul 25;32(40):e71438. doi: 10.1002/chem.71438 (PMC13716023; doi:10.1002/chem.71438)
Supplement: Supplementary file 1 [file CHEM-32-e71438-s001.pdf]

Supporting Information

Influence of Head-to-Tail Cyclization of Lanmodulin-Inspired Peptides on Lanthanide Affinity and Structure

Sophie M. Gutenthaler-Tietze[a], Jerome Kretzschmar[b], Satoru Tsushima[b,c], Patrick Weis[d], Robin Steudtner[b], Björn Drobot*[b], Lena J. Daumann*[a]*

[a] Lehrstuhl für Bioanorganische Chemie, Heinrich-Heine-Universität Düsseldorf, Universitätsstraße 1, 40225 Düsseldorf (Germany)
[b] Institute of Resource Ecology, Helmholtz-Zentrum Dresden-Rossendorf e.V., Bautzner Landstraße 400, 01328 Dresden (Germany)
[c] Institute of Integrated Research, Institute of Science Tokyo, 152-8550, Tokyo (Japan)
[d] Institute for Physical Chemistry II, Karlsruher Institut für Technologie, Fritz-Haber Weg 2, 76131 Karlsruhe (Germany)

Table of Content

1 Solvents and Chemical Reagents 3

1.1. Cyclic Peptides 3

2 Lanthanide-binding Studies 4

2.1 Metal Stocks 4

2.2 Isothermal Titration Calorimetry (ITC) 4

2.3 Time-resolved Laser-induced Fluorescence Spectroscopy (TRLFS) 4

2.4 Circular dichroism (CD) Spectroscopy 5

2.5 Nuclear Magnetic Resonance (NMR) Spectroscopy 6

2.6 Lanthanide-binding studies in the gas phase *via* mass spectrometry 6

3 Molecular dynamics (MD) simulations 7

4 Supplementary Tables and Figures 8

4.1 Supplementary ITC Data 8

4.2 Supplementary TRLFS Data 26

4.3 Supplementary CD Data 29

4.4 Supplementary NMR Data 30

4.5 Supplementary Mass Spectrometry Data 57

4.6 Supplementary MD Data 66

5 Data Availability 67

6 References 67

1 Solvents and Chemical Reagents

Experiments were performed in ultrapure water (type 1, pH 5.6, 18.2 MΩ·cm at 25 °C) if not stated otherwise. For this, demineralised water was further purified using a Synergy® UV system from *Merck* Millipore®. Unless stated otherwise, chemicals were used as supplied and handled according to potential hazards and toxicity**.**

1.1. Cyclic Peptides

Table S1 Molecular weights and amino acid sequences of head-to-tail cyclised lanmodulin-inspired peptides.

Peptide	Molecular Weight (g/mol)	Sequence
EF1-cyc	1327.40	c(DPDKDGTIDLKE)
EF1-R-cyc	1327.40	c(EKLDITGDKDPD)
EF1-G-cy	1384.46	c(GDPDKDGTIDLKE)

Peptides were ordered by *GenScript* and used as delivered by preparing 2 mg/mL gross weight stocks in water. The target peptide concentration was calculated by taking the analytical results provided by *GenScript* (see Table S2) into account using equation (1).

$$\left(\frac{grossweightconc.\left[\frac{mg}{mL}\right]\cdot AAcontent[\%]\cdot LCMSpurity[\%]}{molecularweight\left[\frac{mg}{mmol}\right]}\right)\cdot 10^{6} = targetpeptideconc.\left[\frac{\mu mol}{L}\right] \qquad (1)$$

Table S2 Purity and calculated target peptide concentration in µM of the peptides ordered from GenScript.

Peptide	AA content (%)	HPLC purity (%)	LCMS purity (%)	Target peptide conc. (µM)
EF1-cyc	68.1	98.1	99.9	1025
EF1-R-cyc	62.8	94.2	96.6	891
EF1-G-cyc	80.2	95.0	95.2	1102

2 Lanthanide-binding Studies

2.1 Metal Stocks

Metal-binding experiments were performed with $LnCl_3$ which were prepared from $LnCl_3 \cdot n\,H_2O$ salts as 10 mM stock solutions in ultrapure water (type 1, pH 5.6, 18.2 MΩ·cm at 25 °C) and stored at −20 °C until usage.

2.2 Isothermal Titration Calorimetry (ITC)

All ITC experiments were performed on a *Malvern Panalytical* MicroCal PEAQ-ITC instrument. Titrations were performed over 39 steps (first injection: 0.4 µL, residual injections: 1 µL) in time intervals of 150 s. The reference cell was filled with water, the stirring speed set to 750 rpm and the initial delay to 60 s. Before the titration was started, the sample cell was equilibrated with the buffer and the syringe with the used $LnCl_3$ solution. Analogue titrations were carried out using the same settings but with buffer in the cell, and these were used as background measurement. Experiments were either performed as triplicates with a 30 µM peptide solution in the sample cell and a 900 µM $LnCl_3$ solution in the syringe or as replicates with varying cell concentration (peptide concentration: 30 µM, 45 µM, 60 µM), but constant $LnCl_3$ concentration (900 µM) in the syringe. The obtained data was analysed with MATLAB using a workflow in which the raw ITC data were pre-treated to reduce noise using singular value decomposition (SVD) as described elsewhere[1]. To increase the robustness of the fit, the triplicates or replicates were evaluated globally[2] using a Monte Carlo approach.[3] The analysis was performed without any restrictions and the obtained N-values were used as concentration correction factor in the TRLFS data analysis. The Gibbs free energy (ΔG) and entropy contribution ($T\Delta S$) were calculated from the obtained enthalpy (ΔH) and affinity (K_D) values (Table S3).

2.3 Time-resolved Laser-induced Fluorescence Spectroscopy (TRLFS)

Titration experiments were performed in MOPSO buffer (for Eu^{3+} experiments: 10 mM, 100 mM KCl, pH 6.6; for Cm^{3+} experiments: 10 mM, 100 mM KCl, pH 6.0) at 25 °C in stirred (1200 rpm) 2 mL or 4 mL 10 mm pathlength *Hellma Analytics* quartz cells equipped with a screw cap and septum. The cuvette was placed in a cuvette holder which was connected *via* a light guide to a spectrograph (*Andor*, SR-303i-A). For recording the spectra an intensified CCD (Andor iStar, DH320T-18U-63) was used. The excitation wavelength (*Ekspla*, NT230, ~5 ns pulse) for Eu^{3+} was set to 394 nm (grating: 300 l/mm) and for Cm^{3+} to 396 nm (grating: 600 l/mm), respectively. All data sets were analysed using MATLAB. For this, the raw data were loaded using an adopted version of the *Andor* sif-reader.[4] Detailed data analysis was performed with parallel factor analysis (PARAFAC) using the N-way toolbox for MATLAB[5] with modification described elsewhere.[6,7] For the speciation analysis an adopted version of a MATLAB script from Smith[8] was used. Error estimations were performed by using a Monte Carlo approach.[3] More details on the specific analysis work-flow for the different experimental setups can be found below.

Affinity setup: Peptide (EF1-cyc, EF1-R-cyc) to 2 µM Eu^{3+}: Experimental parameters: linear increasing step size: 3+3·x µs; initial delay: 13 µs (to prevent $^5D_1 \rightarrow {}^7F_j$ transitions), gate width: 200 µs; slit width: 300 µm; accumulations: 200; kinetic series length: 21; gain: 4095. The respective peptide (50 µM peptide, containing 2 µM $EuCl_3$) was added stepwise over 20 additions (0-22.7 µM peptide) to a 1 mL solution containing 2 µM $EuCl_3$; the buffer, salt and Eu^{3+} concentration were held constant. Experiments were performed in duplicates.

Affinity setup: EF1-G-cyc to 5 µM Eu^{3+}: Experimental parameters: linear increasing step size: 3+3·x µs; initial delay: 12 µs (to prevent $^5D_1 \rightarrow {}^7F_j$ transitions), gate width: 100 µs; slit width: 200 µm; accumulations: 200; kinetic series length: 21; gain: 4095. The respective peptide (250 µM peptide, containing 5 µM $EuCl_3$) was added stepwise over 20 additions (0-64.4 µM peptide) to a 1 mL solution containing 5 µM $EuCl_3$; the buffer, salt and Eu^{3+} concentration was held constant during the titration experiment.

Ln^{3+} vs. Eu^{3+} Competition Experiment: Individual samples of the respective peptides (EF1-cyc, EF1-R-cyc, EF1-G-cyc) with the different $LnCl_3$ (La-Lu, except Pm) were mixed in 1.5 mL centrifuge tubes (40 µM Eu^{3+}, 40 µM Ln^{3+}, 10 µM peptide, 10 mM MOPSO, 100 mM KCl, pH 6.6), incubated at 6 °C overnight, transferred into cuvettes and measured with the following parameters: linear increasing step size: 3+3·x µs; initial delay: 12 µs (to prevent $^5D_1 \rightarrow {}^7F_j$ transitions), gate width: 100 µs; slit width: 200 µm; accumulations: 200; kinetic series length: 21; gain: 4095. For data analysis, the competition series was analysed in conjunction with a Eu^{3+} titration series and a pure Eu^{3+} aquo ion sample (Figure S1). The relative abundance of the two Eu^{3+} species (Figure S1B) within the Ln^{3+} series distribution (box in Figure S1A) is low, and the inclusion of additional samples increases the variance along this dimension in the PARAFAC model. For EF1-cyc and EF1-R-cyc, a mini-titration series (addition of peptide (0-10 µM) over 10 steps to a 1 mL solution containing 40 µM $EuCl_3$, buffer, metal and salt concentration kept constant) was performed, while for EF1-G-cyc the Ln^{3+}-competition series was extended by two samples equivalent to sample 24 and 25 in FigureS1A.

To estimate the affinities of the remaining Ln^{3+} ions, the previously determined Eu^{3+} affinity was used as a reference. Using the mean value and standard deviation, 100 synthetic affinity values were randomly drawn from the corresponding distribution and fixed during the fitting procedure for the other Ln^{3+} ions. The fits were performed pairwise using the respective Ln^{3+} sample together with the Eu^{3+} sample.

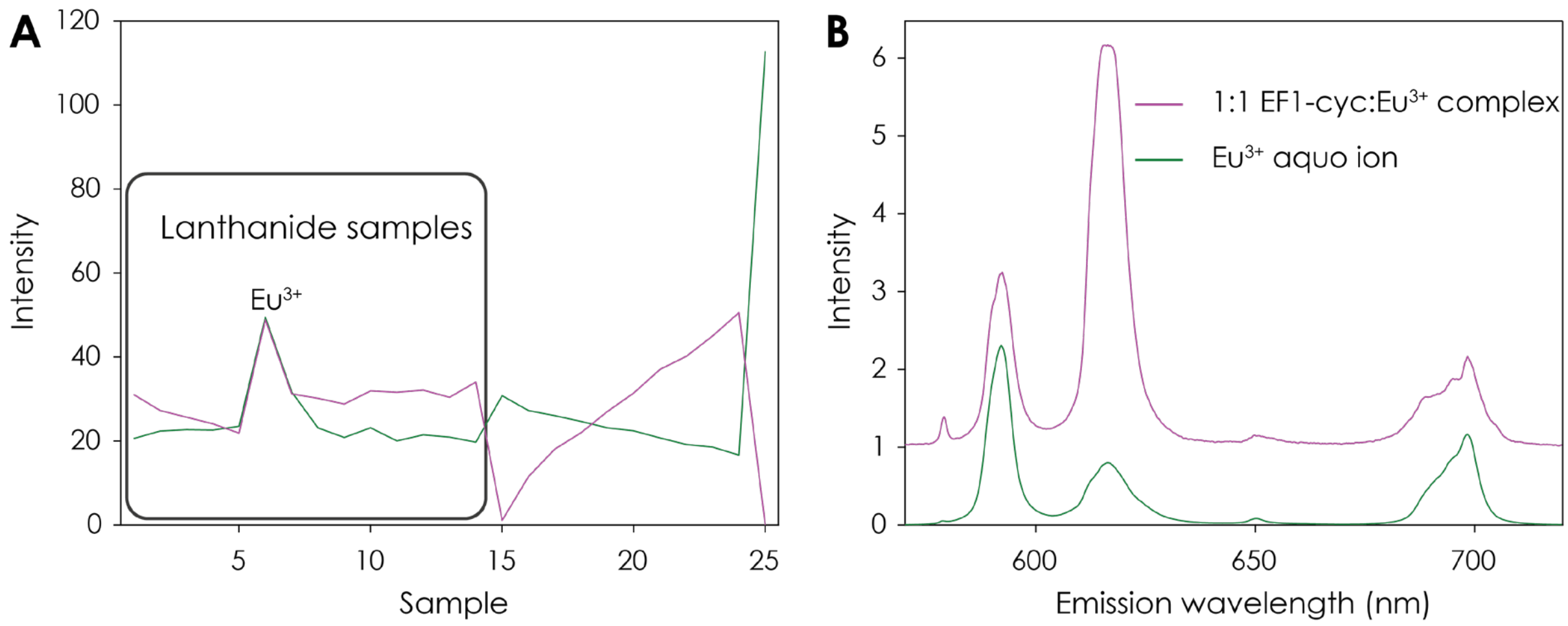

Figure S1 PARAFAC analysis of EF1-cyc setup for the determination of the affinities of the whole (except Pm^{3+}) Ln^{3+} series. **(A)** The Ln^{3+} samples (box) were extended by a short titration series and a sample of the pure Eu^{3+} aquo ion to increase the variance of the two Eu^{3+} species. **(B)** Emission spectra of the two Eu^{3+} species (green – Eu^{3+} aquo ion; magenta – Eu^{3+}-EF1-cyc-complex) in this setup.

Affinity setup: Peptide (EF1-cyc, EF1-R-cyc) to 100 nM Cm^{3+}: Experimental parameters: linear increasing step size: 1+2·x µs; initial delay: 1 µs, gate width: 200 µs; slit width: 300 µm; accumulations: 300; kinetic series length: 15; gain: 4095. The respective peptide (20 µM peptide) was added stepwise over 20 additions (0-4436.5 nM peptide) to a 1.5 mL solution containing 100 nM $CmCl_3$; the buffer and salt concentration were held constant during the titration experiment. To facilitate the handling with the radioactive sample, the titrant solution did not contain any Cm^{3+}. Therefore, there was an unavoidable slight dilution of Cm^{3+} during the titration experiment which was considered during the data analysis.

2.4 Circular dichroism (CD) Spectroscopy

CD spectra were recorded in MOPSO buffer (10 mM, 100 mM KCl, pH 6.6) at 25 °C between 190 nm and 250 nm in a *Hellma Analytics*® quartz cell (110-QS, 1 mm pathlength) using a *JASCO* 1500 CD spectropolarimeter as described previously.[1] Recorded spectra were corrected by subtracting a spectrum of the used buffer using the *Spectragryph* software[9] and the measured ellipticity in mdeg was converted to molar (θ) as described previously.[1] Data was plotted with MATLAB.

2.5 Nuclear Magnetic Resonance (NMR) Spectroscopy

NMR spectra were obtained on an *Agilent* DD2-600 14.1 T system operating at ^{1}H and ^{13}C resonance frequencies of 599.8 and 150.8 MHz, respectively, using a 5 mm oneNMR™ probe. Unless stated otherwise, the measurements were performed at (25 ± 1) °C in 30 mM MES-d_{13} buffer (*Cambridge Isotope Laboratories*) with 100 mM KCl in a 9/1 (v/v) mixture of H_2O/D_2O (99.95% D; containing 0.03% TMSP-d_4, *Deutero*) at pH = 6.6 (pH carefully adjusted using NaOD (99% D, *Deutero*)). All $LnCl_3$ and peptide stock solutions were directly prepared in the described buffer solution. Chemical shifts are reported in parts per million (ppm), relative to the $Si(Me)_3$ signal (δ_H = 0.00 ppm) of internal TMSP-d_4. ^{1}H NMR Ln-titration experiments were conducted upon stepwise addition of the respective $LnCl_3$ over 14 additions (0.0 – 2.0 mM) to a solution containing 200 µM of the respective peptide. Concentrations of the buffer mixture and the peptide were kept constant.

^{1}H and ^{13}C chemical shifts of the peptides were determined by ^{1}H NMR and 2D NMR experiments (^{1}H,^{1}H-TOCSY, ^{1}H,^{13}C-HMBC, ^{1}H,^{13}C-HSQC), using concentrations at least twice as high as for the titration series. ^{1}H NMR spectra were acquired using a pre-saturation sequence (2 s or 1 s in ^{1}H or 2D experiments, respectively) for solvent suppression techniques. ^{1}H spectra were obtained after applying a 2.9 µs (30°) excitation pulse, followed by 2 s acquisition time and 2 s relaxation delay, accumulating a varying number of scans (64 – 256) depending on sample conditions. Heteronuclear single-quantum coherence (^{1}H,^{13}C-HSQC) and Heteronuclear multiple-bond correlation (^{1}H,^{13}C-HMBC) sequences took advantage of adiabatic pulses and gradient-selection, acquiring 2k × 512 and 2k × 1k complex points in the direct (f_2) and indirect (f_1) dimensions, respectively. 64 – 96 (HSQC) and 160 – 192 (HMBC) individual spectra were accumulated per f_1-increment with a relaxation delay of 1 s. Polarization transfer delays of 62.5 and 3.85 ms were applied, respectively, corresponding to ^{1}J = 130 Hz in HSQC and ^{n}J = 8 Hz in HMBC. Zero-quantum-filtered total correlation spectroscopy (zTOCSY) experiments were acquired with 80 ms mixing time, 2k × 256 complex points accumulating 64 individual spectra per f_1 increment, and a relaxation delay of 1 s. All NMR spectra were processed using MestReNova (12.0.2).

Note to the interpretation of the metal titrations: With reference to our former study,[10] we here consider displacements of at least one of the carboxymethylene hydrogens to lower field by at least +100 ppb (parts per billion) as indicative of metal binding. Complexation-induced conformational changes can result in an increase in the methylene hydrogens' distinctiveness (diastereotopicity), causing the other signal to shift upfield. In case of both carboxymethylene signals being displaced upfield, the respective residue is considered non-binding, and only affected by electron density redistribution from alterations of the local environment due to, *e.g.*, steric interaction with other AA side chains.

2.6 Lanthanide-binding studies in the gas phase *via* mass spectrometry

All samples for mass spectrometry measurements were prepared in LCMS grade water with the following final concentrations: 10 µM of the respective peptide and 50 µM $EuCl_3$.

Collision-induced dissociation (CID) experiments were performed on a *Thermo Scientific* LTQ XL Orbitrap mass spectrometer. The respective precursor ions were isolated in the linear ion trap of the instrument with an isolation width of typically 5 mass units, then resonantly excited with an energy of 30 NCE for monocations (= normalised collision energy, instrument specific) and 18 NCE for dications. The ions collide with residual helium gas (typically $4 \cdot 10^{-3}$ mbar) within an excitation period of 30 ms. Under these conditions significant fragmentation is observed, but the precursor ions are still detectable.

Ion mobility measurements (IMS) were performed in a *Waters* cyclic series mass spectrometer, that comprises the combination of a cyclic ion mobility cell (filled with typically 1.7 mbar of nitrogen) and a high-resolution time-of-flight mass spectrometer. The arrival time distributions are calibrated against the set of "Agilent tunemix" ions using the CCS values of Stow *et al.*[11] Details of our calibration procedure have been described previously.[12]

3 Molecular dynamics (MD) simulations

MD simulations and data analyses were performed using the AMBER 22 program package[13] with ff14SB force field applied on the peptide. For Ln^{3+} (Ln = La, Eu, Lu), 12-6-4 Lennard-Jones-type parameters developed by Merz and co-workers[14–16] as well as those modified by Hay and co-workers[17] were employed. For the second set of parameters, which were designed to have more "realistic" metal-protein interactions, since there are only parameters available for La^{3+} ion, the 12-6-4 LJ pairwise oxygen C_{ij} coefficients for Eu^{3+} and Lu^{3+} were set to 16.2 and 15.6 kcal mol^{-1}Å^{-4}, respectively. The structures of short cyclic peptides as studied here are generally less flexible compared to non-cyclic ones. Therefore, the initial structures of peptides were constructed as pure ring without introducing any secondary structure element and including Ln^{3+} ion, and were equilibrated for 500 ns.

Within the simulations the protonation state of the peptides was adjusted to physiological pH and TIP3P waters were added with a minimum water layer thickness of 10 Å. Initially, the steepest descent method was executed for 500 steps, followed by 500 steps of conjugate gradient with a harmonic restraint of 500 kcal mol^{-1} Å^{-1} applied to the peptides. Subsequently, 1000 steps of steepest descent and 1500 steps of conjugate gradient were performed without constraints. The system was heated to 300 K from 0 K for 40 ps with harmonic restraint of 10 kcal mol^{-1} Å^{-1} applied to the peptides. This was followed by an additional 1 ns preconditioning run at 300 K with no restraint on the solutes. Finally, a 500 ns MD simulation run was conducted under periodic boundary conditions in an NPT ensemble. The simulations were terminated and restarted at intervals of 5 ns. The SHAKE algorithm, a 2 fs time integration step, 12 Å as the cut-off for non-bonded interactions, and the particle mesh Ewald (PME) method were utilised. The MD trajectory was recorded every 50 ps. For the data analysis, only the last 100 ns trajectory was taken. In the context of oxygen atom coordination numbers, oxygen atoms within a 2.7 Å distance from metal centres were designated as coordinated atoms.

4 Supplementary Tables and Figures

4.1 Supplementary ITC Data

Table S3 Supplementary values to Table 1 of the main manuscript; n (used as concentration correction factor of the cell concentration during the analysis), Δ*H* values of the global analysis of three independent Eu^{3+} to peptide ITC titration experiments with different peptide concentrations in the cell. Δ*G* and *T*Δ*S* were calculated using the Δ*H* and the obtained K_D value; $T = 298.15$ K; the given uncertainties are the result of the used Monte Carlo approach.

	n	Δ*H* (kJ/mol)	Δ*G* (kJ/mol)	*T*Δ*S* (kJ/mol)
**EF1-cyc**	1.15 ± 0.004	32.6 ± 0.2	−31.4 ± 0.1	64.0 ± 0.1
**EF1-R-cyc**	1.39 ± 0.007	27.8 ± 0.2	−29.0 ± 0.1	56.9 ± 0.1
**EF1-G-cyc**	0.98 ± 0.004	30.5 ± 0.1	−32.1 ± 0.1	62.6 ± 0.1

Table S4 ITC data of $LnCl_3$ to EF1-cyc titration experiments with a peptide concentration of 30 µM in the cell and metal concentration of 900 µM in the syringe. Experiments were performed in triplicates and analysed globally; n (used as concentration correction factor of the cell concentration during the analysis). Δ*G* and *T*Δ*S* were calculated using the Δ*H* and the obtained K_D value; $T = 298.15$ K; the given uncertainties are the result of the used Monte Carlo approach.

Ln^{3+}	n	K_D (µM)	Δ*H* (kJ/mol)	Δ*G* (kJ/mol)	*T*Δ*S* (kJ/mol)
**La**	1.14 ± 0.009	7.3 ± 0.2	24.5 ± 0.2	−29.3 ± 0.07	53.8 ± 0.2
**Ce**	1.11 ± 0.003	5.2 ± 0.1	29.6 ± 0.1	−30.2 ± 0.04	59.8 ± 0.1
**Pr**	1.18 ± 0.005	4.0 ± 0.1	27.8 ± 0.2	−30.8 ± 0.05	58.6 ± 0.1
**Nd**	1.18 ± 0.006	3.9 ± 0.1	28.1 ± 0.2	−30.9 ± 0.07	59.0 ± 0.2
**Sm**	1.08 ± 0.003	3.0 ± 0.1	32.5 ± 0.1	−31.5 ± 0.05	64.0 ± 0.1
**Eu**	1.11 ± 0.006	3.3 ± 0.1	34.6 ± 0.3	−31.3 ± 0.07	65.9 ± 0.2
**Gd**	1.17 ± 0.005	4.9 ± 0.1	36.0 ± 0.2	−30.3 ± 0.05	66.3 ± 0.2
**Tb**	1.21 ± 0.006	5.6 ± 0.1	40.5 ± 0.3	−30.0 ± 0.06	70.5 ± 0.2
**Dy**	1.09 ± 0.008	5.7 ± 0.2	43.7 ± 0.5	−29.9 ± 0.08	73.7 ± 0.4
**Ho**	1.15 ± 0.010	7.6 ± 0.2	42.7 ± 0.5	−29.2 ± 0.08	72.0 ± 0.4
**Er**	1.17 ± 0.010	8.4 ± 0.3	44.9 ± 0.5	−29.0 ± 0.08	73.9 ± 0.5
**Tm**	1.26 ± 0.010	9.1 ± 0.2	43.4 ± 0.4	−28.8 ± 0.06	72.1 ± 0.4
**Yb**	1.04 ± 0.004	7.5 ± 0.1	51.1 ± 0.3	−29.3 ± 0.04	80.4 ± 0.3
**Lu**	1.14 ± 0.005	10.1 ± 0.1	49.2 ± 0.3	−28.5 ± 0.03	77.7 ± 0.2

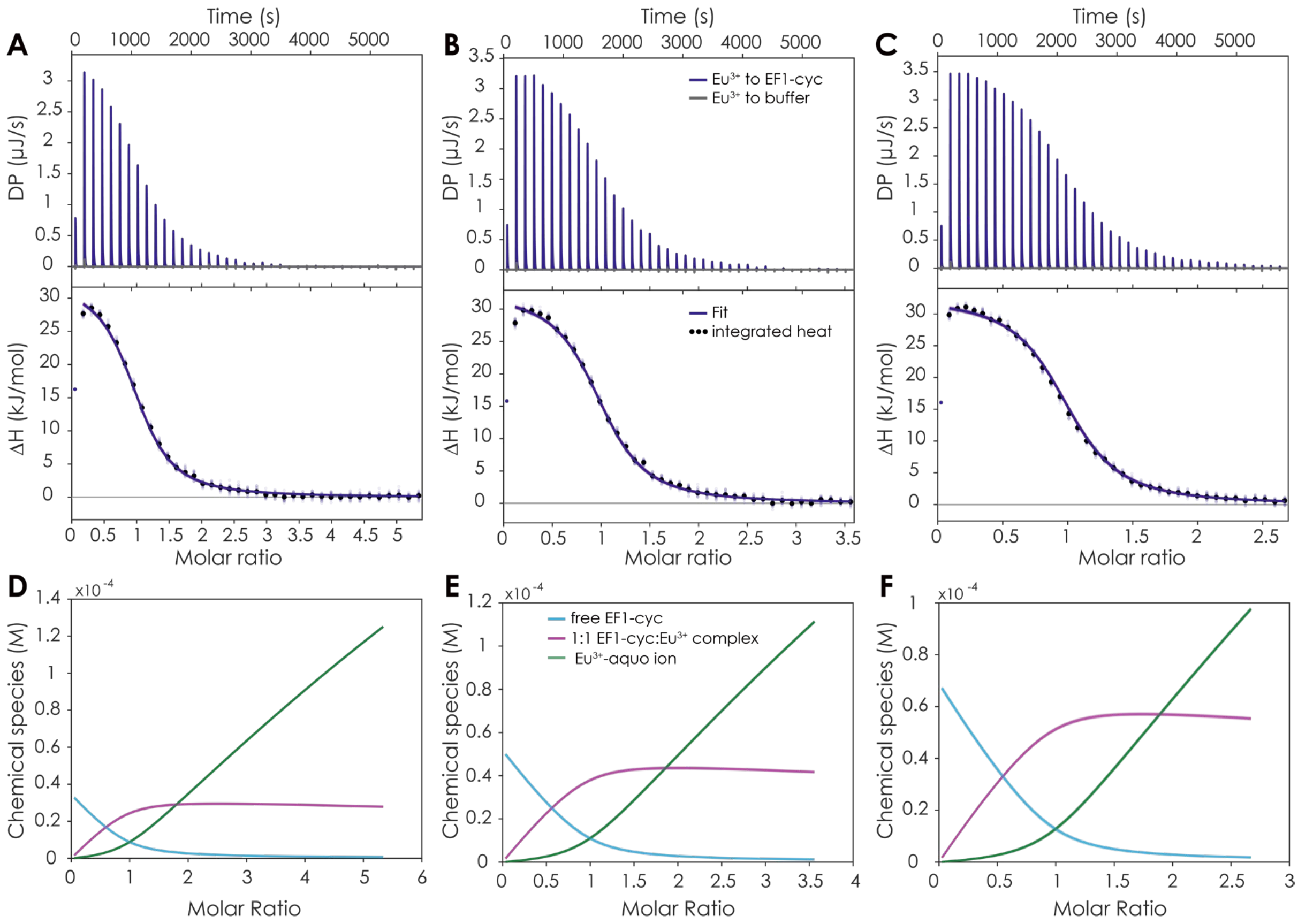

Figure S2: ITC data of EF1-cyc binding to Eu^{3+}. Thermogram, integrated heat, and used fit with a model for a 1:1 binding interaction obtained by titrating a 900 µM $EuCl_3$ solution to **(A)** 30 µM peptide **(B)** 45 µM peptide and **(C)** 60 µM peptide. For the three independently obtained data sets a global fit was used; the first titration point was excluded from the analysis in all cases. Chemical species distribution observed in the ITC titration experiments **(D)** 900 µM $EuCl_3$ to 30 µM peptide, **(E)** 900 µM $EuCl_3$ to 45 µM peptide and **(F)** 900 µM $EuCl_3$ to 60 µM peptide. Experimental conditions: pH 6.6, 25 °C, 10 mM MOPSO buffer, 100 mM KCl. Overlayed lines/ symbols represent 100 MC runs which were used for error estimation (see Section 2.2).

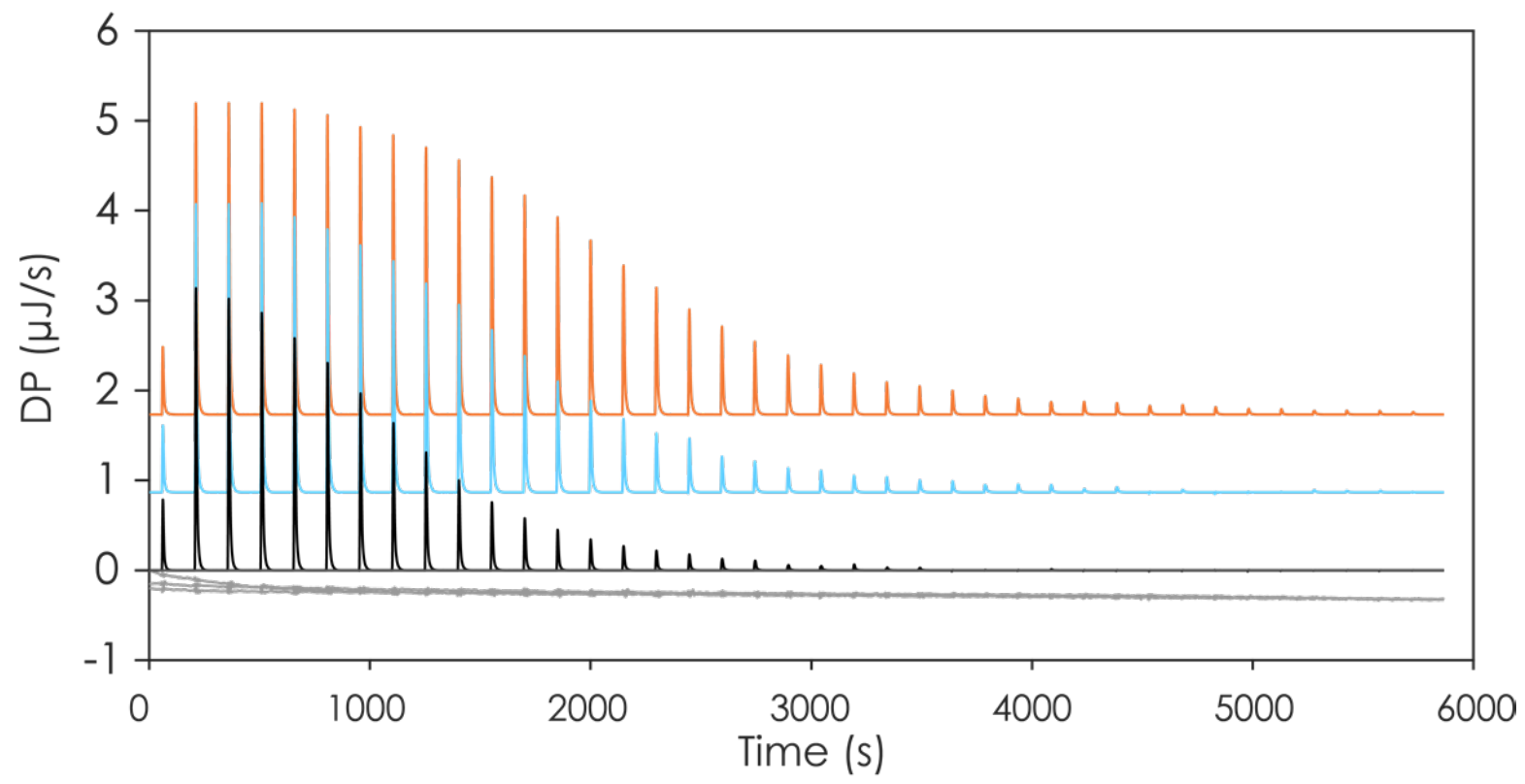

Figure S3 SVD-corrected thermograms of the $EuCl_3$ to EF1-cyc titrations (orange: 60 µM set, blue: 45 µM set, black: 30 µM set) shown in Figure S2; the residuals for all three titrations are shown in grey. For better visualisation, the data was stacked with an offset to the *x*-axis.

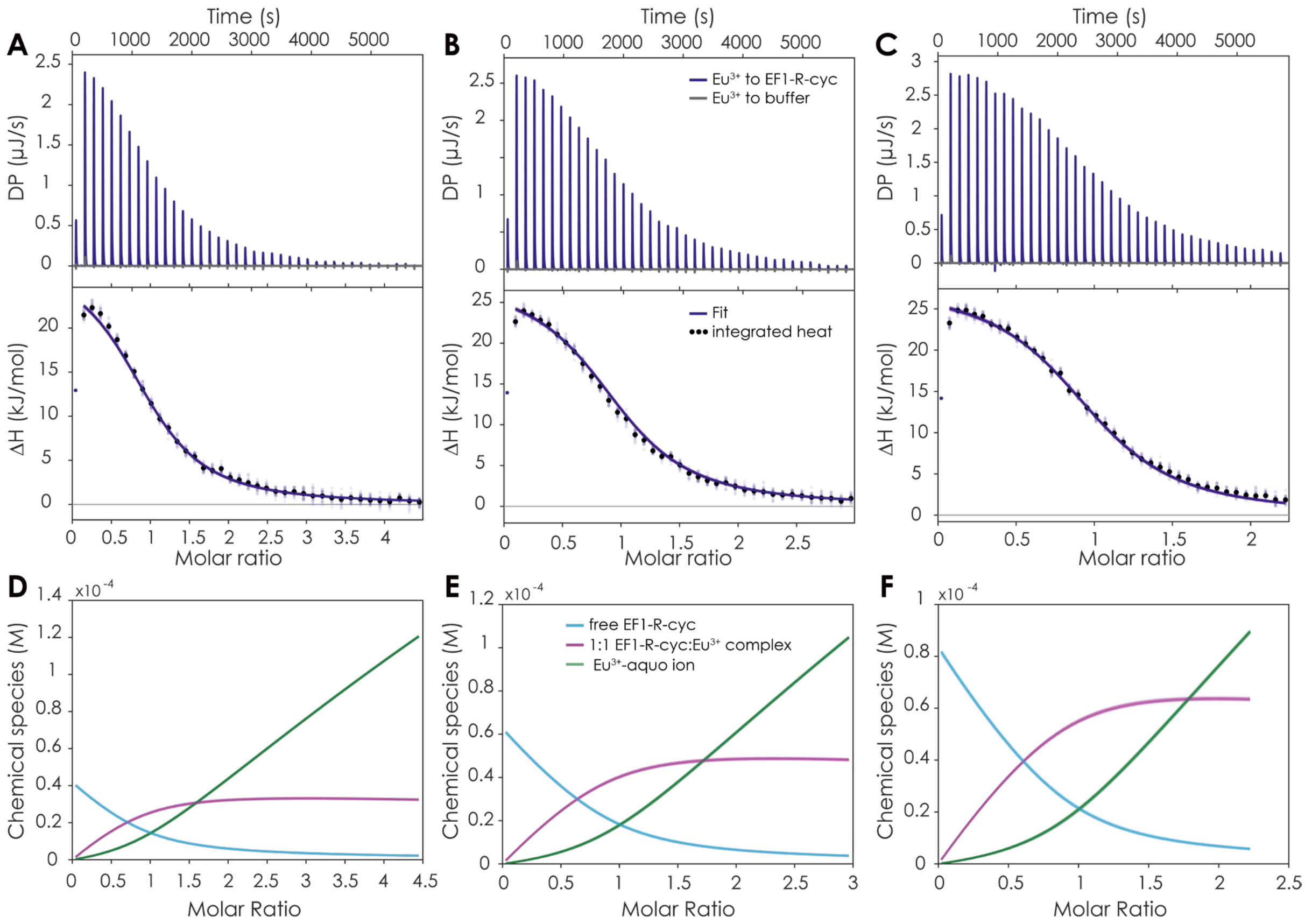

Figure S4 ITC data of EF1-R-cyc binding to Eu^{3+}. Thermogram, integrated heat, and used fit with a model for a 1:1 binding interaction obtained by titrating a 900 µM $EuCl_3$ solution to **(A)** 30 µM peptide **(B)** 45 µM peptide and **(C)** 60 µM peptide. For the three independently obtained data sets a global fit was used; the first titration point was excluded from the analysis in all cases. Chemical species distribution observed in the ITC titration experiments **(D)** 900 µM $EuCl_3$ to 30 µM peptide, **(E)** 900 µM $EuCl_3$ to 45 µM peptide and **(F)** 900 µM $EuCl_3$ to 60 µM peptide. Experimental conditions: pH 6.6, 25 °C, 10 mM MOPSO buffer, 100 mM KCl. Overlayed lines/ symbols represent 100 MC runs which were used for error estimation (see Section 2.2).

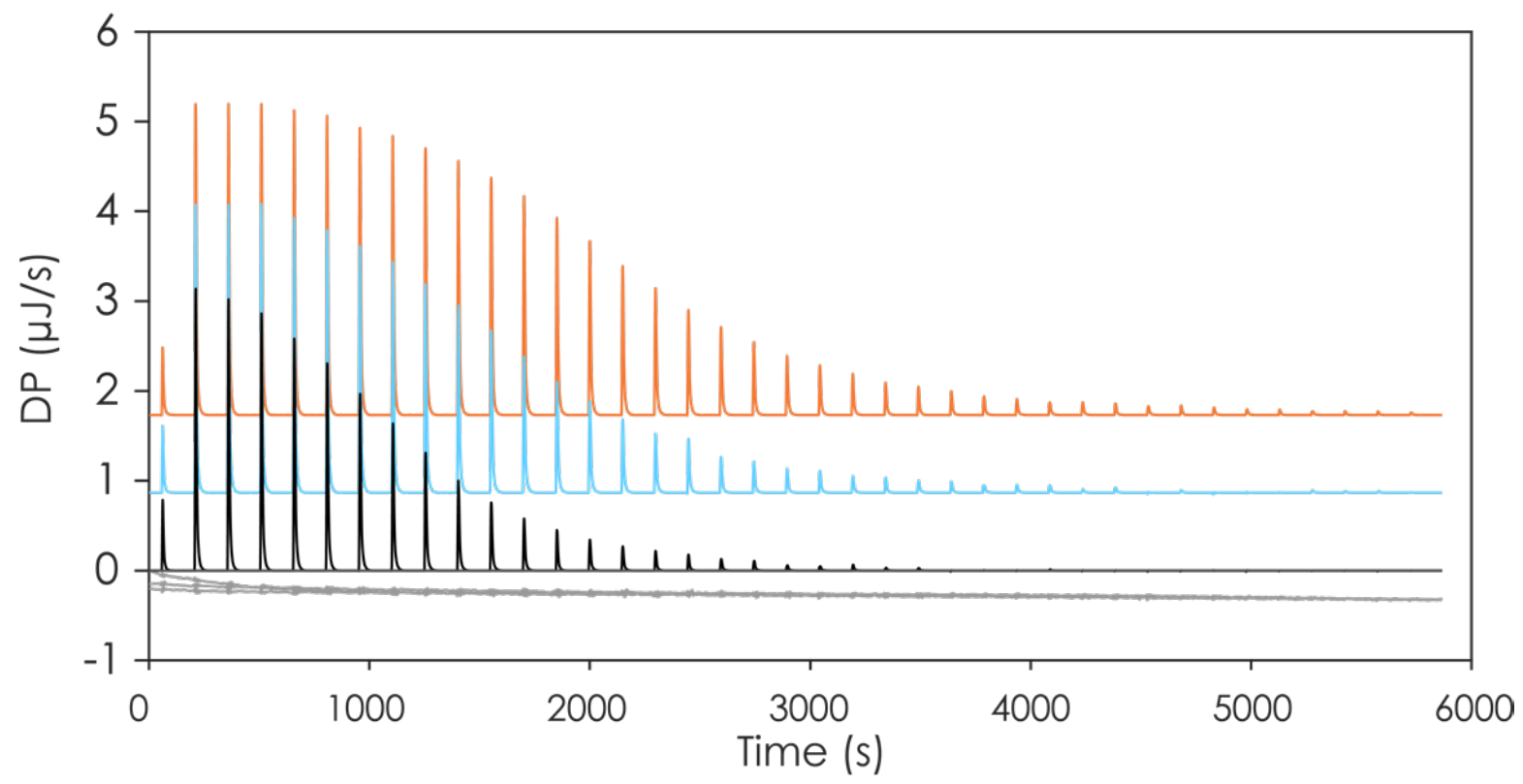

Figure S5 SVD-corrected thermograms of the $EuCl_3$ to EF1-R-cyc titrations (orange: 60 µM set, blue: 45 µM set, black: 30 µM set) shown in Figure S4; the residuals for all three titrations are shown in grey. For better visualisation, the data was stacked with an offset to the *x*-axis.

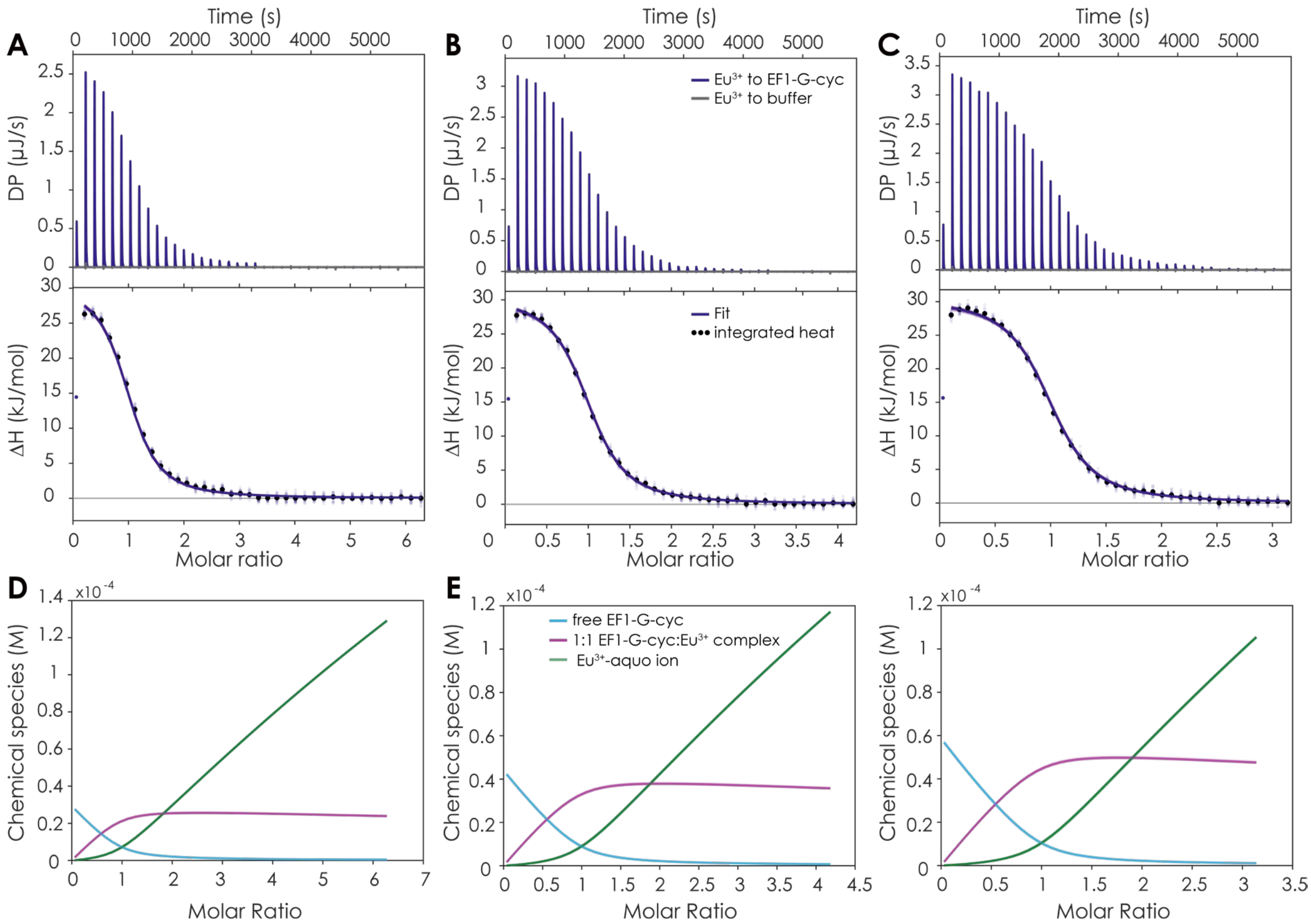

Figure S6 ITC data of EF1-G-cyc binding to Eu^{3+}. Thermogram, integrated heat, and used fit with a model for a 1:1 binding interaction obtained by titrating a 900 µM $EuCl_3$ solution to **(A)** 30 µM peptide **(B)** 45 µM peptide and **(C)** 60 µM peptide. For the three independently obtained data sets a global fit was used; the first titration point was excluded from the analysis in all cases. Chemical species distribution observed in the ITC titration experiments **(D)** 900 µM $EuCl_3$ to 30 µM peptide, **(E)** 900 µM $EuCl_3$ to 45 µM peptide and **(F)** 900 µM $EuCl_3$ to 60 µM peptide. Experimental conditions: pH 6.6, 25 °C, 10 mM MOPSO buffer, 100 mM KCl. Overlayed lines/ symbols represent 100 MC runs which were used for error estimation (see Section 2.2).

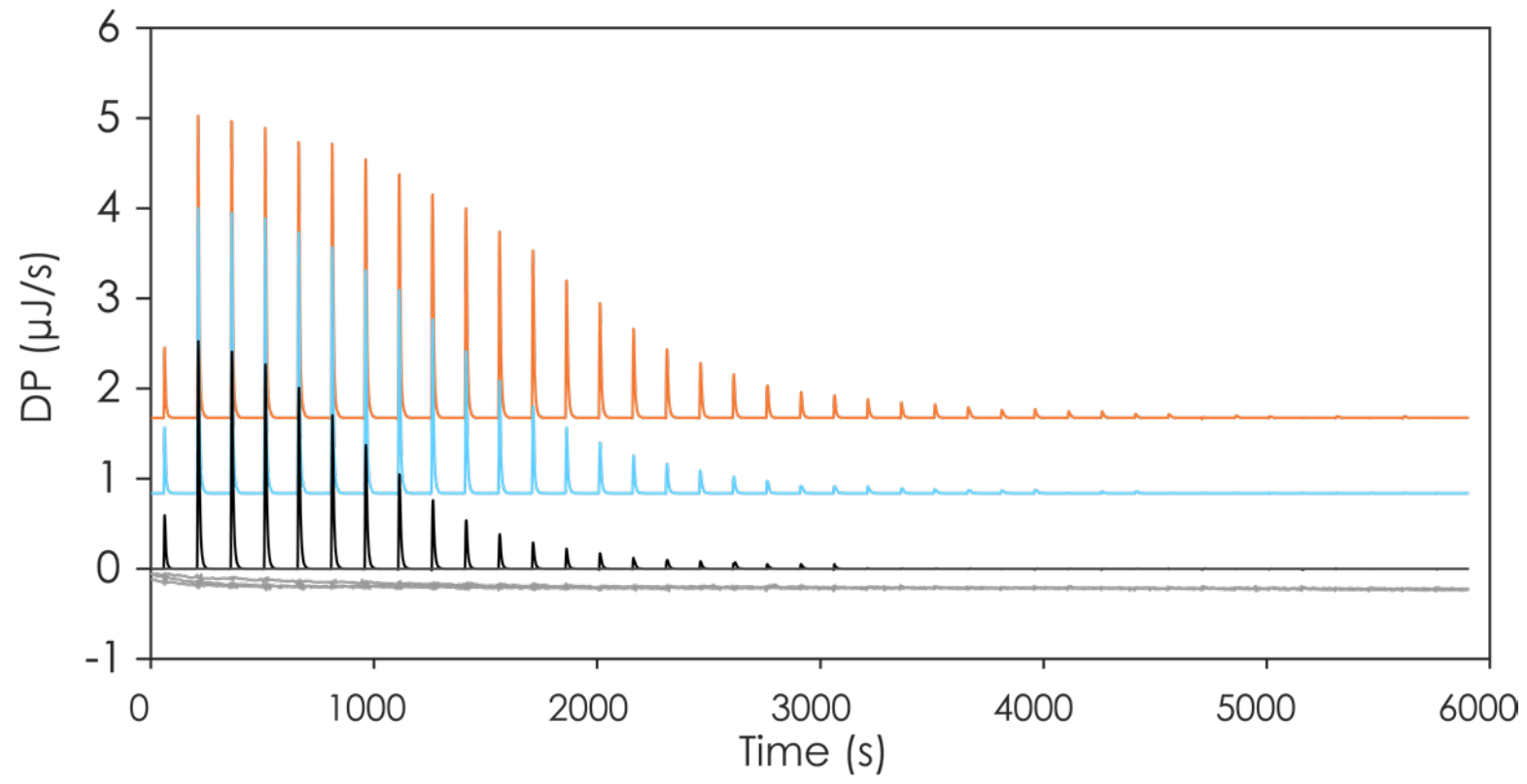

Figure S7 SVD-corrected thermograms of the $EuCl_3$ to EF1-R-cyc titrations (orange: 60 µM set, blue: 45 µM set, black: 30 µM set) shown in Figure S6; the residuals for all three titrations are shown in grey. For better visualisation, the data was stacked with an offset to the *x*-axis.

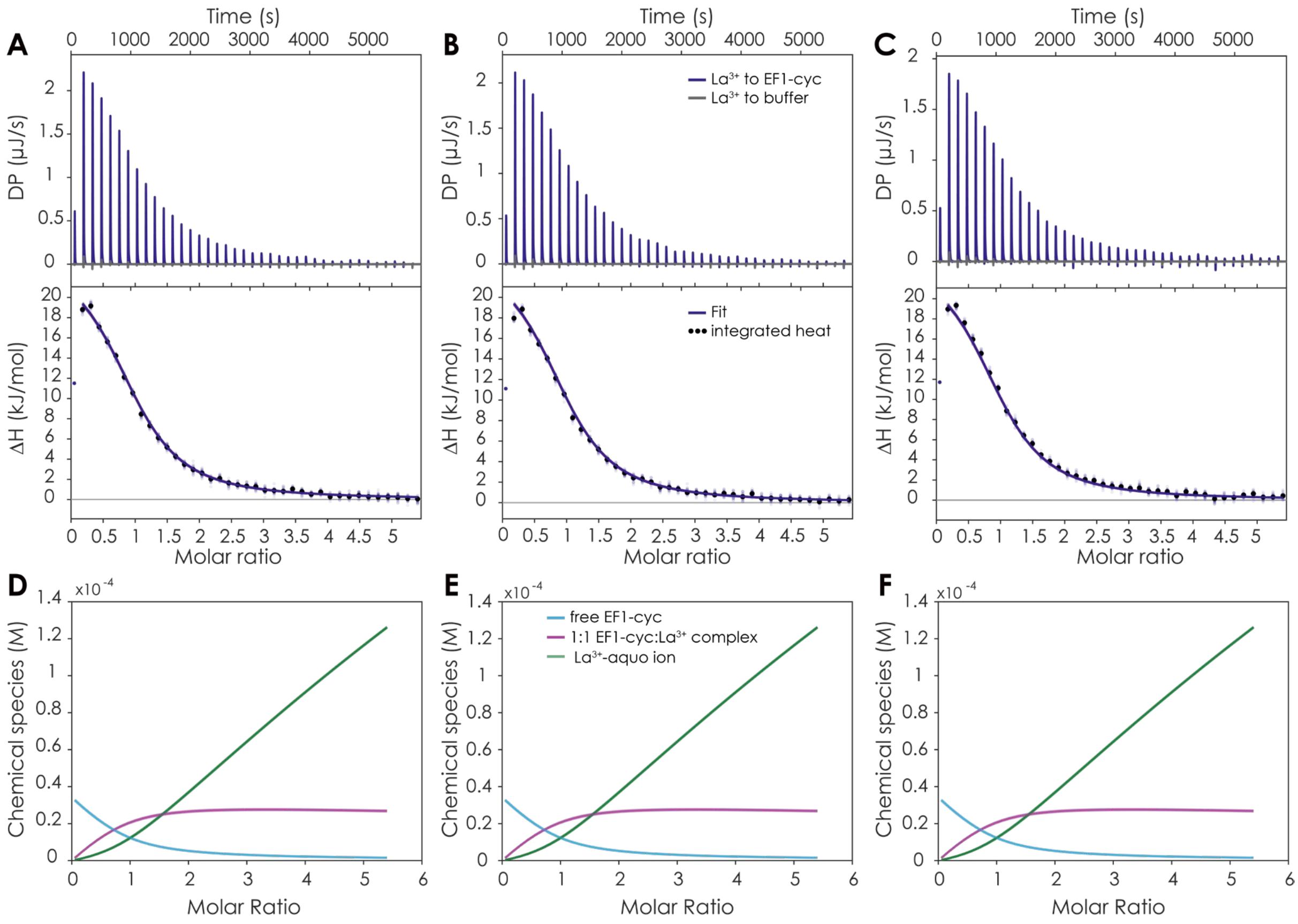

Figure S8 ITC data of EF1-cyc binding to La^{3+}. Thermogram, integrated heat, and used fit with a model for a 1:1 binding interaction obtained by titrating a 900 µM $LaCl_3$ solution to 30 µM peptide in triplicates **(A)** to **(C)**. The data was analysed globally; the first titration point was excluded from the analysis in all cases. The corresponding chemical species distribution for dataset **(A)** is shown in **(D)**, for **(B)** in **(E)** and for **(C)** in **(F)**. Experimental conditions: pH 6.6, 25 °C, 10 mM MOPSO buffer, 100 mM KCl. Overlayed lines/ symbols represent 100 MC runs which were used for error estimation (see Section 2.2).

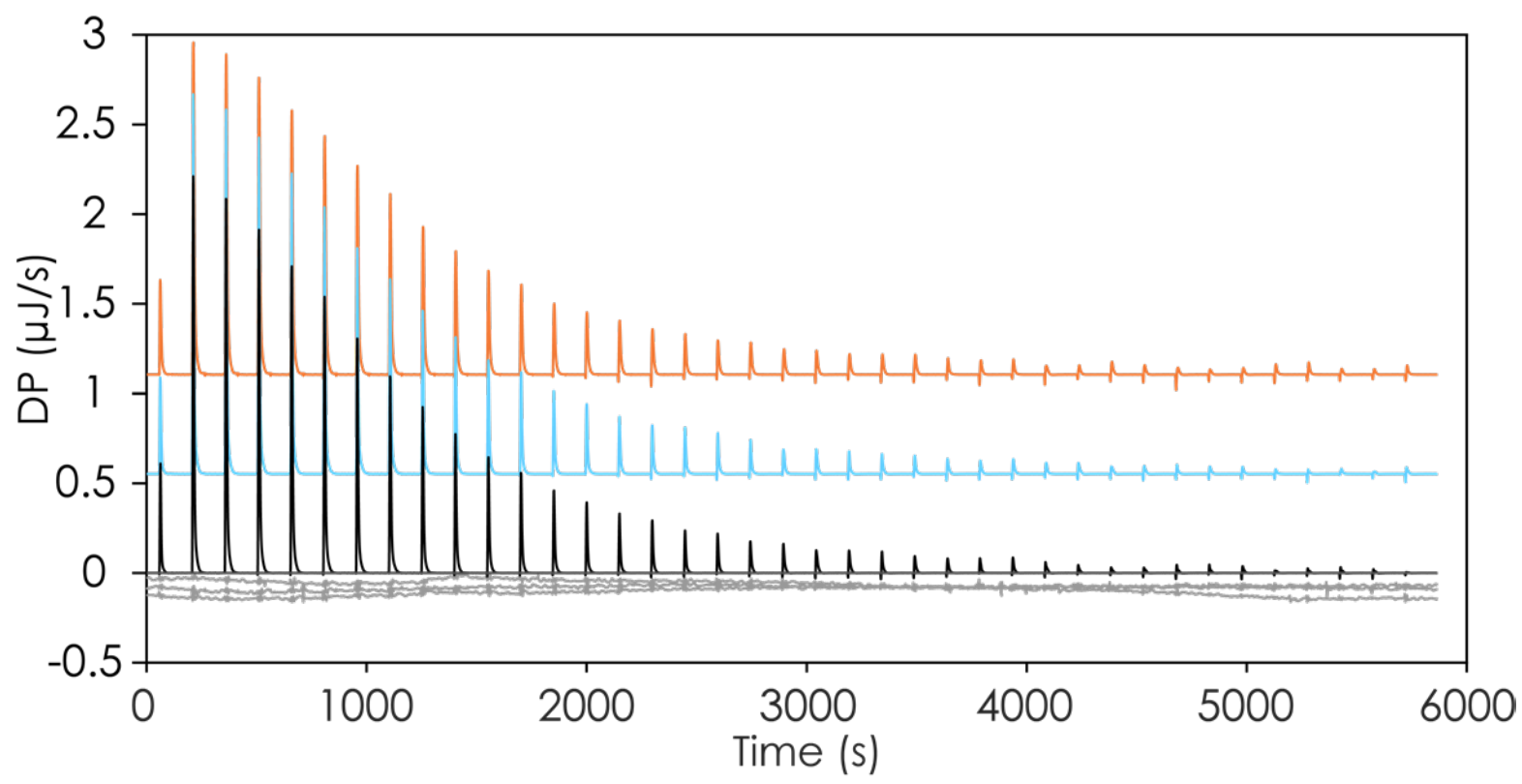

Figure S9 SVD-corrected thermograms of the 900 µM $LaCl_3$ to 30 µM EF1-cyc titrations performed in triplicates (black: corresponding to Figure S8A, blue: corresponding to Figure S8B, orange: corresponding to Figure S8C) shown in Figure S8; the residuals for all three titrations are shown in grey. For better visualisation, the data was stacked with an offset to the *x*-axis.

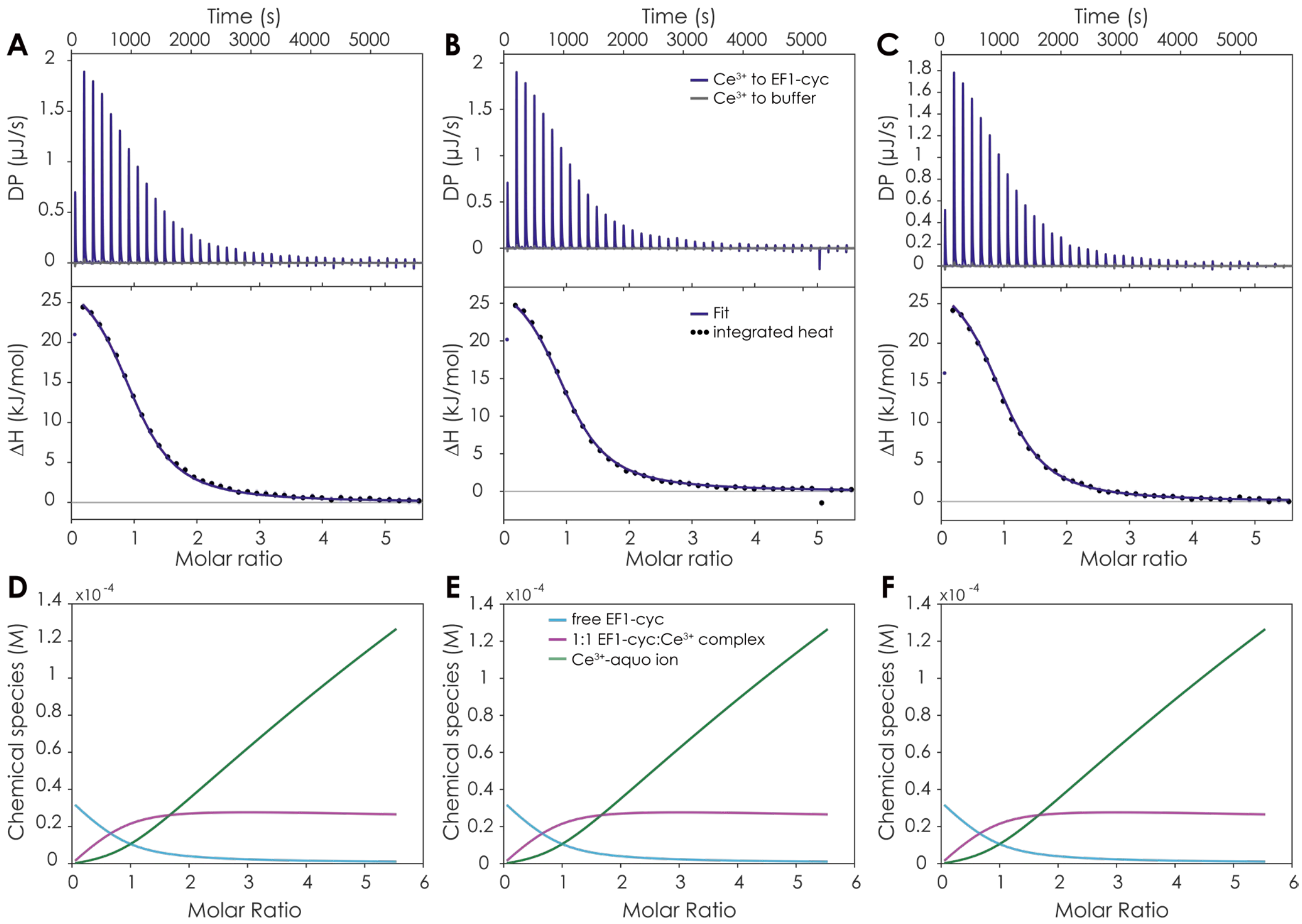

Figure S10 ITC data of EF1-cyc binding to Ce^{3+}. Thermogram, integrated heat, and used fit with a model for a 1:1 binding interaction obtained by titrating a 900 µM $CeCl_3$ solution to 30 µM peptide in triplicates **(A)** to **(C)**. The data was analysed globally; the first titration point was excluded from the analysis in all cases. The corresponding chemical species distribution for dataset **(A)** is shown in **(D)**, for **(B)** in **(E)** and for **(C)** in **(F)**. Experimental conditions: pH 6.6, 25 °C, 10 mM MOPSO buffer, 100 mM KCl. Overlayed lines/ symbols represent 100 MC runs which were used for error estimation (see Section 2.2).

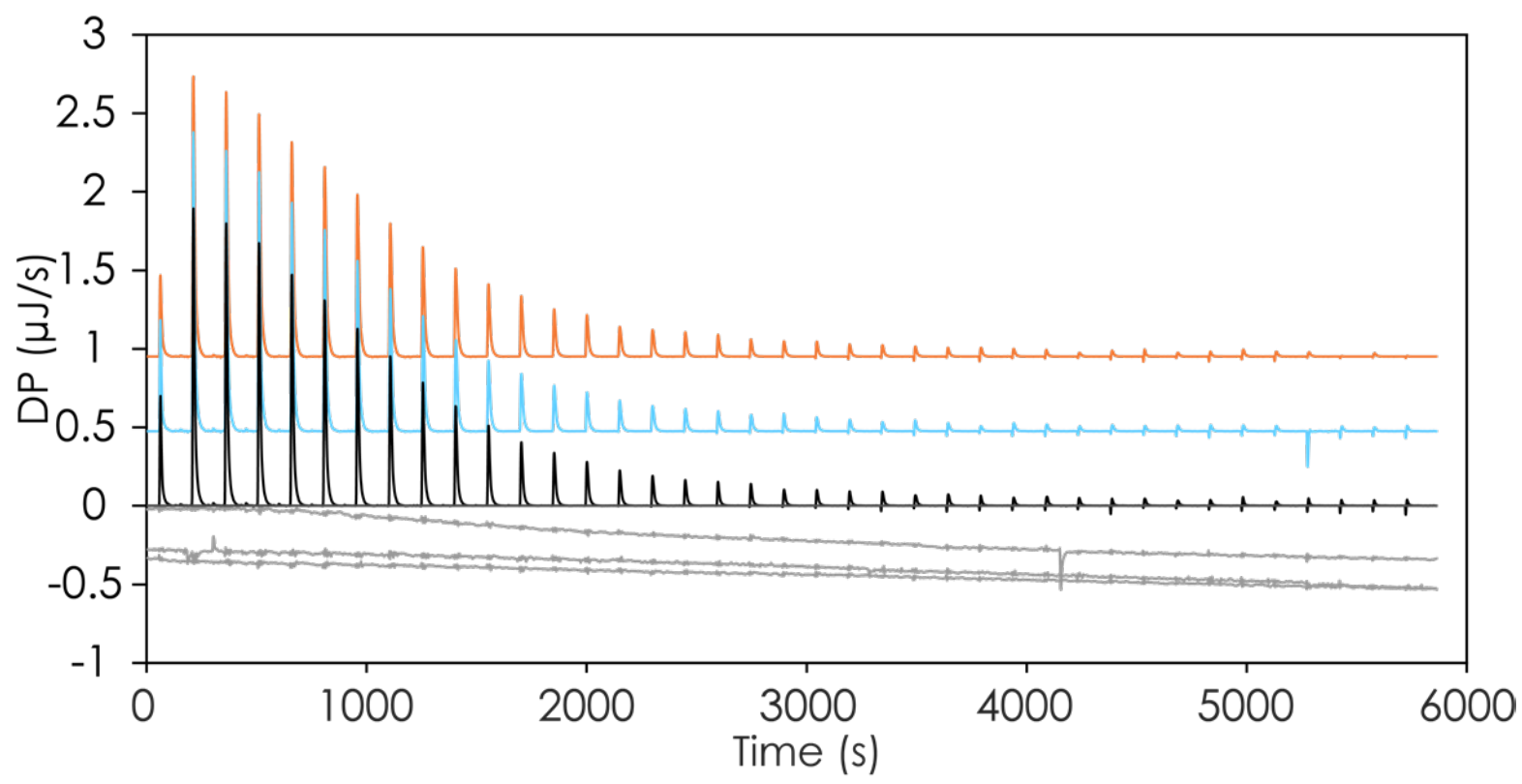

Figure S11 SVD-corrected thermograms of the 900 µM $CeCl_3$ to 30 µM EF1-cyc titrations performed in triplicates (black: corresponding to Figure S10A, blue: corresponding to Figure S10B, orange: corresponding to Figure S10C) shown in Figure S10; the residuals for all three titrations are shown in grey. For better visualisation, the data was stacked with an offset to the *x*-axis.

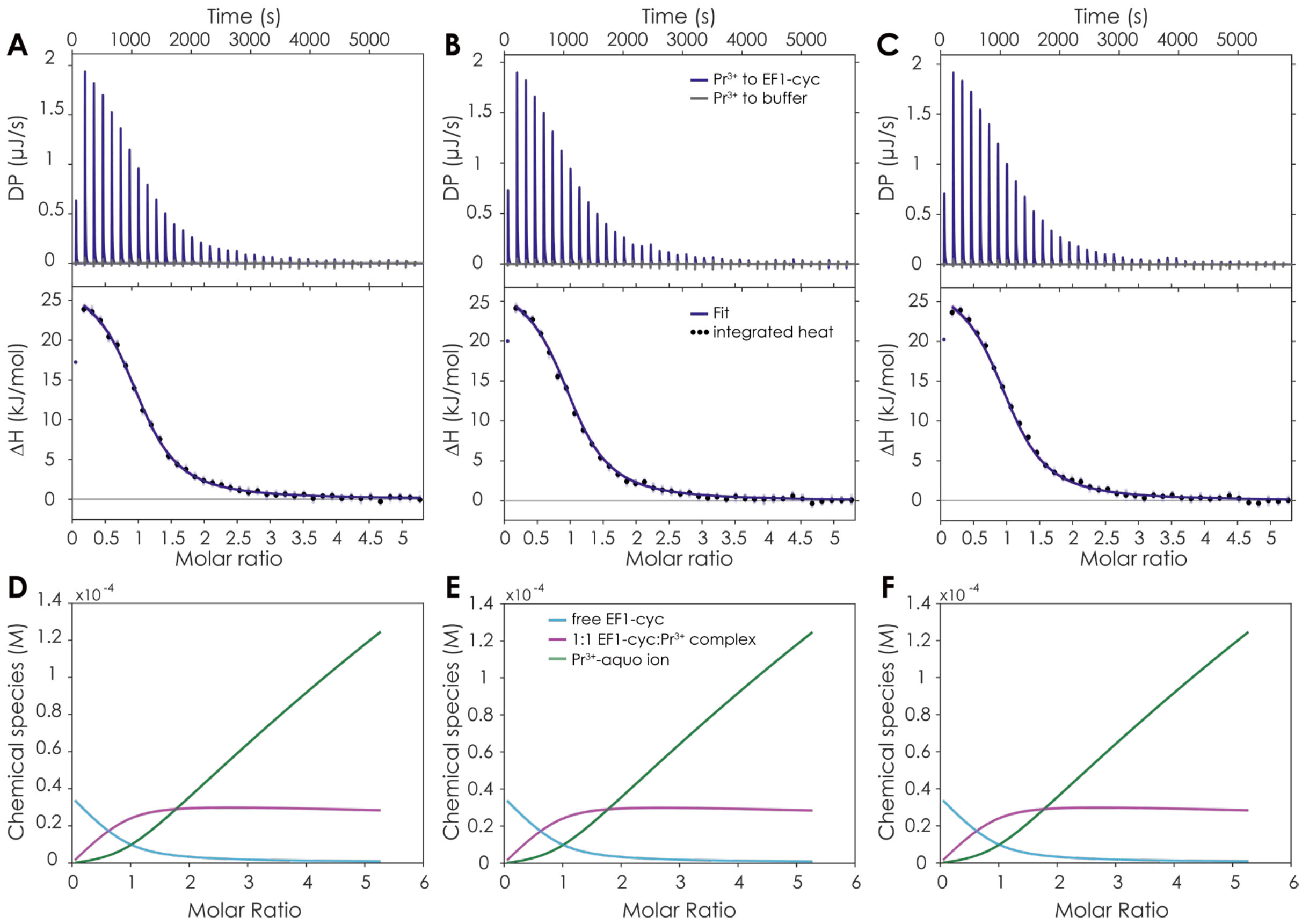

Figure S12 ITC data of EF1-cyc binding to Pr^{3+}. Thermogram, integrated heat, and used fit with a model for a 1:1 binding interaction obtained by titrating a 900 µM $PrCl_3$ solution to 30 µM peptide in triplicates **(A)** to **(C)**. The data was analysed globally; the first titration point was excluded from the analysis in all cases. The corresponding chemical species distribution for dataset **(A)** is shown in **(D)**, for **(B)** in **(E)** and for **(C)** in **(F)**. Experimental conditions: pH 6.6, 25 °C, 10 mM MOPSO buffer, 100 mM KCl. Overlayed lines/ symbols represent 100 MC runs which were used for error estimation (see Section 2.2).

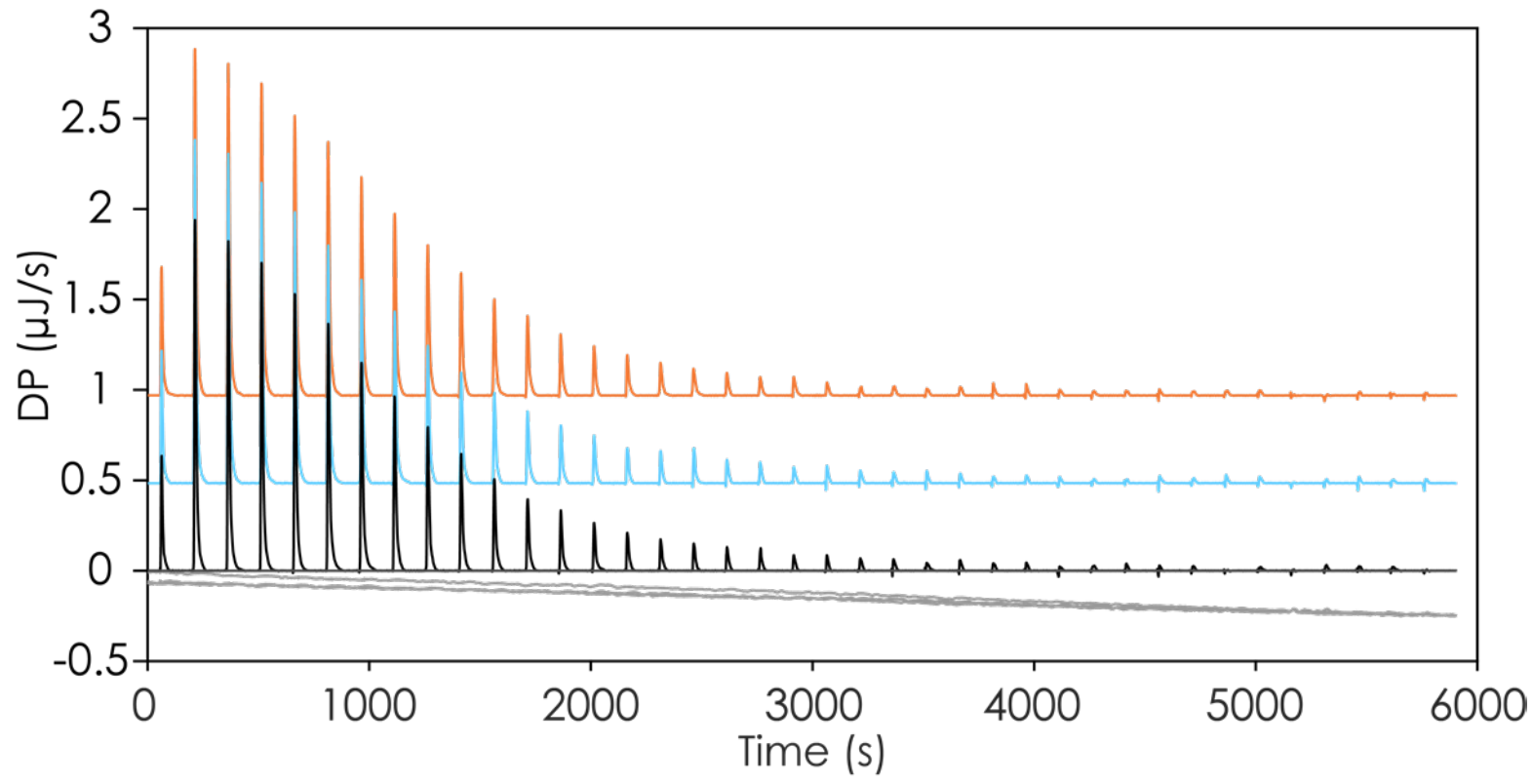

Figure S13 SVD-corrected thermograms of the 900 µM $PrCl_3$ to 30 µM EF1-cyc titrations performed in triplicates (black: corresponding to Figure S12A, blue: corresponding to Figure S12B, orange: corresponding to Figure S12C) shown in Figure S12; the residuals for all three titrations are shown in grey. For better visualisation, the data was stacked with an offset to the *x*-axis.

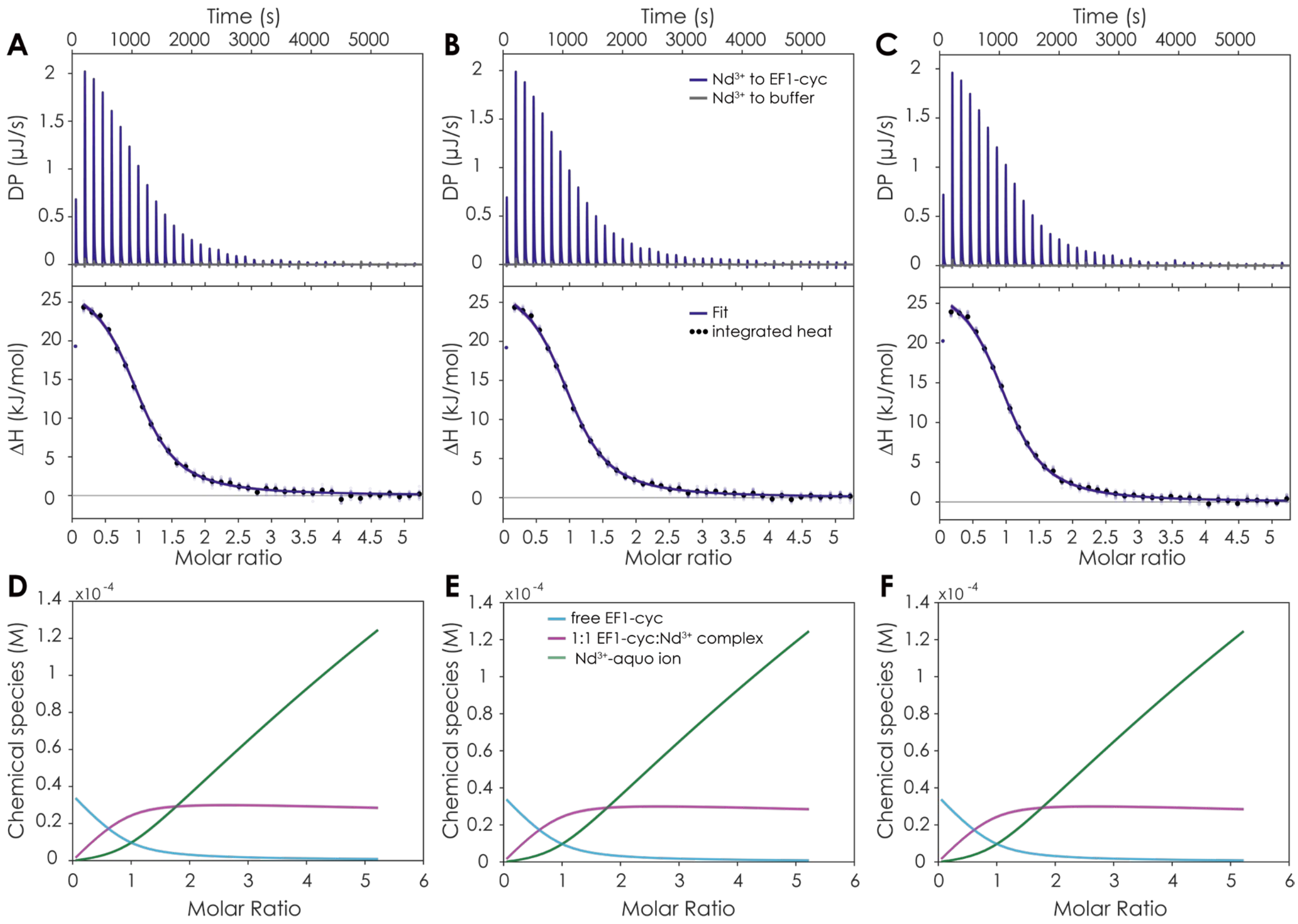

Figure S14 ITC data of EF1-cyc binding to Nd^{3+}. Thermogram, integrated heat, and used fit with a model for a 1:1 binding interaction obtained by titrating a 900 µM $NdCl_3$ solution to 30 µM peptide in triplicates **(A)** to **(C)**. The data was analysed globally; the first titration point was excluded from the analysis in all cases. The corresponding chemical species distribution for dataset **(A)** is shown in **(D)**, for **(B)** in **(E)** and for **(C)** in **(F)**. Experimental conditions: pH 6.6, 25 °C, 10 mM MOPSO buffer, 100 mM KCl. Overlayed lines/ symbols represent 100 MC runs which were used for error estimation (see Section 2.2).

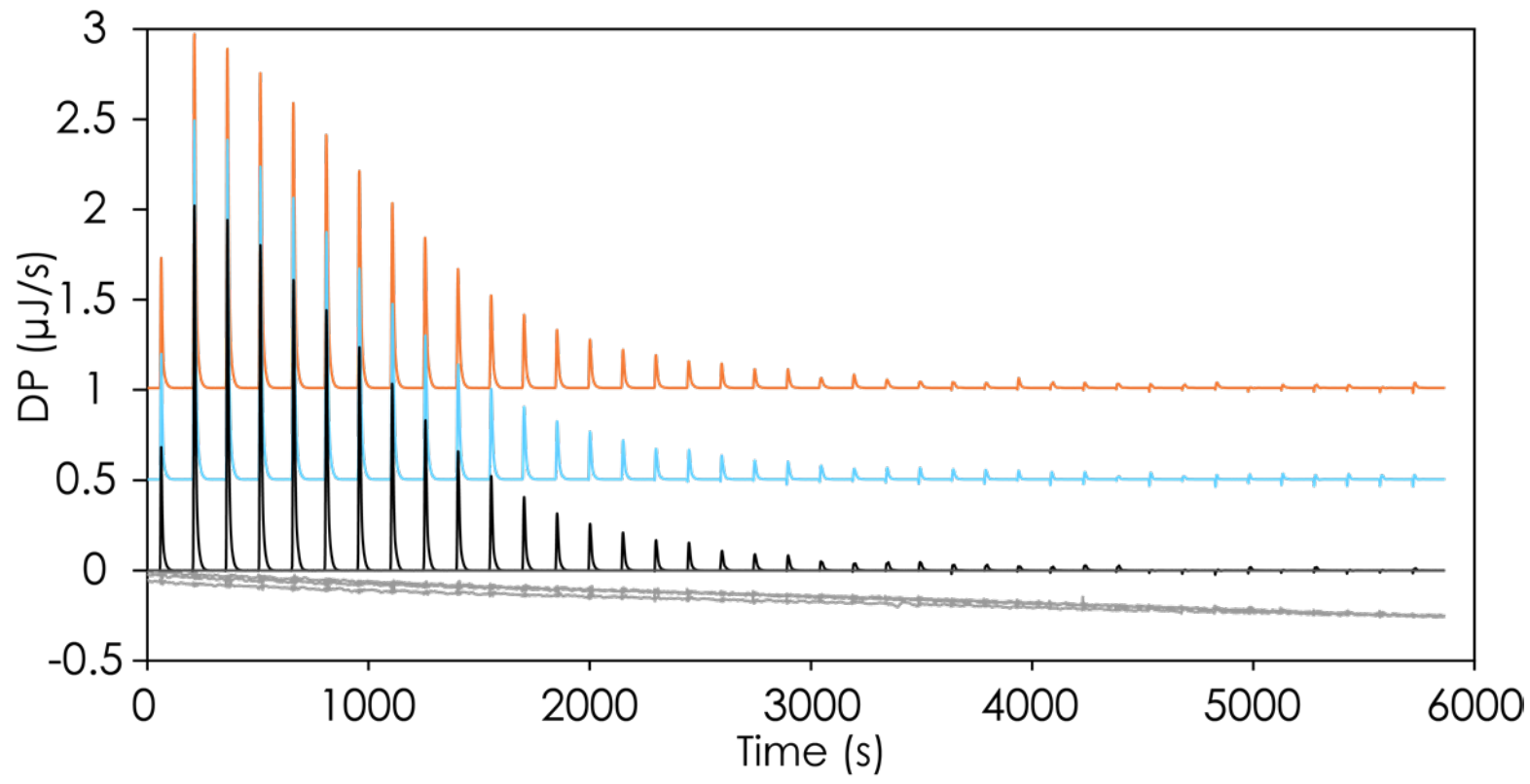

Figure S15 SVD-corrected thermograms of the 900 µM $NdCl_3$ to 30 µM EF1-cyc titrations performed in triplicates (black: corresponding to Figure S14A, blue: corresponding to Figure S14B, orange: corresponding to Figure S14C) shown in Figure S14; the residuals for all three titrations are shown in grey. For better visualisation, the data was stacked with an offset to the *x*-axis.

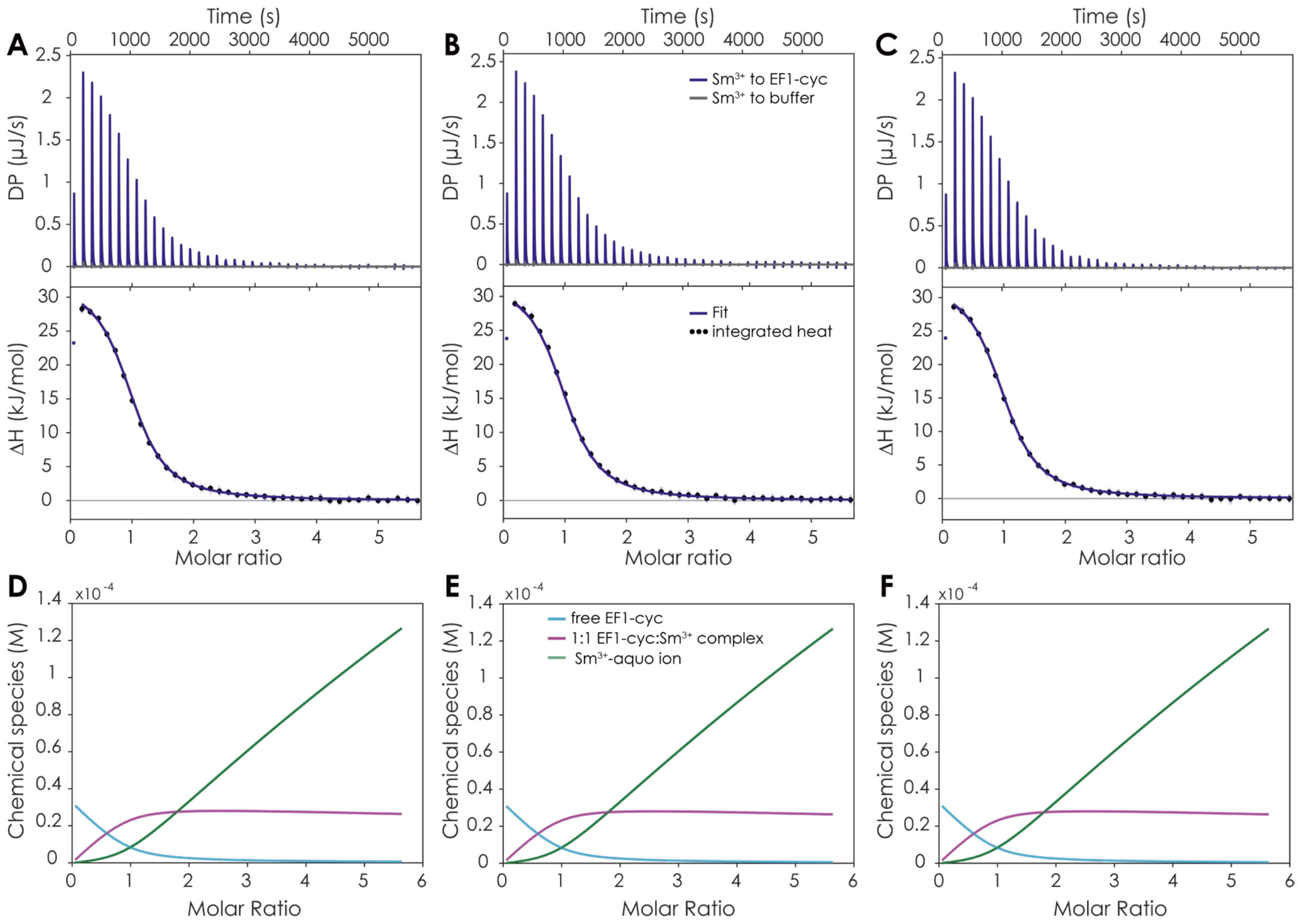

Figure S16 ITC data of EF1-cyc binding to Sm^{3+}. Thermogram, integrated heat, and used fit with a model for a 1:1 binding interaction obtained by titrating a 900 µM $SmCl_3$ solution to 30 µM peptide in triplicates **(A)** to **(C)**. The data was analysed globally; the first titration point was excluded from the analysis in all cases. The corresponding chemical species distribution for dataset **(A)** is shown in **(D)**, for **(B)** in **(E)** and for **(C)** in **(F)**. Experimental conditions: pH 6.6, 25 °C, 10 mM MOPSO buffer, 100 mM KCl. Overlayed lines/ symbols represent 100 MC runs which were used for error estimation (see Section 2.2).

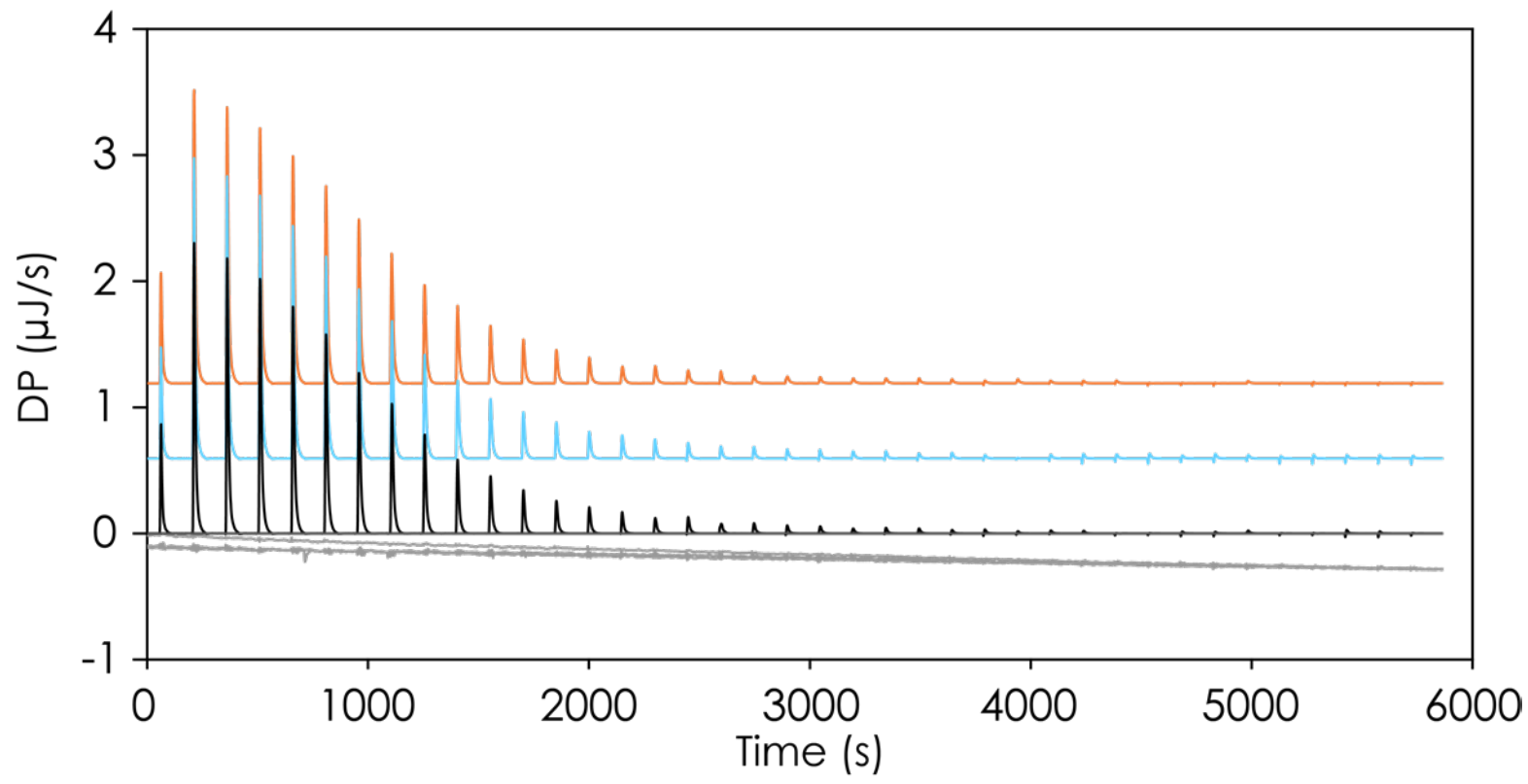

Figure S17 SVD-corrected thermograms of the 900 µM $SmCl_3$ to 30 µM EF1-cyc titrations performed in triplicates (black: corresponding to Figure S16A, blue: corresponding to Figure S16B, orange: corresponding to Figure S16C) shown in Figure S16; the residuals for all three titrations are shown in grey. For better visualisation, the data was stacked with an offset to the *x*-axis.

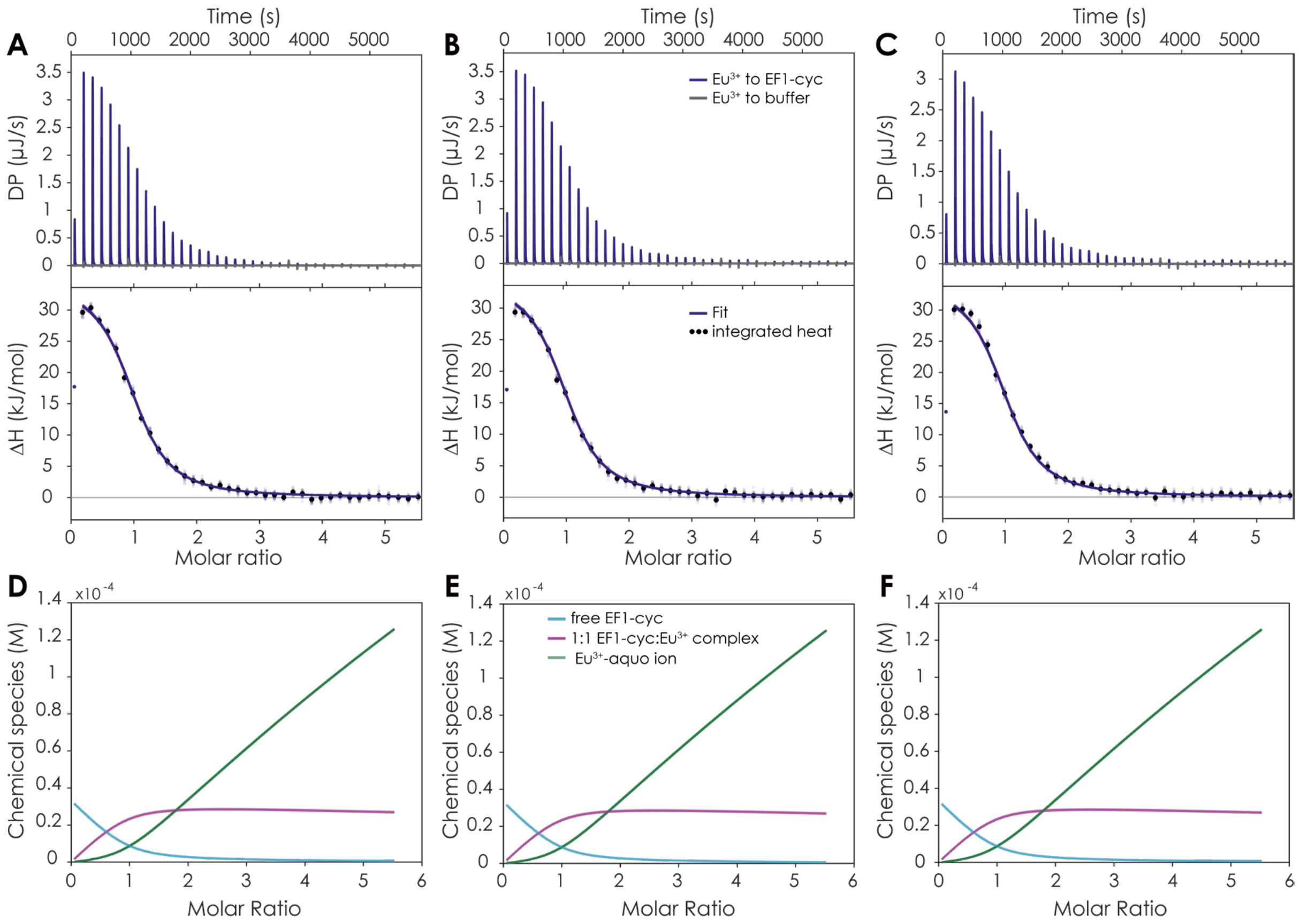

Figure S18 ITC data of EF1-cyc binding to Eu^{3+}. Thermogram, integrated heat, and used fit with a model for a 1:1 binding interaction obtained by titrating a 900 µM $EuCl_3$ solution to 30 µM peptide in triplicates **(A)** to **(C)**. The data was analysed globally; the first titration point was excluded from the analysis in all cases. The corresponding chemical species distribution for dataset **(A)** is shown in **(D)**, for **(B)** in **(E)** and for **(C)** in **(F)**. Experimental conditions: pH 6.6, 25 °C, 10 mM MOPSO buffer, 100 mM KCl. Overlayed lines/ symbols represent 100 MC runs which were used for error estimation (see Section 2.2).

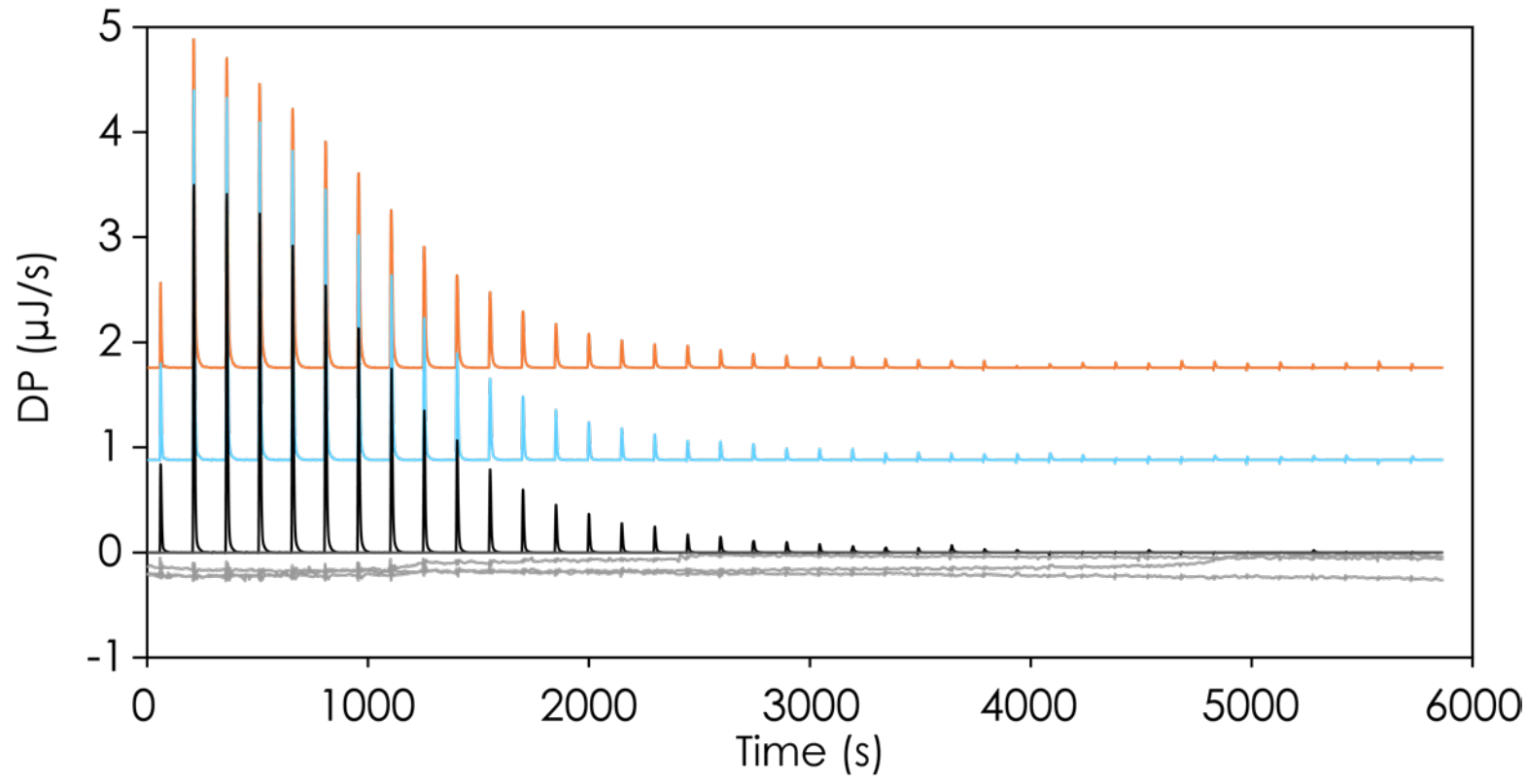

Figure S19 SVD-corrected thermograms of the 900 µM $EuCl_3$ to 30 µM EF1-cyc titrations performed in triplicates (black: corresponding to Figure S18A, blue: corresponding to Figure S18B, orange: corresponding to Figure S18C) shown in Figure S18; the residuals for all three titrations are shown in grey. For better visualisation, the data was stacked with an offset to the *x*-axis.

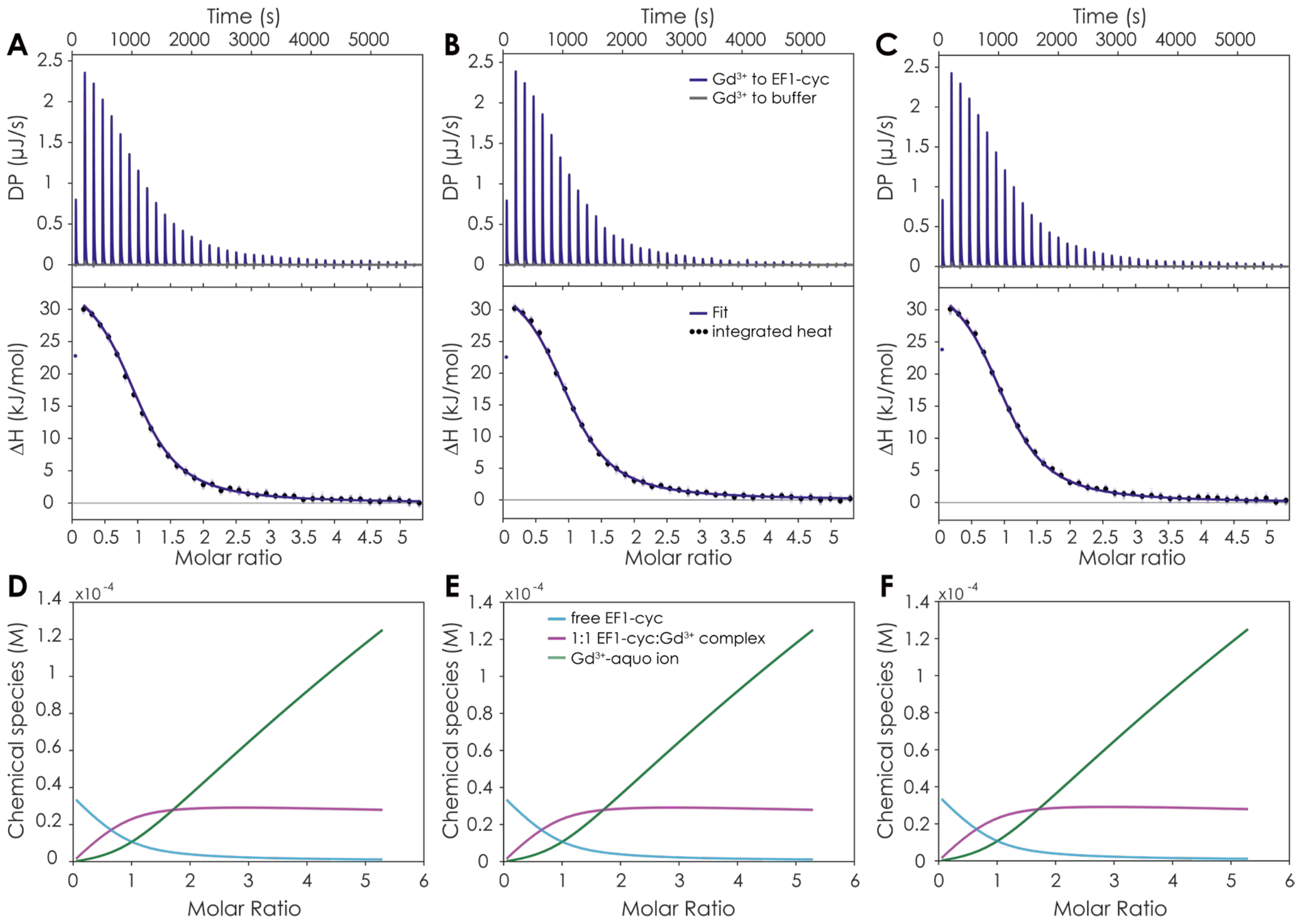

Figure S20 ITC data of EF1-cyc binding to Gd^{3+}. Thermogram, integrated heat, and used fit with a model for a 1:1 binding interaction obtained by titrating a 900 µM $GdCl_3$ solution to 30 µM peptide in triplicates **(A)** to **(C)**. The data was analysed globally; the first titration point was excluded from the analysis in all cases. The corresponding chemical species distribution for dataset **(A)** is shown in **(D)**, for **(B)** in **(E)** and for **(C)** in **(F)**. Experimental conditions: pH 6.6, 25 °C, 10 mM MOPSO buffer, 100 mM KCl. Overlayed lines/ symbols represent 100 MC runs which were used for error estimation (see Section 2.2).

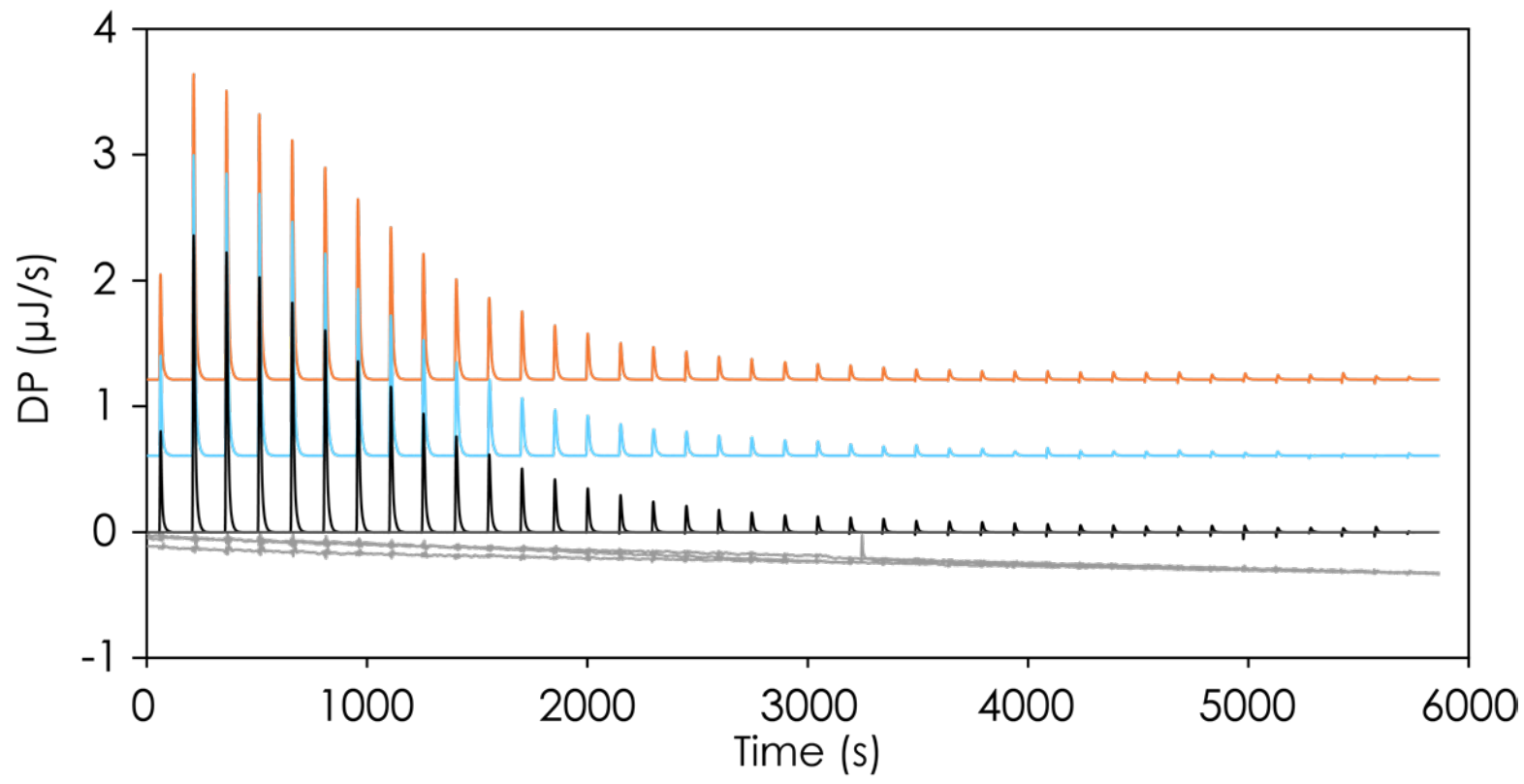

Figure S21 SVD-corrected thermograms of the 900 µM $GdCl_3$ to 30 µM EF1-cyc titrations performed in triplicates (black: corresponding to Figure S20A, blue: corresponding to Figure S20B, orange: corresponding to Figure S20C) shown in Figure S20; the residuals for all three titrations are shown in grey. For better visualisation, the data was stacked with an offset to the *x*-axis.

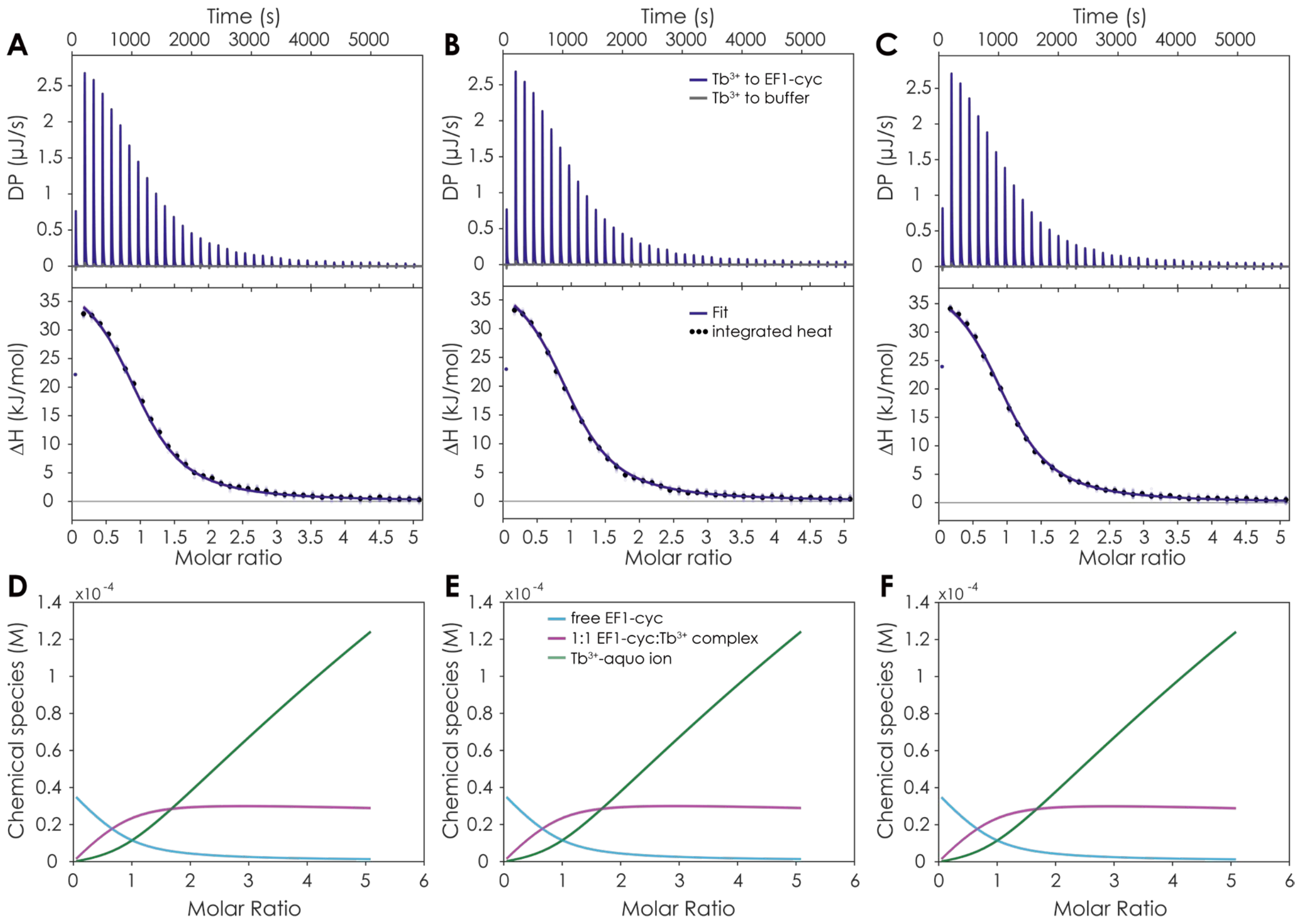

Figure S22 ITC data of EF1-cyc binding to Tb^{3+}. Thermogram, integrated heat, and used fit with a model for a 1:1 binding interaction obtained by titrating a 900 µM $TbCl_3$ solution to 30 µM peptide in triplicates **(A)** to **(C)**. The data was analysed globally; the first titration point was excluded from the analysis in all cases. The corresponding chemical species distribution for dataset **(A)** is shown in **(D)**, for **(B)** in **(E)** and for **(C)** in **(F)**. Experimental conditions: pH 6.6, 25 °C, 10 mM MOPSO buffer, 100 mM KCl. Overlayed lines/ symbols represent 100 MC runs which were used for error estimation (see Section 2.2).

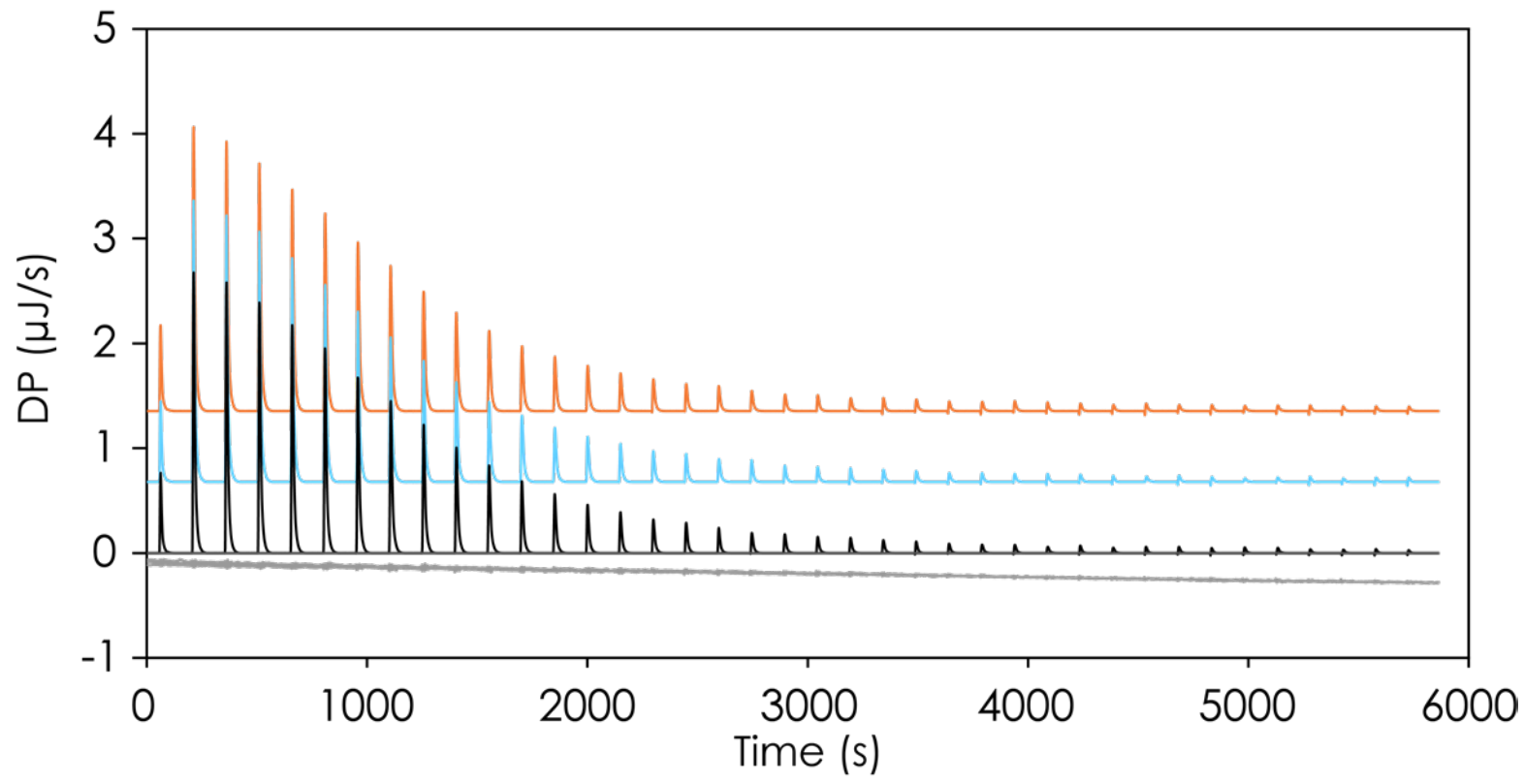

Figure S23 SVD-corrected thermograms of the 900 µM $TbCl_3$ to 30 µM EF1-cyc titrations performed in triplicates (black: corresponding to Figure S22A, blue: corresponding to Figure S22B, orange: corresponding to Figure S22C) shown in Figure S22; the residuals for all three titrations are shown in grey. For better visualisation, the data was stacked with an offset to the *x*-axis.

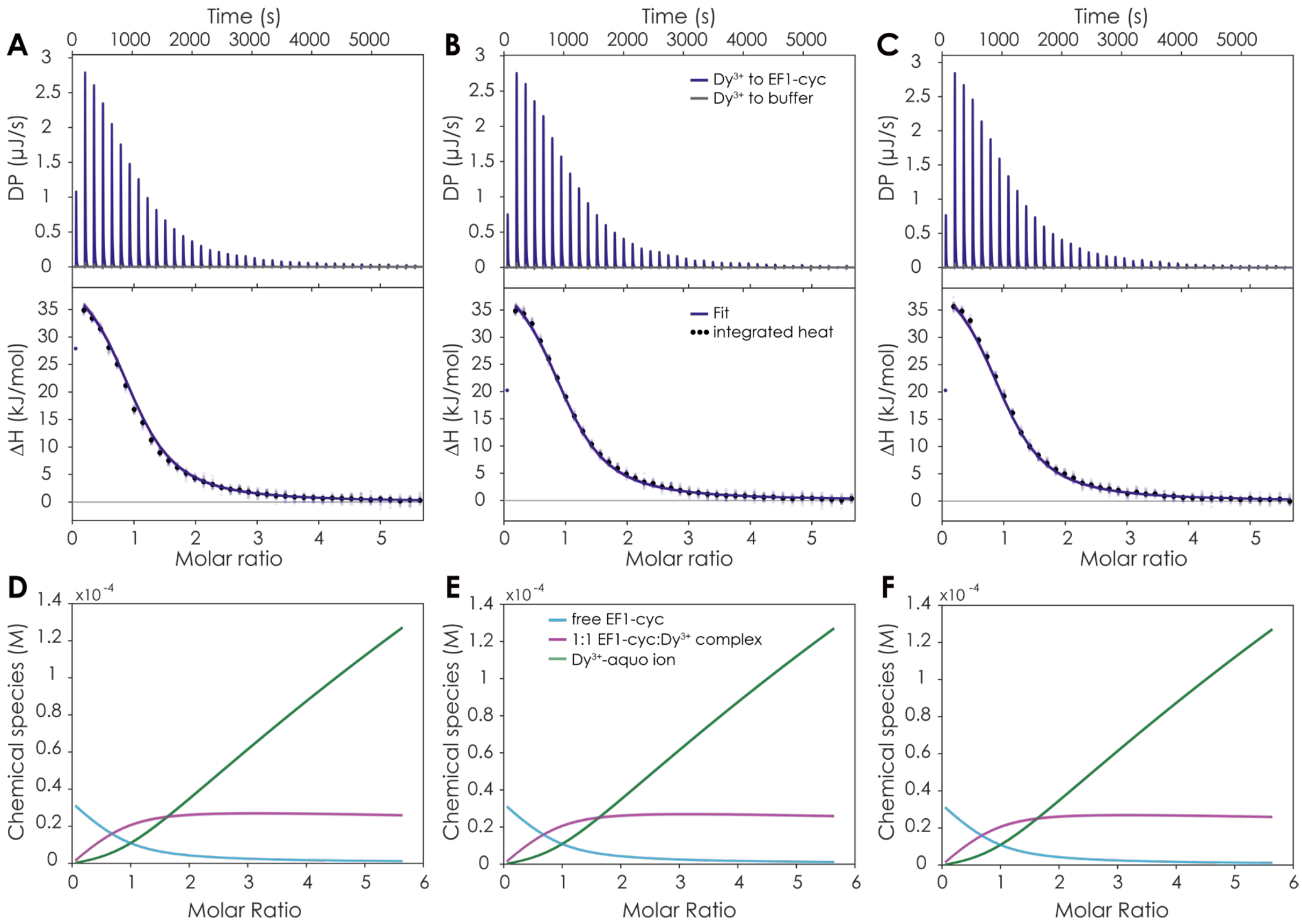

Figure S24 ITC data of EF1-cyc binding to Dy^{3+}. Thermogram, integrated heat, and used fit with a model for a 1:1 binding interaction obtained by titrating a 900 µM $DyCl_3$ solution to 30 µM peptide in triplicates **(A)** to **(C)**. The data was analysed globally; the first titration point was excluded from the analysis in all cases. The corresponding chemical species distribution for dataset **(A)** is shown in **(D)**, for **(B)** in **(E)** and for **(C)** in **(F)**. Experimental conditions: pH 6.6, 25 °C, 10 mM MOPSO buffer, 100 mM KCl. Overlayed lines/ symbols represent 100 MC runs which were used for error estimation (see Section 2.2).

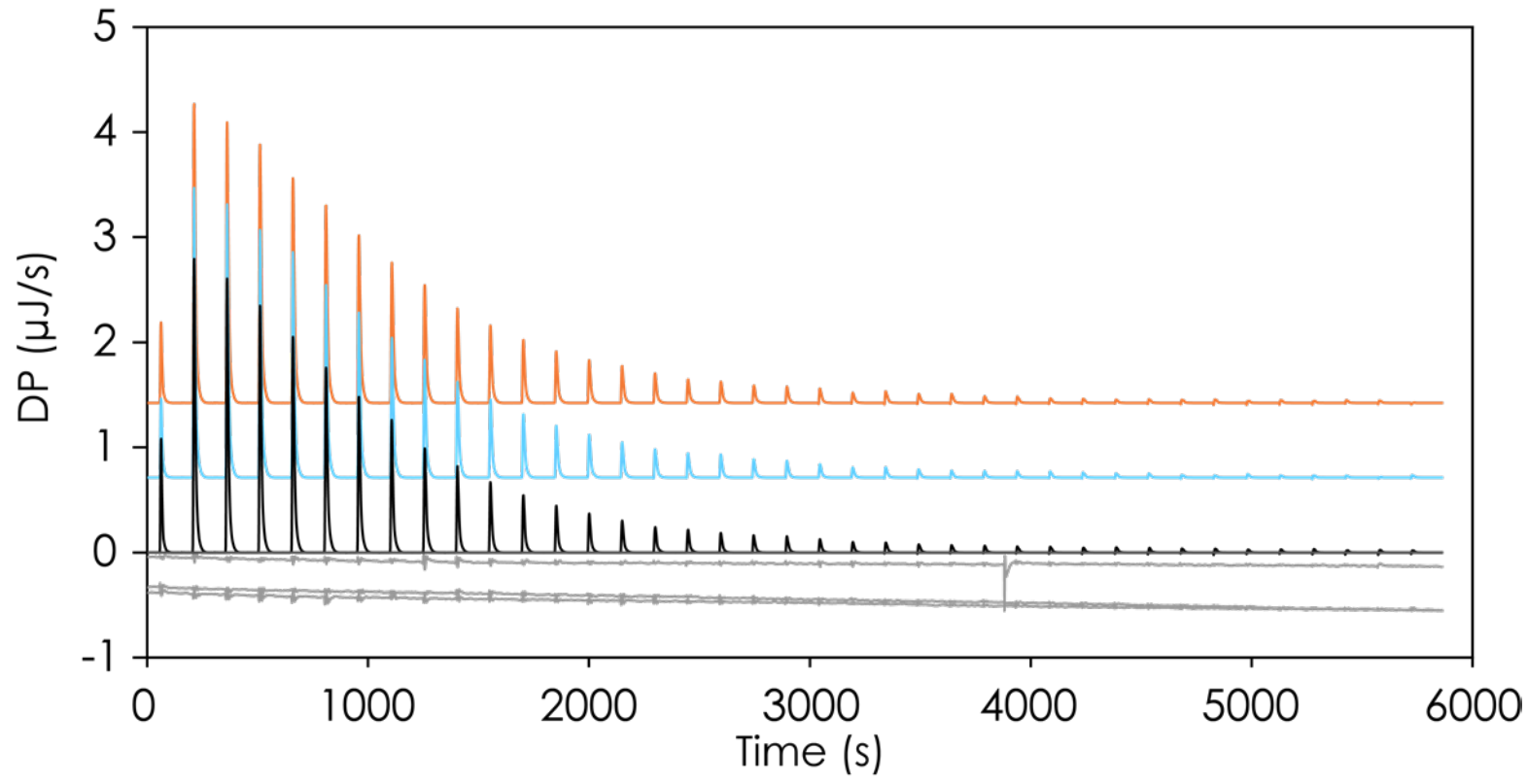

Figure S25 SVD-corrected thermograms of the 900 µM $DyCl_3$ to 30 µM EF1-cyc titrations performed in triplicates (black: corresponding to Figure S24A, blue: corresponding to Figure S24B, orange: corresponding to Figure S24C) shown in Figure S24; the residuals for all three titrations are shown in grey. For better visualisation, the data was stacked with an offset to the *x*-axis.

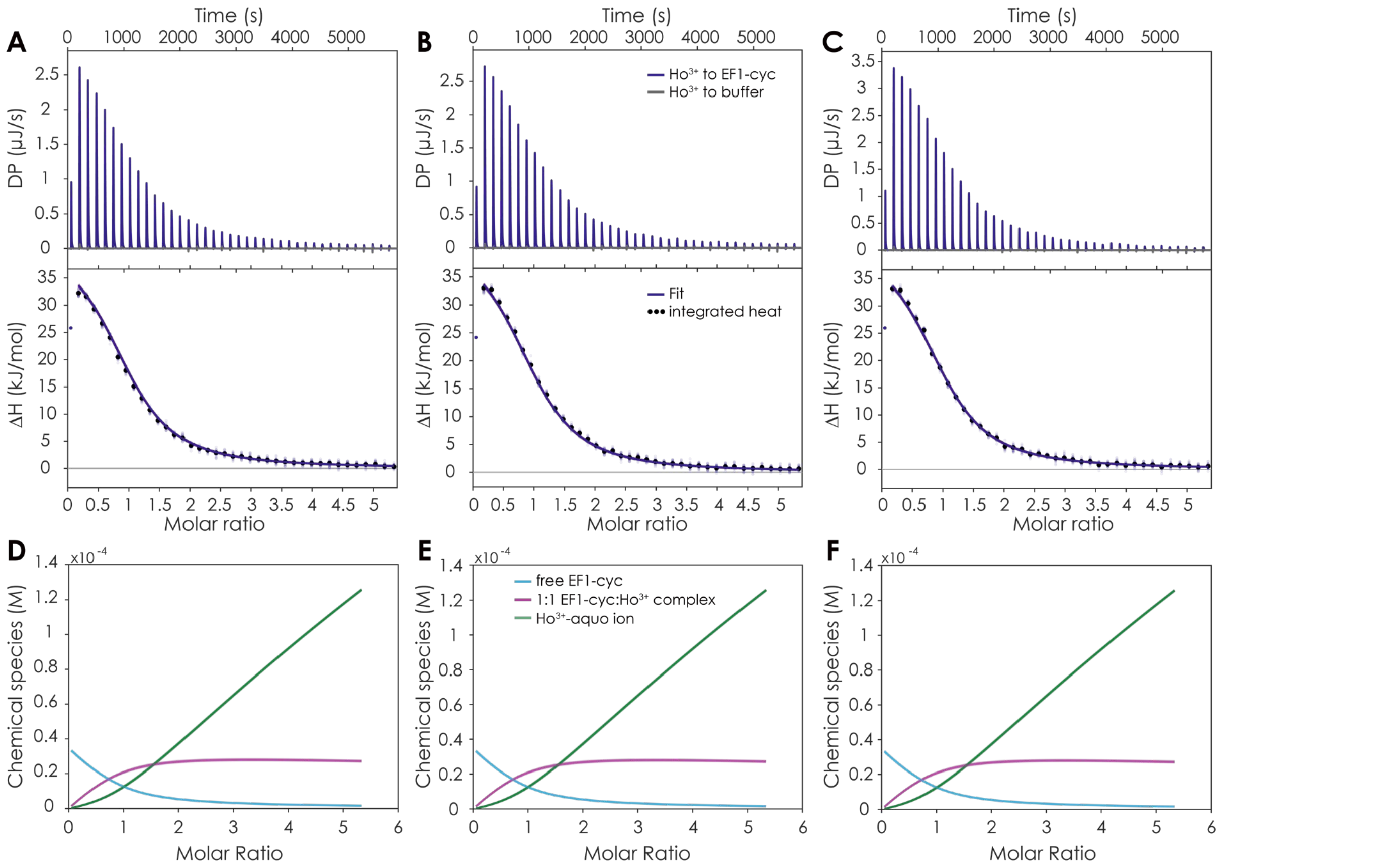

Figure S26 ITC data of EF1-cyc binding to Ho^{3+}. Thermogram, integrated heat, and used fit with a model for a 1:1 binding interaction obtained by titrating a 900 µM $HoCl_3$ solution to 30 µM peptide in triplicates **(A)** to **(C)**. The data was analysed globally; the first titration point was excluded from the analysis in all cases. The corresponding chemical species distribution for dataset **(A)** is shown in **(D)**, for **(B)** in **(E)** and for **(C)** in **(F)**. Experimental conditions: pH 6.6, 25 °C, 10 mM MOPSO buffer, 100 mM KCl. Overlayed lines/ symbols represent 100 MC runs which were used for error estimation (see Section 2.2).

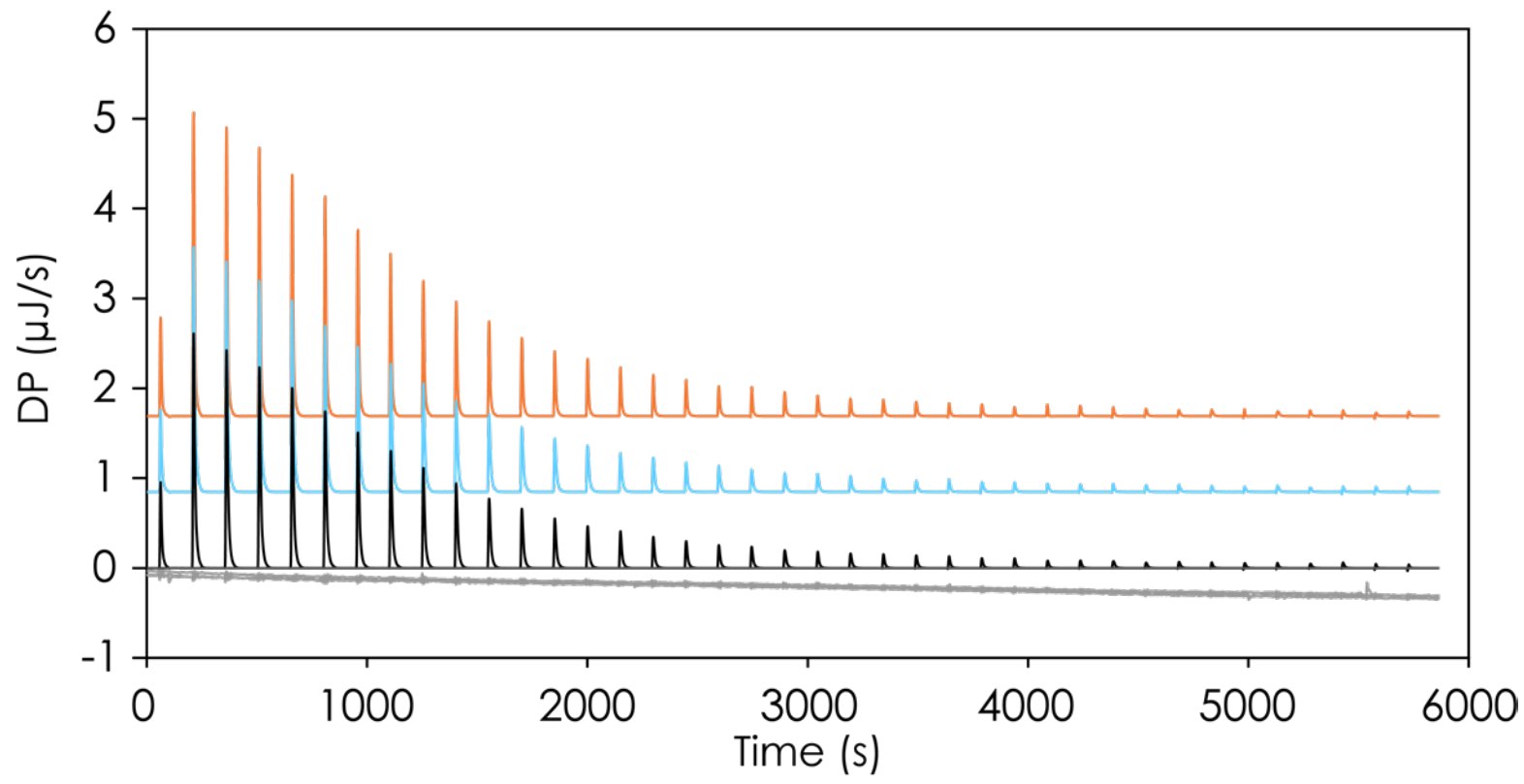

Figure S27 SVD-corrected thermograms of the 900 µM $HoCl_3$ to 30 µM EF1-cyc titrations performed in triplicates (black: corresponding to Figure S26A, blue: corresponding to Figure S26B, orange: corresponding to Figure S26C) shown in Figure S26; the residuals for all three titrations are shown in grey. For better visualisation, the data was stacked with an offset to the *x*-axis.

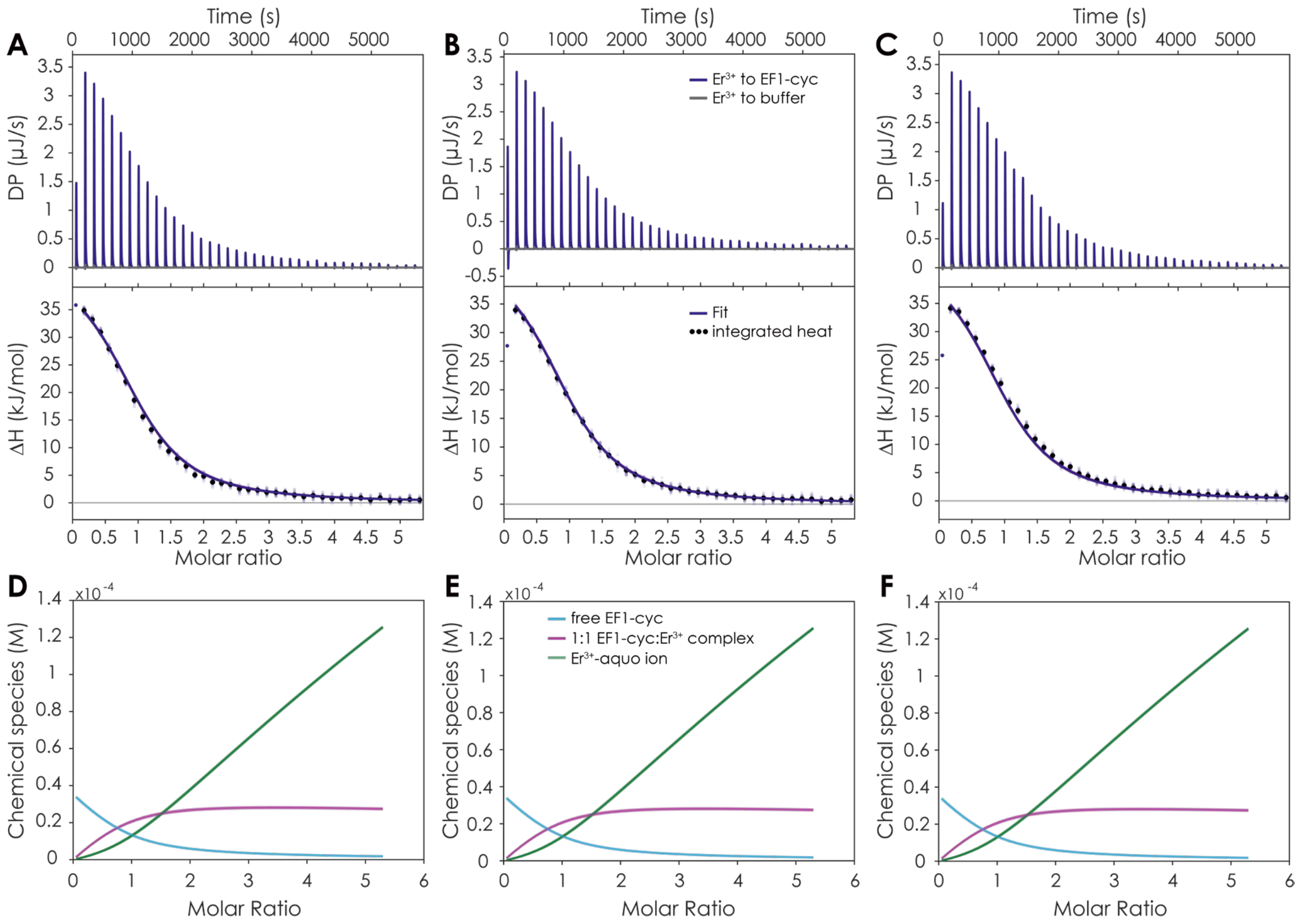

Figure S28 ITC data of EF1-cyc binding to Er^{3+}. Thermogram, integrated heat, and used fit with a model for a 1:1 binding interaction obtained by titrating a 900 µM $ErCl_3$ solution to 30 µM peptide in triplicates **(A)** to **(C)**. The data was analysed globally; the first titration point was excluded from the analysis in all cases. The corresponding chemical species distribution for dataset **(A)** is shown in **(D)**, for **(B)** in **(E)** and for **(C)** in **(F)**. Experimental conditions: pH 6.6, 25 °C, 10 mM MOPSO buffer, 100 mM KCl. Overlayed lines/ symbols represent 100 MC runs which were used for error estimation (see Section 2.2).

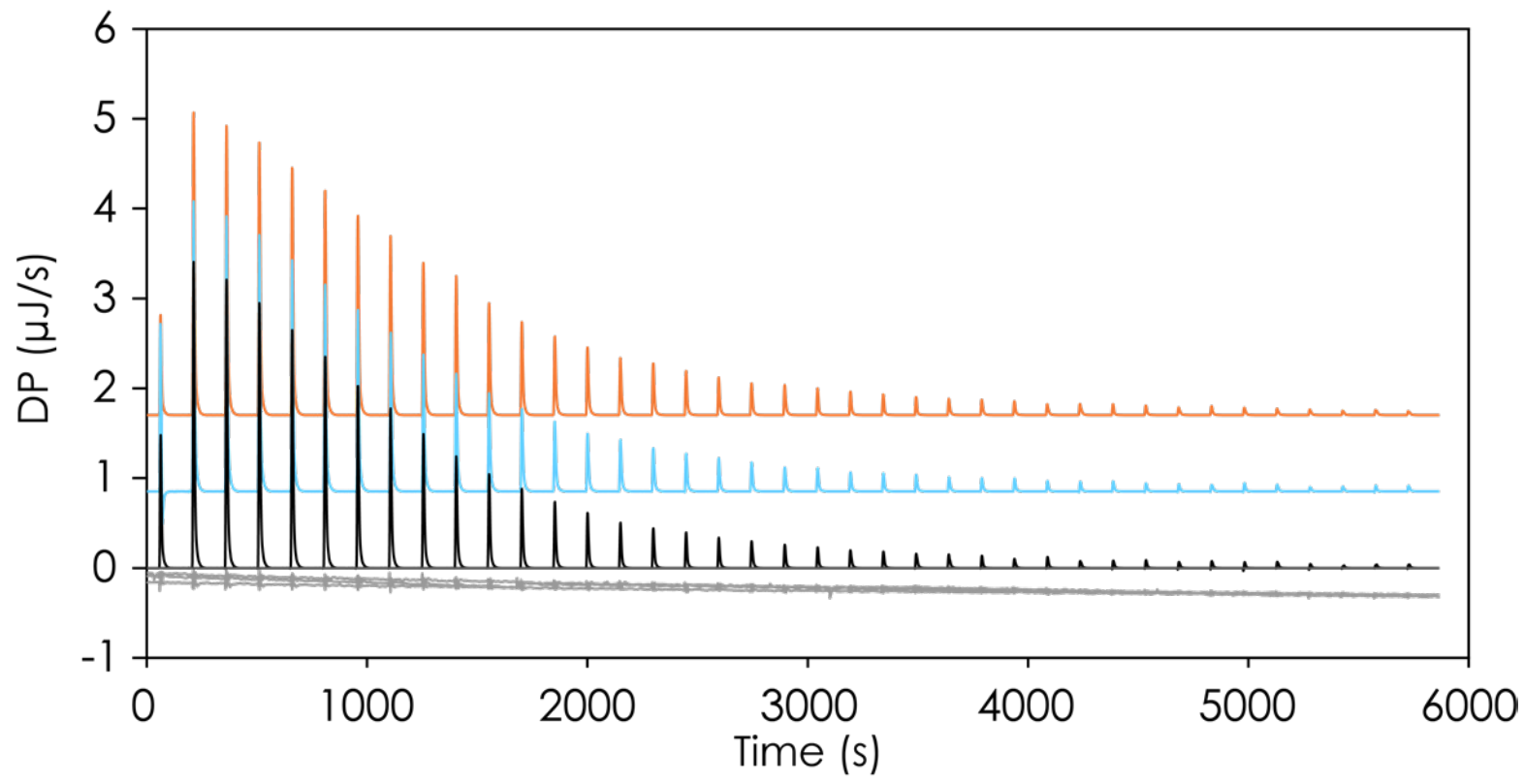

Figure S29 SVD-corrected thermograms of the 900 µM $ErCl_3$ to 30 µM EF1-cyc titrations performed in triplicates (black: corresponding to Figure S28A, blue: corresponding to Figure S28B, orange: corresponding to Figure S28C) shown in Figure S28; the residuals for all three titrations are shown in grey. For better visualisation, the data was stacked with an offset to the *x*-axis.

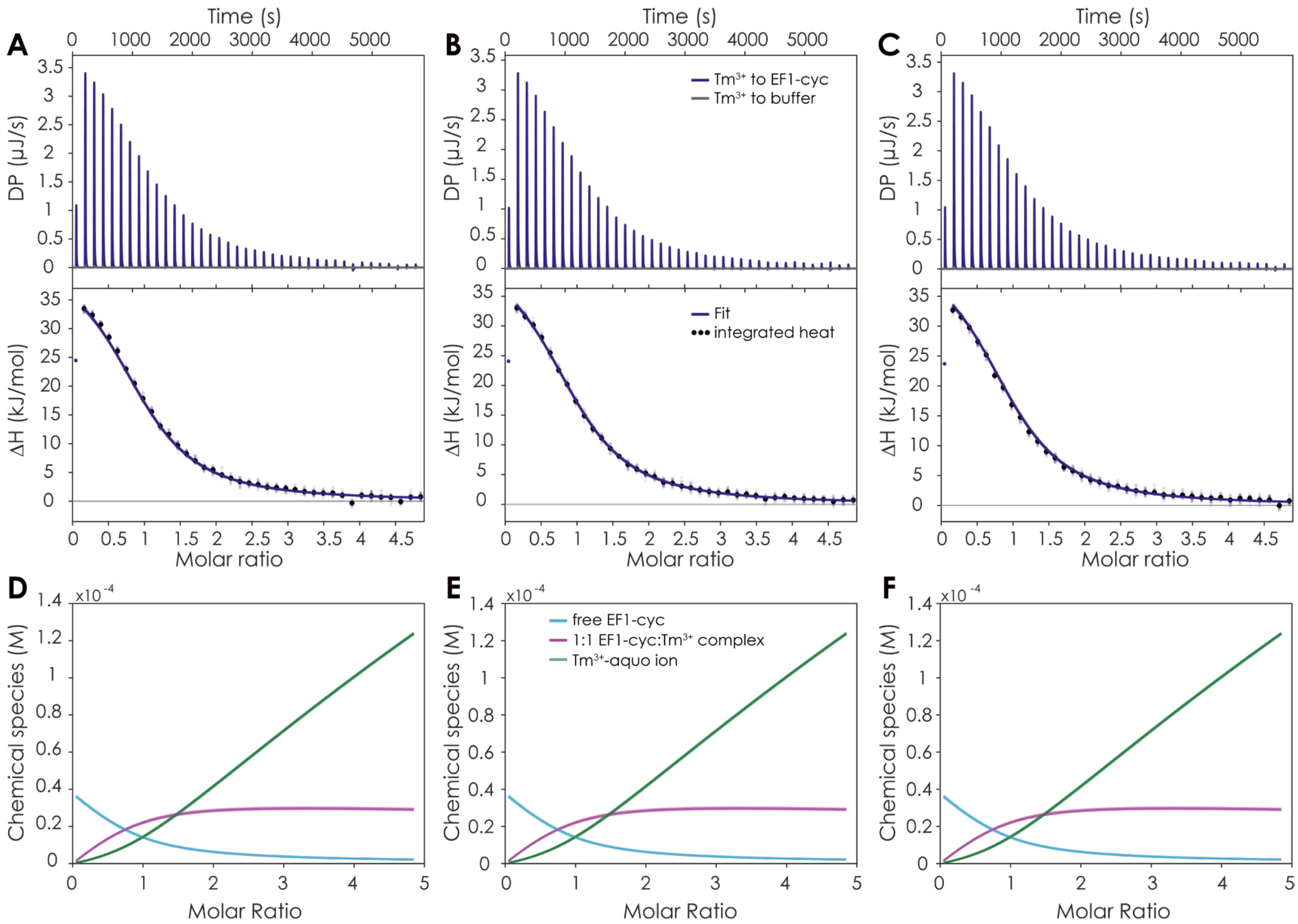

Figure S30 ITC data of EF1-cyc binding to Tm^{3+}. Thermogram, integrated heat, and used fit with a model for a 1:1 binding interaction obtained by titrating a 900 µM $TmCl_3$ solution to 30 µM peptide in triplicates **(A)** to **(C)**. The data was analysed globally; the first titration point was excluded from the analysis in all cases. The corresponding chemical species distribution for dataset **(A)** is shown in **(D)**, for **(B)** in **(E)** and for **(C)** in **(F)**. Experimental conditions: pH 6.6, 25 °C, 10 mM MOPSO buffer, 100 mM KCl. Overlayed lines/ symbols represent 100 MC runs which were used for error estimation (see Section 2.2).

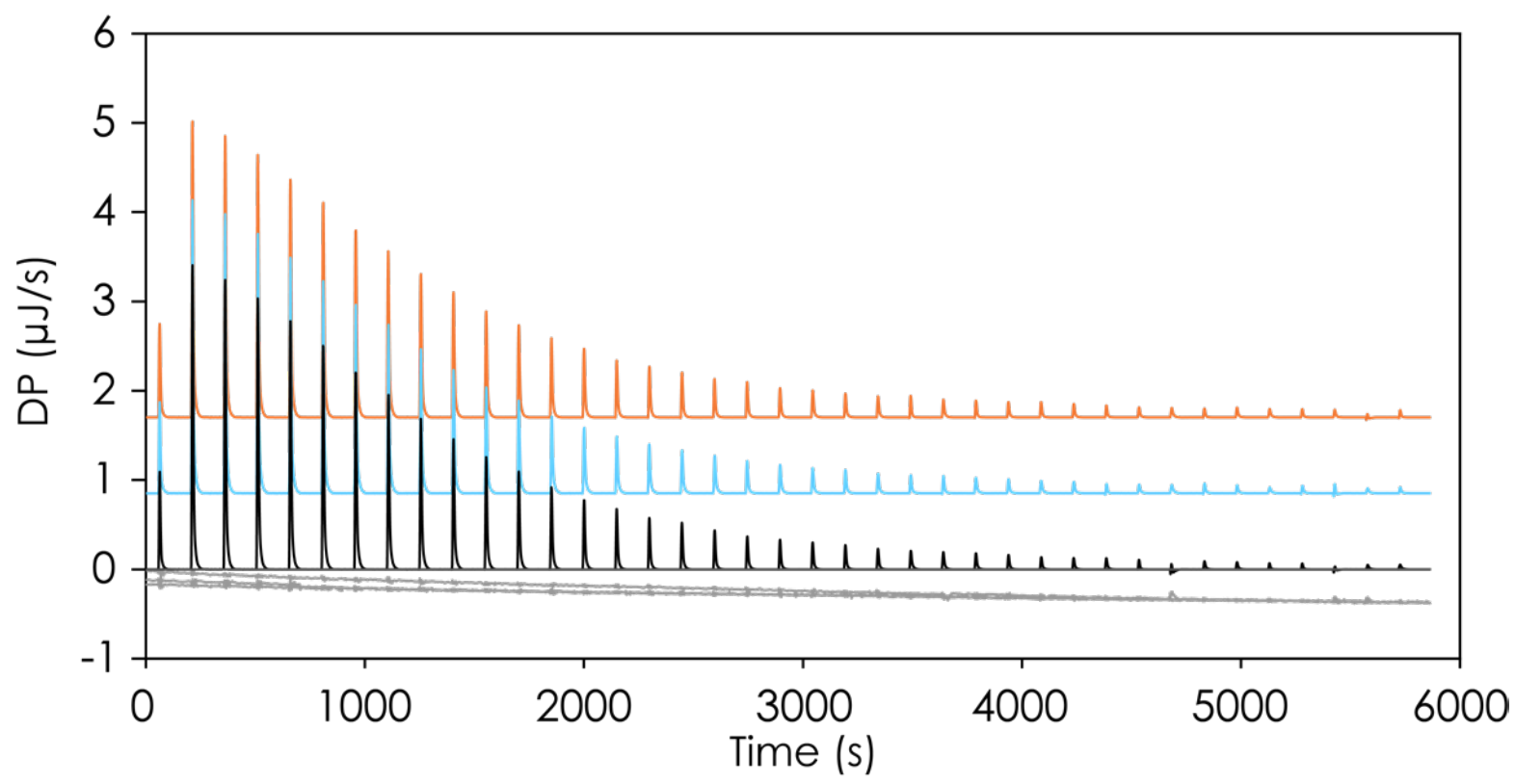

Figure S31 SVD-corrected thermograms of the 900 µM $TmCl_3$ to 30 µM EF1-cyc titrations performed in triplicates (black: corresponding to Figure S30A, blue: corresponding to Figure S30B, orange: corresponding to Figure S30C) shown in Figure S30; the residuals for all three titrations are shown in grey. For better visualisation, the data was stacked with an offset to the *x*-axis.

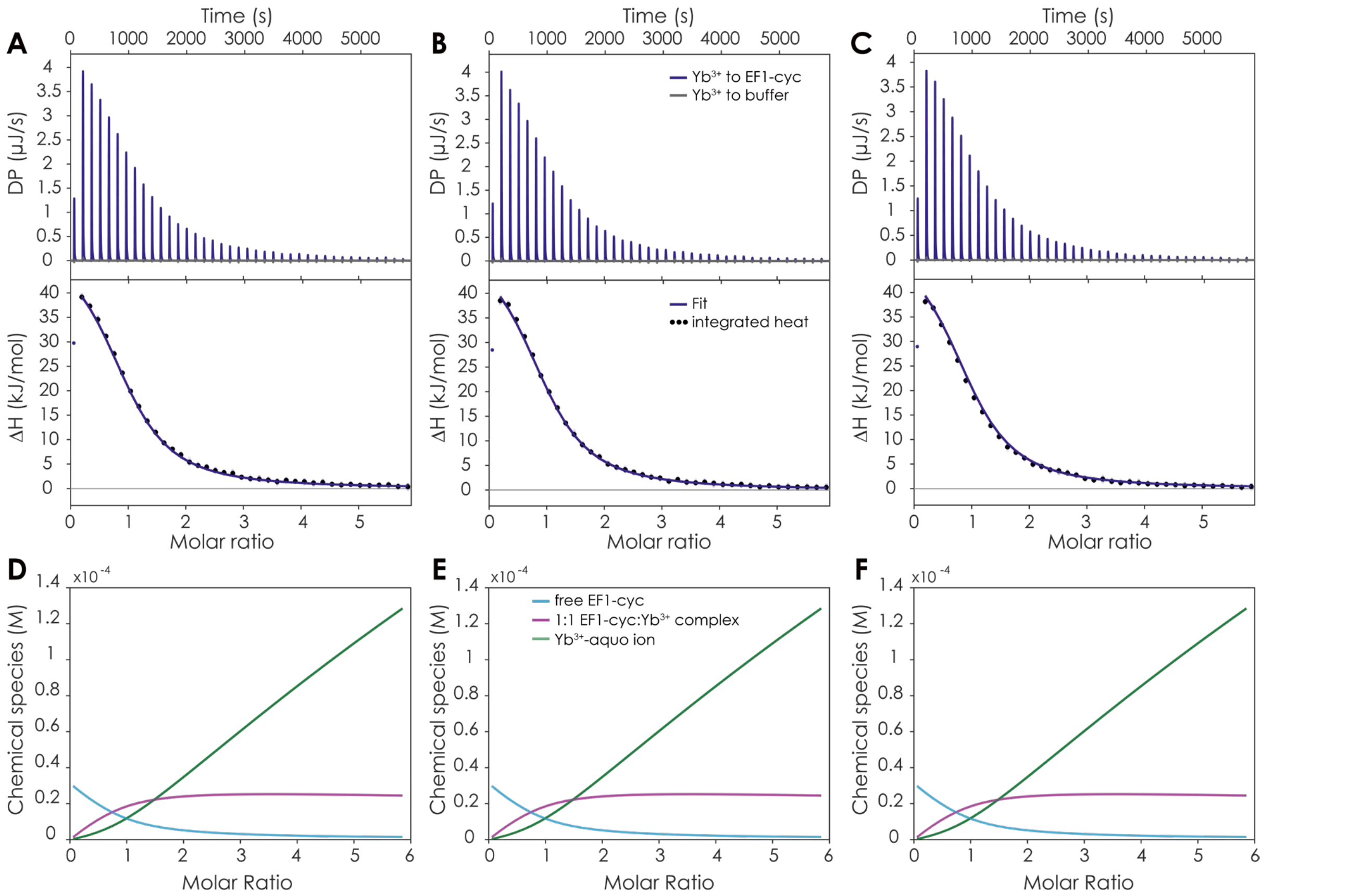

Figure S32 ITC data of EF1-cyc binding to Yb^{3+}. Thermogram, integrated heat, and used fit with a model for a 1:1 binding interaction obtained by titrating a 900 µM $YbCl_3$ solution to 30 µM peptide in triplicates **(A)** to **(C)**. The data was analysed globally; the first titration point was excluded from the analysis in all cases. The corresponding chemical species distribution for dataset **(A)** is shown in **(D)**, for **(B)** in **(E)** and for **(C)** in **(F)**. Experimental conditions: pH 6.6, 25 °C, 10 mM MOPSO buffer, 100 mM KCl. Overlayed lines/ symbols represent 100 MC runs which were used for error estimation (see Section 2.2).

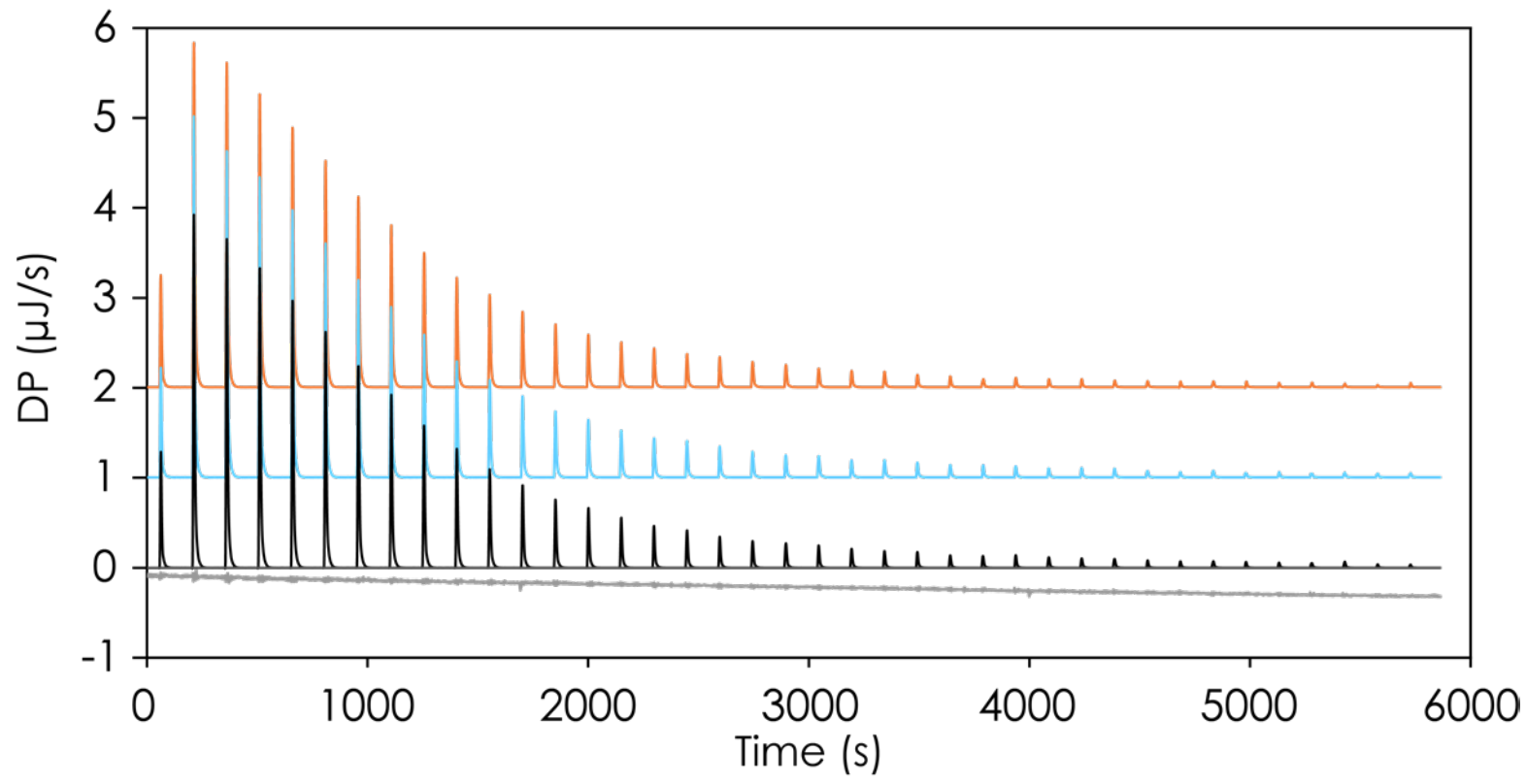

Figure S33 SVD-corrected thermograms of the 900 µM $YbCl_3$ to 30 µM EF1-cyc titrations performed in triplicates (black: corresponding to Figure S32A, blue: corresponding to Figure S32B, orange: corresponding to Figure S32C) shown in Figure S32; the residuals for all three titrations are shown in grey. For better visualisation, the data was stacked with an offset to the *x*-axis.

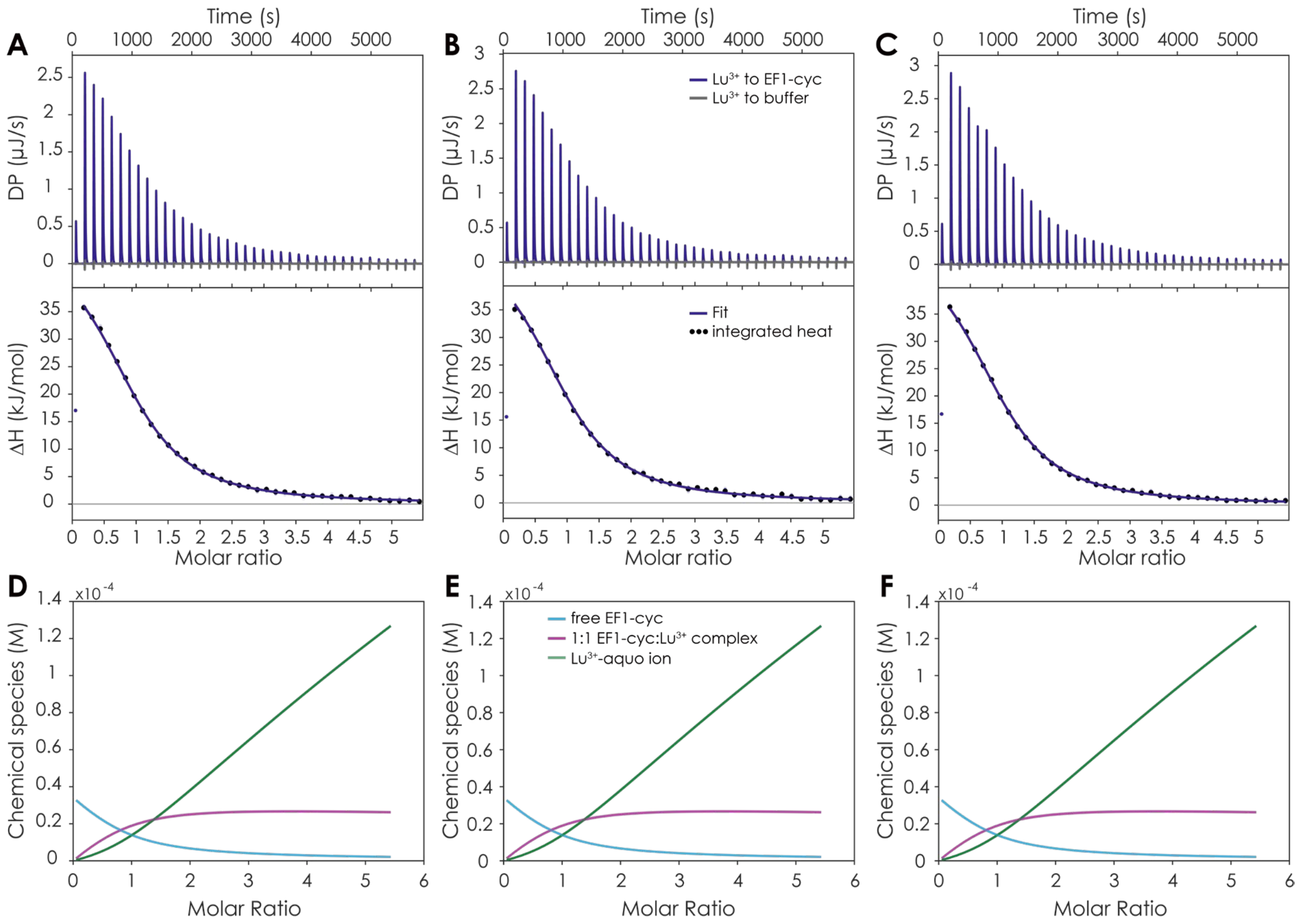

Figure S34 ITC data of EF1-cyc binding to Lu^{3+}. Thermogram, integrated heat, and used fit with a model for a 1:1 binding interaction obtained by titrating a 900 µM $LuCl_3$ solution to 30 µM peptide in triplicates **(A)** to **(C)**. The data was analysed globally; the first titration point was excluded from the analysis in all cases. The corresponding chemical species distribution for dataset **(A)** is shown in **(D)**, for **(B)** in **(E)** and for **(C)** in **(F)**. Experimental conditions: pH 6.6, 25 °C, 10 mM MOPSO buffer, 100 mM KCl. Overlayed lines/ symbols represent 100 MC runs which were used for error estimation (see Section 2.2).

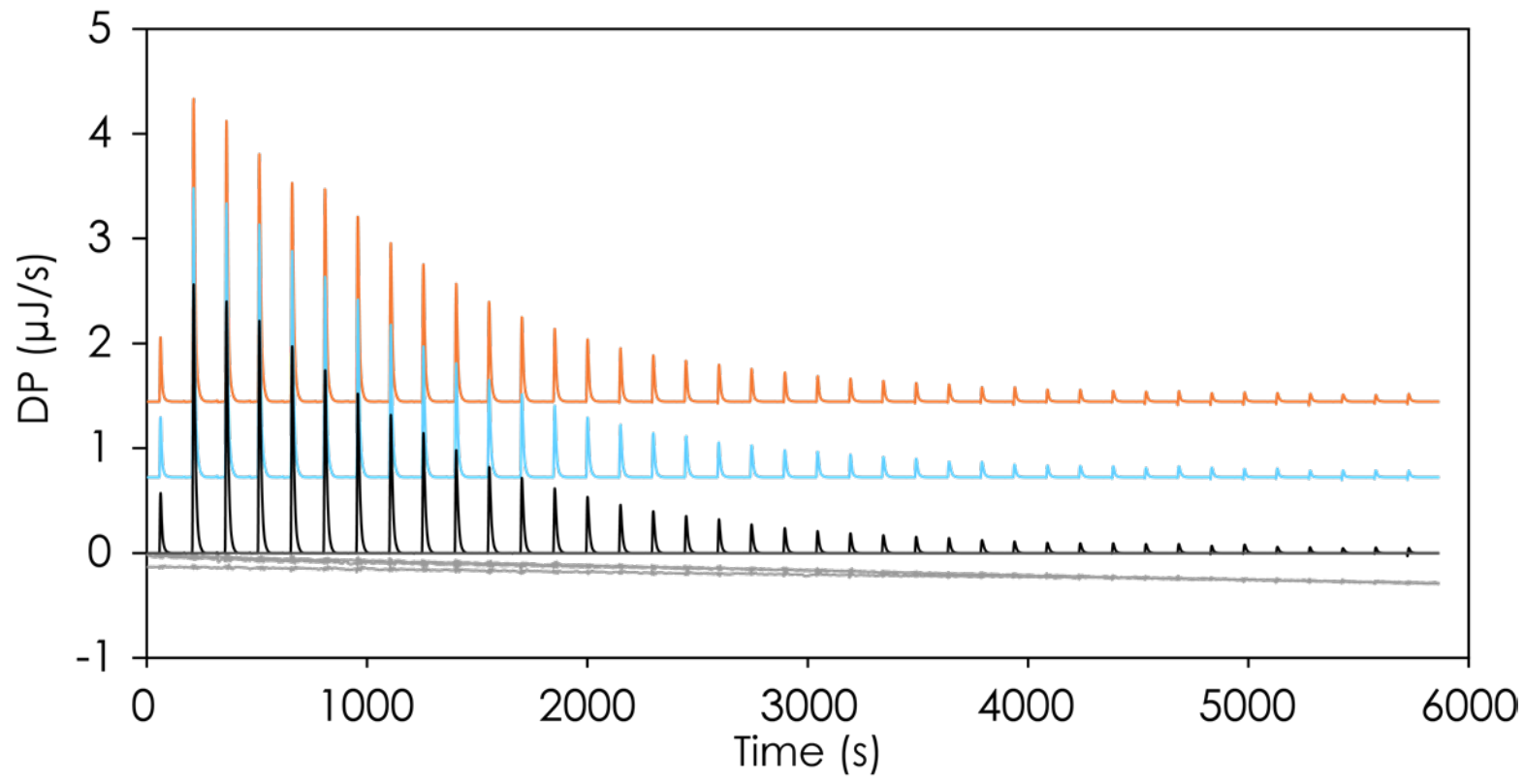

Figure S35 SVD-corrected thermograms of the 900 µM $LuCl_3$ to 30 µM EF1-cyc titrations performed in triplicates (black: corresponding to Figure S34A, blue: corresponding to Figure S34B, orange: corresponding to Figure S34C) shown in Figure S34; the residuals for all three titrations are shown in grey. For better visualisation, the data was stacked with an offset to the *x*-axis.

4.2 Supplementary TRLFS Data

Table S5 Supplementary data to Table 1 in the main manuscript. The data corresponds to the graphs shown in Figure 2 in the main manuscript and to Figures S36 and S37.

	**τ (µs) Eu-aquo ion**
EF1-cyc	111 ± 1
EF1-R-cyc	110 ± 1

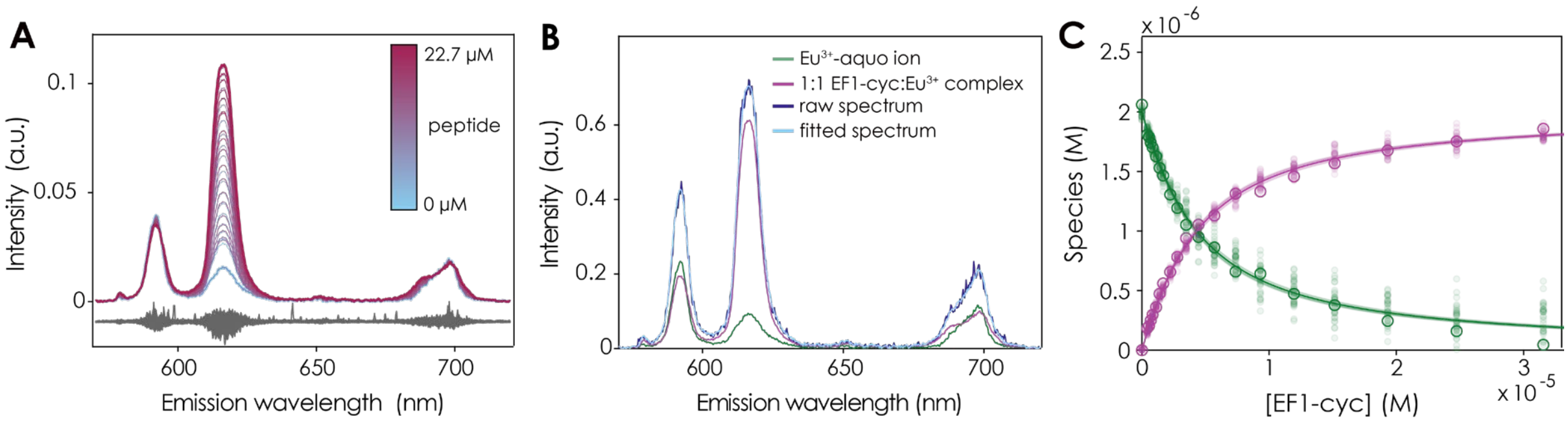

Figure S36 TRLFS titration experiment of 50 µM EF1-cyc to 2 µM Eu^{3+}. Experimental conditions: 10 mM MOPSO, 100 mM KCl, pH 6.6, λ_{ex}(Eu) = 394 nm. The experiment was performed in duplicates and analysed globally. **(A)** t_0 spectra of the PARAFAC model normalised to the $^5D_0 \rightarrow {}^7F_1$ transition; removed noise shown in grey. **(B)** Deconvoluted spectra of a representative step showing the spectrum of the Eu^{3+}-aquo ion and the 1:1 complex. **(C)** Speciation obtained for the second independently measured titration series. The colour code shown in **B** applies as well to **C**; transparent lines/ symbols represent MC runs used for error estimation (see Section 2.3 for more details).

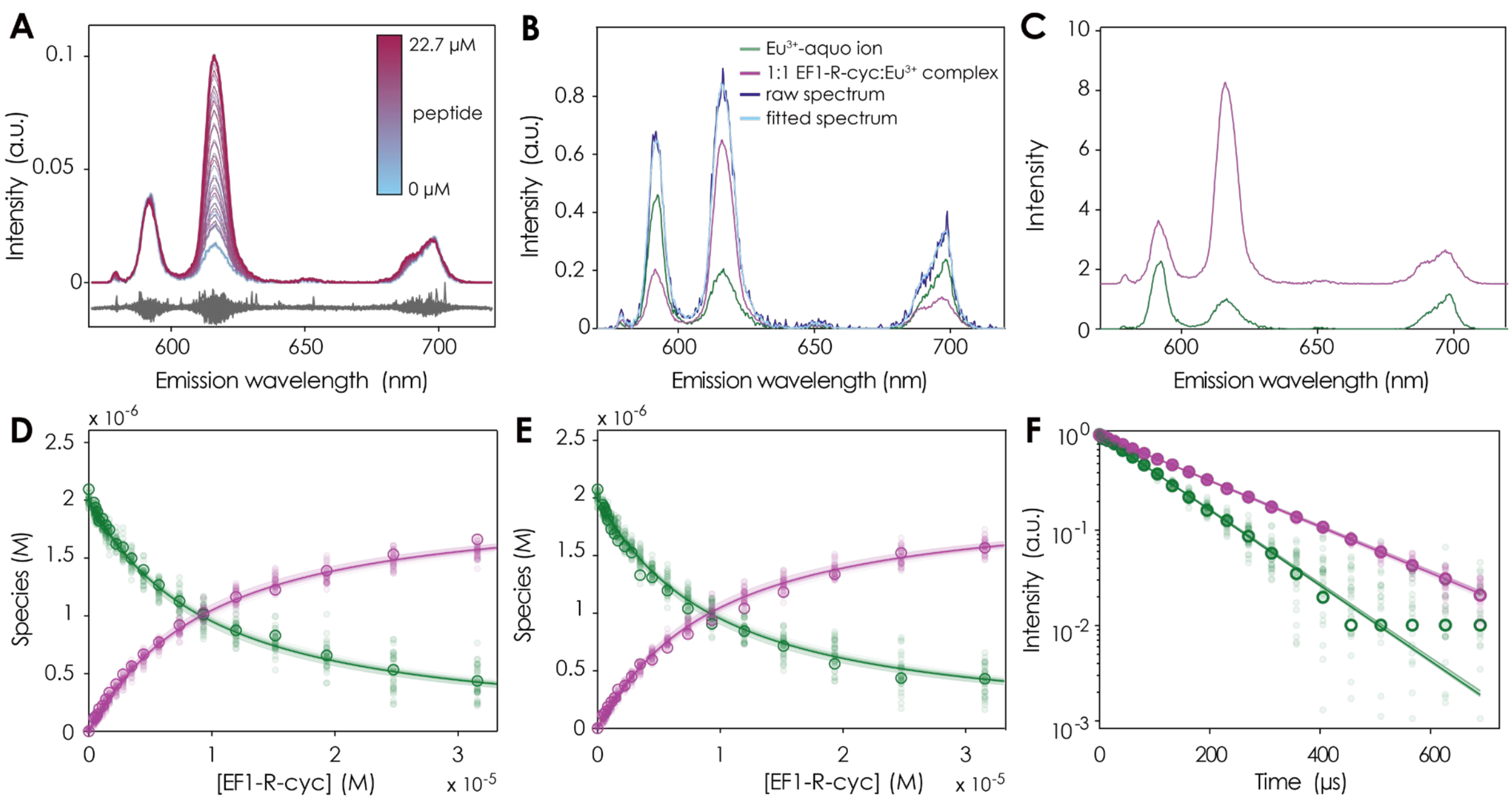

Figure S37 TRLFS titration experiment of 50 µM EF1-R-cyc to 2 µM Eu^{3+}. Experimental conditions: 10 mM MOPSO, 100 mM KCl, pH 6.6, λ_{ex}(Eu) = 394 nm. The experiment was performed in duplicates and analysed globally. **(A)** t_0 spectra of the PARAFAC model normalised to the $^5D_0 \rightarrow {}^7F_1$ transition; removed noise shown in grey. **(B)** Deconvoluted spectra of a representative step showing the spectrum of the Eu^{3+}-aquo ion and the 1:1 complex. **(C)** Stacked spectra of the two different species: Eu^{3+}-aquo ion and the 1:1 EF1-R-cyc:Eu^{3+} complex. **(D)** and **(E)** Speciations obtained for the two independently measured titration series. **(F)** Lifetimes of the observed species. The colour code shown in **B** applies as well to **C**, **D**, **E** and **F**; transparent lines/ symbols represent MC runs used for error estimation (see Section 2.3 for more details).

Table S6 K_D values and luminescence lifetimes (**τ**) obtained from the EF1-G-cyc to $EuCl_3$ TRLFS titration experiment. The corresponding analysis is shown in Figure S38.

	K_D (µM)	τ (µs) 1:1 complex	τ (µs) Eu-aquo ion
EF1-G-cyc	1.5 ± 0.1	311 ± 7	111 ± 1

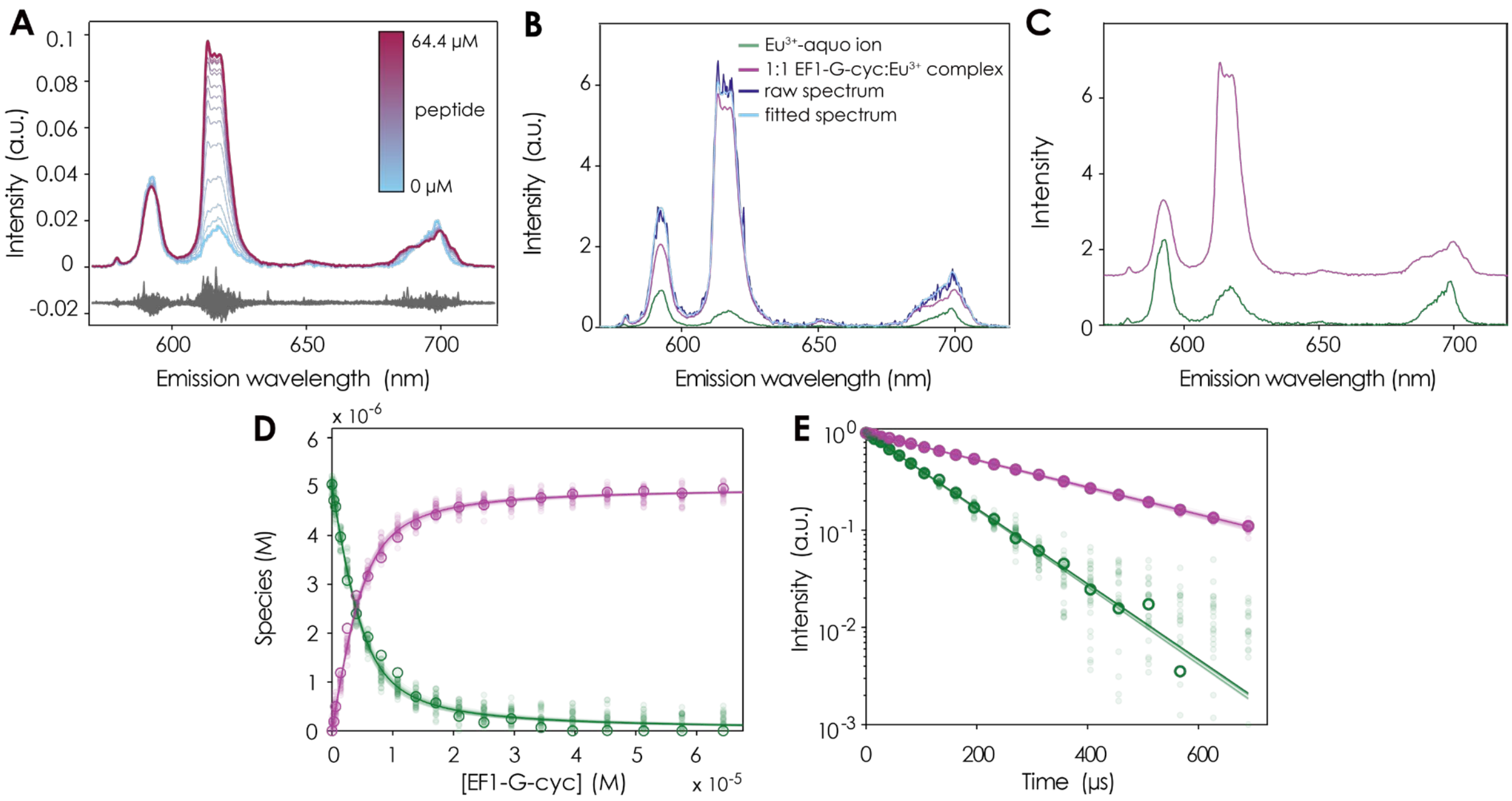

Figure S38 TRLFS titration experiment of 250 µM EF1-G-cyc to 5 µM Eu^{3+}. Experimental conditions: 10 mM MOPSO 100 mM KCl, pH 6.6, λ_{ex}(Eu) = 394 nm. **(A)** t_0 spectra of the PARAFAC model normalised to the $^5D_0 \rightarrow {}^7F_1$ transition; removed noise shown in grey. **(B)** Deconvoluted spectra of a representative step showing the spectrum of the Eu^{3+}-aquo ion and the 1:1 complex. **(C)** Stacked spectra of the two different species: Eu^{3+}-aquo ion and the 1:1 EF1-G-cyc:Eu^{3+} complex. **(D)** Speciation of the two different species. **(E)** Lifetimes of the observed species. The colour code shown in **B** applies as well to **C**, **D**, and **E**; shaded lines/ symbols represent MC runs used for error estimation (see Section 2.3 for more details).

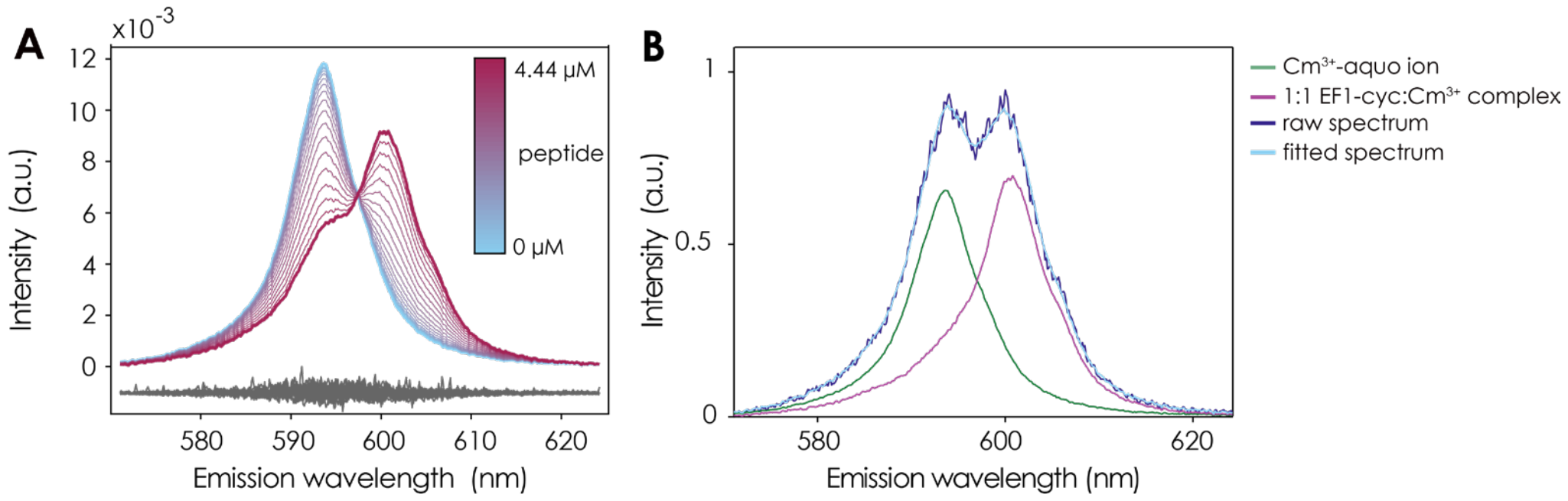

Figure S39 Supplementary graphs to the data shown in Figure 3 in the main manuscript of the 20 µM EF1-cyc to 100 nM Cm^{3+} TRLFS titration experiment. Experimental conditions: 10 mM MOPSO, 100 mM KCl, pH 6.0, λ_{ex}(Cm) = 396 nm. **(A)** t_0 spectra of the PARAFAC model area-normalised; removed noise shown in grey. **(B)** Deconvoluted spectra of a representative step showing the spectrum of the Cm^{3+}-aquo ion and the 1:1 complex.

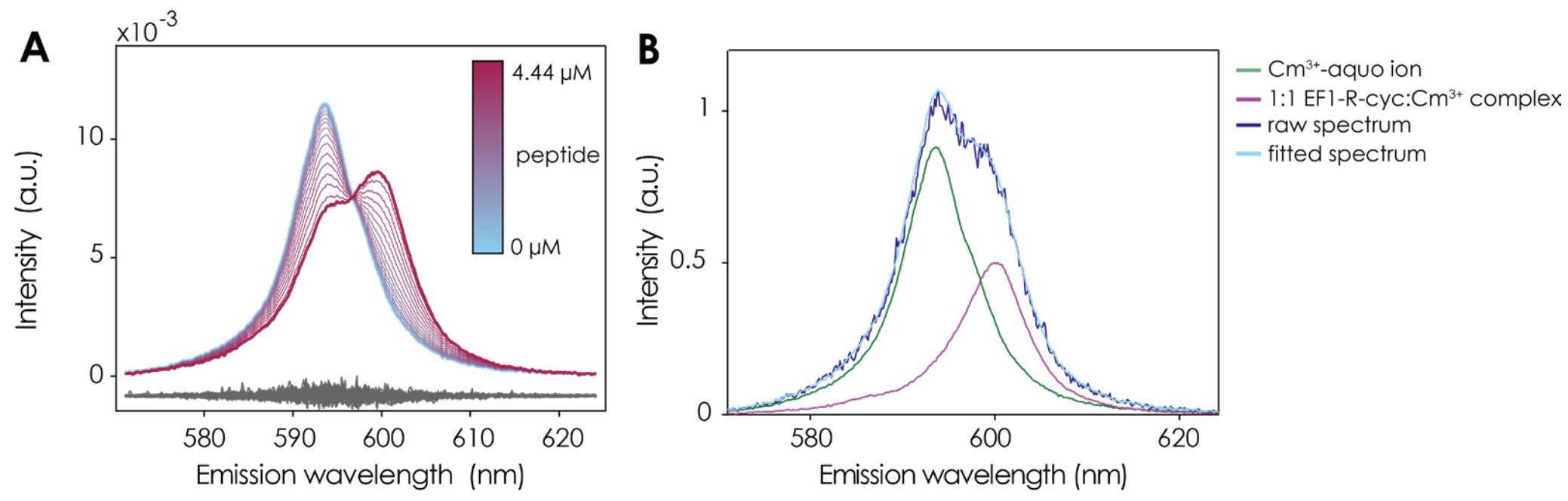

Figure S40 Supplementary graphs to the data shown in Figure 3 in the main manuscript of the 20 µM EF1-R-cyc to 100 nM Cm^{3+} TRLFS titration experiment. Experimental conditions: 10 mM MOPSO, 100 mM KCl, pH 6.0, λ_{ex}(Cm) = 396 nm. **(A)** t_0 spectra of the PARAFAC model area-normalised; removed noise shown in grey. **(B)** Deconvoluted spectra of a representative step showing the spectrum of the Cm^{3+}-aquo ion and the 1:1 complex.

Table S7 K_D values for EF1-cyc, EF1-R-cyc and EF1-G-cyc with different Lns determined by TRLFS using a competition experiment with Eu^{3+}. The values correspond to Figure 7 in the main manuscript and Figures S41 and Figure S42. The given uncertainties are the result of the used Monte Carlo approach during the analysis.

	**K_D (µM)**		
**Ln^{3+}**	**EF1-cyc**	**EF1-R-cyc**	**EF1-G-cyc**
**La**	6.3 ± 1.4	16.6 ± 3.3	4.7 ± 1.2
**Ce**	4.1 ± 0.9	11.0 ± 2.1	2.6 ± 0.6
**Pr**	3.5 ± 0.8	8.5 ± 1.6	2.9 ± 0.7
**Nd**	3.1 ± 0.7	6.9 ± 1.3	2.3 ± 0.5
**Sm**	2.4 ± 0.5	5.4 ± 1.0	1.3 ± 0.3
**Eu**	3.3 ± 0.2	8.1 ± 0.5	1.5 ± 0.1
**Gd**	3.8 ± 0.9	8.2 ± 1.6	2.7 ± 0.7
**Tb**	5.2 ± 1.2	10.6 ± 2.0	2.1 ± 0.5
**Dy**	5.0 ± 1.1	9.9 ± 1.9	2.5 ± 0.6
**Ho**	6.1 ± 1.4	12.5 ± 2.4	2.6 ± 0.6
**Er**	6.9 ± 1.5	12.6 ± 2.4	2.7 ± 0.7
**Tm**	6.8 ± 1.5	12.2 ± 2.3	4.3 ± 1.1
**Yb**	5.9 ± 1.3	9.8 ± 1.8	2.6 ± 0.6
**Lu**	9.1 ± 2.1	13.6 ± 2.6	4.6 ± 1.2

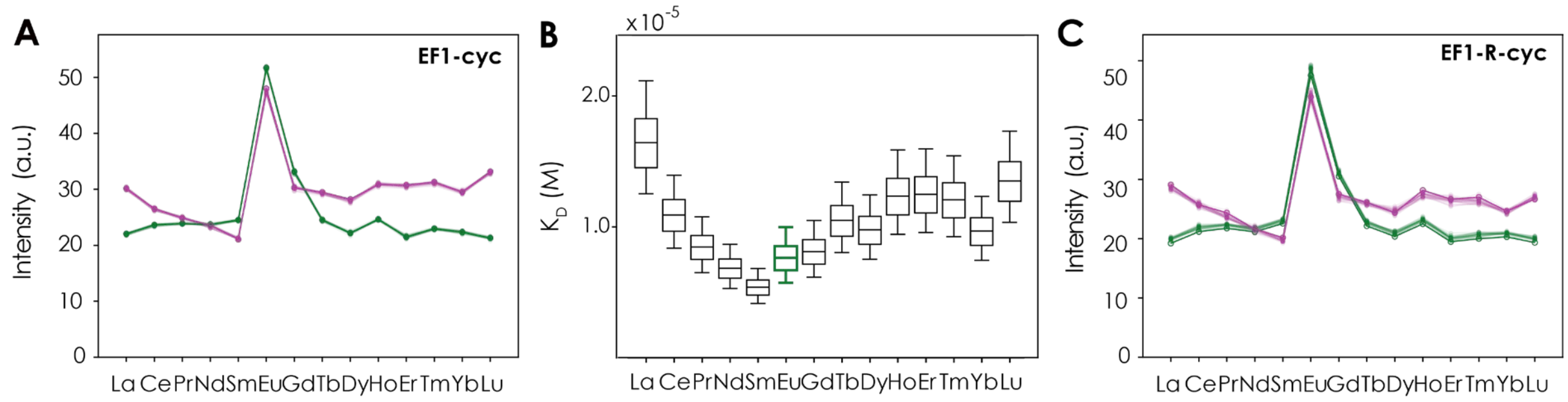

Figure S41 Lanthanide competition experiment between different Lns (except Pm) *vs.* Eu^{3+} with EF1-cyc and EF1-R-cyc. **(A)** PARAFAC (solid line) and MC (transparent lines) distribution of the Ln-series for the competition series of the peptides EF1-cyc shown in Figure 7C in the main manuscript. **(B)** Binding affinities, shown as box plot (median and quartiles), for the peptide EF1-R-cyc and **(C)** the corresponding PARAFAC (solid line) and MC (transparent lines) distribution of the Ln-series for the 1:1 peptide:Eu^{3+} complex. For A and C: Magenta represents the observed 1:1 complex and green the Eu^{3+}-aquo ion (see Section 2.3 for more experimental details).

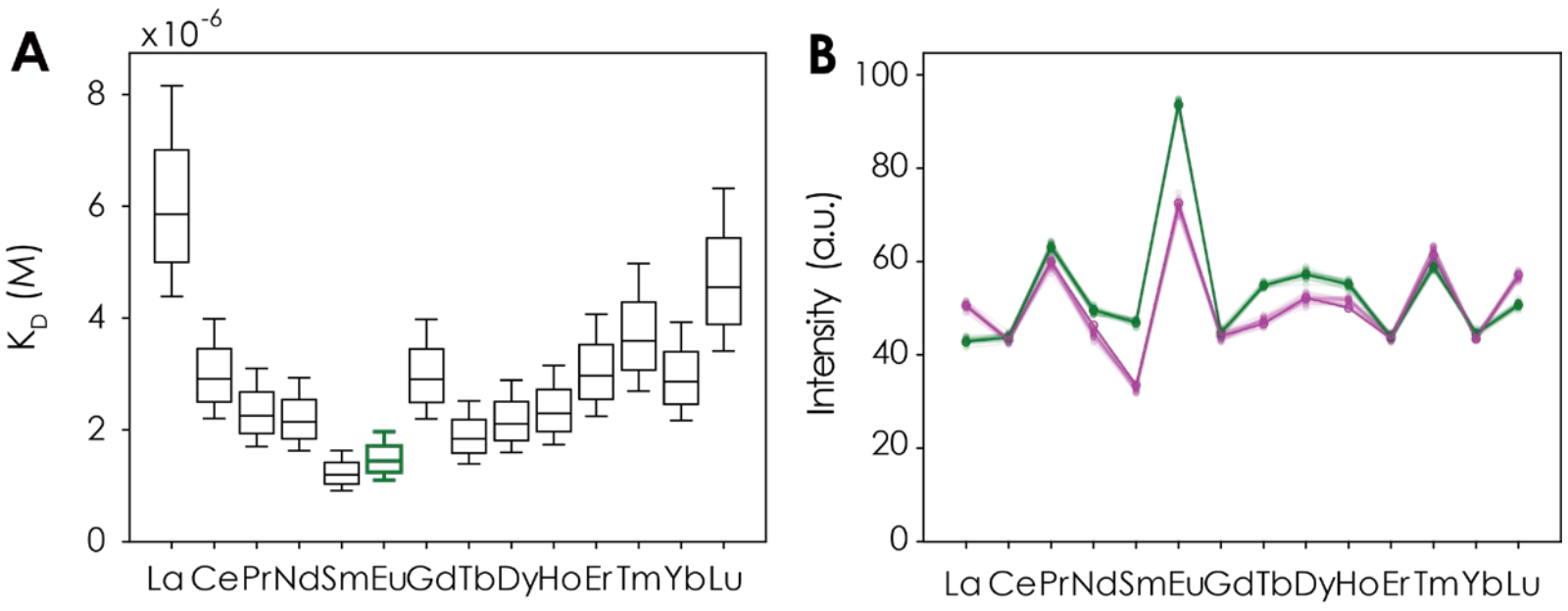

Figure S42 Lanthanide competition experiment between different Lns (except Pm) *vs.* Eu^{3+} with EF1-G-cyc. **(A)** Binding affinities shown as box plot (median and quartiles). **(B)** PARAFAC (solid line) and MC (transparent lines) distribution of the Ln-series for the 1:1 peptide:Eu^{3+} complex (magenta) and the free Eu^{3+}-aquo ion (green). See Section 2.3 for more experimental details.

4.3 Supplementary CD Data

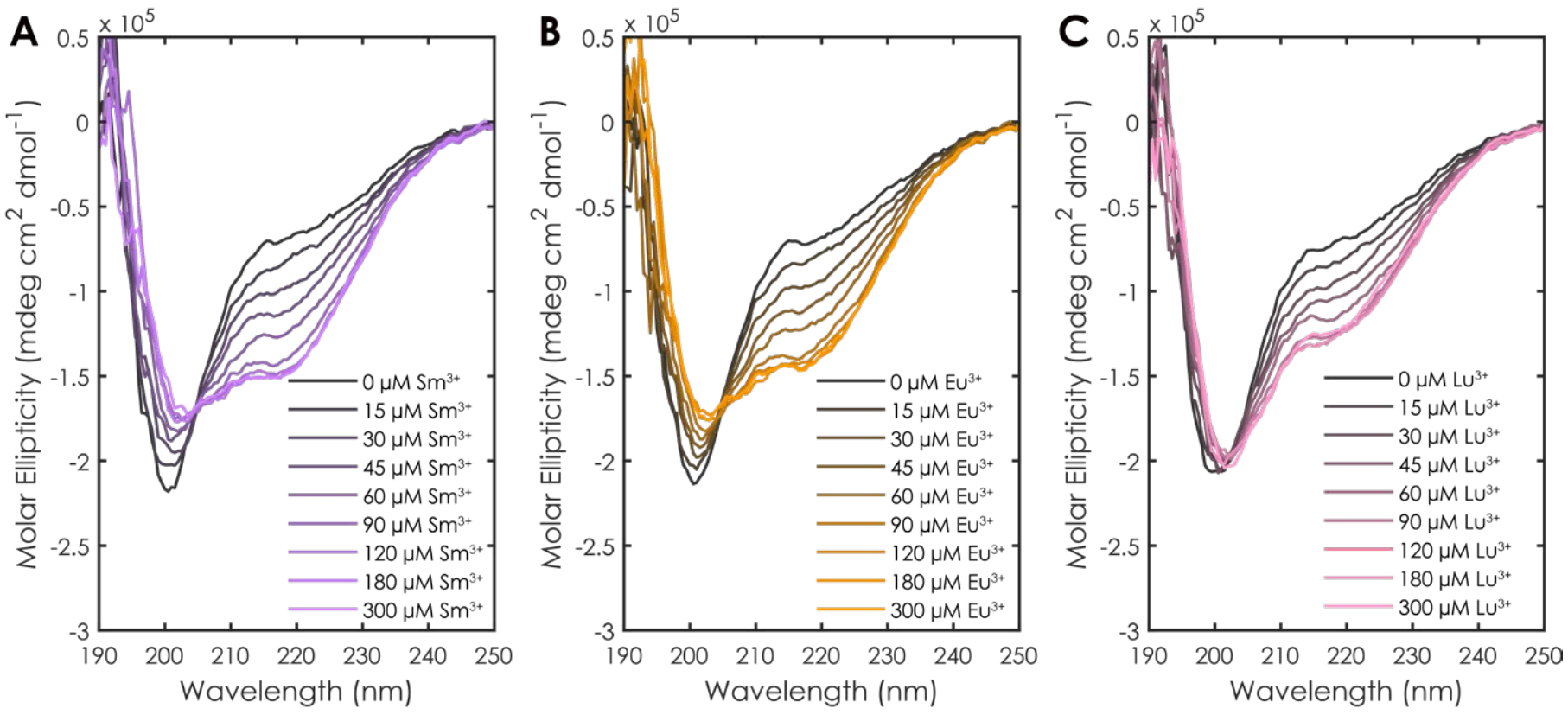

Figure S43 CD spectra of lanthanide chloride to EF1-cyc (60 µM) titration experiments at 25 °C and pH 6.6 (10 mM MOPSO, 100 mM KCl). **(A)** Sm^{3+} to EF1-cyc, **(B)** Eu^{3+} to EF1-cyc, **(C)** Lu^{3+} to EF1-cyc.

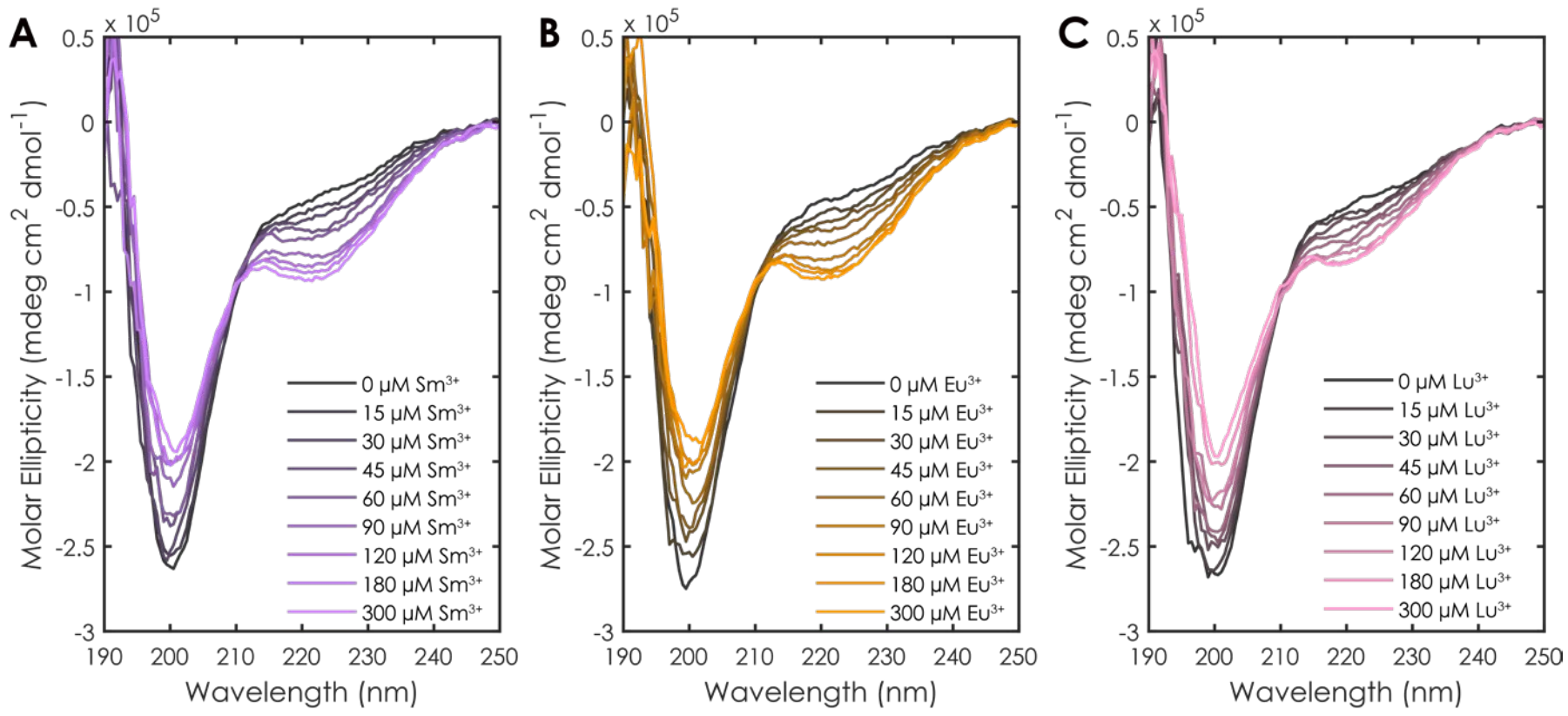

Figure S44 CD spectra of lanthanide chloride to EF1-R-cyc (60 µM) titration experiments at 25 °C and pH 6.6 (10 mM MOPSO, 100 mM KCl). **(A)** Sm^{3+} to EF1-R-cyc, **(B)** Eu^{3+} to EF1-R-cyc, **(C)** Lu^{3+} to EF1-R-cyc.

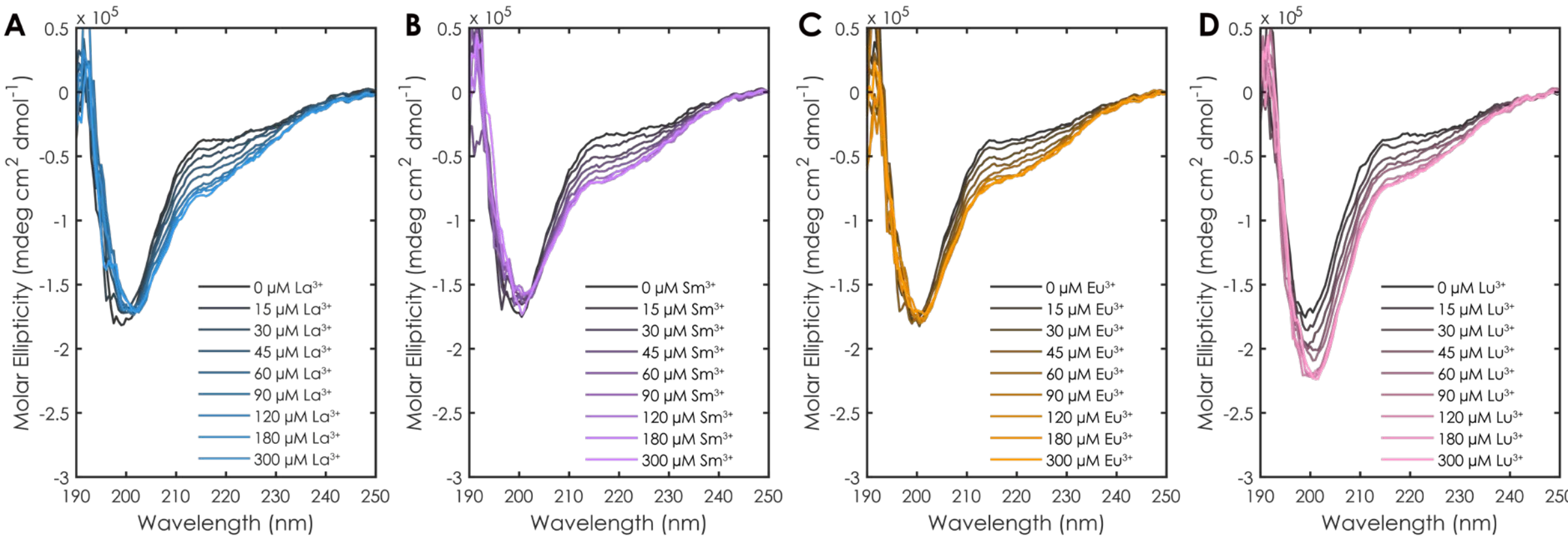

Figure S45 CD spectra of lanthanide chloride to EF1-G-cyc (60 μM) titration experiments at 25 °C and pH 6.6 (10 mM MOPSO, 100 mM KCl). **(A)** La^{3+} to EF1-G-cyc, **(B)** Sm^{3+} to EF1-G-cyc, **(C)** Eu^{3+} to EF1-G-cyc, **(D)** Lu^{3+} to EF1-G-cyc.

4.4 Supplementary NMR Data

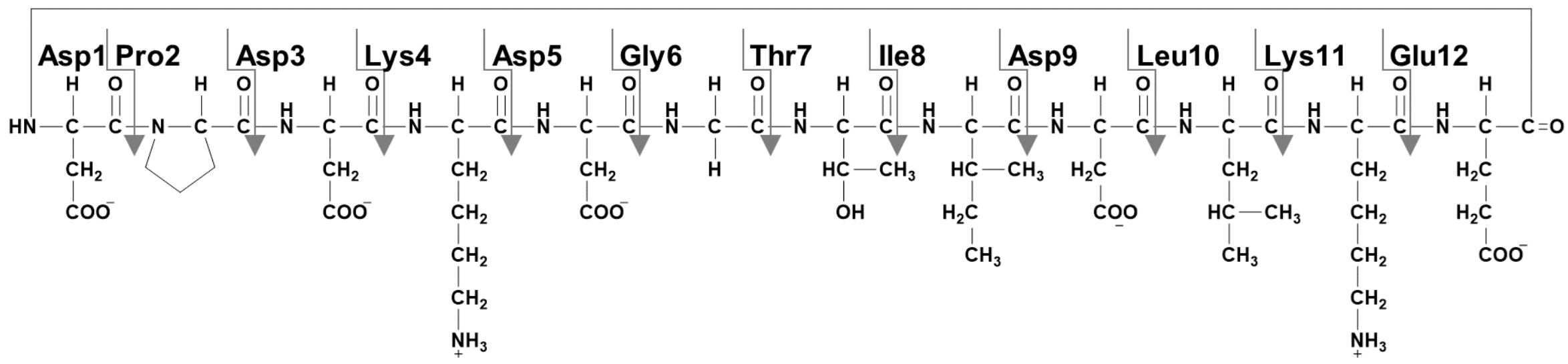

Figure S46 Structural view of the peptide EF1-cyc separated by spin system.

Table S8 ^{1}H NMR chemical shifts of EF1-cyc (600 MHz, H_2O/D_2O (9:1, *v/v*) + 0.003% TMSP-d_4, 30 mM MES-d_{13}, 100 mM KCl).

	NH	Hα	Hβ	Hγ	Hδ	Hε
**Asp1**	8.07	4.94	2.80, 2.52	—	—	—
**Pro2**	—	4.42	2.31, 1.99	2.01	3.83, 3.75	—
**Asp3**	8.41	4.67	2.78, 2.68	—	—	—
**Lys4**	7.85	4.31	1.87, 1.81	1.41	1.69	3.02
**Asp5**	8.39	4.64	2.72, 2.70	—	—	—
**Gly6**	8.45	4.09, 3.96	—	—	—	—
**Thr7**	8.14	4.38	4.28	1.21	—	—
**Ile8**	8.22	4.25	1.93	1.47, 1.19, 0.92	0.88	—
**Asp9**	8.37	4.59	2.75, 2.64	—	—	—
**Leu10**	8.12	4.30	1.70, 1.65	1.63	0.95, 0.90	—
**Lys11**	8.23	4.26	1.85	1.44	1.70	3.02
**Glu12**	8.30	4.23	2.08, 1.97	2.27, 2.23	—	—

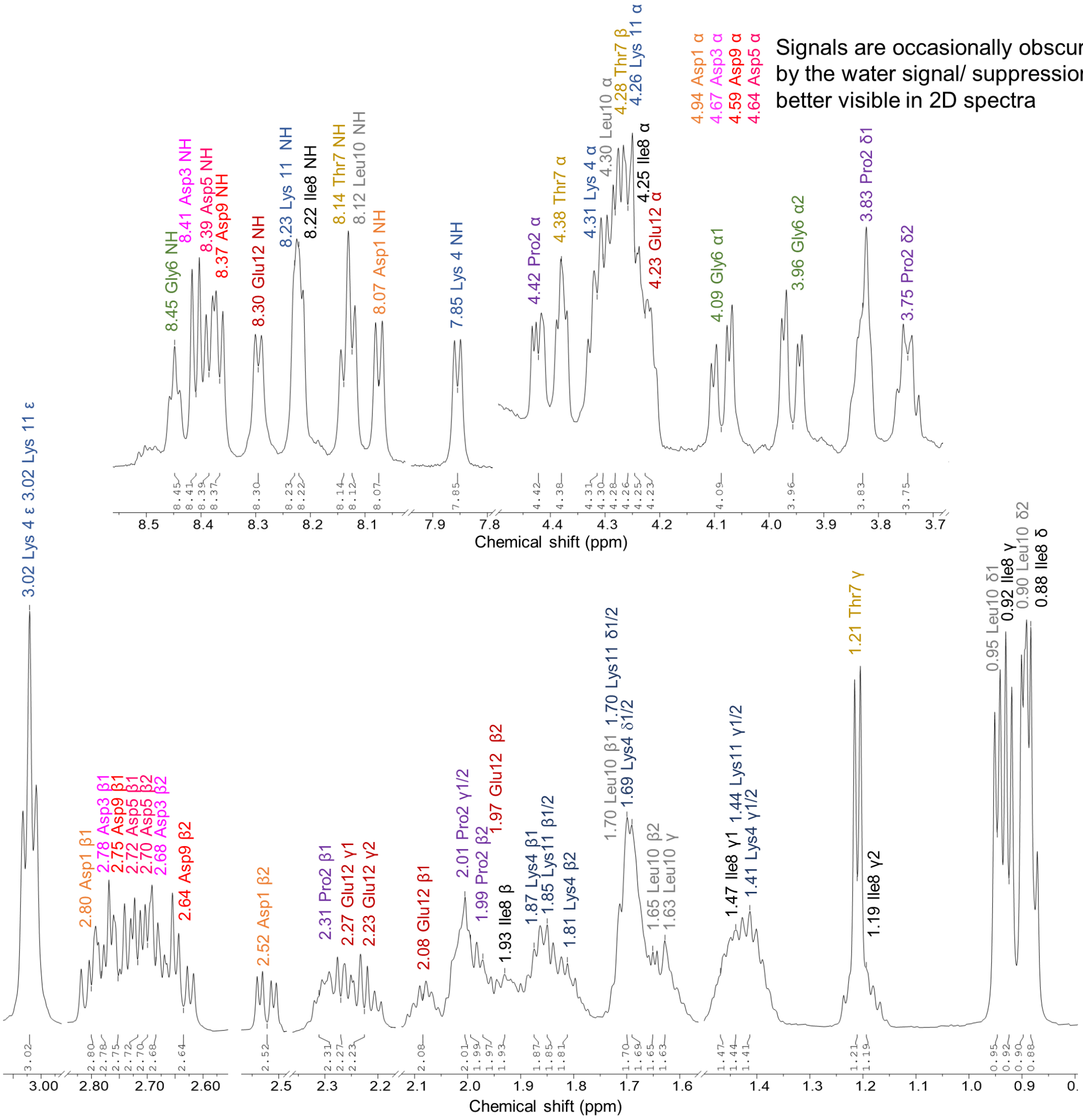

Figure S47 Overview of the assignment of the ^{1}H NMR signals of EF1-cyc, colour-coded by amino acid (600 MHz, H_2O/D_2O (9:1) + 0.003% TMSP-d_4, 30 mM MES-d_{13}, 100 mM KCl).

Table S9 ^{13}C NMR chemical shifts of EF1-cyc obtained from HSQC and HMBC 2D experiments (600 MHz, H_2O/D_2O (9:1, *v/v*) + 0.003% TMSP-d_4, 30 mM MES-d_{13}, 100 mM KCl).

	C=O	Cα	Cβ	Cγ	Cδ	Cε	COOH
**Asp1**	174.6	52.2	42.0	—	—	—	180.2
**Pro2**	177.4	64.2	32.4	27.3	51.0	—	—
**Asp3**	176.5	54.6	41.5	—	—	—	180.5
**Lys4**	176.4	56.5	33.1	24.7	29.2	42.2	—
**Asp5**	177.1	54.8	41.5	—	—	—	180.3
**Gly6**	174.7	45.7	—	—	—	—	—
**Thr7**	175.0	62.2	69.9	21.8	—	—	—
**Ile8**	176.0	61.6	39.1	27.3, 17.7	13.3	—	—
**Asp9**	176.2	54.6	41.5	—	—	—	180.2
**Leu10**	177.5	55.7	42.1	27.1	25.2, 23.6	—	—
**Lys11**	176.9	56.9	32.9	24.9	29.2	42.0	—
**Glu12**	176.0	57.0	30.1	36.5	—	—	184.0

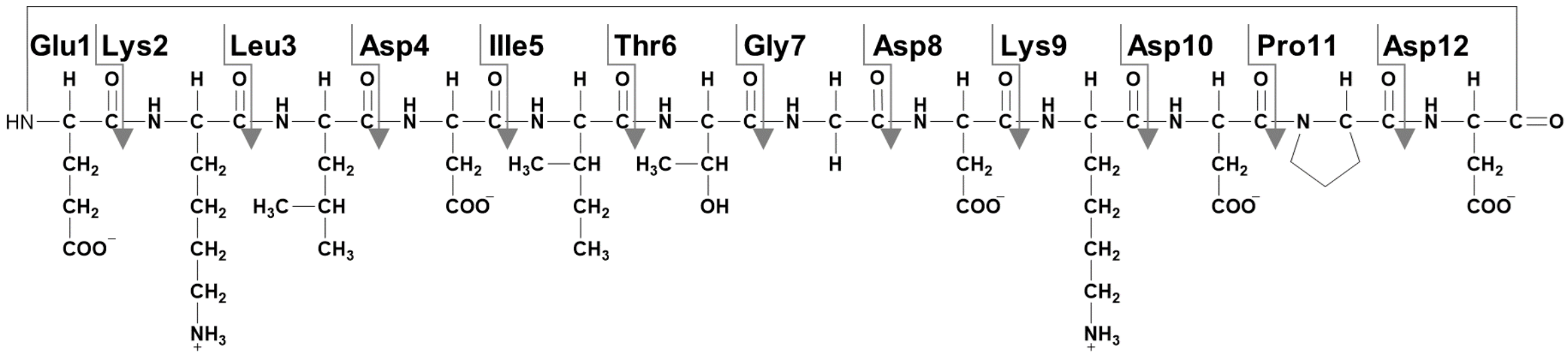

Figure S48 Structural view of the peptide EF1-R-cyc separated by spin system.

Table S10 ^{1}H NMR chemical shifts of EF1-R-cyc (600 MHz, H_2O/D_2O (9:1, *v/v*) + 0.003% TMSP-d_4, 30 mM MES-d_{13}, 100 mM KCl).

	NH	Hα	Hβ	Hγ	Hδ	Hε
**Glu1**	8.21	4.18	2.15, 2.09	2.30, 2.27	—	—
**Lys2**	8.36	4.30	1.85	1.46, 1.42	1.70	3.03
**Leu3**	8.28	4.40	1.70, 1.59	1.60	0.93, 0.87	—
**Asp4**	8.40	4.66	2.78, 2.67	—	—	—
**Ile5**	8.06	4.28	1.98	1.44, 1.21, 0.95	0.90	—
**Thr6**	8.26	4.38	4.30	1.24	—	—
**Gly7**	8.31	4.10, 3.91	—	—	—	—
**Asp8**	8.19	4.61	2.68, 2.61	—	—	—
**Lys9**	8.27	4.38	1.83, 1.77	1.42, 1.38	1.67	3.02
**Asp10**	8.37	4.68	2.78, 2.68	—	—	—
**Pro11**	—	4.40	2.32, 1.97	2.03	3.81, 3.77	—
**Asp12**	8.44	4.86	2.76, 2.67	—	—	—

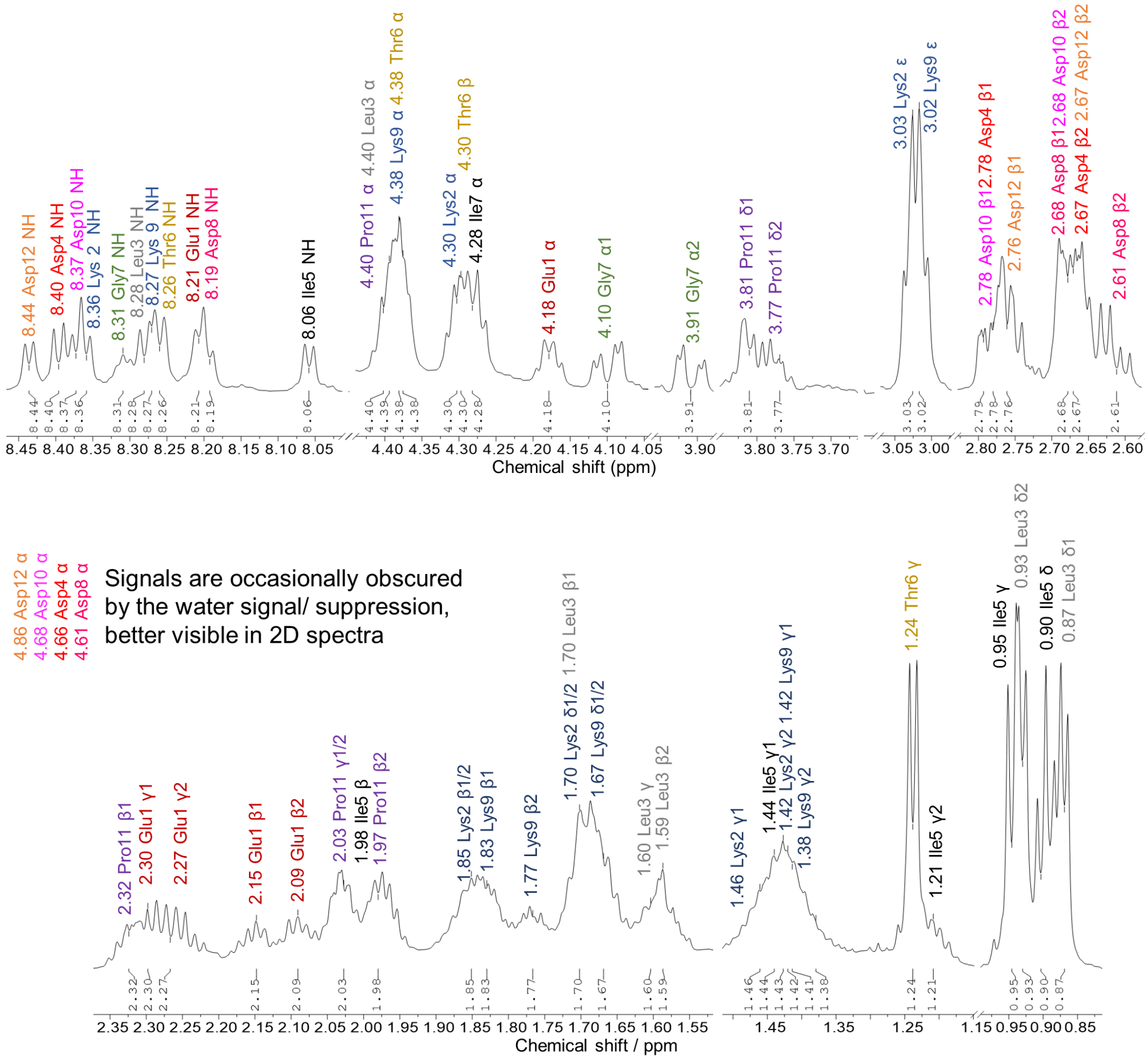

Figure S49 Overview and assignment of the ^{1}H NMR signals of EF1-R-cyc, colour-coded by amino acid (600 MHz, H_2O/D_2O (9:1, *v/v*) + 0.003% TMSP-d_4, 30 mM MES-d_{13}, 100 mM KCl).

Table S11 ^{13}C NMR chemical shifts of EF1-R-cyc obtained from HSQC and HMBC 2D experiments (600 MHz, H_2O/D_2O (9:1, *v/v*) + 0.003% TMSP-d_4, 30 mM MES-d_{13}, 100 mM KCl).

	C=O	Cα	Cβ	Cγ	Cδ	Cε	COOH
**Glu1**	176.5	57.3	29.6	36.5	—	—	183.9
**Lys2**	176.9	56.8	32.8	25.0	29.0	42.3	—
**Leu3**	177.2	55.4	42.8	27.2	25.2, 23.5	—	—
**Asp4**	176.6	54.6	41.4	—	—	—	180.4
**Ile5**	176.8	61.8	39.1	27.3, 17.9	13.5	—	—
**Thr6**	175.4	62.4	70.1	21.9	—	—	—
**Gly7**	174.0	45.7	—	—	—	—	—
**Asp8**	176.1	54.5	41.5	—	—	—	180.3
**Lys9**	177.0	56.2	33.4	24.7	29.2	42.3	—
**Asp10**	175.2	54.0	41.3	—	—	—	180.4
**Pro11**	176.2	64.5	32.3	27.5	51.1	—	—
**Asp12**	176.4	52.7	41.8	—	—	—	180.1

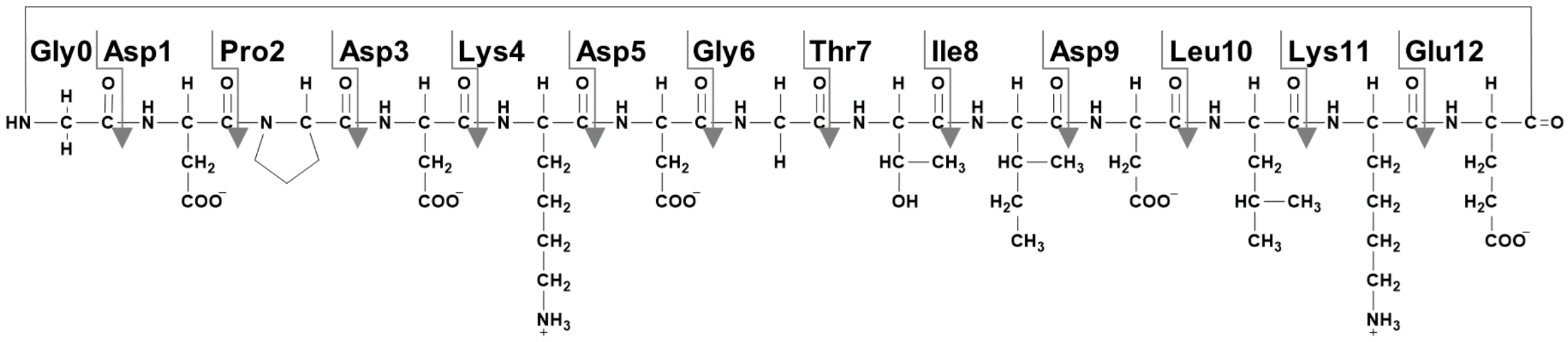

Figure S50 Structural view of the peptide EF1-G-cyc separated by spin system.

Table S12 ^{1}H NMR chemical shifts of EF1-G-cyc (600 MHz, H_2O/D_2O (9:1, *v/v*) + 0.003% TMSP-d_4, 30 mM MES-d_{13}, 100 mM KCl).

	NH	Hα	Hβ	Hγ	Hδ	Hε
**Gly0**	8.38	4.05, 3.86	—	—	—	—
**Asp1**	8.16	4.95	2.78, 2.57	—	—	—
**Pro2**	—	4.45	2.27, 1.98	2.03	3.87, 3.82	
**Asp3**	8.41	4.63	2.76, 2.70	—	—	—
**Lys4**	8.03	4.30	1.87, 1.80	1.42	1.69	3.02
**Asp5**	8.36	4.64	2.73, 2.72	—	—	—
**Gly6**	8.38	4.09, 3.94	—	—	—	—
**Thr7**	8.12	4.35	4.26	1.20	—	—
**Ile8**	8.18	4.20	1.89	1.47, 1.19, 0.90	0.87	—
**Asp9**	8.34	4.61	2.74, 2.61	—	—	—
**Leu10**	8.10	4.30	1.68, 1.63	1.63	0.94, 0.88	—
**Lys11**	8.44	4.29	1.86	1.45	1.70	3.03
**Glu12**	8.33	4.26	2.08, 1.98	2.25	—	—

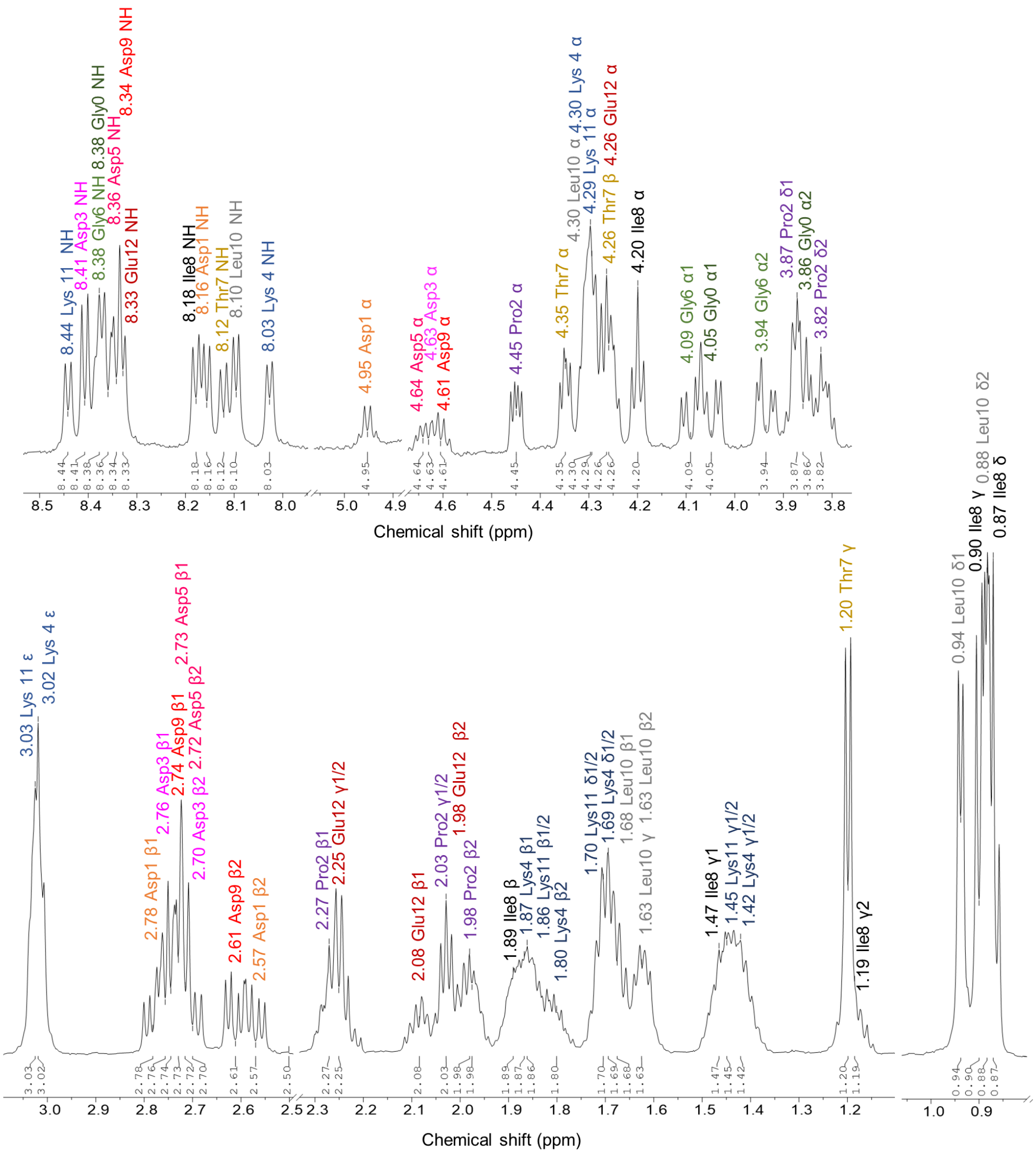

Figure S51 Overview and assignment of the ^{1}H NMR signals of EF1-G-cyc, colour-coded by amino acid **(**600 MHz, H_2O/D_2O (9:1, *v/v*) + 0.003% TMSP-d_4, 30 mM MES-d_{13}, 100 mM KCl).

Table S13 ^{13}C NMR chemical shifts of EF1-G-cyc obtained from HSQC and HMBC 2D experiments (600 MHz, H_2O/D_2O (9:1, *v/v*) + 0.003% TMSP-d_4, 30 mM MES-d_{13}, 100 mM KCl).

	**C=O**	**Cα**	**Cβ**	**Cγ**	**Cδ**	**Cε**	**COOH**
**Gly0**	173.6	45.4	—	—	—	—	—
**Asp1**	175.0	52.2	41.6	—	—	—	180.0
**Pro2**	177.2	63.8	32.3	27.3	50.9	—	—
**Asp3**	176.8	54.5	41.5	—	—	—	180.4
**Lys4**	176.6	56.9	33.2	24.7	29.2	42.4	—
**Asp5**	177.0	54.8	41.6	—	—	—	180.4
**Gly6**	174.8	45.8	—	—	—	—	—
**Thr7**	174.9	62.4	69.9	21.1	—	—	—
**Ile8**	175.9	61.6	39.2	27.5, 17.7	13.2	—	—
**Asp9**	176.1	54.4	41.3	—	—	—	180.3
**Leu10**	177.7	55.8	42.5	27.1	25.2, 23.7	—	—
**Lys11**	176.9	56.8	33.0	24.8	29.1	42.3	—
**Glu12**	177.0	57.1	30.1	36.3	—	—	183.9

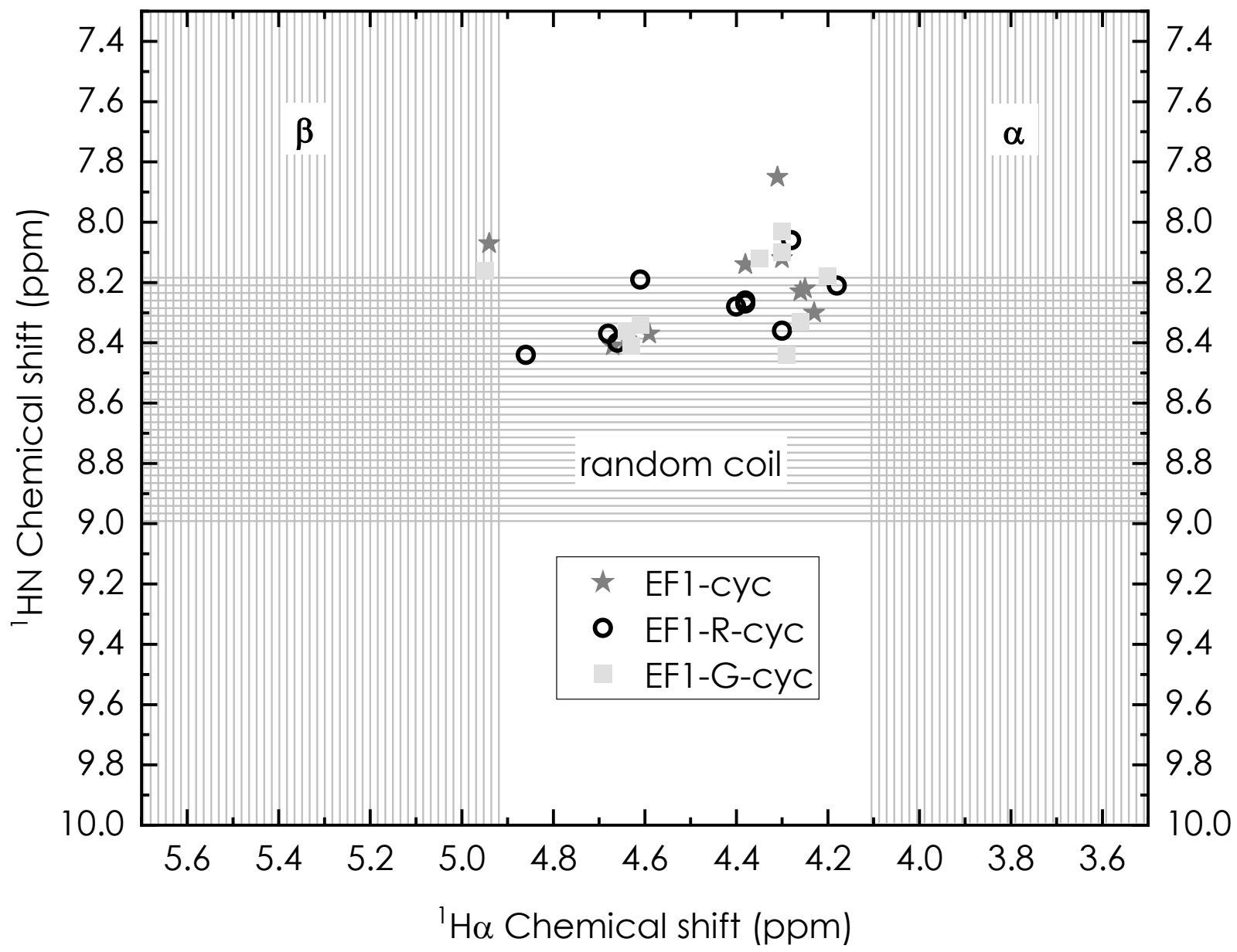

Figure S52 CD-by-NMR plot of the NH/Hα fingerprint ^{1}H NMR region according to Wishart *et al*.[18]

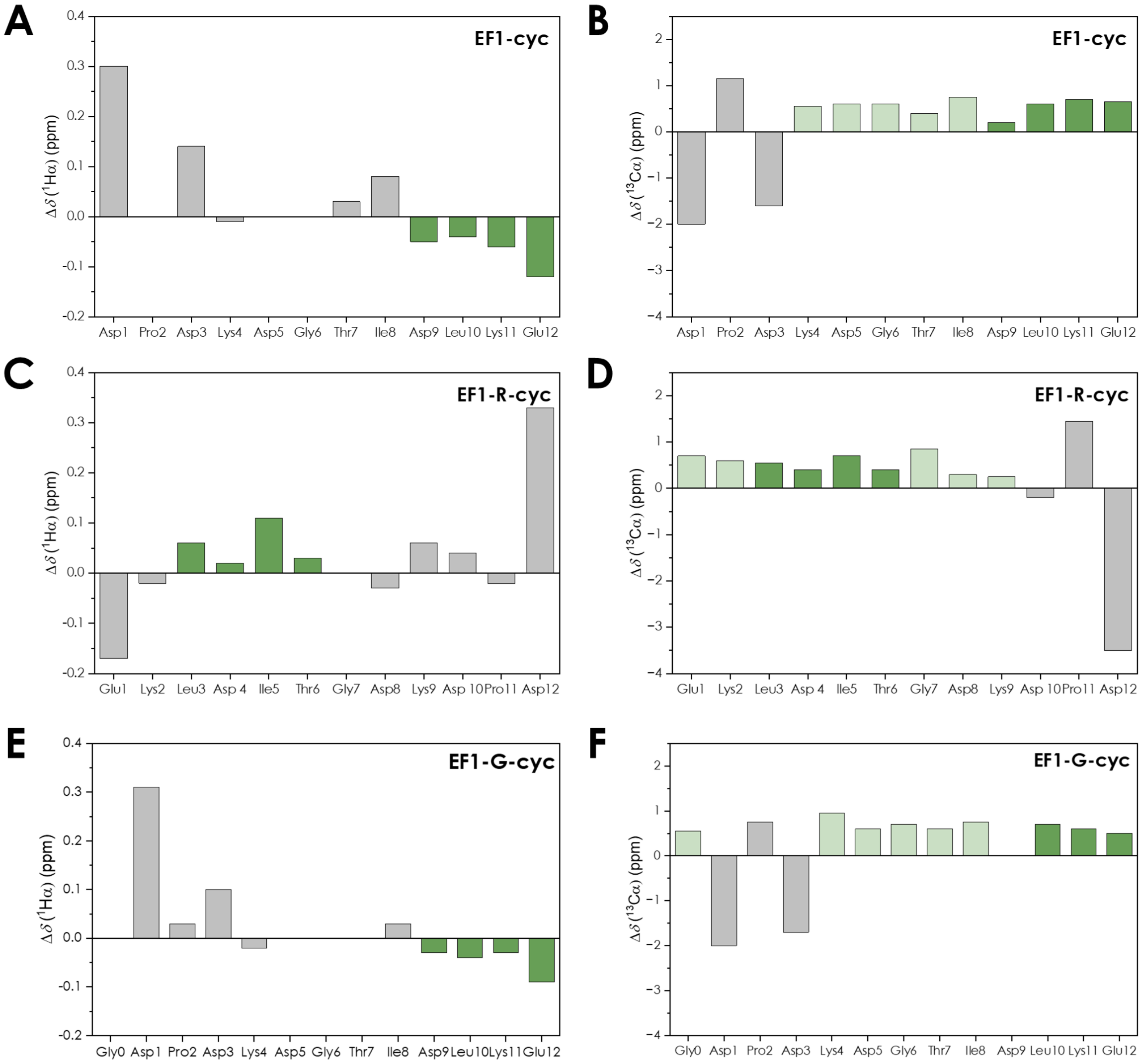

Figure S53 ^{1}Hα secondary chemical shifts (SCS) of **(A)** EF1-cyc, **(C)** EF1-R-cyc, and **(E)** EF1-G-cyc as well as ^{13}Cα SCS of **(B)** EF1-cyc, **(D)** EF1-R-cyc and **(F)** EF1-G-cyc. Both SCS were obtained by subtracting nearest neighbour effects-corrected random coil chemical shifts values[18] from the observed chemical shift values of the respective peptide (for ^{1}Hα Gly was ignored on purpose; see Tables S8-S12 for the observed Hα and Cα shifts of the respective peptide). In A, C, E a train of at least four consecutive amino acids showing the same inclination is coloured green. In B, D and F consecutive amino acids showing the same inclination are coloured light green, while amino acids indicating coincidence with consecutive ^{1}Hα SCS are shown in dark green.

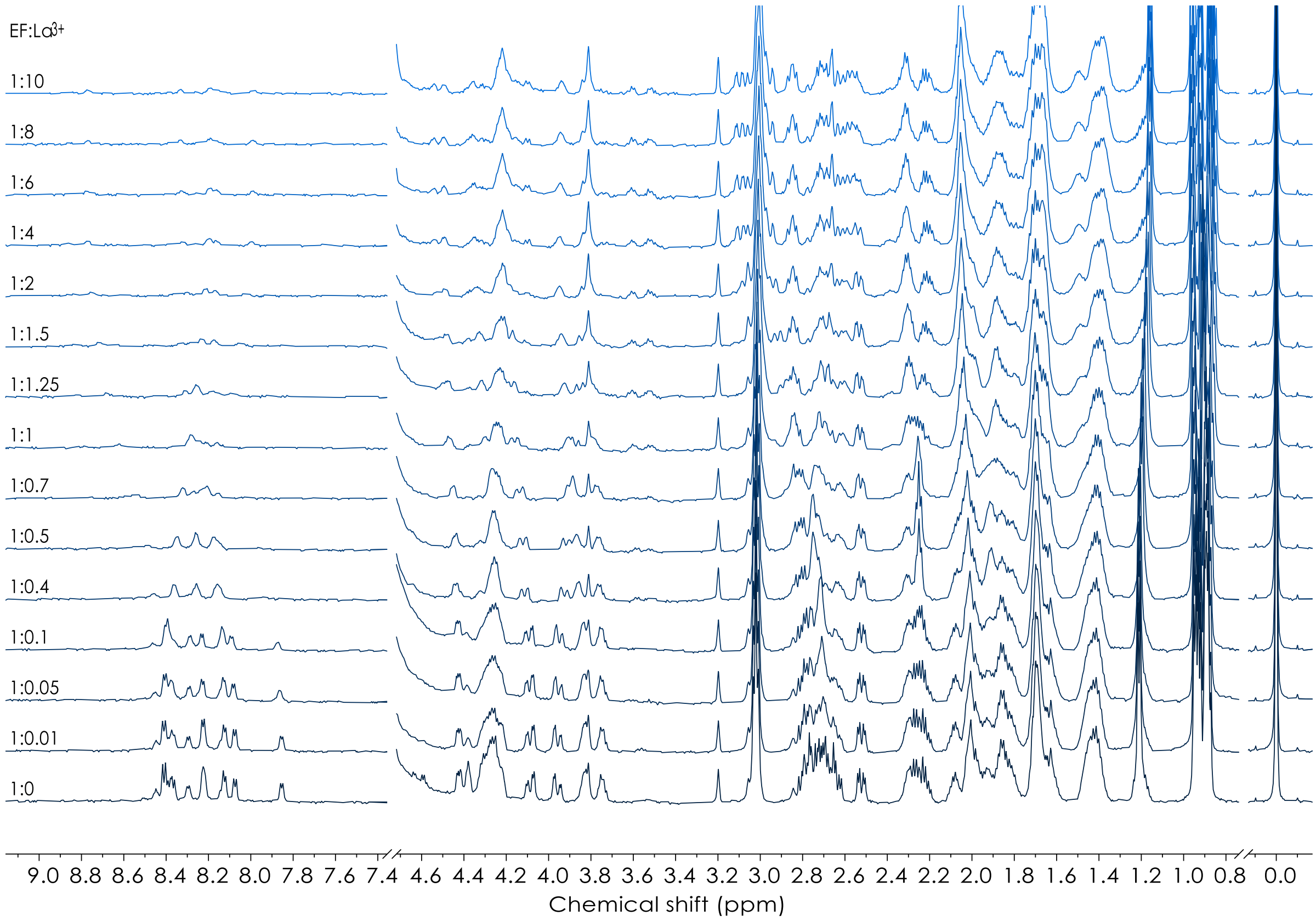

Figure S54 Stacked ^{1}H NMR spectra of the titration of $LaCl_3$ to EF1-cyc in increasing amounts (0 µM to 2 mM); the peptide and buffer concentration was kept constant (600 MHz, H_2O/D_2O (9:1, *v/v*) + 0.003% TMSP-d_4, 30 mM MES-d_{13}, 100 mM KCl). All spectra were area normalised to the TMSP-d_4 signal at 0 ppm.

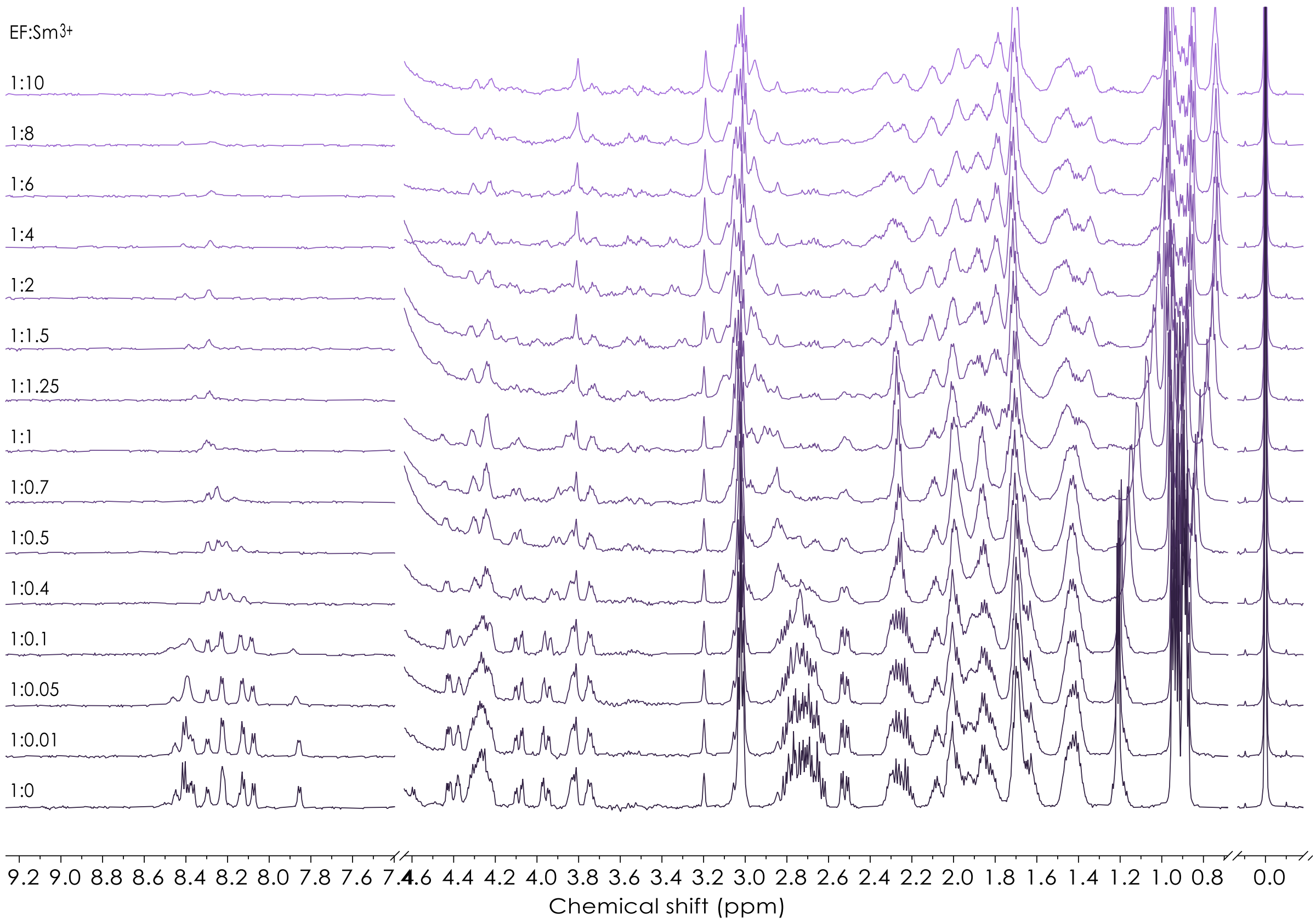

Figure S55 Stacked ^{1}H NMR spectra of the titration of $SmCl_3$ to EF1-cyc in increasing amounts (0 µM to 2 mM); the peptide and buffer concentration was kept constant (600 MHz, H_2O/D_2O (9:1, *v/v*) + 0.003% TMSP-d_4, 30 mM MES-d_{13}, 100 mM KCl). All spectra were area normalised to the TMSP-d_4 signal at 0 ppm.

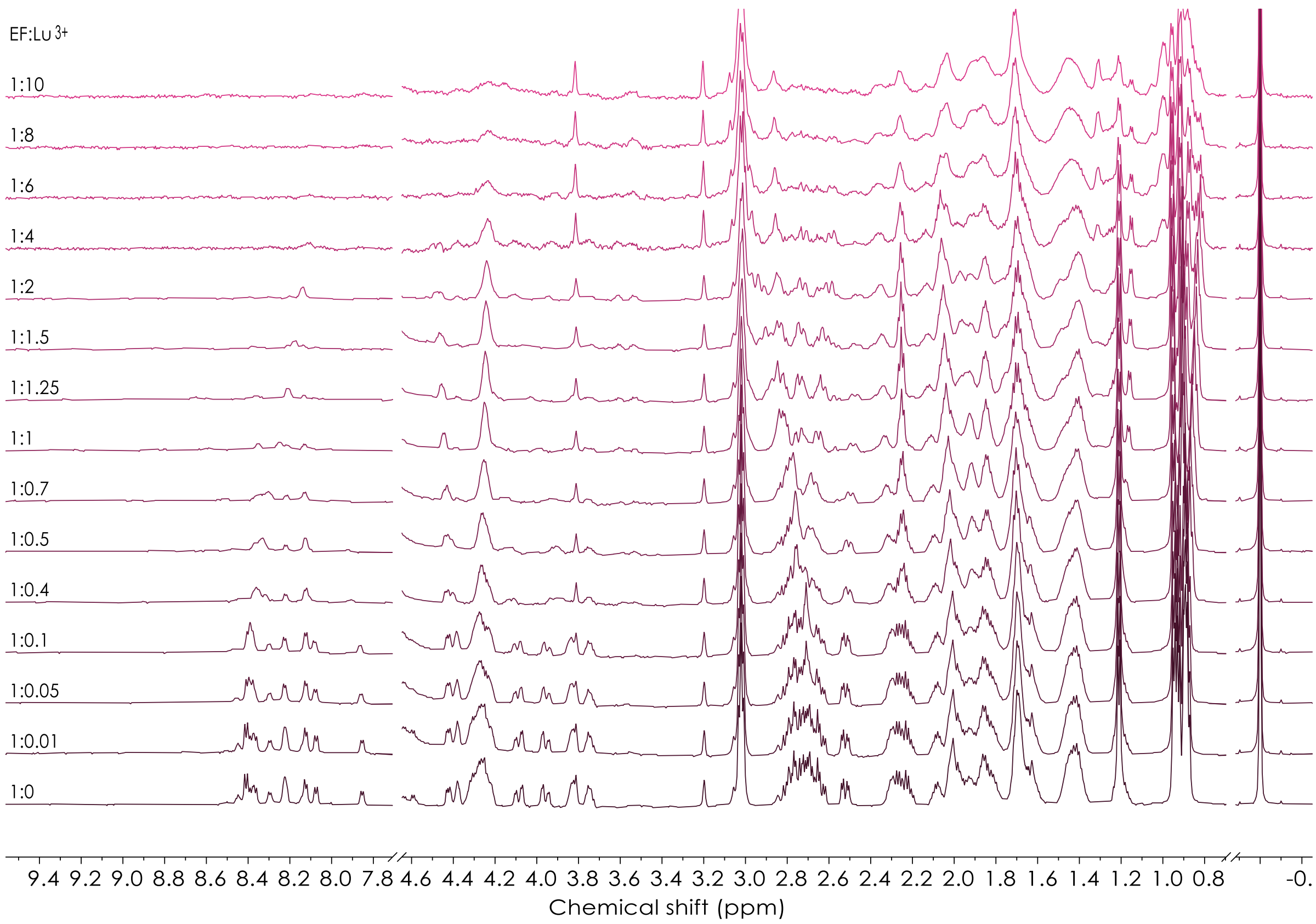

Figure S56 Stacked ^{1}H NMR spectra of the titration of $LuCl_3$ to EF1-cyc in increasing amounts (0 µM to 2 mM); the peptide and buffer concentration was kept constant (600 MHz, H_2O/D_2O (9:1, *v/v*) + 0.003% TMSP-d_4, 30 mM MES-d_{13}, 100 mM KCl). All spectra were area normalised to the TMSP-d_4 signal at 0 ppm.

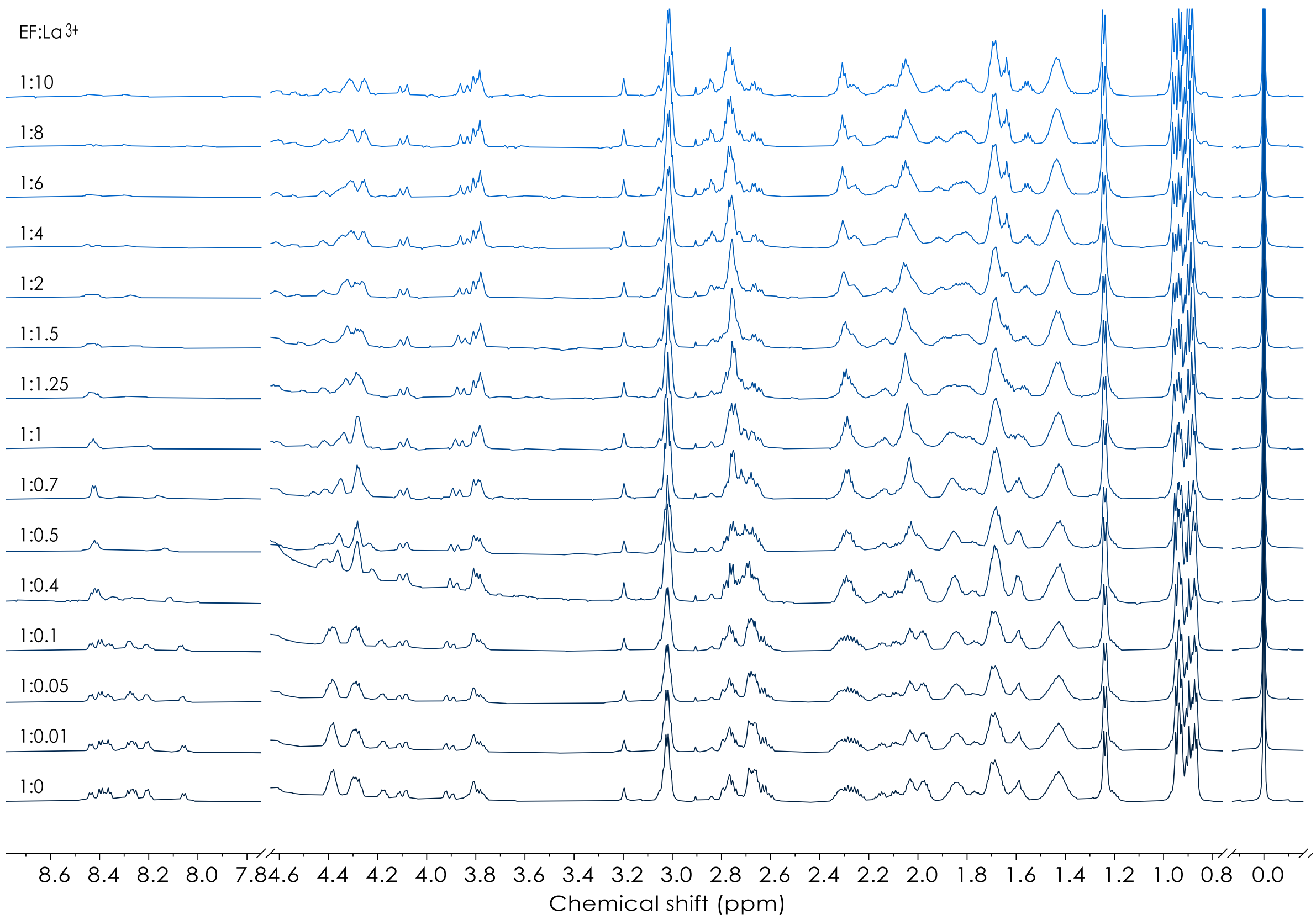

Figure S57 Stacked ^{1}H NMR spectra of the titration of $LaCl_3$ to EF1-R-cyc in increasing amounts (0 µM to 2 mM); the peptide and buffer concentration was kept constant (600 MHz, H_2O/D_2O (9:1, *v/v*) + 0.003% TMSP-d_4, 30 mM MES-d_{13}, 100 mM KCl). All spectra were area normalised to the TMSP-d_4 signal at 0 ppm.

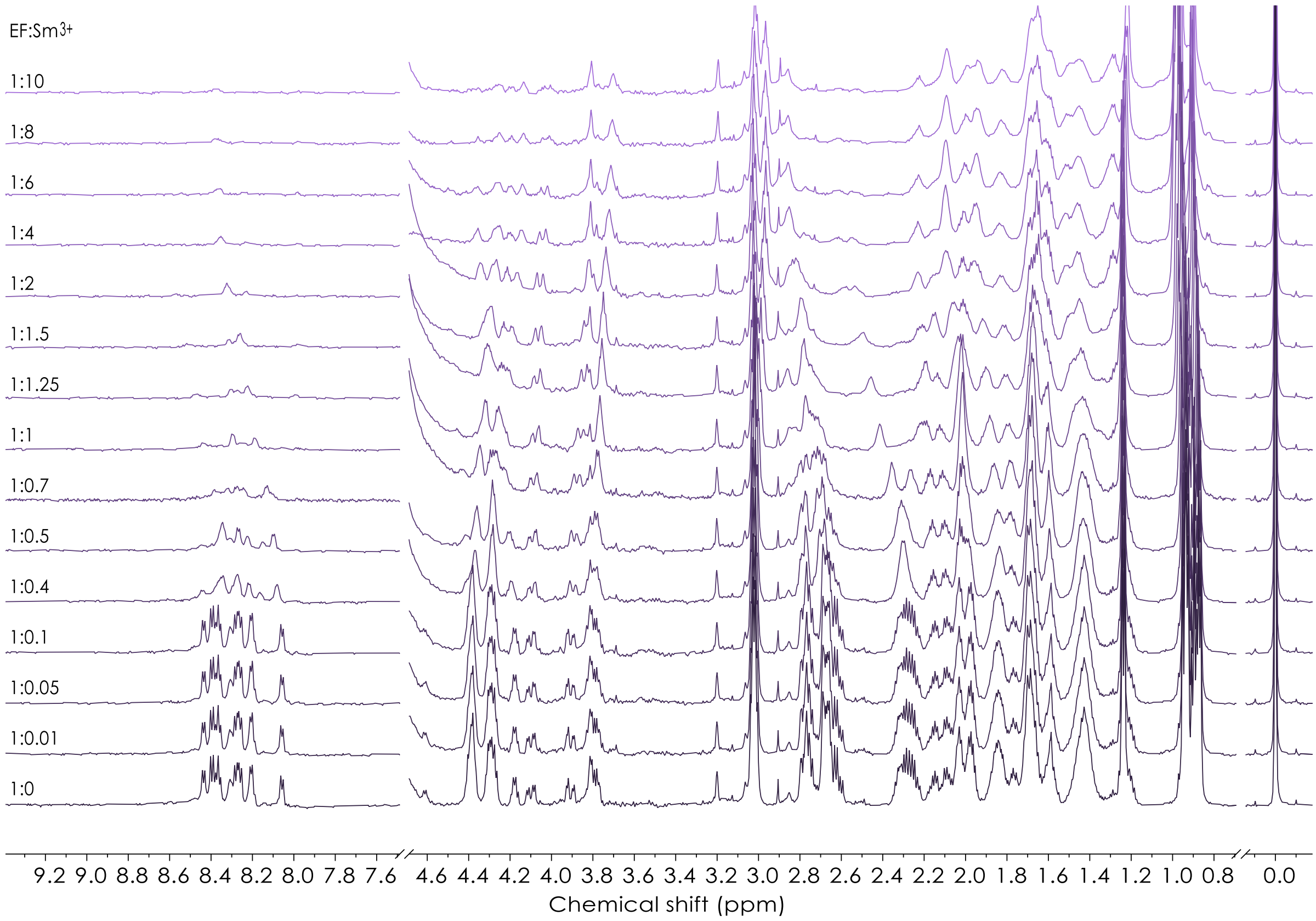

Figure S58 Stacked ^{1}H NMR spectra of the titration of $SmCl_3$ to EF1-R-cyc in increasing amounts (0 µM to 2 mM); the peptide and buffer concentration was kept constant (600 MHz, H_2O/D_2O (9:1, *v/v*) + 0.003% TMSP-d_4, 30 mM MES-d_{13}, 100 mM KCl). All spectra were area normalised to the TMSP-d_4 signal at 0 ppm.

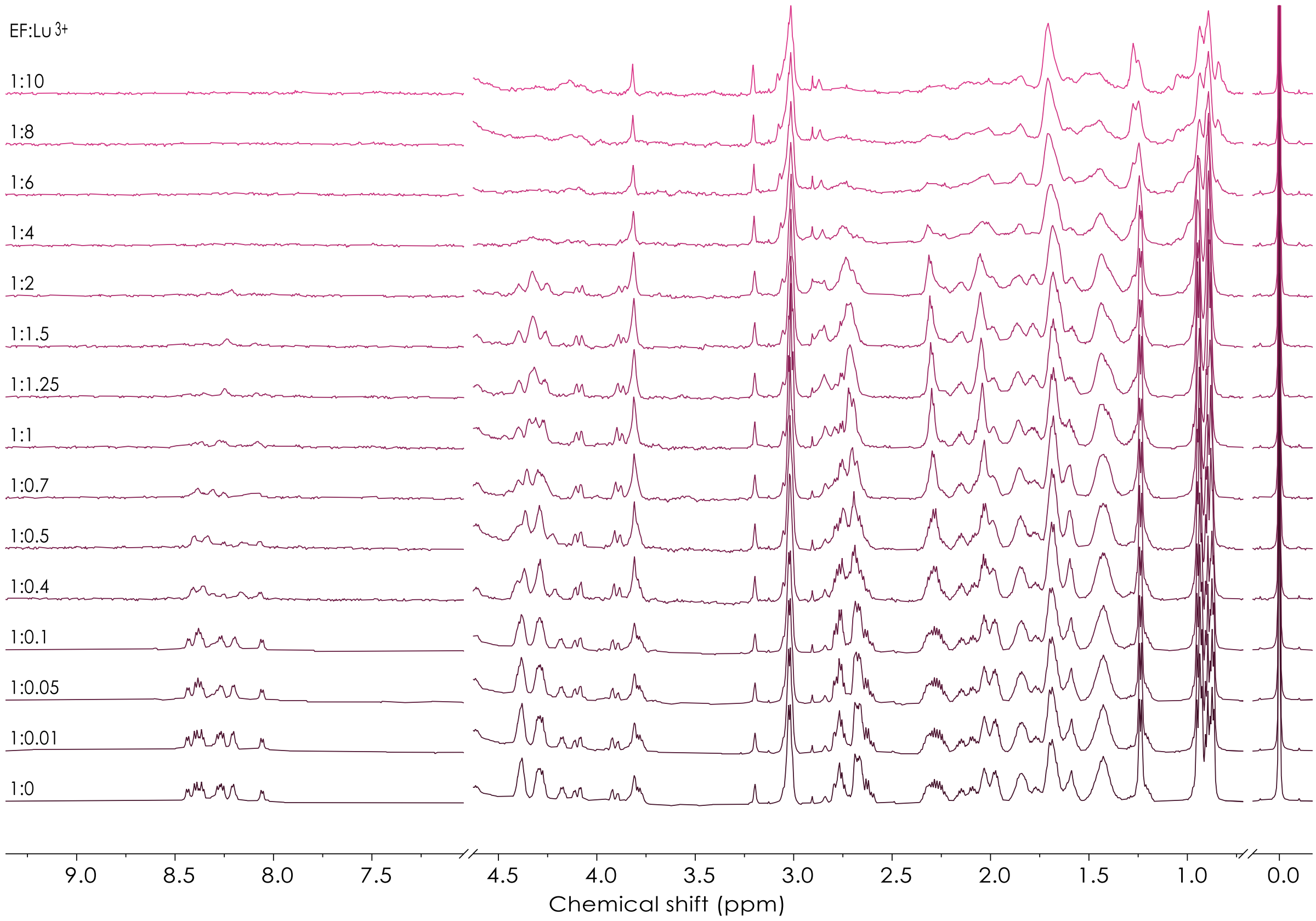

Figure S59 Stacked ^{1}H NMR spectra of the titration of $LuCl_3$ to EF1-R-cyc in increasing amounts (0 µM to 2 mM); the peptide and buffer concentration was kept constant (600 MHz, H_2O/D_2O (9:1, *v/v*) + 0.003% TMSP-d_4, 30 mM MES-d_{13}, 100 mM KCl). All spectra were area normalised to the TMSP-d_4 signal at 0 ppm.

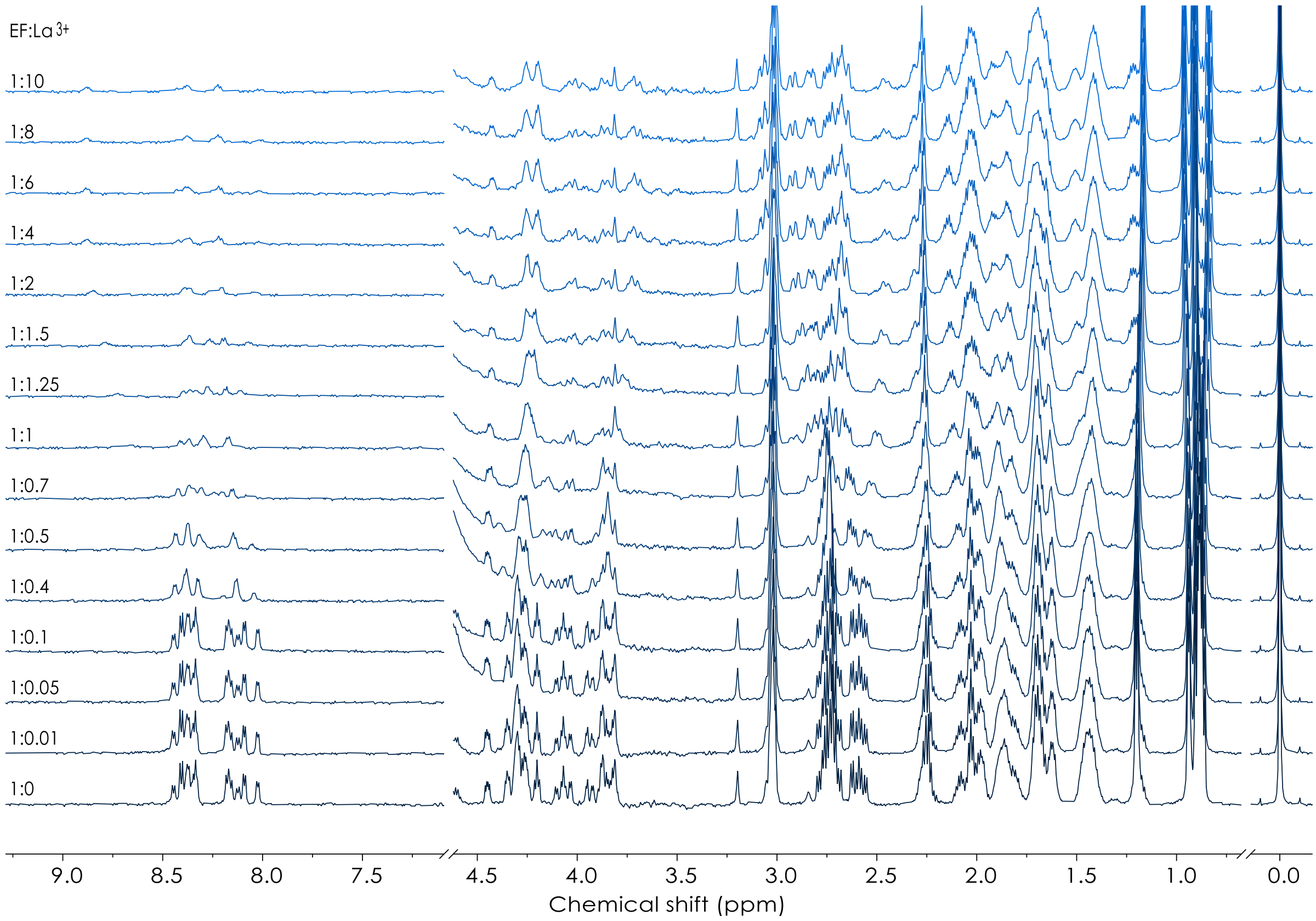

Figure S60 Stacked ^{1}H NMR spectra of the titration of $LaCl_3$ to EF1-G-cyc in increasing amounts (0 µM to 2 mM); the peptide and buffer concentration was kept constant (600 MHz, H_2O/D_2O (9:1, *v/v*) + 0.003% TMSP-d_4, 30 mM MES-d_{13}, 100 mM KCl). All spectra were area normalised to the TMSP-d_4 signal at 0 ppm.

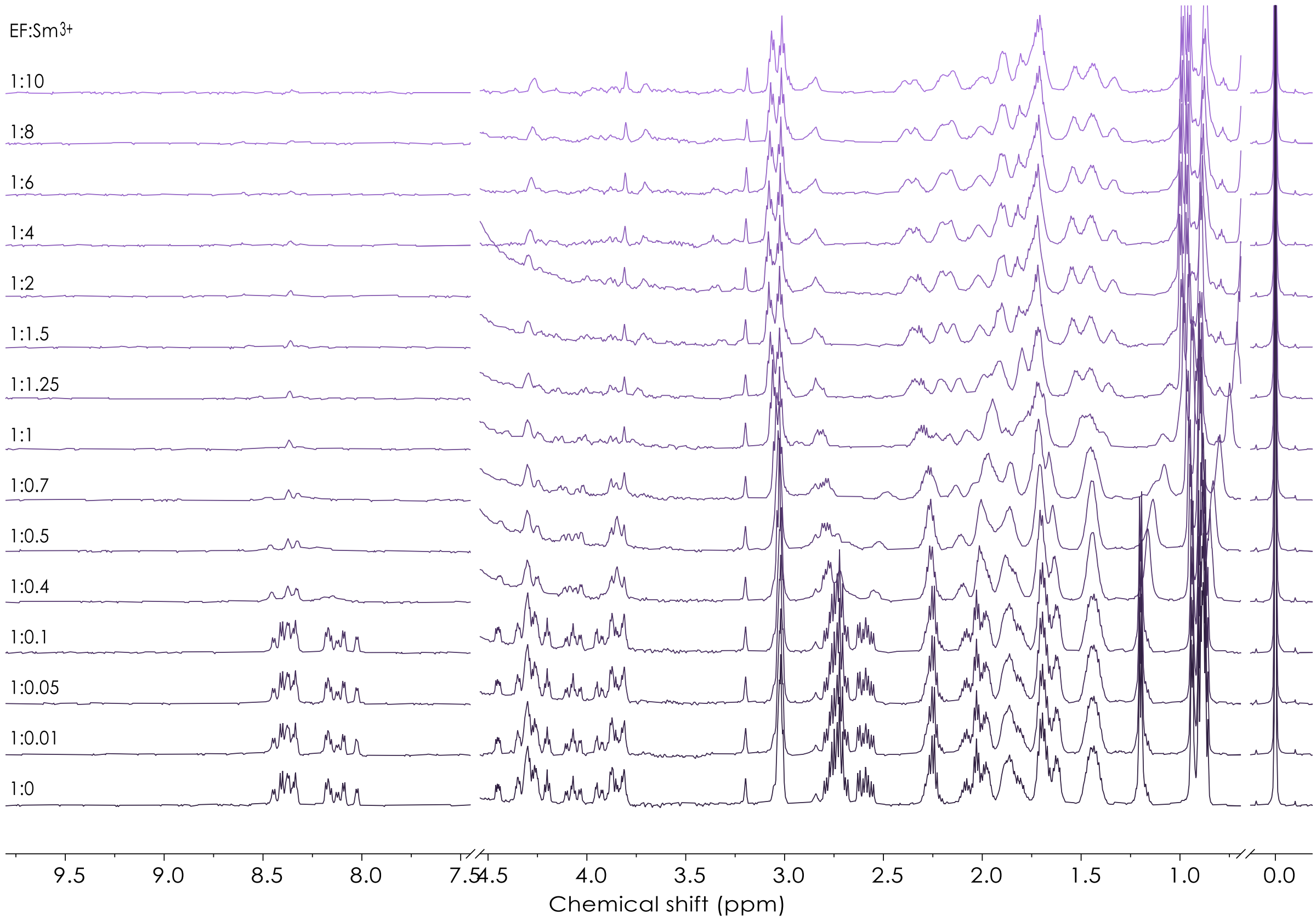

Figure S61 Stacked ^{1}H NMR spectra of the titration of $SmCl_3$ to EF1-G-cyc in increasing amounts (0 µM to 2 mM); the peptide and buffer concentration was kept constant (600 MHz, H_2O/D_2O (9:1, *v/v*) + 0.003% TMSP-d_4, 30 mM MES-d_{13}, 100 mM KCl). All spectra were area normalised to the TMSP-d_4 signal at 0 ppm.

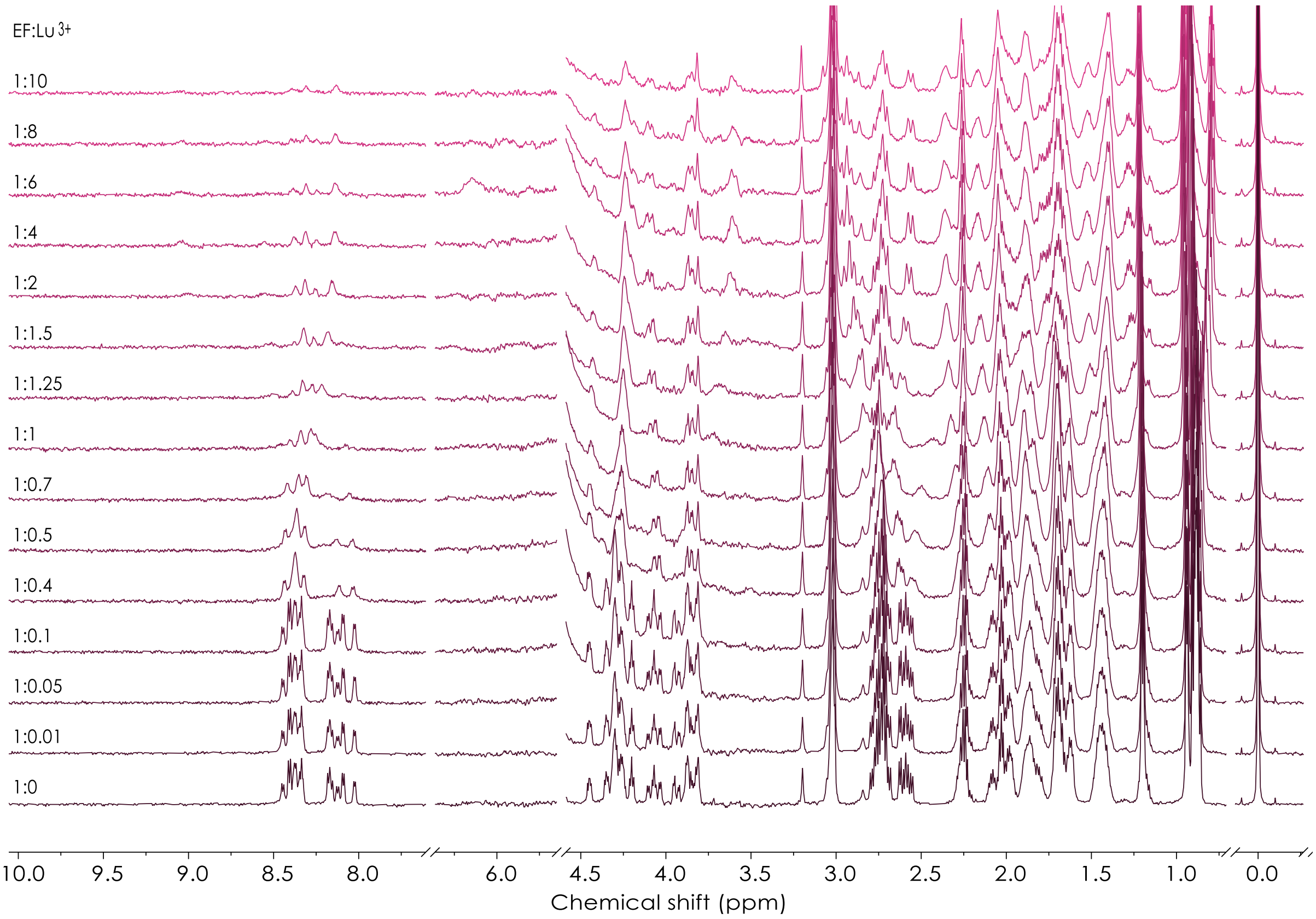

Figure S62 Stacked ^{1}H NMR spectra of the titration of $LuCl_3$ to EF1-G-cyc in increasing amounts (0 µM to 2 mM); the peptide and buffer concentration was kept constant (600 MHz, H_2O/D_2O (9:1, *v/v*) + 0.003% TMSP-d_4, 30 mM MES-d_{13}, 100 mM KCl). All spectra were area normalised to the TMSP-d_4 signal at 0 ppm.

Table S14 Chemical shifts (in ppm) of Asp and Glu signals observed for the 1:1 EF1-cyc to La^{3+} complex after metal-saturation determined with a combination of the ^{1}H spectra of the titration series and a TOCSY spectrum. Metal-induced shifts (in parentheses) relative to the metal-free peptide are given in parts per billion (ppb).

	NH	Hα	Hβ1	Hβ2	Hγ1	Hγ2
**Asp1**	8.38<br>(+300 ppb)	5.07<br>(+130 ppb)	2.84<br>(+40 ppb)	2.55<br>(+30 ppb)	-	-
**Asp3**	7.99<br>(−420 ppb)	4.68<br>(+10 ppb)	3.10<br>(+320 ppb)	2.65<br>(−30 ppb)	-	-
**Asp5**	8.19<br>(−200 ppb)	4.72<br>(+80 ppb)	2.95<br>(+230 ppb)	2.67<br>(−30 ppb)	-	-
**Asp9**	8.77<br>(+400 ppb)	4.74<br>(+150 ppb)	2.72<br>(−30 ppb)	2.59<br>(−50 ppb)	-	-
**Glu12**	8.15<br>(−150 ppb)	4.36<br>(+130 ppb)	2.04<br>(−40 ppb)	2.04<br>(+70 ppb)	2.26<br>(+40 ppb)	2.26<br>(−10 ppb)

Table S15 Chemical shifts (in ppm) of Asp and Glu signals observed for the 1:1 EF1-cyc to Sm^{3+} complex after metal-saturation determined with a combination of the ^{1}H spectra of the titration series and a TOCSY spectrum. Complexation-induced shifts (in parentheses) relative to the metal-free peptide are given in parts per billion (ppb). For some Asp signal sets it was not possible to unambiguously determine the respective Asp within the sequence. In such cases all possible Asp are given and the one used for calculating the shift in respective to the peptide only sample is underlined. The number of involved Asp residues in binding is not affected, as always the same number of involved Asp residues can be determined, even if the signals sets get permutated for calculating the shifts.

	NH	Hα	Hβ1	Hβ2	Hγ1	Hγ2
**Asp1**	8.15<br>(+70 ppb)	*n.d.*	2.67<br>(−130 ppb)	2.52<br>(0 ppb)	-	-
**Asp<u>3</u>/5/9**	*n.d.*	*n.d.*	*n.d.*	*n.d.*	-	-
**Asp3/<u>5</u>/9**	*n.d*	5.06<br>(+420 ppb)	3.19<br>(+470 ppb)	3.19<br>(+490 ppb)	-	-
**Asp3/5/<u>9</u>**	*n.d*	4.95<br>(+360 ppb)	3.34<br>(+590 ppb)	2.97<br>(+330 ppb)	-	-
**Glu12**	8.30<br>(0 ppb)	4.26<br>(+30 ppb)	2.11<br>(+30 ppb)	2.03<br>(+60 ppb)	2.28<br>(+10 ppb)	2.28<br>(−50 ppb)

Table S16 Chemical shifts (in ppm) of Asp and Glu signals observed for the 1:1 EF1-cyc to Lu^{3+} complex after metal-saturation determined with a combination of the ^{1}H spectra of the titration series and a TOCSY spectrum. Metal-induced shifts (in grey and in parentheses) relative to the metal-free peptide are given in parts per billion (ppb).

	NH	Hα	Hβ1	Hβ2	Hγ1	Hγ2
**Asp1**	8.24<br>(+160 ppb)	*n.d.*	2.84<br>(+40 ppb)	2.47<br>(−50 ppb)	-	-
**Asp3**	8.33<br>(−80 ppb)	4.72<br>(+50 ppb)	2.93<br>(+150 ppb)	2.61<br>(−70 ppb)	-	-
**Asp5**	8.14<br>(−250 ppb)	4.66<br>(+20 ppb)	2.98<br>(+260 ppb)	2.74<br>(+40 ppb)	-	-
**Asp9**	8.74<br>(+370 ppb)	*n.d.*	2.87<br>(+120 ppb)	2.78<br>(+140 ppb)	-	-
**Glu12**	8.42<br>(+120 ppb)	4.26<br>(+30 ppb)	2.14<br>(+60 ppb)	2.04<br>(+70 ppb)	2.26<br>(−10 ppb)	2.26<br>(+30 ppb)

Table S17 Chemical shifts (in ppm) of Asp and Glu signals observed for the EF1-R-cyc to La^{3+} 1:1 complex after metal-saturation determined with a combination of the ^{1}H spectra of the titration series and a TOCSY spectrum. Metal-induced signal shifts (in parentheses) are expressed in parts per billion (ppb). For the Asp signal sets it was not possible to unambiguously determine the respective Asp within the sequence. In such cases, all possible Asp spin systems are listed. Asp β resonances of Asp spin systems most likely involved in binding are highlighted in bold and underlined.

	NH	Hα	Hβ1	Hβ2	Hγ1	Hγ2
**Glu1**	8.23<br>(+20 ppb)	4.38<br>(+200 ppb)	2.13<br>(−20 ppb)	2.05<br>(−40 ppb)	2.31<br>(+10 ppb)	2.31<br>(+40 ppb)
**Asp4/8/10/12**	8.44	4.61	<u>**2.82**</u>	<u>**2.78**</u>	-	-
**Asp4/8/10/12**	*n.d.*	4.62	<u>**2.86**</u>	<u>**2.74**</u>	-	-
**Asp4/8/10/12**	8.41	5.01	2.77	2.77	-	-
**Asp4/8/10/12**	8.45	4.70	2.77	2.66	-	-

Table S18 Chemical shifts (in ppm) of Asp and Glu signals observed for the 1:1 EF1-R-cyc to Sm^{3+} complex after metal-saturation determined with a combination of the ^{1}H spectra of the titration series and a TOCSY spectrum. Metal-induced signal shifts (in parentheses) are expressed in parts per billion (ppb). For the Asp signal sets it was not possible to unambiguously determine the respective Asp within the sequence. In such cases, all possible Asp spin systems are listed. Asp β resonances of Asp spin systems most likely involved in binding are highlighted in bold and underlined.

	NH	Hα	Hβ1	Hβ2	Hγ1	Hγ2
**Glu1**	8.31	4.34	2.23	2.16	2.56	2.53
	(+100 ppb)	(+160 ppb)	(+80 ppb)	(+70 ppb)	(+260 ppb)	(+260 ppb)
**Asp4/8/10/12**	*n.d.*	*n.d.*	***n.d.***	***n.d.***	-	-
**Asp4/8/10/12**	*n.d.*	5.04	**2.94**	**2.94**	-	-
**Asp4/8/10/12**	*n.d.*	4.58	**2.84**	**2.79**	-	-
**Asp4/8/10/12**	7.91	4.48	**2.82**	**2.82**		

Table S19 Chemical shifts (in ppm) of Asp and Glu signals observed for the 1:1 EF1-R-cyc to Lu^{3+} complex after metal-saturation determined with a combination of the ^{1}H spectra of the titration series and a TOCSY spectrum. Metal-induced signal shifts (in parentheses) are expressed in parts per billion (ppb). For the Asp signal sets it was not possible to unambiguously determine the respective Asp within the sequence. In such cases, all possible Asp spin systems are listed. Asp β resonances of Asp spin systems most likely involved in binding are highlighted in bold and underlined.

	NH	Hα	Hβ1	Hβ2	Hγ1	Hγ2
**Glu1**	*n.d.*	4.33	2.15	2.05	2.31	2.15
		(+150 ppb)	(0 ppb)	(−40 ppb)	(+14 ppb)	(−120 ppb)
**Asp4/8/10/12**	8.22	4.70	**2.89**	**2.68**	-	-
**Asp4/8/10/12**	8.33	4.92	**2.90**	**2.75**	-	-
**Asp4/8/10/12**	8.49	4.60	2.75	2.71	-	-
**Asp4/8/10/12**	8.00	4.68	2.76	2.72	-	-

Table S20 Chemical shifts (in ppm) of Asp and Glu signals observed for the 1:1 EF1-G-cyc to La^{3+} complex after metal-saturation determined with a combination of the ^{1}H spectra of the titration series and a TOCSY spectrum. Metal-induced signal shifts (in parentheses) are expressed in parts per billion (ppb). For some Asp signal sets it was not possible to unambiguously determine the respective Asp within the sequence. In such cases all possible Asp are given and the one used for calculating the shift in respective to the peptide only sample is underlined. The number of involved Asp residues in binding is not affected, as always, the same number of involved Asp residues can be determined, even if the signals sets get permutated for calculating the shifts.

	NH	Hα	Hβ1	Hβ2	Hγ1	Hγ2
**Asp1**	n.d.	5.23	2.74	2.45	-	-
		(+282 ppb)	(−37 ppb)	(−116 ppb)		
**Asp3/5/9**	8.04	4.66	3.05	2.66	-	-
	(−370 ppb)	(+30 ppb)	(+290 ppb)	(−40 ppb)		
**Asp3/5/9**	8.24	4.66	2.91	2.70	-	-
	(−120 ppb)	(+20 ppb)	(+180 ppb)	(−20 ppb)		
**Asp3/5/9**	8.85	*n.d.*	2.82	2.68	-	-
	(+510 ppb)		(+80 ppb)	(+70 ppb)		
**Glu12**	8.27	4.25	2.14	2.02	2.27	2.27
	(−60 ppb)	(−10 ppb)	(+60 ppb)	(+70 ppb)	(+20 ppb)	(+20 ppb)

Table S21 Chemical shifts (in ppm) of Asp and Glu signals observed for the 1:1 EF1-G-cyc to Sm^{3+} complex after metal-saturation determined with a combination of the ^{1}H spectra of the titration series and a TOCSY spectrum. Metal-induced signal shifts (in parentheses) are expressed in parts per billion (ppb). *Only one Asp signal set can be found again, all others vanish in the presence of access metal.

	NH	Hα	Hβ1	Hβ2	Hγ1	Hγ2
**Asp1**	*n.d.*	*n.d.*	*n.d.*	*n.d.*		
**Asp3/5/9***	*n.d.*	5.00	3.35	3.03	-	-
**Glu12**	*n.d.*	4.24	2.22	2.17	2.36	2.32
		(−20 ppb)	(+140 ppb)	(+220 ppb)	(+110 ppb)	(+70 ppb)

Table S22 Chemical shifts (in ppm) of Asp and Glu signals observed for the 1:1 EF1-G-cyc to Lu^{3+} complex after metal-saturation determined with a combination of the ^{1}H spectra of the titration series and a TOCSY spectrum. Metal-induced signal shifts (in grey and in parentheses) are expressed in parts per billion (ppb). For some Asp signal sets it was not possible to unambiguously determine the respective Asp within the sequence. In such cases all possible Asp are given and the one used for calculating the shift in respective to the peptide only sample is underlined. The number of involved Asp residues in binding is not affected, as always, the same number of involved Asp residues can be determined, even if the signals sets get permutated for calculating the shifts.

	NH	Hα	Hβ1	Hβ2	Hγ1	Hγ2
**Asp1**	*n.d.*	5.44	2.76	2.33	-	-
		(+490 ppb)	(−20 ppb)	(−240 ppb)		
**Asp3/5/9** (3)	8.16	4.63	2.94	2.71	-	-
	(−250 ppb)	(0 ppb)	(+180 ppb)	(+10 ppb)		
**Asp3/5/9** (5)	9.02	*n.d.*	3.02	2.72	-	-
	(+660 ppb)		(+290 ppb)	(0 ppb)		
**Asp3/5/9** (9)	8.52	*n.d.*	2.91	2.57	-	-
	(+180 ppb)		(+170 ppb)	(−40 ppb)		
**Glu12**	8.26	4.22	2.16	2.02	2.26	2.26
	(−70 ppb)	(−40 ppb)	(+80 ppb)	(+70 ppb)	(+10 ppb)	(+10 ppb)

Table S23 Chemical shifts (in ppm) of the Pro2 spin system observed for the 1:1 EF1-cyc to Ln^{3+} complex (Ln = La, Sm, Lu) after metal-saturation determined with a combination of the ^{1}H spectra of the titration series and a TOCSY spectrum. Metal-induced signal shifts (in parentheses) are expressed in parts per billion (ppb).

	NH	Hα	Hβ1	Hβ2	Hγ1/2	Hδ1	Hδ2
**La^{3+}**	-	4.49	2.31	2.06	2.05	3.94	3.81
		(+70 ppb)	(0 ppb)	(+70 ppb)	(+490 ppb)	(+110 ppb)	(+60 ppb)
**Sm^{3+}**	-	4.47	2.25	2.00	1.90	3.85	3.73
		(+50 ppb)	(−60 ppb)	(+10 ppb)	(+490 ppb)	(+20 ppb)	(−20 ppb)
**Lu^{3+}**	-	4.25	2.25	2.14	2.04	3.79	3.79
		(−170 ppb)	(−60 ppb)	(+150 ppb)	(+490 ppb)	(−40 ppb)	(+40 ppb)

Table S24 Chemical shifts (in ppm) of the Pro11 spin system observed for the 1:1 EF1-R-cyc to Ln^{3+} complex (Ln = La, Sm, Lu) after metal-saturation determined with a combination of the ^{1}H spectra of the titration series and a TOCSY spectrum. Metal-induced signal shifts (in parentheses) are expressed in parts per billion (ppb).

	NH	Hα	Hβ1	Hβ2	Hγ1/2	Hδ1	Hδ2
**La^{3+}**	-	4.54	2.26	2.09	2.05	3.79	3.79
		(+140 ppb)	(−60 ppb)	(+120 ppb)	(+20 ppb)	(−20 ppb)	(+20 ppb)
**Sm^{3+}**	-	4.28	2.10	2.02	1.98	3.74	3.74
		(−120 ppb)	(−220 ppb)	(+50 ppb)	(−50 ppb)	(−70 ppb)	(−30 ppb)
**Lu^{3+}**	-	4.52	2.28	2.08	2.03	3.82	3.82
		(+120 ppb)	(−40 ppb)	(+110 ppb)	(0 ppb)	(−10 ppb)	(+50 ppb)

Table S25 Chemical shifts (in ppm) of the Pro2 spin system observed for the 1:1 EF1-G-cyc to Ln^{3+} complex (Ln = La, Sm, Lu) after metal-saturation determined with a combination of the ^{1}H spectra of the titration series and a TOCSY spectrum. Metal-induced signal shifts (in parentheses) are expressed in parts per billion (ppb).

	NH	Hα	Hβ1	Hβ2	Hγ	Hδ1	Hδ2
**La^{3+}**	-	4.42	2.31	2.06	2.03	3.96	3.74
		(−30 ppb)	(+40 ppb)	(+80 ppb)	(0 ppb)	(+90 ppb)	(−80 ppb)
**Sm^{3+}**	-	4.38	2.21	1.92	1.89	3.98	3.71
		(−70 ppb)	(−60 ppb)	(−60 ppb)	(−140 ppb)	(+110 ppb)	(−110 ppb)
**Lu^{3+}**	-	4.42	2.36	2.06	2.03	3.62	3.62
		(−30 ppb)	(+90 ppb)	(+80 ppb)	(0 ppb)	(−250 ppb)	(−200 ppb)

Table S26 Chemical shifts (in ppm) of the Gly6 spin system observed for the 1:1 EF1-cyc to Ln^{3+} complex (Ln = La, Sm, Lu) after metal-saturation determined with a combination of the ^{1}H spectra of the titration series and a TOCSY spectrum. Metal-induced signal shifts (in parentheses) are expressed in parts per billion (ppb).

	NH	Hα1	Hα2
**La^{3+}**	*n.d.*	4.20	3.86
		(+100 ppb)	(−100 ppb)
**Sm^{3+}**	*n.d.*	4.12	3.79
		(+30 ppb)	(−170 ppb)
**Lu^{3+}**	*n.d.*	4.27	3.79
		(+180 ppb)	(−170 ppb)

Table S27 Chemical shifts (in ppm) of the Gly7 spin system observed for the 1:1 EF1-R-cyc to Ln^{3+} complex (Ln = La, Sm, Lu) after metal-saturation determined with a combination of the ^{1}H spectra of the titration series and a TOCSY spectrum. Metal-induced signal displacements (in parentheses) are expressed in parts per billion (ppb).

	NH	Hα1	Hα2
**La^{3+}**	*n.d.*	4.09	3.85
		(−10 ppb)	(−60 ppb)
**Sm^{3+}**	8.57	4.06	3.81
	(+260 ppb)	(−40 ppb)	(−100 ppb)
**Lu^{3+}**	8.48	4.09	3.88
	(+170 ppb)	(−10 ppb)	(−30 ppb)

Table S28 Chemical shifts (in ppm) of the Gly0 and Gly6 spin systems observed for the 1:1 EF1-G-cyc to Ln^{3+} complex (Ln = La, Sm, Lu) after metal-saturation determined with a combination of the ^{1}H spectra of the titration series and a TOCSY spectrum. Metal-induced signal displacements (in parentheses) are expressed in parts per billion (ppb).

	Gly0			Gly6		
	**NH**	**Hα1**	**Hα2**	**NH**	**Hα1**	**Hα2**
**La^{3+}**	8.36	4.03	3.86	*n.d.*	4.27	3.71
	(−20 ppb)	(−20 ppb)	(0 ppb)		(+180 ppb)	(−230 ppb)
**Sm^{3+}**	8.36	4.01	3.87	*n.d.*	4.18	3.55
	(−20 ppb)	(−40 ppb)	(+10 ppb)		(+90 ppb)	(−390 ppb)
**Lu^{3+}**	8.31	4.09	3.86	8.37	4.24	3.62
	(−70 ppb)	(+40 ppb)	(0 ppb)	(−10 ppb)	(+150 ppb)	(−320 ppb)

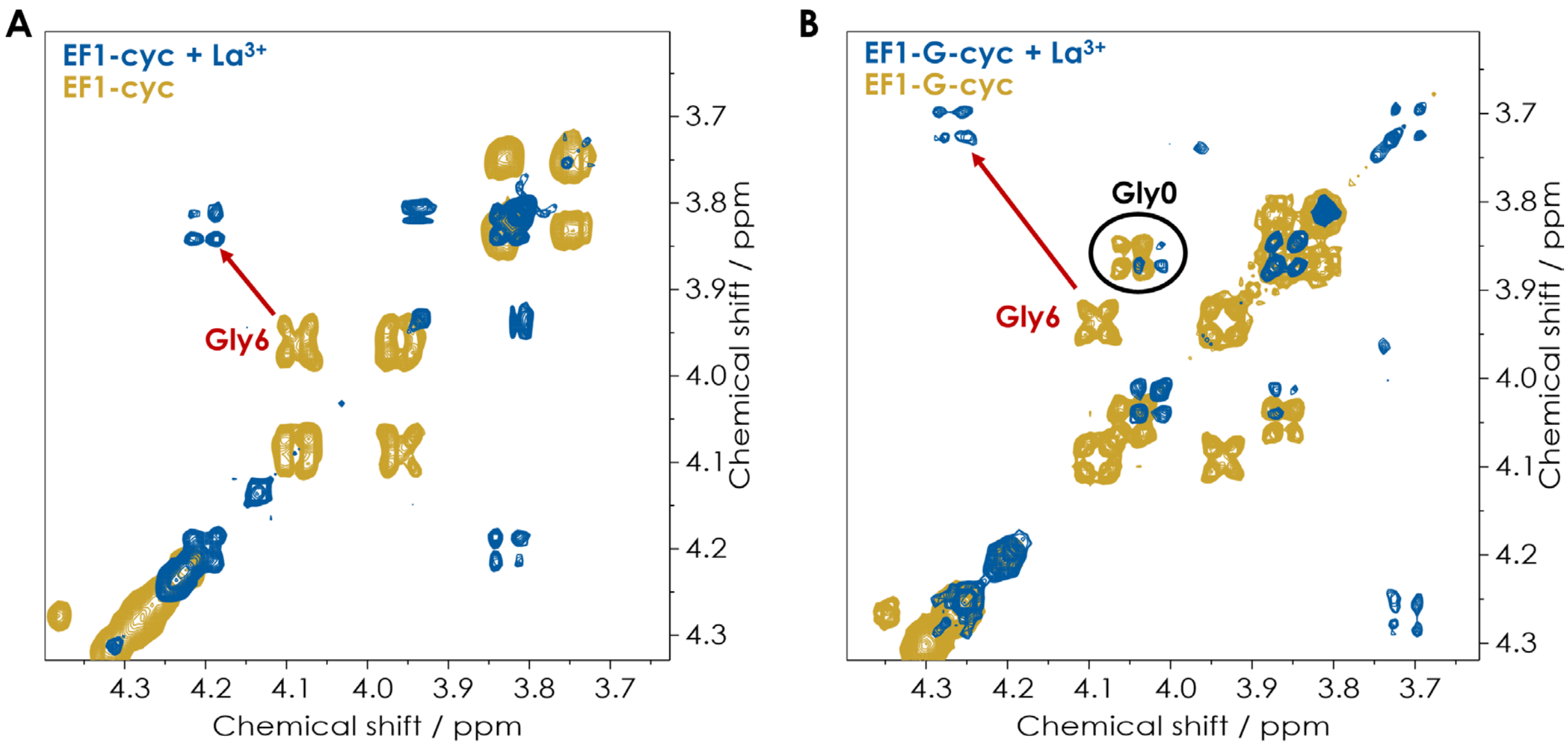

Figure S63 Gly region of superimposed TOCSY spectra of EF1-cyc **(A)** and EF1-G-cyc **(B)** in absence (gold) and presence of excess $LaCl_3$ (blue). Experimental conditions for the NMR spectra: 600 MHz, H_2O/D_2O (9:1, *v/v*) + 0.003% TMSP-d_4, pH 6.6, 30 mM MES-d_{13}, 100 mM KCl. For both peptides a significant displacement of the Gly6 Hα signals can be observed, while the Hα resonances of Gly0 remain almost unchanged.

Detailed Discussion to the Section "Influence of a Glycine Insertion between Aspartic and Glutamic Acid in EF1-cyc"

For both peptides (EF1-cyc and EF1-G-cyc) and all the metals investigated, we observe significant displacements of the Gly6 Hα signals (see Figure S63 for exemplary data with La^{3+} and Tables S26 and S28), suggesting the involvement of the adjacent Asp5 in the coordination of Ln^{3+}. In contrast to that, the Hα resonances of Gly0 remain almost unchanged (see Table S28, SI) and when looking at Gly0, Asp1, Pro2, Asp3 changes in the backbone become apparent (cf. Tables S20-S22; S25). With excess La^{3+} and Lu^{3+}, respectively, the NH signals of Asp1 are not detectable and the corresponding Hα are extremely shifted downfield (+280 and +490 ppb) while β1 and β2 reveal minor (−37 and −20 ppb) and major (−120 and −240 ppb) displacements, but upfield. Furthermore, in the Lu^{3+} complex, the Pro2 Hδ display notable upfield-shifts (−250 and −200 ppb) while in the La^{3+} complex these resonances become more distinct (+90 and −80 ppb) (cf. Table S25, SI). This supports the assumption that, against our initial hypothesis, introducing Gly0 did not facilitate the involvement of Asp1 in Ln binding, but the spectroscopic signature points to conformational adjustments upon metal ion coordination in other parts of the peptide sequence. Furthermore, none of the Glu12 resonances reveal a notable shift, thus indicating that Glu12 is not involved in the coordination (Tables S20–S22, SI). In presence of paramagnetic Sm^{3+}, only one EF1-G-cyc Asp spin system is detectable which cannot be unambiguously assigned. However, due to the significant shift of the Gly6 Hα2 resonance, we assume that Asp5 is again involved in coordination.

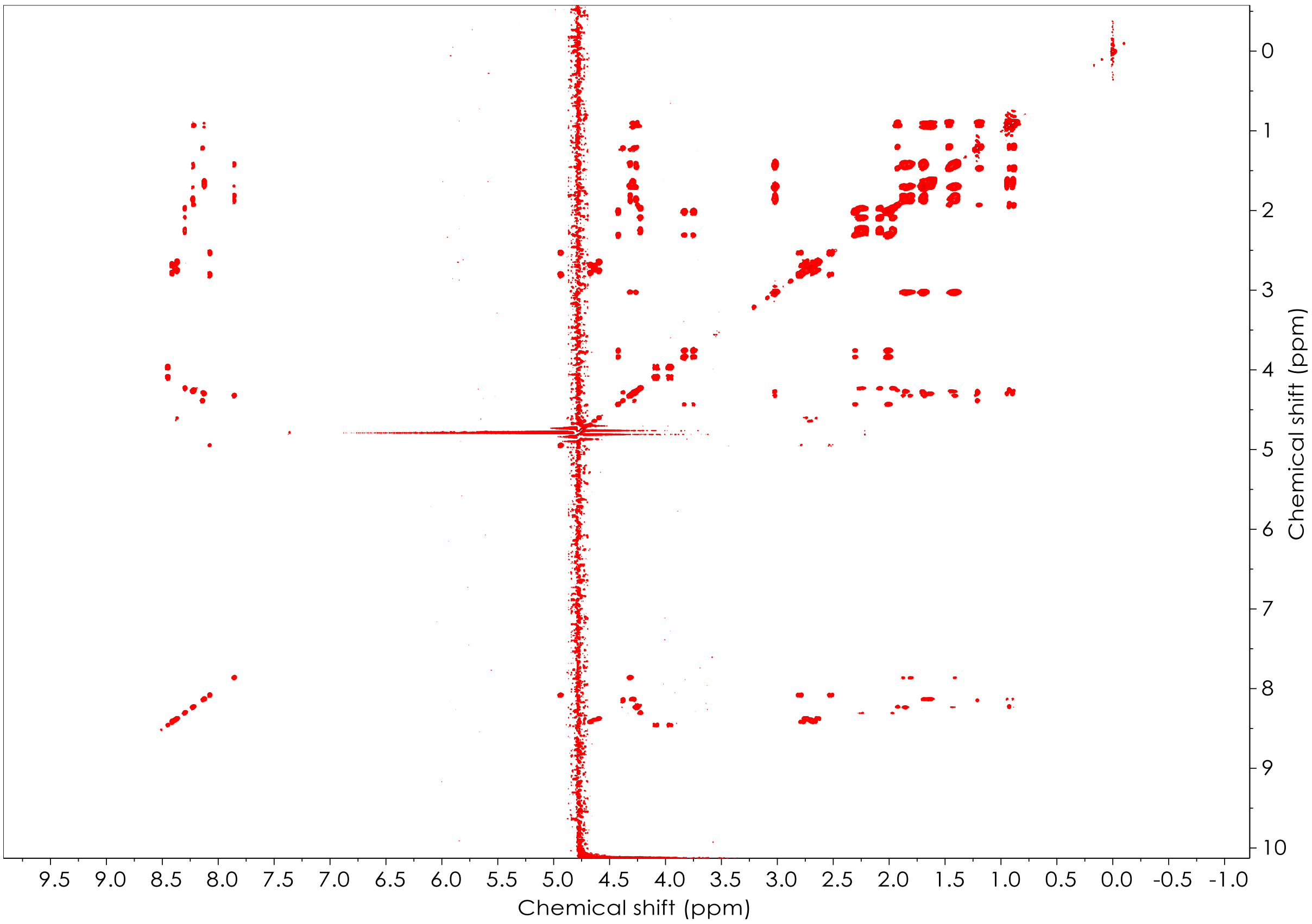

Figure S64 zTOCSY spectrum of Ln-free EF1-cyc (600 MHz, H_2O/D_2O (9:1, *v/v*) + 0.003% TMSP-d_4, 30 mM MES-d_{13}, 100 mM KCl).

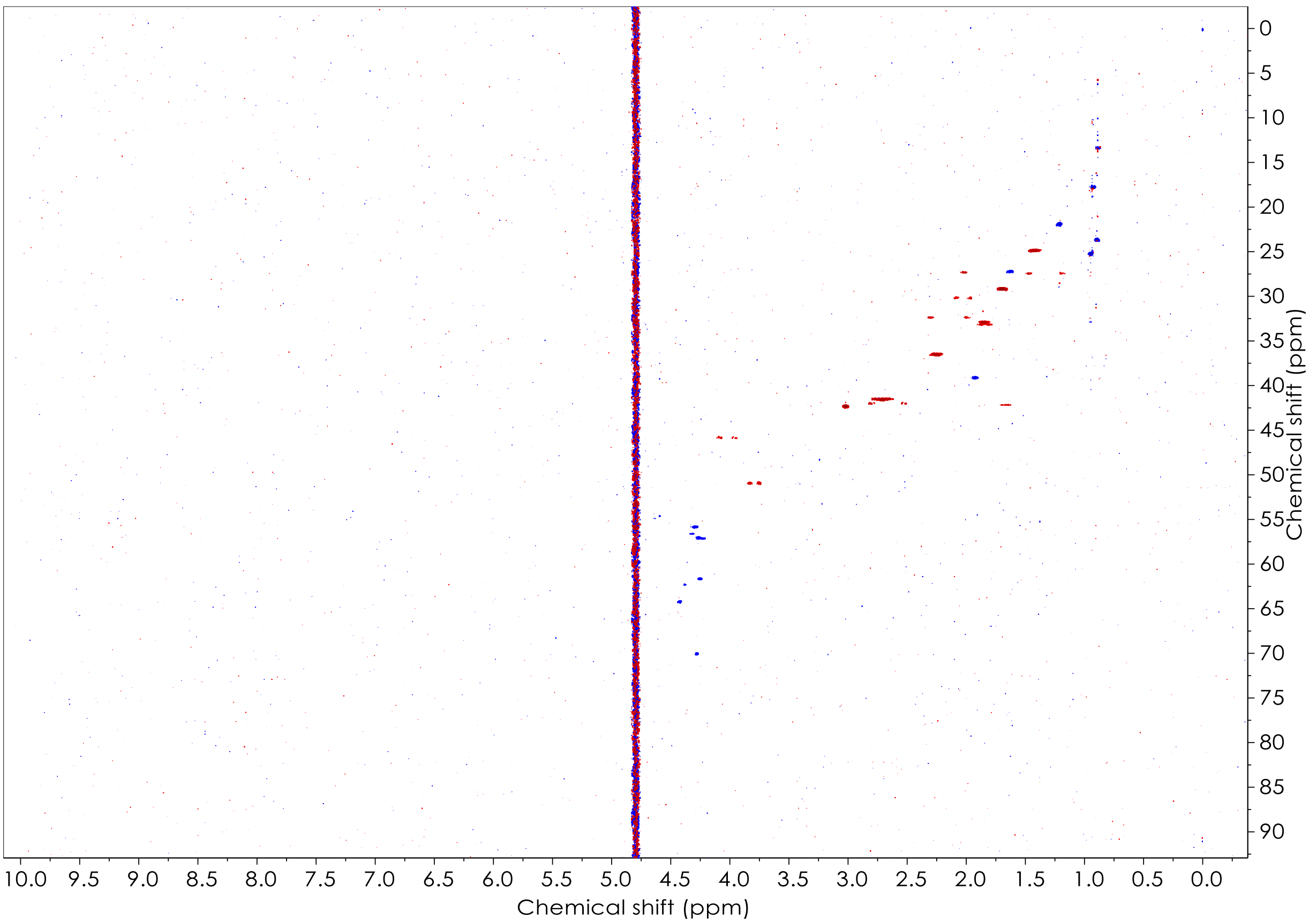

Figure S65 HSQC spectrum of Ln-free EF1-cyc (600 MHz, H_2O/D_2O (9:1, *v/v*) + 0.003% TMSP-d_4, 30 mM MES-d_{13}, 100 mM KCl).

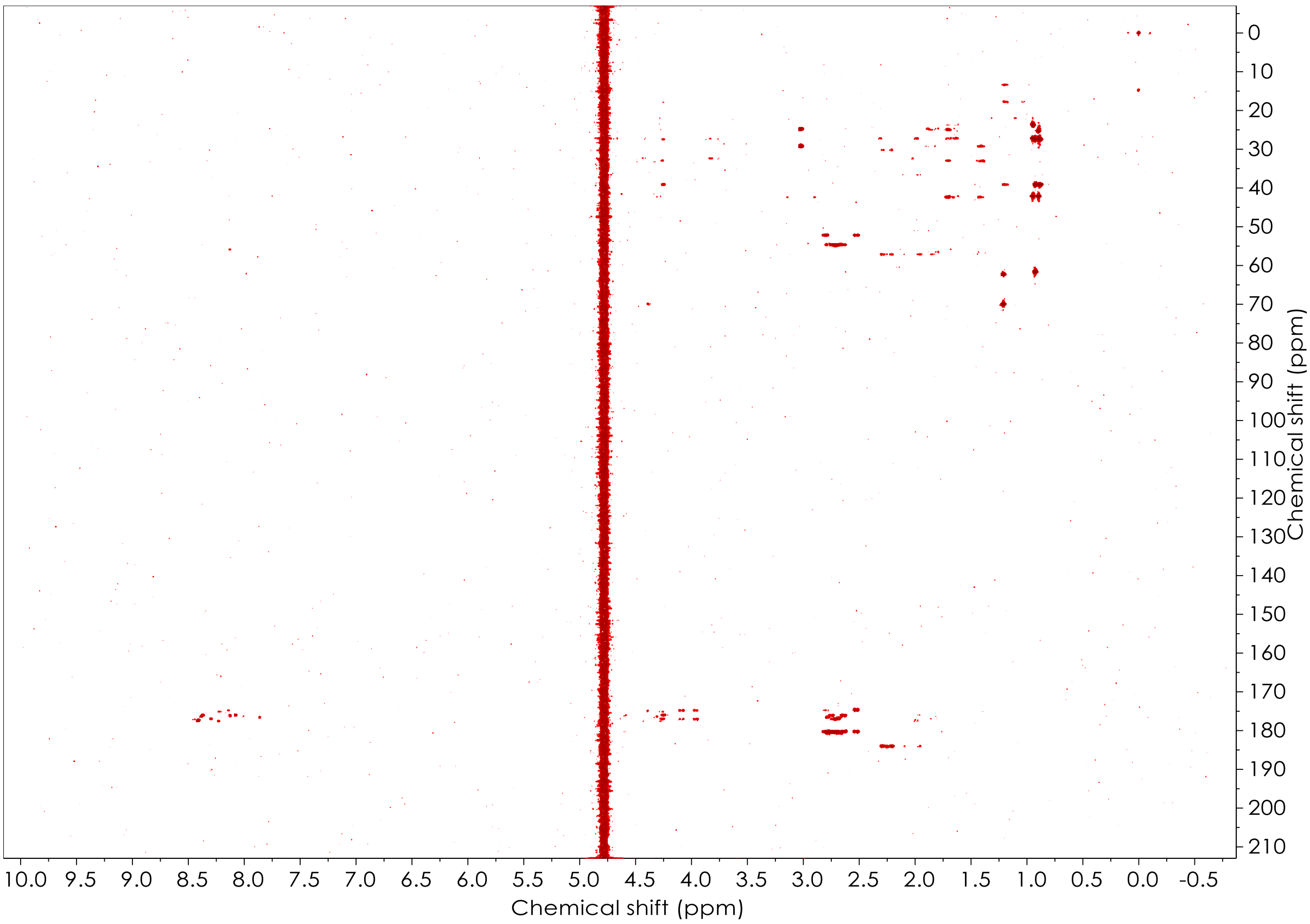

Figure S66 HMBC spectrum of Ln-free EF1-cyc (600 MHz, H_2O/D_2O (9:1, *v/v*) + 0.003% TMSP-d_4, 30 mM MES-d_{13}, 100 mM KCl).

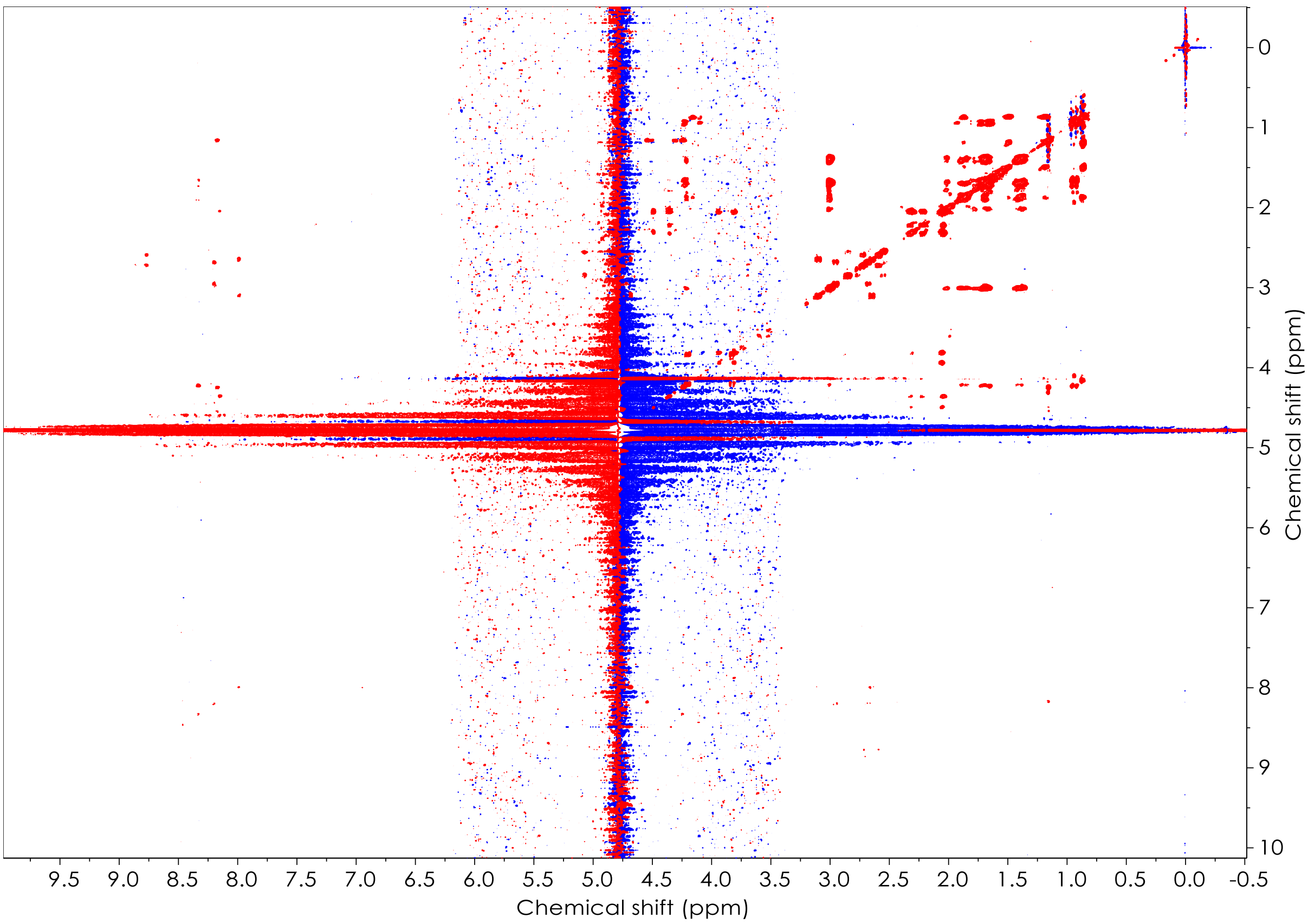

Figure S67 zTOCSY spectrum of EF1-cyc in the presence of excess $LaCl_3$ (1:10 EF:La^{3+} ratio) (600 MHz, H_2O/D_2O (9:1, *v/v*) + 0.003% TMSP-d_4, 30 mM MES-d_{13}, 100 mM KCl).

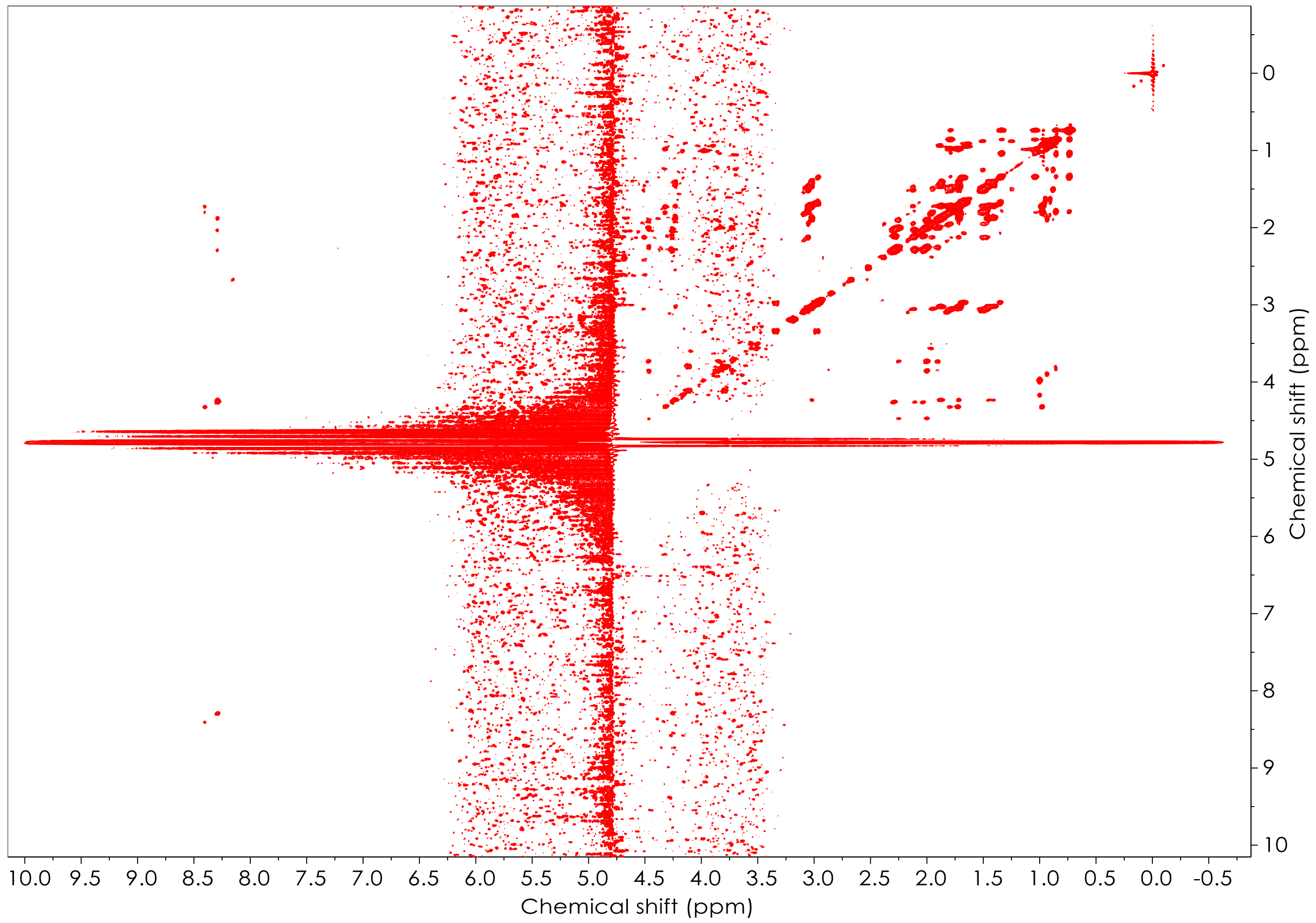

Figure S68 zTOCSY spectrum of EF1-cyc in the presence of excess $SmCl_3$ (1:2 EF:Sm^{3+} ratio) (600 MHz, H_2O/D_2O (9:1, *v/v*) + 0.003% TMSP-d_4, 30 mM MES-d_{13}, 100 mM KCl).

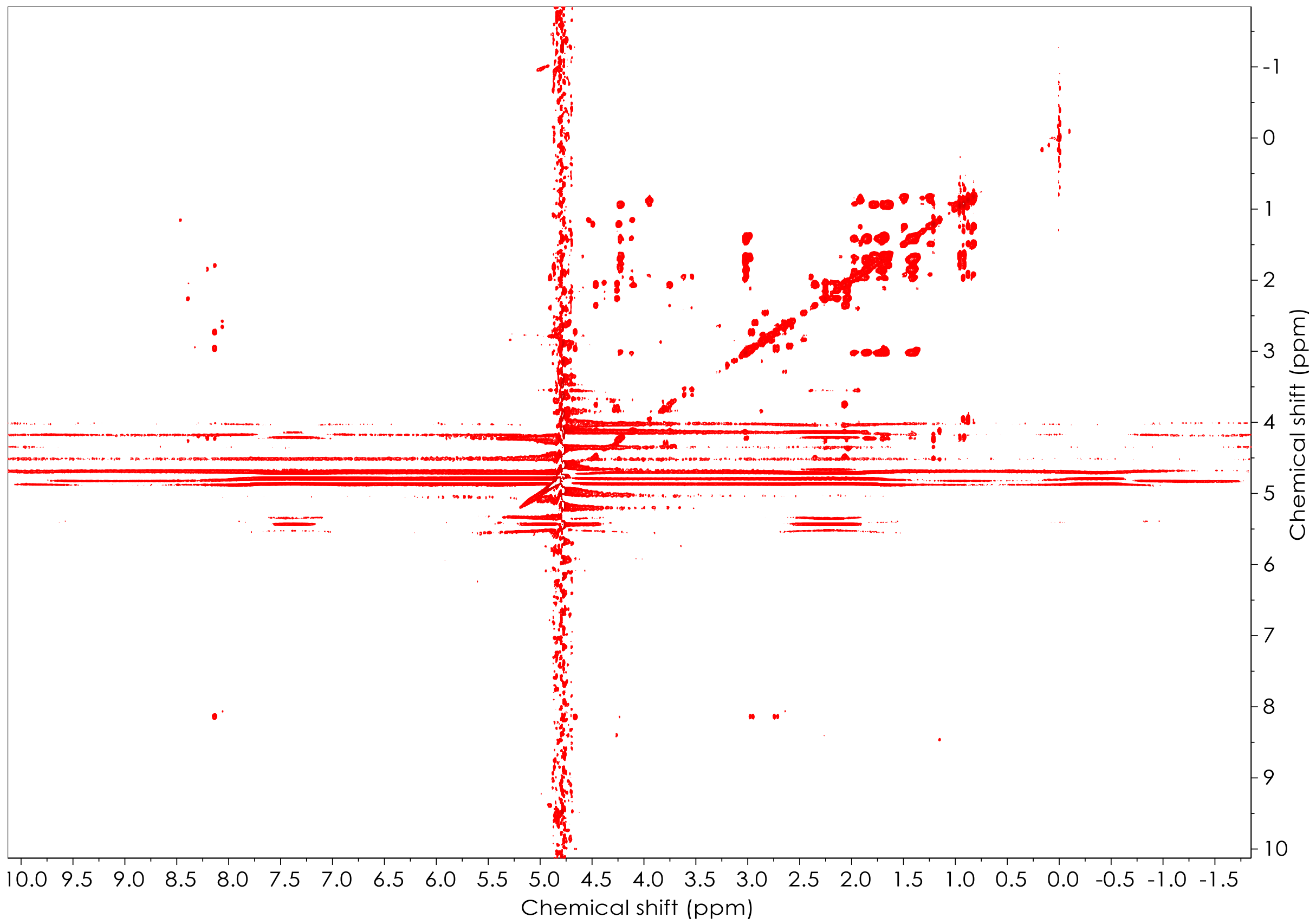

Figure S69 zTOCSY spectrum of EF1-cyc in the presence of excess $LuCl_3$ (1:2 EF:Lu^{3+} ratio) (600 MHz, H_2O/D_2O (9:1, *v/v*) + 0.003% TMSP-d_4, 30 mM MES-d_{13}, 100 mM KCl).

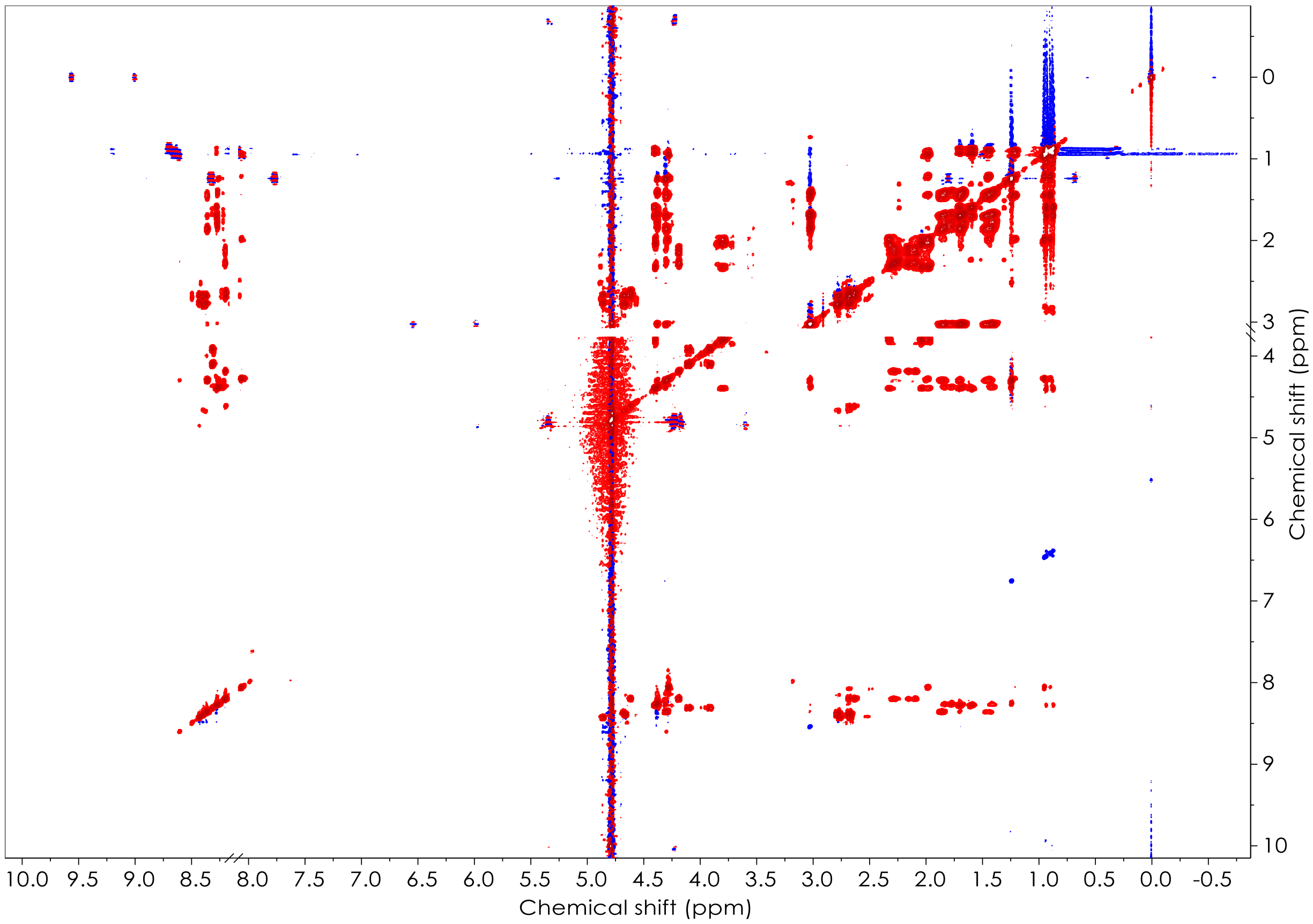

Figure S70 zTOCSY spectrum of Ln-free EF1-R-cyc (600 MHz, H_2O/D_2O (9:1, *v/v*) + 0.003% TMSP-d_4, 30 mM MES-d_{13}, 100 mM KCl).

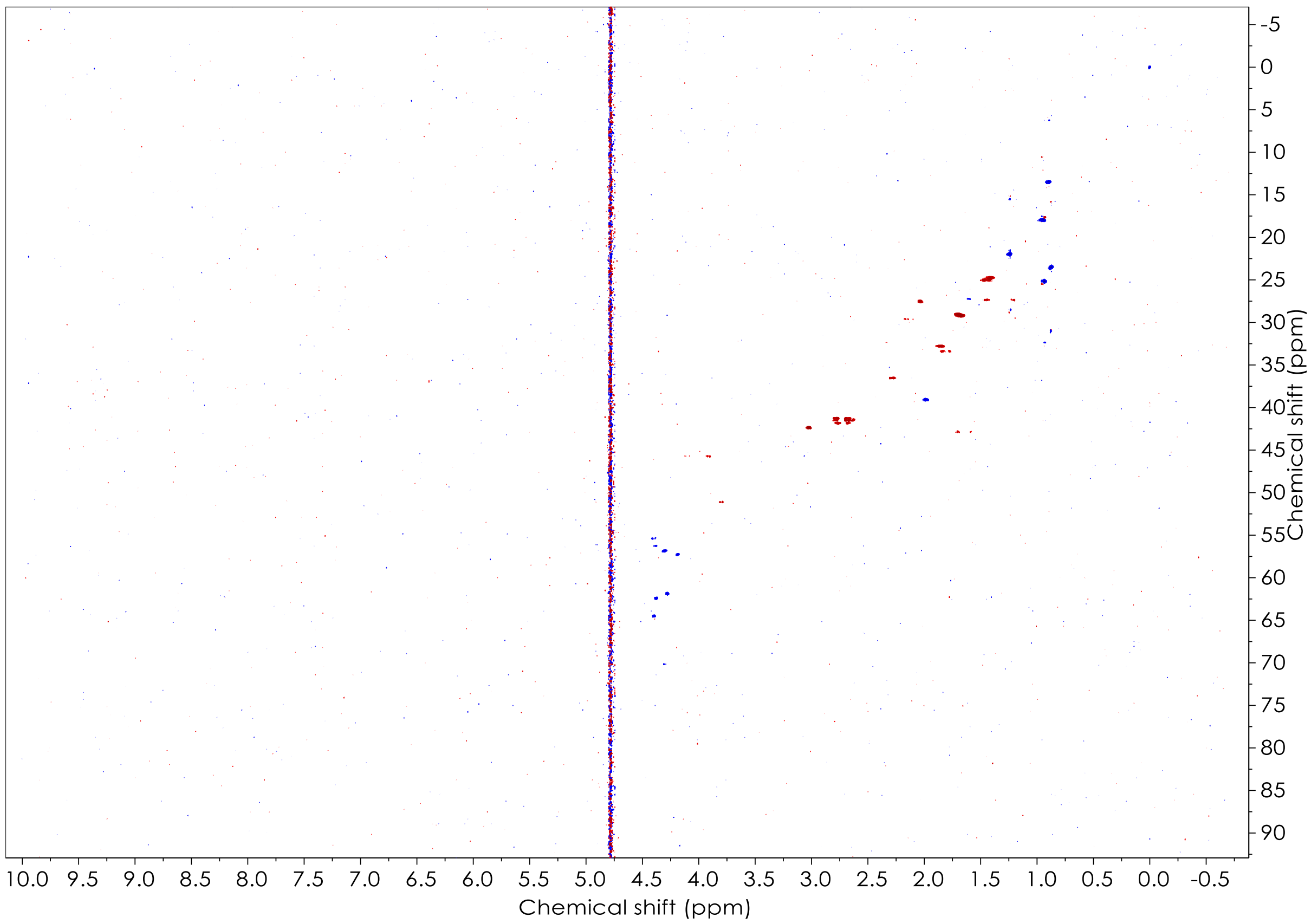

Figure S71 HSQC spectrum of Ln-free EF1-R-cyc (600 MHz, H_2O/D_2O (9:1, *v/v*) + 0.003% TMSP-d_4, 30 mM MES-d_{13}, 100 mM KCl).

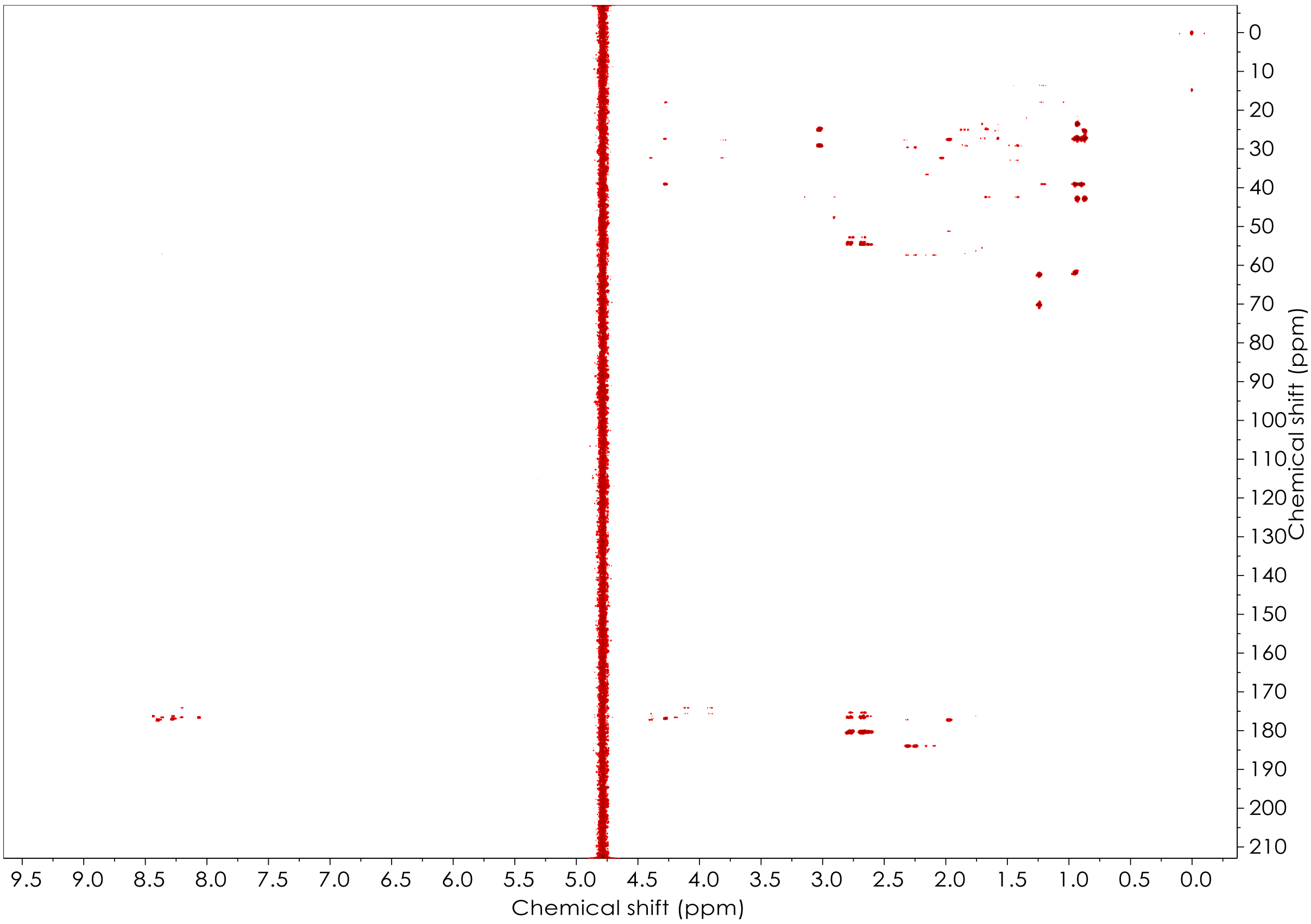

Figure S72 HMBC spectrum of Ln-free EF1-R-cyc (600 MHz, H_2O/D_2O (9:1, *v/v*) + 0.003% TMSP-d_4, 30 mM MES-d_{13}, 100 mM KCl).

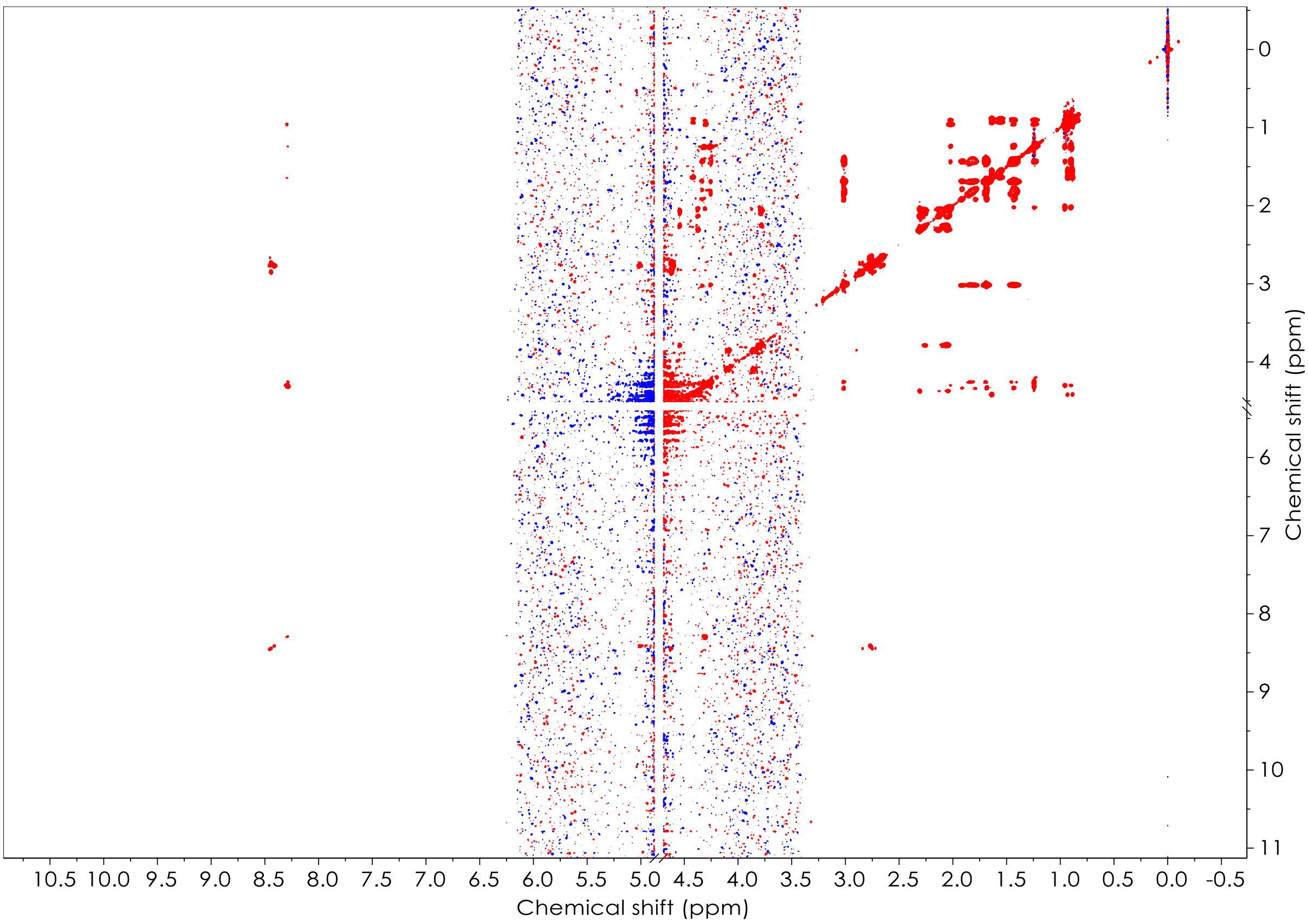

Figure S73 zTOCSY spectrum of EF1-R-cyc in the presence of excess $LaCl_3$ (1:10 EF:La^{3+} ratio) (600 MHz, H_2O/D_2O (9:1, *v/v*) + 0.003% TMSP-d_4, 30 mM MES-d_{13}, 100 mM KCl).

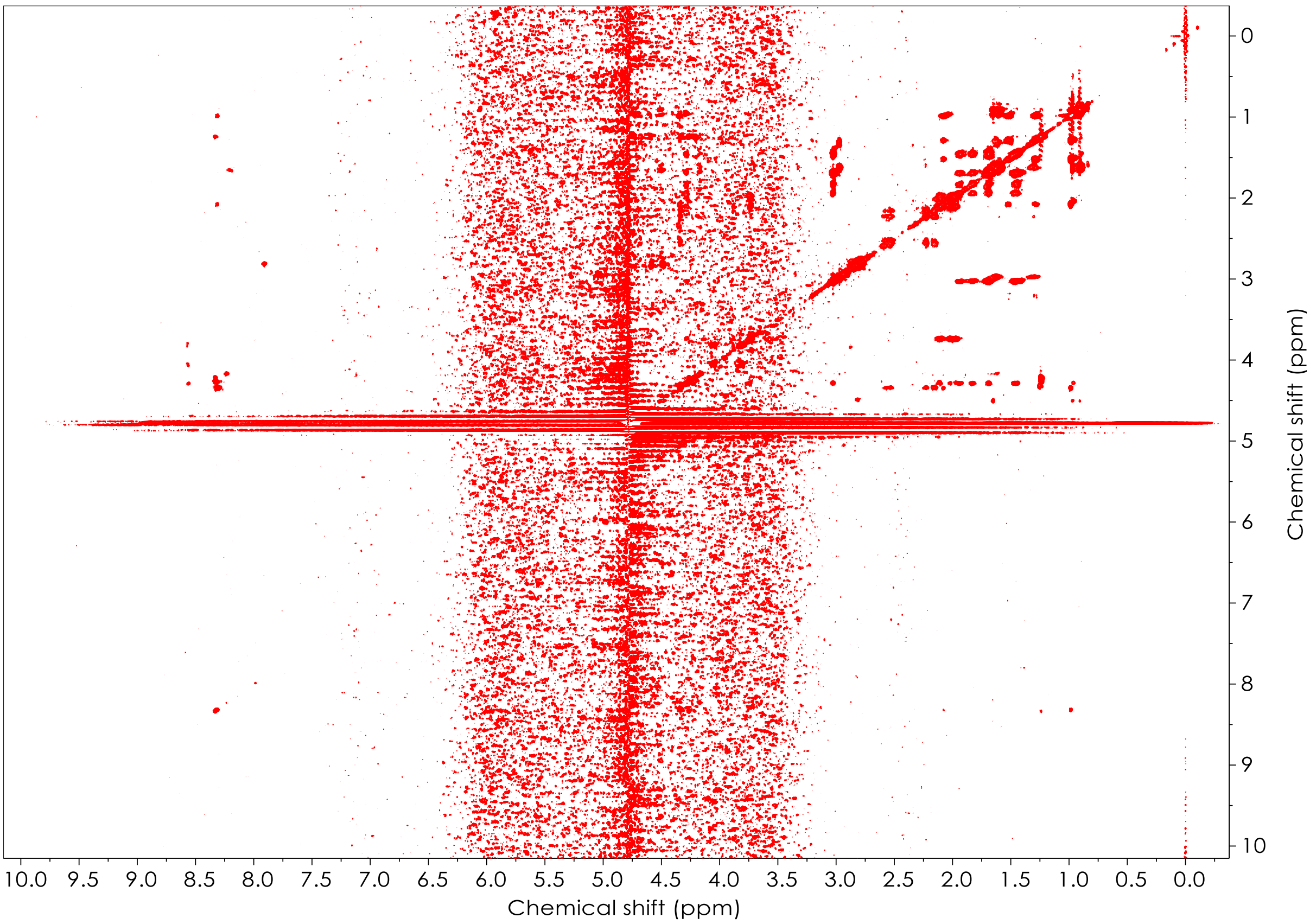

Figure S74 zTOCSY spectrum of EF1-R-cyc in the presence of excess $SmCl_3$ (1:2 EF:Sm^{3+} ratio) (600 MHz, H_2O/D_2O (9:1, *v/v*) + 0.003% TMSP-d_4, 30 mM MES-d_{13}, 100 mM KCl).

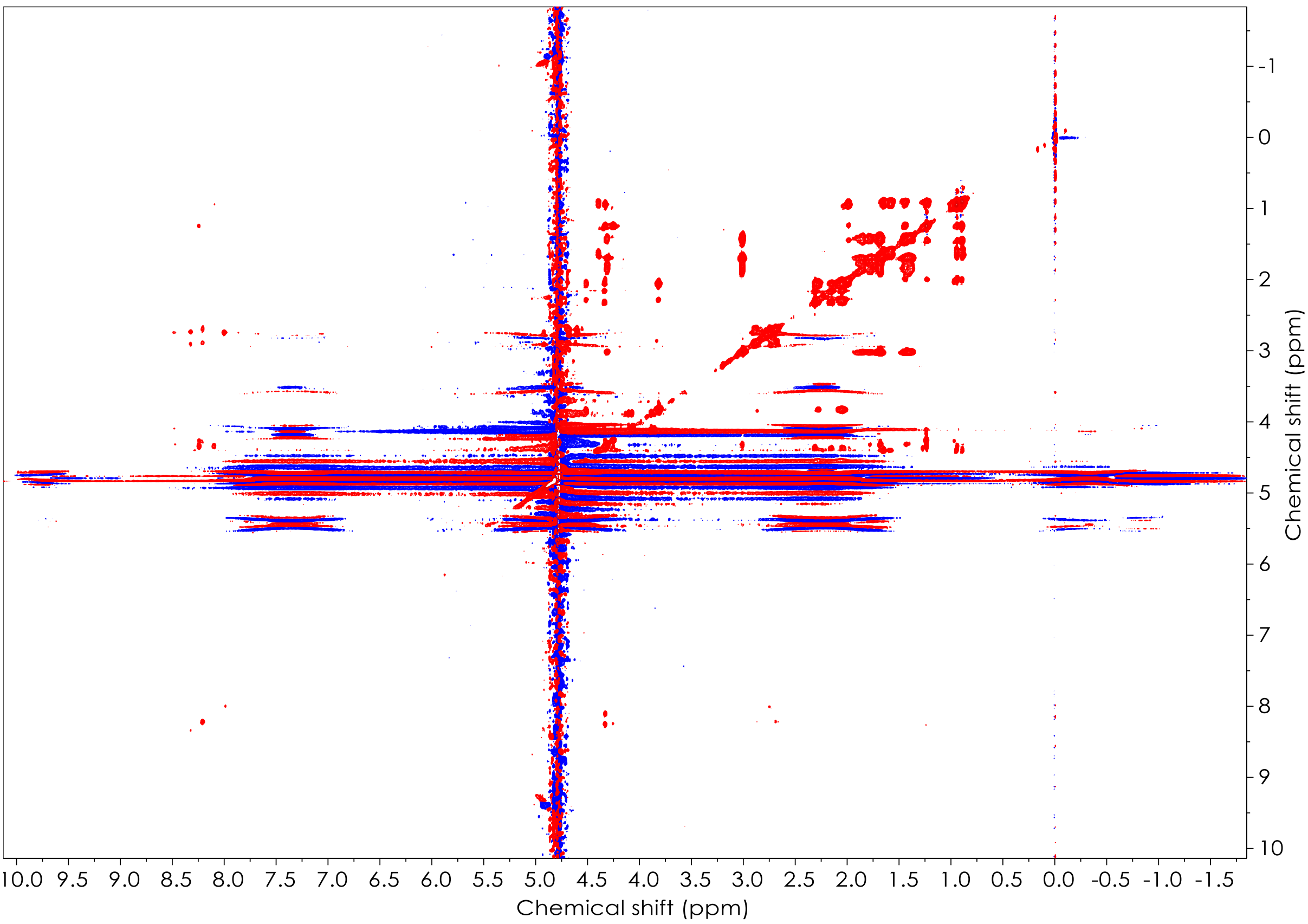

Figure S75 zTOCSY spectrum of EF1-R-cyc in the presence of excess $LuCl_3$ (1:2 EF:Lu^{3+} ratio) (600 MHz, H_2O/D_2O (9:1, *v/v*) + 0.003% TMSP-d_4, 30 mM MES-d_{13}, 100 mM KCl).

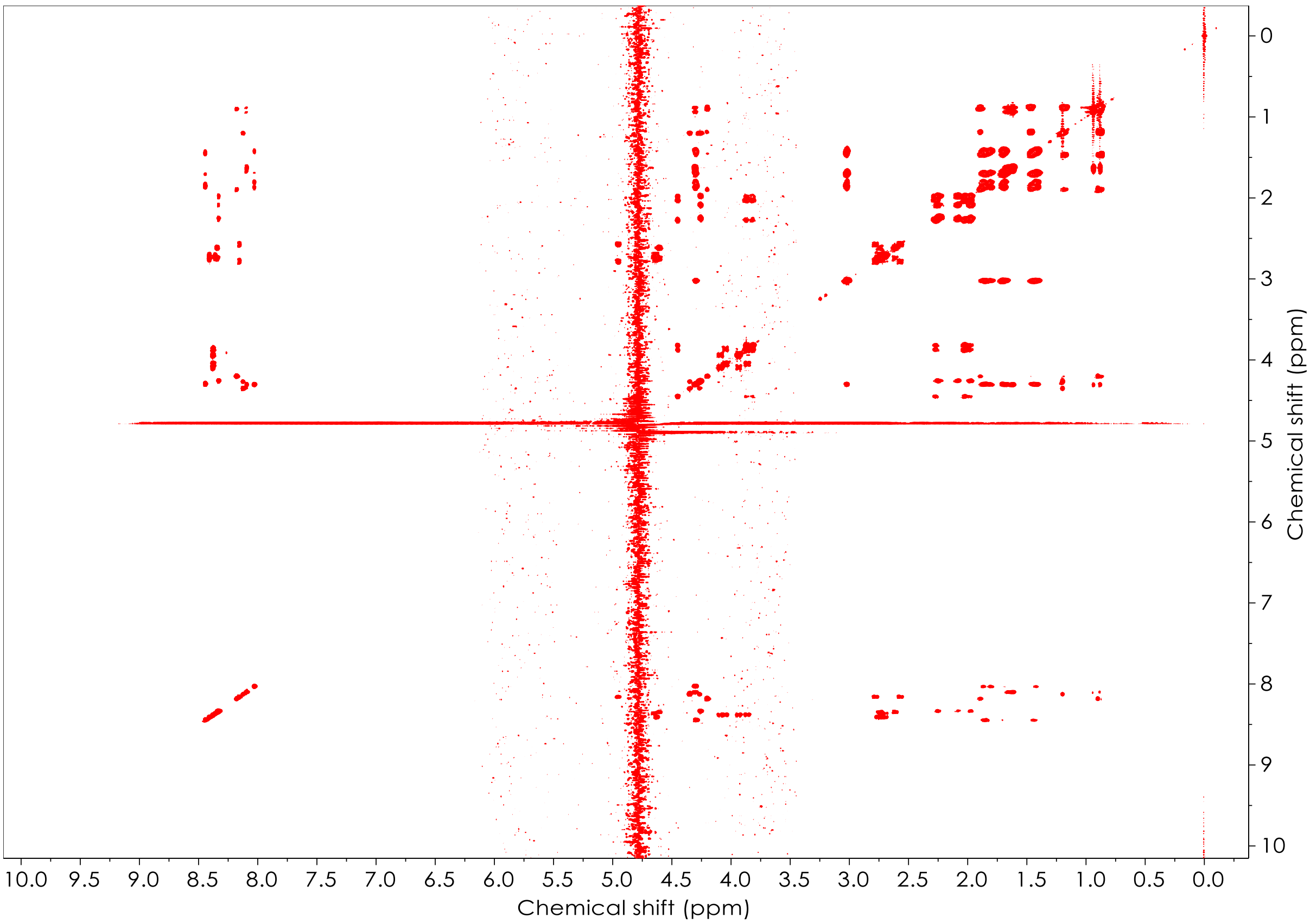

Figure S76 zTOCSY spectrum of Ln-free EF1-G-cyc (600 MHz, H_2O/D_2O (9:1, *v/v*) + 0.003% TMSP-d_4, 30 mM MES-d_{13}, 100 mM KCl).

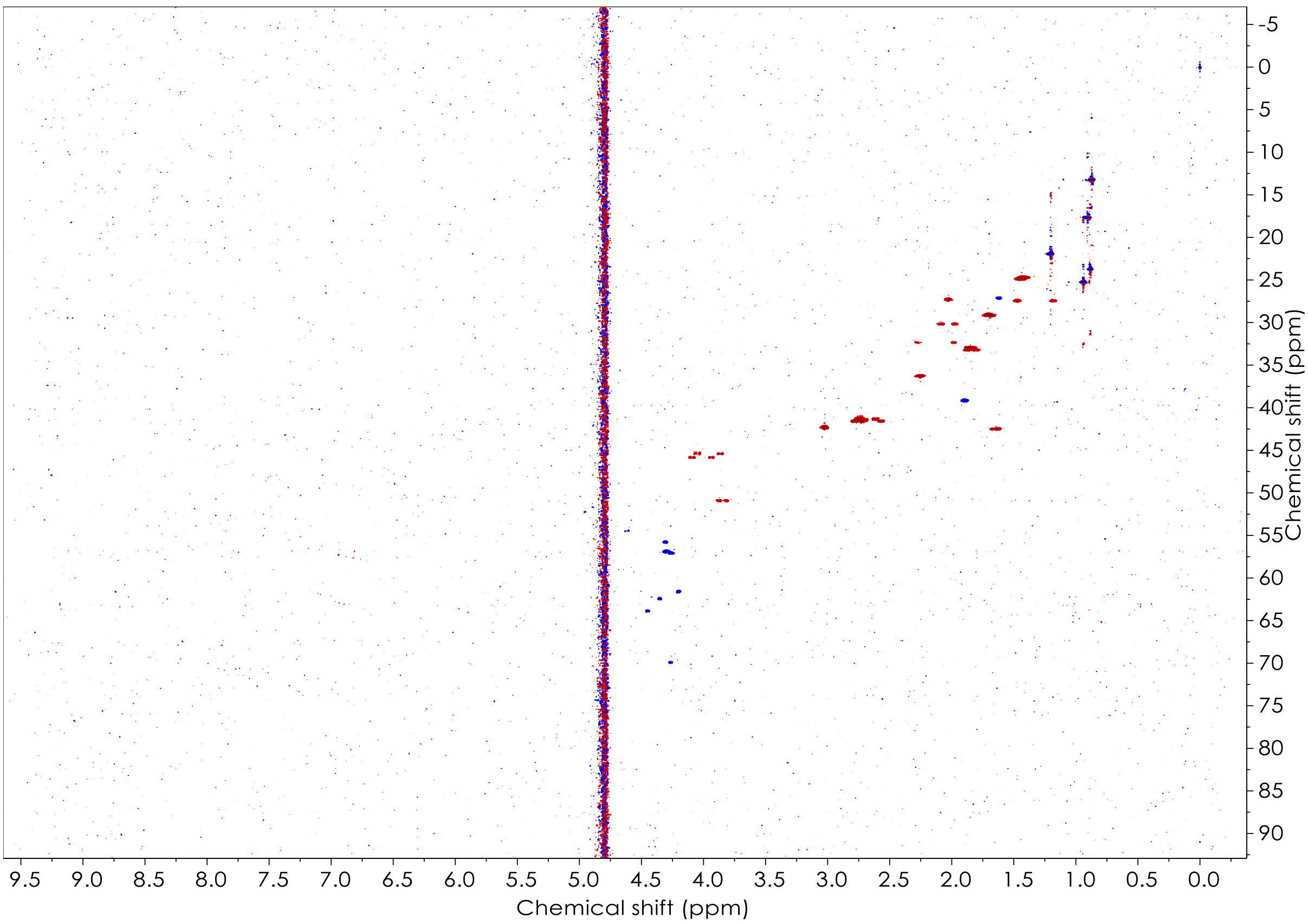

Figure S77 HSQC spectrum of Ln-free EF1-G-cyc (600 MHz, H_2O/D_2O (9:1, *v/v*) + 0.003% TMSP-d_4, 30 mM MES-d_{13}, 100 mM KCl).

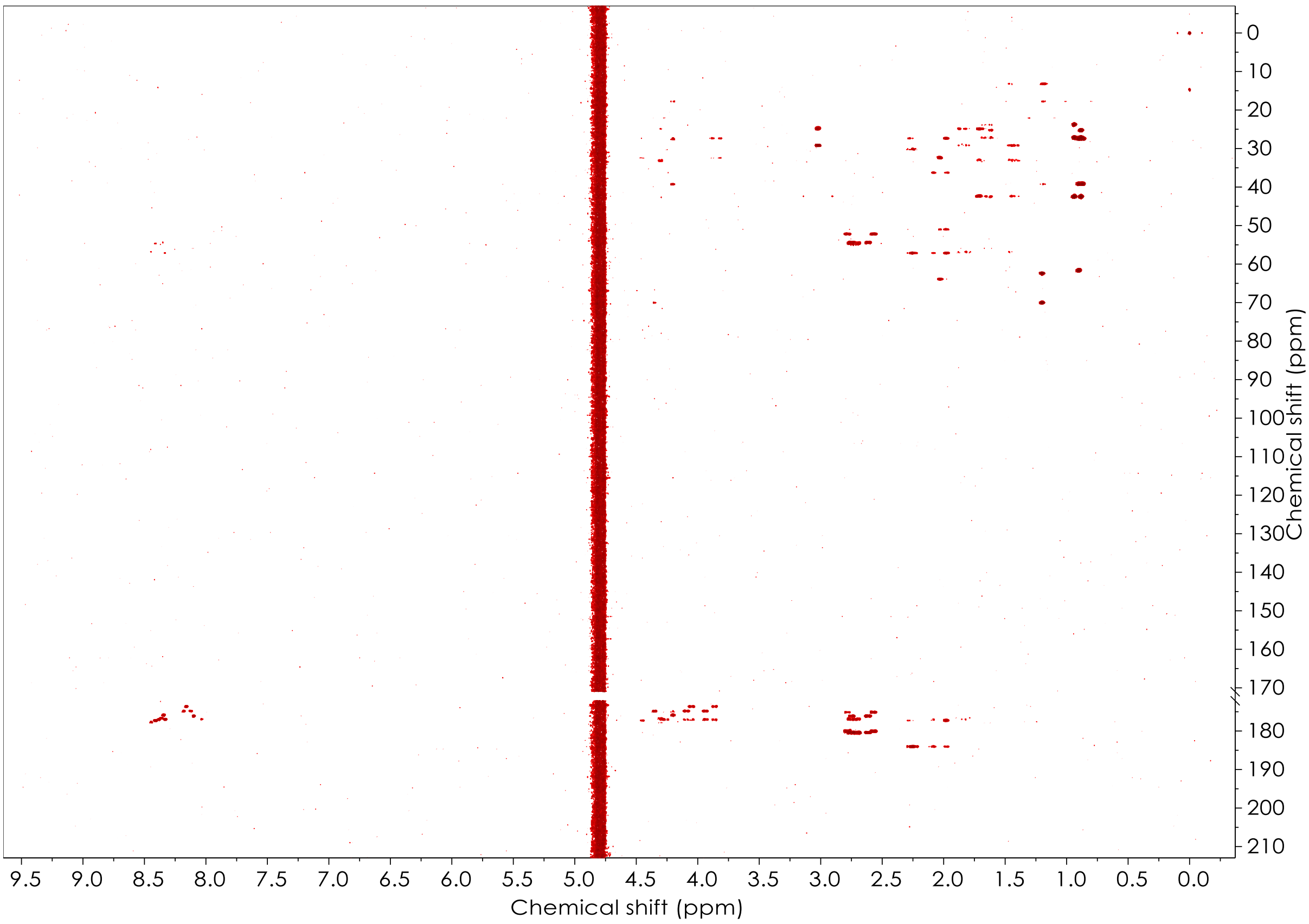

Figure S78 HMBC spectrum of Ln-free EF1-G-cyc (600 MHz, H_2O/D_2O (9:1, *v/v*) + 0.003% TMSP-d_4, 30 mM MES-d_{13}, 100 mM KCl).

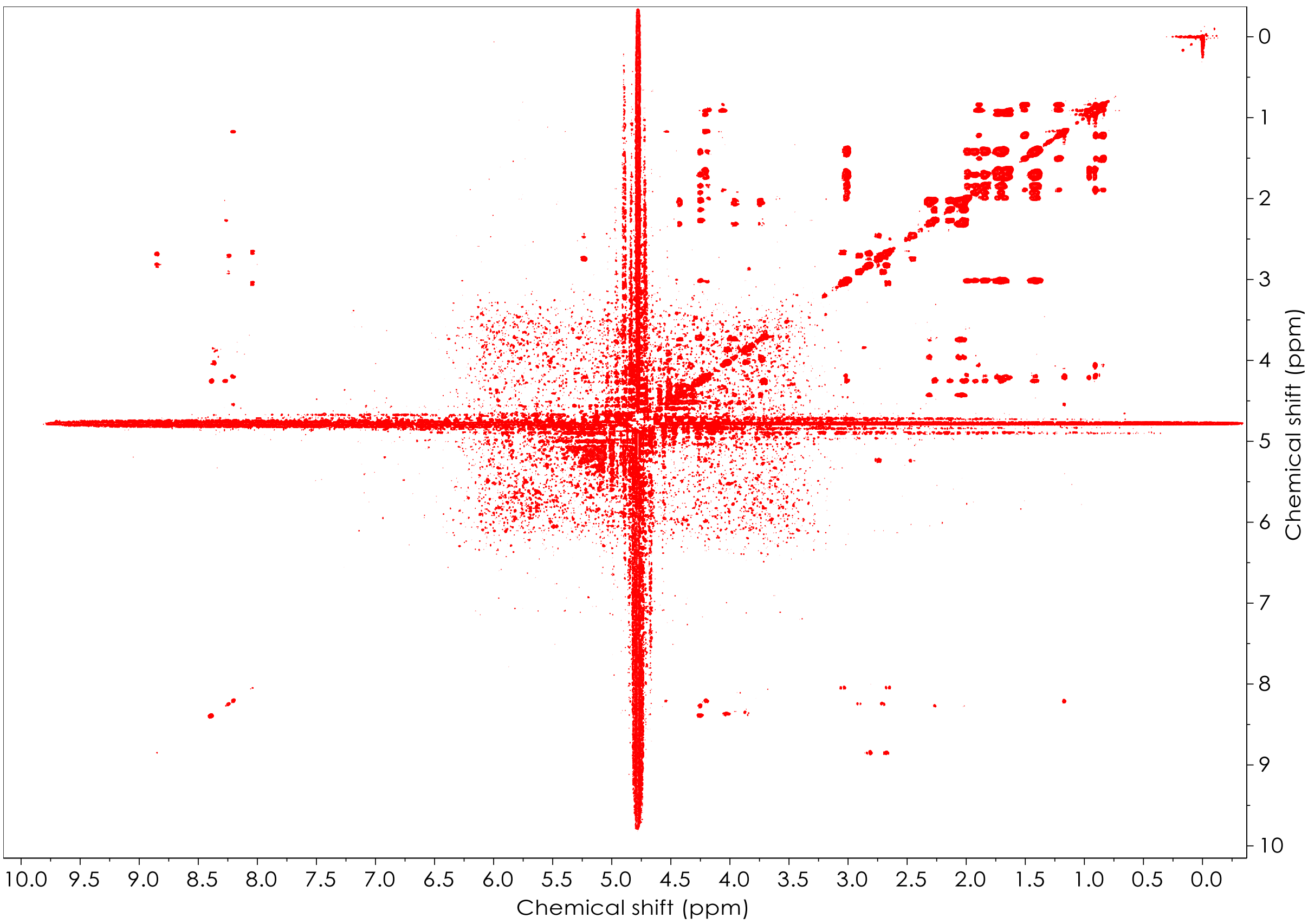

Figure S79 zTOCSY spectrum of EF1-G-cyc in the presence of excess $LaCl_3$ (1:2 EF:La^{3+} ratio) (600 MHz, H_2O/D_2O (9:1, *v/v*) + 0.003% TMSP-d_4, 30 mM MES-d_{13}, 100 mM KCl).

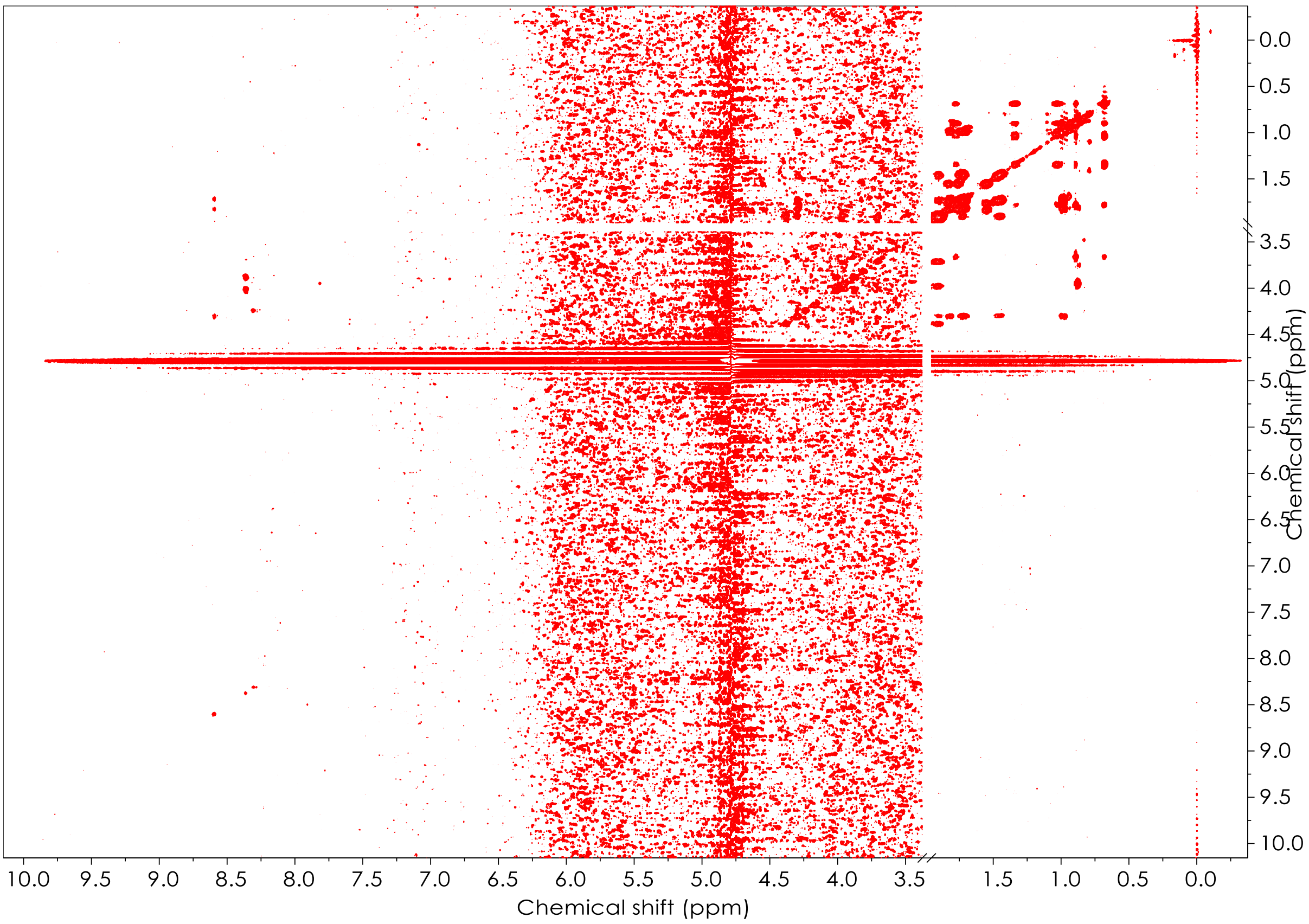

Figure S80 zTOCSY spectrum of EF1-G-cyc in the presence of excess $SmCl_3$ (1:2 EF:Sm^{3+} ratio) (600 MHz, H_2O/D_2O (9:1, *v/v*) + 0.003% TMSP-d_4, 30 mM MES-d_{13}, 100 mM KCl).

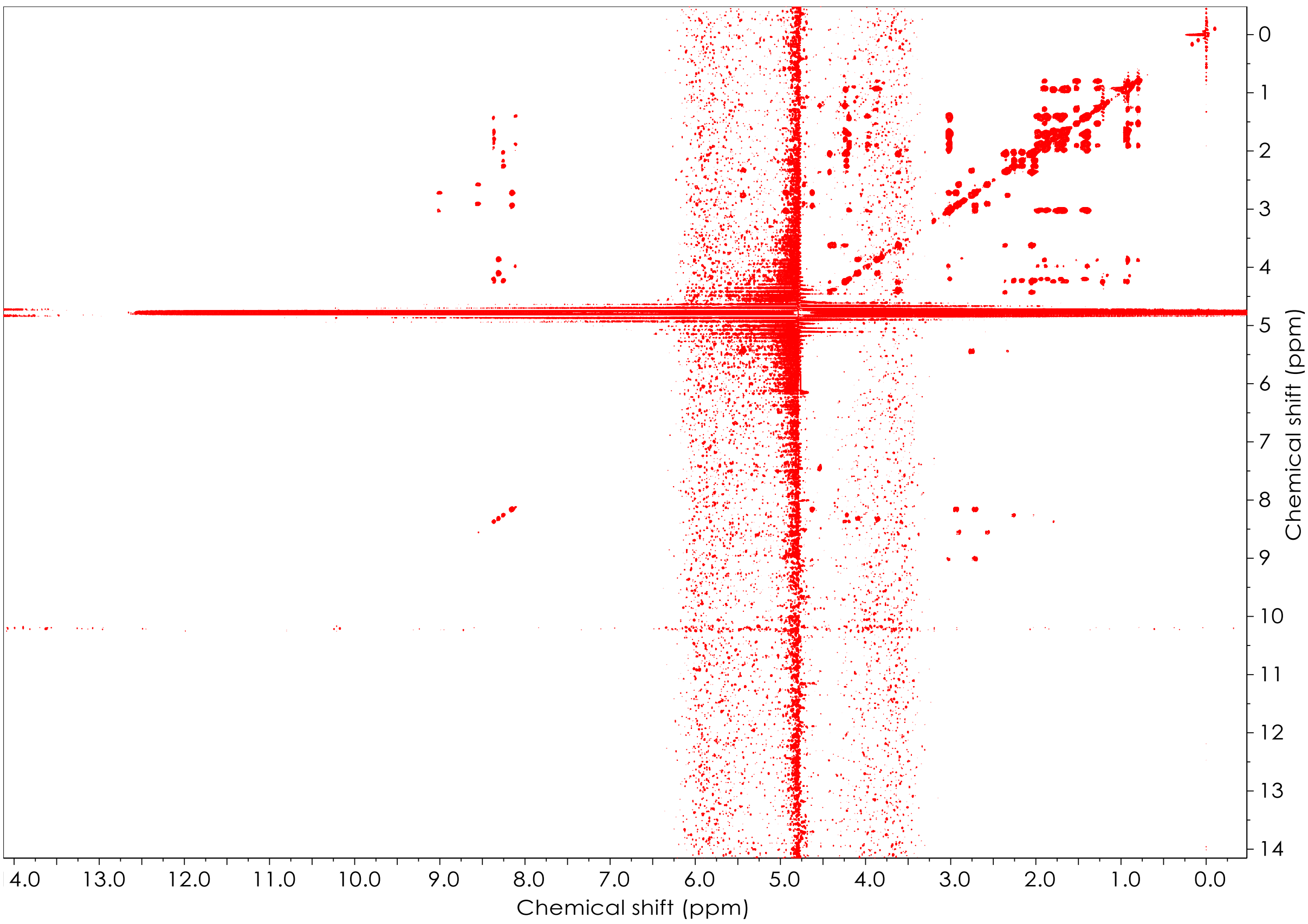

Figure S81 zTOCSY spectrum of EF1-G-cyc in the presence of excess $LuCl_3$ (1:2 EF:Lu^{3+} ratio) (600 MHz, H_2O/D_2O (9:1, *v/v*) + 0.003% TMSP-d_4, 30 mM MES-d_{13}, 100 mM KCl).

4.5 Supplementary Mass Spectrometry Data

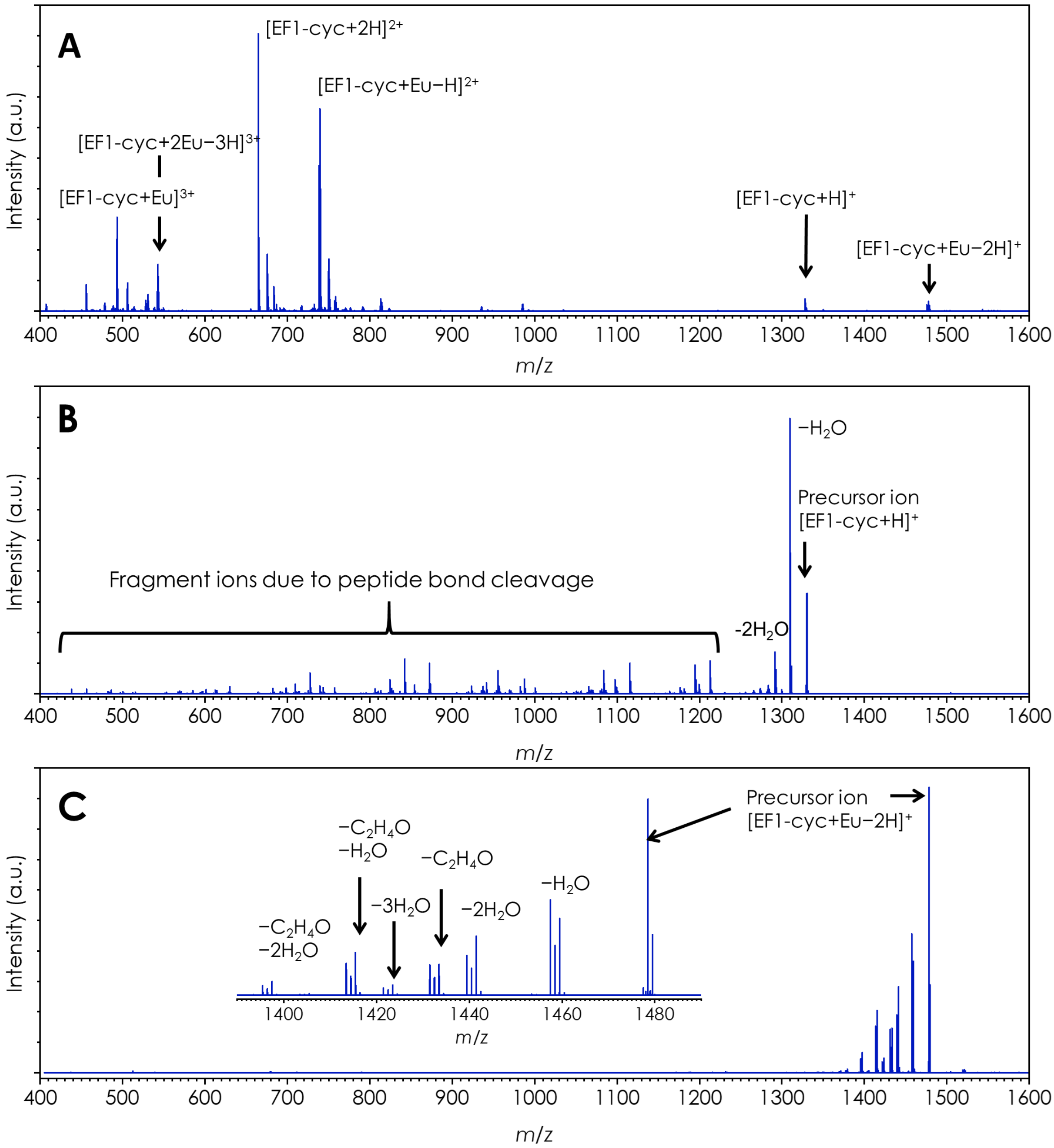

Figure S82 (A) Overview mass spectrum of a solution of 10 µM EF1-cyc with 50 µM $EuCl_3$ in LCMS water. Note that the relative intensities of the different charge states depend on the source conditions such as spray voltage and tube lens voltage. **(B)** Collision-induced dissociation of [EF1-cyc+H]$^+$, *m/z* 1326.6376. Besides water loss, a large number of fragments due to peptide bond cleavage of the backbone is observed (see Table S28). **(C)** Collision-induced dissociation of [EF1-cyc+Eu−2H]$^+$, *m/z* 1475.5340. The fragmentation pattern is dominated by loss of multiple water and neutral C_2H_4O molecules; backbone fragmentation is basically absent.

Table S29 Collision-induced dissociation of [EF1-cyc+H]$^+$ ($C_{56}H_{91}N_{14}O_{23}^+$), matched peaks. Observed fragment ions of [EF1-cyc+H]$^+$ (see Figure 9B), identified by comparison with *in-silico* predictions based on peptide bond cleavage (b,y-Fragments, according to the nomenclature of Roepstorff and Fohlman.[19]) The fragments were assigned with mMass5.5.0.[20] Column "slice": The numbers in first square bracket correspond to the position of the ring cleavage within the sequence, the numbers in the second to the fragment length. The sequences in the last column do not represent the complete peptide, due to the ring cleavage their "C-terminus" is not a carboxylic acid, but an acylium ion or an oxazolone group.

ion	slice	*m/z*	z	sequence
M −H_2O	[12|1][1-12]*	1309.6270	1	DPDKDGTIDLKE*
M−2H_2O	[12|1][1-12]	1291.6165	1	DPDKDGTIDLKE*
b11	[9|10][1-11]	1212.6107	1	LKEDPDKDGTI
b11	[5|6][1-11]	1212.6107	1	GTIDLKEDPDK
b11	[3|4][1-11]	1212.6107	1	KDGTIDLKEDP
b11	[1|2][1-11]	1212.6107	1	PDKDGTIDLKE
b11−H_2O	[9|10][1-11]	1194.6001	1	LKEDPDKDGTI
b11−H_2O	[5|6][1-11]	1194.6001	1	GTIDLKEDPDK
b11−H_2O	[3|4][1-11]	1194.6001	1	KDGTIDLKEDP
b11−H_2O	[1|2][1-11]	1194.6001	1	PDKDGTIDLKE
b10	[3|4][1-10]	1115.5579	1	KDGTIDLKED
b10	[2|3][1-10]	1115.5579	1	DKDGTIDLKE
b10−H_2O	[3|4][1-10]	1097.5473	1	KDGTIDLKED
b10−H_2O	[2|3][1-10]	1097.5473	1	DKDGTIDLKE
b10	[1|2][1-10]	1083.5681	1	PDKDGTIDLK
b10−H_2O	[1|2][1-10]	1065.5575	1	PDKDGTIDLK
b9	[3|4][1-9]	1000.531	1	KDGTIDLKE
b9	[4|5][1-9]	987.4629	1	DGTIDLKED
b9−H_2O	[3|4][1-9]	982.5204	1	KDGTIDLKE
b9	[11|12][1-9]	971.4316	1	EDPDKDGTI
b9−H_2O	[4|5][1-9]	969.4524	1	DGTIDLKED
b9	[1|2][1-9]	955.4731	1	PDKDGTIDL
b8	[9|10][1-8]	941.4575	1	LKEDPDKD
b8	[8|9][1-8]	941.4575	1	DLKEDPDK
b9−H_2O	[1|2][1-9]	937.4625	1	PDKDGTIDL
b8−H_2O	[9|10][1-8]	923.4469	1	LKEDPDKD
b8−H_2O	[8|9][1-8]	923.4469	1	DLKEDPDK
b8	[5|6][1-8]	872.436	1	GTIDLKED
b8	[4|5][1-8]	872.436	1	DGTIDLKE
b8−H_2O	[5|6][1-8]	854.4254	1	GTIDLKED
b8−H_2O	[4|5][1-8]	854.4254	1	DGTIDLKE
b8	[12|1][1-8]	842.389	1	DPDKDGTI
b8	[1|2][1-8]	842.389	1	PDKDGTID
b7	[10|11][1-7]	828.3734	1	KEDPDKD
b7	[9|10][1-7]	826.4305	1	LKEDPDK
b8−H_2O	[12|1][1-8]	824.3785	1	DPDKDGTI
b8−H_2O	[1|2][1-8]	824.3785	1	PDKDGTID
b7	[8|9][1-7]	813.3625	1	DLKEDPD
b7−H_2O	[10|11][1-7]	810.3628	1	KEDPDKD
b7	[5|6][1-7]	757.409	1	GTIDLKE

b7	[4|5][1-7]	743.3934	1	DGTIDLK
b7	[3|4][1-7]	743.3934	1	KDGTIDL
b7−H_2O	[5|6][1-7]	739.3985	1	GTIDLKE
b7	[1|2][1-7]	727.3621	1	PDKDGTI
b6	[10|11][1-6]	713.3464	1	KEDPDK
b7−H_2O	[1|2][1-7]	709.3515	1	PDKDGTI
b6	[9|10][1-6]	698.3355	1	LKEDPD
b6	[8|9][1-6]	698.3355	1	DLKEDP
b6−H_2O	[11|12][1-6]	682.2679	1	EDPDKD
b6	[3|4][1-6]	630.3093	1	KDGTID
b6	[2|3][1-6]	630.3093	1	DKDGTI
b6−H_2O	[3|4][1-6]	612.2988	1	KDGTID
b6−H_2O	[2|3][1-6]	612.2988	1	DKDGTI
b5	[8|9][1-5]	601.2828	1	DLKED
b5	[11|12][1-5]	585.2515	1	EDPDK
b5	[10|11][1-5]	585.2515	1	KEDPD
b5−H_2O	[8|9][1-5]	583.2722	1	DLKED
b5	[12|1][1-5]	571.2358	1	DPDKD
b5−H_2O	[11|12][1-5]	567.2409	1	EDPDK
b5−H_2O	[10|11][1-5]	567.2409	1	KEDPD
b5−H_2O	[12|1][1-5]	553.2253	1	DPDKD
b5	[3|4][1-5]	515.2824	1	KDGTI
b5	[1|2][1-5]	513.2304	1	PDKDG
b5	[5|6][1-5]	500.2715	1	GTIDL
b5−H_2O	[3|4][1-5]	497.2718	1	KDGTI
b4	[9|10][1-4]	486.2558	1	LKED
b4	[8|9][1-4]	486.2558	1	DLKE
b5−H_2O	[4|5][1-5]	484.2038	1	DGTID
b5−H_2O	[5|6][1-5]	482.2609	1	GTIDL
b4−H_2O	[9|10][1-4]	468.2453	1	LKED
b4−H_2O	[8|9][1-4]	468.2453	1	DLKE
b4	[12|1][1-4]	456.2089	1	DPDK
b4	[1|2][1-4]	456.2089	1	PDKD
b4−H_2O	[12|1][1-4]	438.1983	1	DPDK
b4−H_2O	[1|2][1-4]	438.1983	1	PDKD

*and the 11 cyclic permutations thereof that are the result of the cleavage of one peptide bond within the cyclic peptide

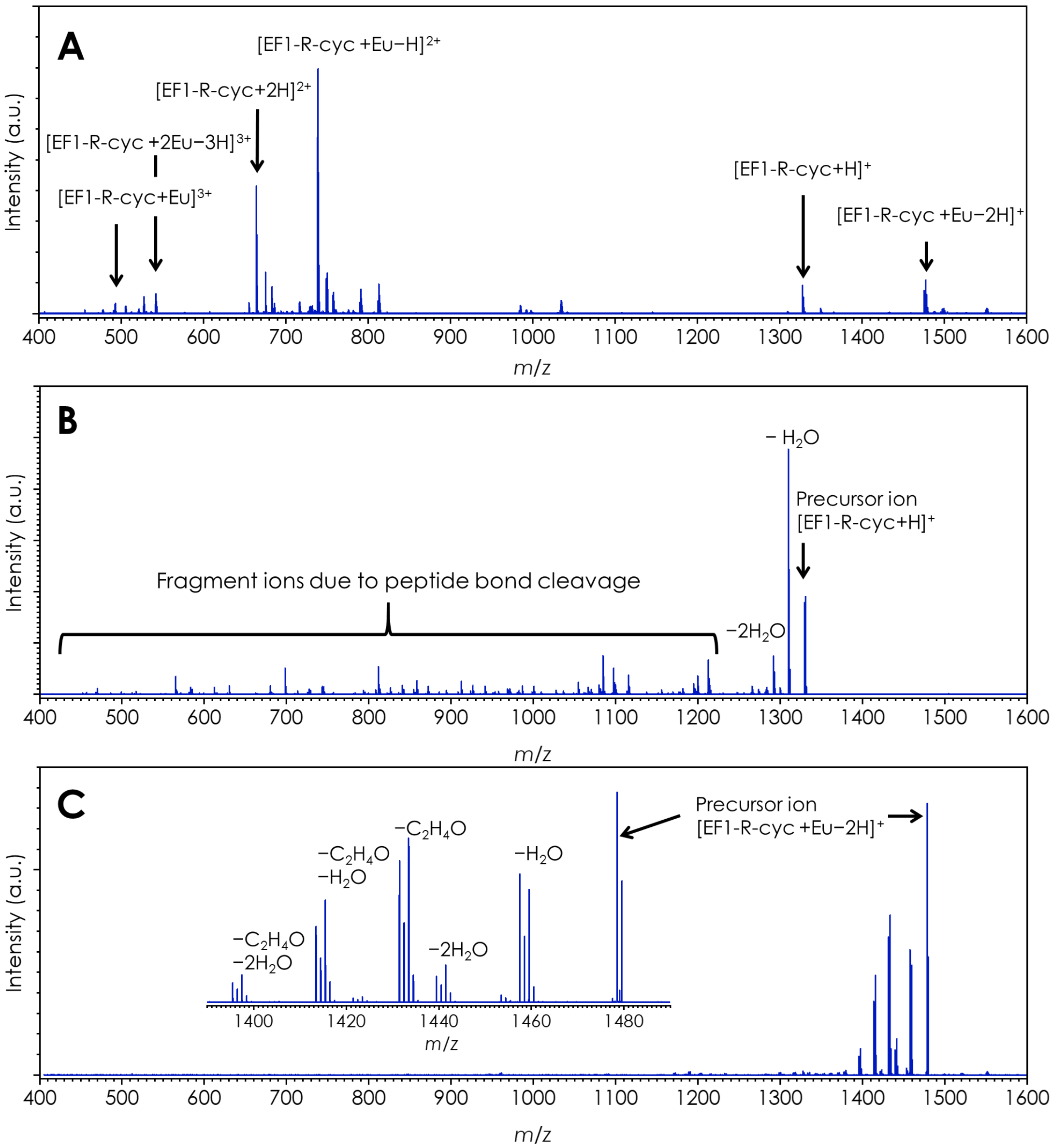

Figure S83 **(A)** Overview mass spectrum of a solution of 10 µM EF1-R-cyc with 50 µM $EuCl_3$ in LCMS water. Note that the relative intensities of the different charge states depend somewhat on the source conditions such as spray voltage and tube lens voltage. **(B)** Collision-induced dissociation of [EF1-R-cyc+H]$^+$, *m/z* 1326.6376. Besides water loss a large number of fragments due to peptide bond cleavage of the backbone is observed. **(C)** Collision-induced dissociation of [EF1-R-cyc +Eu−2H]$^+$, *m/z* 1475.5340. The fragmentation pattern is dominated by multiple water loss and loss of neutral C_2H_4O, backbone fragmentation is basically absent.

Table S30 Collision-induced dissociation of [EF1-R-cyc+H]+ ($C_{56}H_{91}N_{14}O_{23}^{+}$), matched peaks. Observed fragment ions of [EF1-R-cyc+H]+ (see Figure S81B), identified by comparison with *in-silico* predictions based on peptide bond cleavage (b,y-Fragments, according to the nomenclature of Roepstorff and Fohlman.[19]) The fragments were assigned with mMass5.5.0.[20] Column “slice”: The numbers in first square bracket correspond to the position of the ring cleavage within the sequence, the numbers in the second to the fragment length. The sequences in the last column do not represent the complete peptide, due to the ring cleavage their “C-terminus” is not a carboxylic acid, but an acylium ion or an oxazolone group.

Ion	slice	*m/z*	*z*	sequence
M −H_2O	[12|1][1-12]	1309.627	1	EKLDITGDKDPD*
M−2H_2O	[12|1][1-12]	1291.6165	1	EKLDITGDKDPD*
b11	[12|1][1-11]	1212.6107	1	EKLDITGDKDP
b11	[10|11][1-11]	1212.6107	1	PDEKLDITGDK
b11	[8|9][1-11]	1212.6107	1	KDPDEKLDITG
b11	[4|5][1-11]	1212.6107	1	ITGDKDPDEKL
b11	[9|10][1-11]	1199.5426	1	DPDEKLDITGD
b11	[2|3][1-11]	1199.5426	1	LDITGDKDPDE
b11−H_2O	[12|1][1-11]	1194.6001	1	EKLDITGDKDP
b11−H_2O	[10|11][1-11]	1194.6001	1	PDEKLDITGDK
b11−H_2O	[8|9][1-11]	1194.6001	1	KDPDEKLDITG
b11−H_2O	[4|5][1-11]	1194.6001	1	ITGDKDPDEKL
b11−H_2O	[9|10][1-11]	1181.5321	1	DPDEKLDITGD
b11−H_2O	[2|3][1-11]	1181.5321	1	LDITGDKDPDE
b10	[7|8][1-10]	1169.5685	1	DKDPDEKLDI
b10	[8|9][1-10]	1155.5892	1	KDPDEKLDIT
b10−H_2O	[8|9][1-10]	1137.5786	1	KDPDEKLDIT
b10	[12|1][1-10]	1115.5579	1	EKLDITGDKD
b10	[11|12][1-10]	1115.5579	1	DEKLDITGDK
b10−H_2O	[12|1][1-10]	1097.5473	1	EKLDITGDKD
b10−H_2O	[11|12][1-10]	1097.5473	1	DEKLDITGDK
b10	[10|11][1-10]	1084.5157	1	PDEKLDITGD
b10	[9|10][1-10]	1084.5157	1	DPDEKLDITG
b10−H_2O	[10|11][1-10]	1066.5051	1	PDEKLDITGD
b10−H_2O	[9|10][1-10]	1066.5051	1	DPDEKLDITG
b9	[8|9][1-9]	1054.5415	1	KDPDEKLDI
b9	[9|10][1-9]	1027.4942	1	DPDEKLDIT
b9−H_2O	[9|10][1-9]	1009.4837	1	DPDEKLDIT
b9	[12|1][1-9]	1000.531	1	EKLDITGDK
b9	[1|2][1-9]	986.5153	1	KLDITGDKD
b9	[4|5][1-9]	971.4316	1	ITGDKDPDE
b9	[3|4][1-9]	957.416	1	DITGDKDPD
b9−H_2O	[4|5][1-9]	953.4211	1	ITGDKDPDE
b8	[8|9][1-8]	941.4575	1	KDPDEKLD
b8	[7|8][1-8]	941.4575	1	DKDPDEKL
b8	[9|10][1-8]	926.4466	1	DPDEKLDI
b8	[10|11][1-8]	912.4673	1	PDEKLDIT
b8	[12|1][1-8]	872.436	1	EKLDITGD
b8	[11|12][1-8]	872.436	1	DEKLDITG
b8	[2|3][1-8]	858.4203	1	LDITGDKD

b8	[4|5][1-8]	842.389	1	ITGDKDPD
b8	[3|4][1-8]	842.389	1	DITGDKDP
b8−H_2O	[2|3][1-8]	840.4098	1	LDITGDKD
b7	[8|9][1-7]	826.4305	1	KDPDEKL
b7	[10|11][1-7]	811.4196	1	PDEKLDI
b7−H_2O	[8|9][1-7]	808.4199	1	KDPDEKL
b7−H_2O	[9|10][1-7]	795.3519	1	DPDEKLD
b7−H_2O	[10|11][1-7]	793.409	1	PDEKLDI
b7	[6|7][1-7]	757.2999	1	GDKDPDE
b7	[3|4][1-7]	745.3363	1	DITGDKD
b7	[2|3][1-7]	743.3934	1	LDITGDK
b7	[1|2][1-7]	743.3934	1	KLDITGD
b7−H_2O	[3|4][1-7]	727.3257	1	DITGDKD
b6	[8|9][1-6]	713.3464	1	KDPDEK
b6	[10|11][1-6]	698.3355	1	PDEKLD
b6	[9|10][1-6]	698.3355	1	DPDEKL
b6−H_2O	[10|11][1-6]	680.325	1	PDEKLD
b6−H_2O	[9|10][1-6]	680.325	1	DPDEKL
b6	[4|5][1-6]	630.3093	1	ITGDKD
b6	[3|4][1-6]	630.3093	1	DITGDK
b6−H_2O	[4|5][1-6]	612.2988	1	ITGDKD
b6−H_2O	[3|4][1-6]	612.2988	1	DITGDK
b5	[10|11][1-5]	583.3086	1	PDEKL
b5−H_2O	[10|11][1-5]	565.298	1	PDEKL
b5	[5|6][1-5]	517.2253	1	TGDKD
b5−H_2O	[5|6][1-5]	499.2147	1	TGDKD
b4	[12|1][1-4]	486.2558	1	EKLD
b4	[11|12][1-4]	486.2558	1	DEKL
b4	[10|11][1-4]	470.2245	1	PDEK
b4	[9|10][1-4]	457.1565	1	DPDE
b4−H_2O	[10|11][1-4]	452.214	1	PDEK

*and the 11 cyclic permutations thereof that are the result of the cleavage of one peptide bond within the cyclic peptide

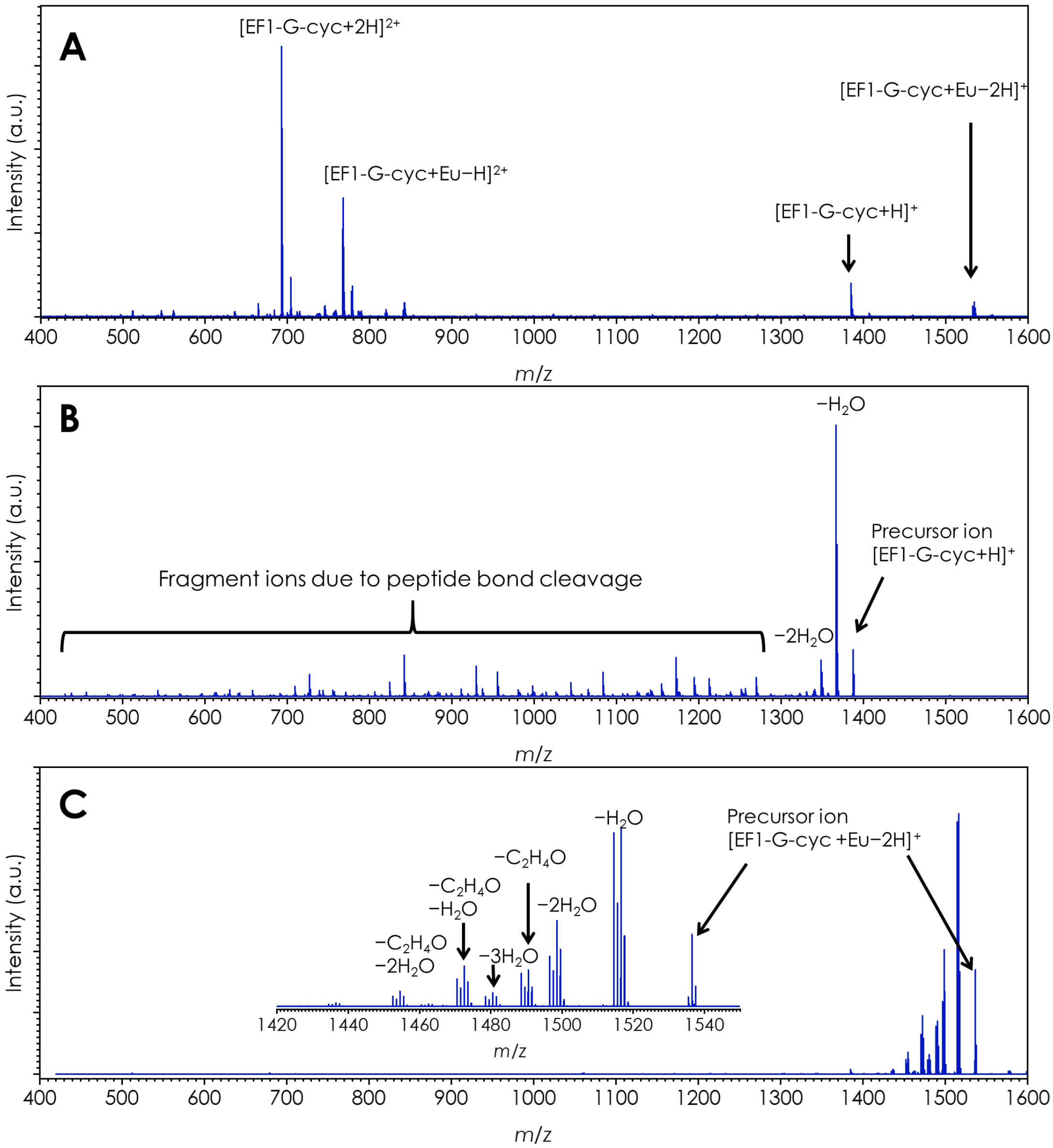

Figure S84 **(A)** Overview mass spectrum of a solution of 10 µM EF1-G-cyc with 50 µM $EuCl_3$ in LCMS water. Note that the relative intensities of the different charge states depend somewhat on the source conditions such as spray voltage and tube lens voltage. **(B)** Collision-induced dissociation of $[EF1\text{-}G\text{-}cyc+H]^+$, *m/z* 1384.6591. Besides water loss a large number of fragments due to peptide bond cleavage of the backbone is observed. **(C)** Collision-induced dissociation of $[EF1\text{-}G\text{-}cyc+Eu-2H]^+$, *m/z* 1532.5554. The fragmentation pattern is dominated by multiple water loss and loss of neutral C_2H_4O, backbone fragmentation is basically absent.

Table 31 Collision-induced dissociation of [EF1-G-cyc+H]$^+$ ($C_{58}H_{94}N_{15}O_{24}^+$), matched peaks. Observed fragment ions of [EF1-G-cyc+H]$^+$ (see Figure S82B), identified by comparison with *in-silico* predictions based on peptide bond cleavage (b,y-Fragments, according to the nomenclature of Roepstorff and Fohlman.[19]) The fragments were assigned with mMass5.5.0.[20] Column "slice": The numbers in first square bracket correspond to the position of the ring cleavage within the sequence, the numbers in the second to the fragment length. The sequences in the last column do not represent the complete peptide, due to the ring cleavage their "C-terminus" is not a carboxylic acid, but an acylium ion or an oxazolone group.

ion	slice	*m/z*	*z*	sequence
M−H_2O	[13|1][1-13]	1366.6485	1	GDPDKDGTIDLKE*
M−2H_2O	[13|1][1-13]	1348.6380	1	GDPDKDGTIDLKE*
b12	[10|11][1-12]	1269.6321	1	LKEGDPDKDGTI
b12	[6|7][1-12]	1269.6321	1	GTIDLKEGDPDK
b12	[4|5][1-12]	1269.6321	1	KDGTIDLKEGDP
b12	[2|3][1-12]	1269.6321	1	PDKDGTIDLKEG
b12	[12|13][1-12]	1256.5641	1	EGDPDKDGTIDL
b12	[5|6][1-12]	1256.5641	1	DGTIDLKEGDPD
b12−H_2O	[12|13][1-12]	1238.5535	1	EGDPDKDGTIDL
b12−H_2O	[5|6][1-12]	1238.5535	1	DGTIDLKEGDPD
b11	[7|8][1-11]	1212.6107	1	TIDLKEGDPDK
b11	[2|3][1-11]	1212.6107	1	PDKDGTIDLKE
b11−H_2O	[7|8][1-11]	1194.6001	1	TIDLKEGDPDK
b11−H_2O	[2|3][1-11]	1194.6001	1	PDKDGTIDLKE
b11	[4|5][1-11]	1172.5794	1	KDGTIDLKEGD
b11	[3|4][1-11]	1172.5794	1	DKDGTIDLKEG
b11−H_2O	[4|5][1-11]	1154.5688	1	KDGTIDLKEGD
b11−H_2O	[3|4][1-11]	1154.5688	1	DKDGTIDLKEG
b11	[6|7][1-11]	1141.5372	1	GTIDLKEGDPD
b11	[5|6][1-11]	1141.5372	1	DGTIDLKEGDP
b11−H_2O	[11|12][1-11]	1138.5375	1	KEGDPDKDGTI
b11−H_2O	[10|11][1-11]	1138.5375	1	LKEGDPDKDGT
b11−H_2O	[12|13][1-11]	1125.4695	1	EGDPDKDGTID
b10	[9|10][1-10]	1113.5059	1	DLKEGDPDKD
b10	[2|3][1-10]	1083.5681	1	PDKDGTIDLK
b10−H_2O	[2|3][1-10]	1065.5575	1	PDKDGTIDLK
b10	[4|5][1-10]	1057.5524	1	KDGTIDLKEG
b10	[5|6][1-10]	1044.4844	1	DGTIDLKEGD
b10−H_2O	[5|6][1-10]	1026.4738	1	DGTIDLKEGD
b10	[13|1][1-10]	1014.4374	1	GDPDKDGTID
b9	[10|11][1-9]	998.4789	1	LKEGDPDKD
b9	[9|10][1-9]	998.4789	1	DLKEGDPDK
b9−H_2O	[4|5][1-9]	982.5204	1	KDGTIDLKE
b9−H_2O	[10|11][1-9]	980.4684	1	LKEGDPDKD
b9−H_2O	[9|10][1-9]	980.4684	1	DLKEGDPDK
b9	[2|3][1-9]	955.4731	1	PDKDGTIDL
b9−H_2O	[2|3][1-9]	937.4625	1	PDKDGTIDL
b9	[6|7][1-9]	929.4575	1	GTIDLKEGD
b9	[5|6][1-9]	929.4575	1	DGTIDLKEG
b9−H_2O	[6|7][1-9]	911.4469	1	GTIDLKEGD

b9−H_2O	[5|6][1-9]	911.4469	1	DGTIDLKEG
b9	[13|1][1-9]	899.4105	1	GDPDKDGTI
b8	[10|11][1-8]	883.4520	1	LKEGDPDK
b8	[4|5][1-8]	871.4884	1	KDGTIDLK
b8−H_2O	[7|8][1-8]	854.4254	1	TIDLKEGD
b8−H_2O	[5|6][1-8]	854.4254	1	DGTIDLKE
b8	[2|3][1-8]	842.3890	1	PDKDGTID
b8	[1|2][1-8]	842.3890	1	DPDKDGTI
b8−H_2O	[2|3][1-8]	824.3785	1	PDKDGTID
b8−H_2O	[1|2][1-8]	824.3785	1	DPDKDGTI
b8	[6|7][1-8]	814.4305	1	GTIDLKEG
b7	[8|9][1-7]	771.3883	1	IDLKEGD
b7	[7|8][1-7]	757.4090	1	TIDLKEG
b7	[6|7][1-7]	757.4090	1	GTIDLKE
b7	[10|11][1-7]	755.3570	1	LKEGDPD
b7	[9|10][1-7]	755.3570	1	DLKEGDP
b7	[5|6][1-7]	743.3934	1	DGTIDLK
b7	[4|5][1-7]	743.3934	1	KDGTIDL
b7−H_2O	[7|8][1-7]	739.3985	1	TIDLKEG
b7−H_2O	[6|7][1-7]	739.3985	1	GTIDLKE
b7	[2|3][1-7]	727.3621	1	PDKDGTI
b7−H_2O	[2|3][1-7]	709.3515	1	PDKDGTI
b6	[9|10][1-6]	658.3042	1	DLKEGD
b6	[12|13][1-6]	642.2729	1	EGDPDK
b6	[11|12][1-6]	642.2729	1	KEGDPD
b6	[4|5][1-6]	630.3093	1	KDGTID
b6	[3|4][1-6]	630.3093	1	DKDGTI
b6−H_2O	[4|5][1-6]	612.2988	1	KDGTID
b6−H_2O	[3|4][1-6]	612.2988	1	DKDGTI
b6−H_2O	[2|3][1-6]	596.2675	1	PDKDGT
b5	[10|11][1-5]	543.2773	1	LKEGD
b5	[9|10][1-5]	543.2773	1	DLKEG
b5	[4|5][1-5]	515.2824	1	KDGTI
b5−H_2O	[4|5][1-5]	497.2718	1	KDGTI
b5−H_2O	[6|7][1-5]	482.2609	1	GTIDL
b4	[2|3][1-4]	456.2089	1	PDKD
b4	[1|2][1-4]	456.2089	1	DPDK
b4−H_2O	[2|3][1-4]	438.1983	1	PDKD
b4−H_2O	[1|2][1-4]	438.1983	1	DPDK
b4	[11|12][1-4]	430.1932	1	KEGD

*and the 12 cyclic permutations thereof that are the result of the cleavage of one peptide bond within the cyclic peptide

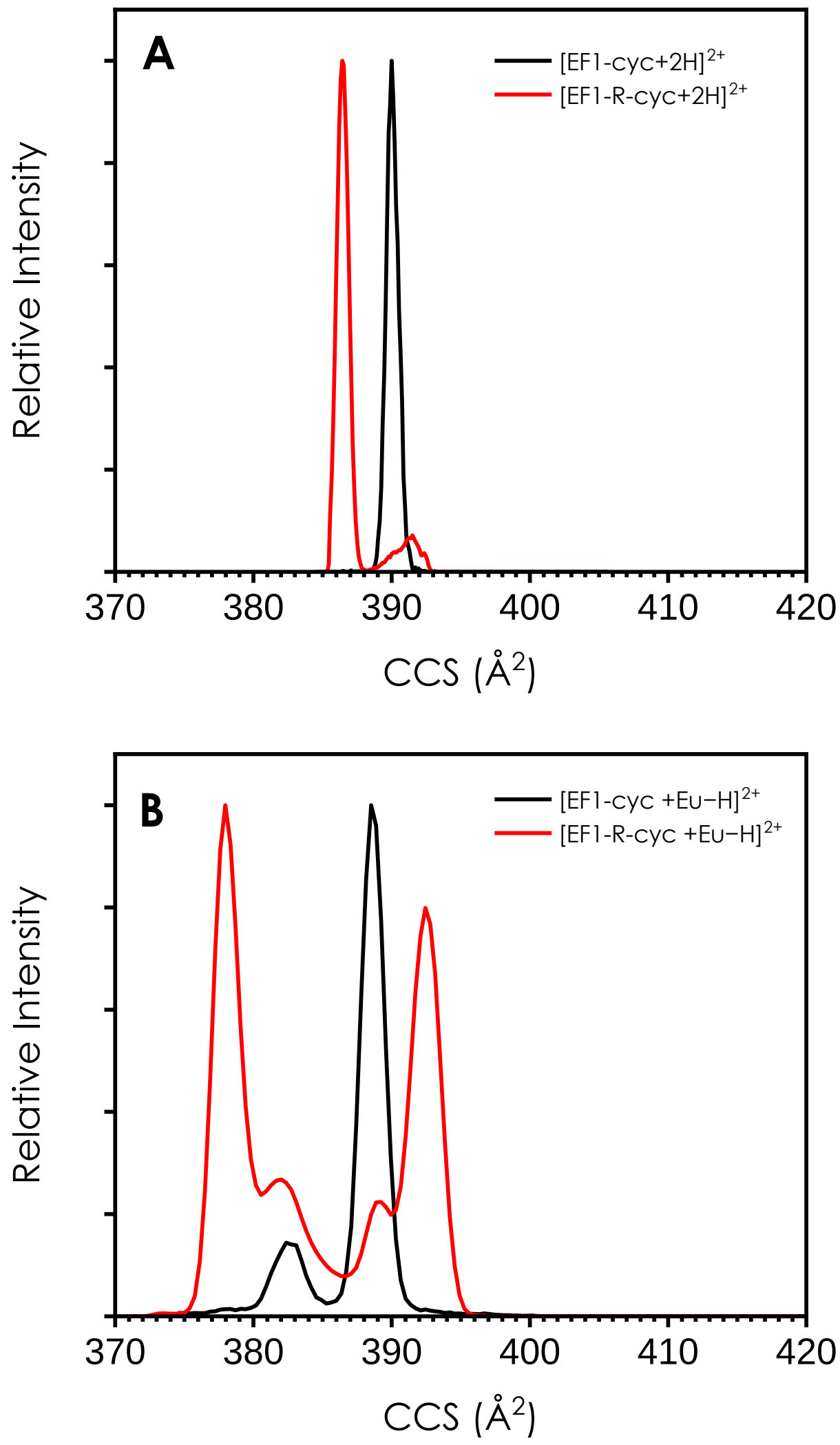

Figure S85 (A) Mobilograms of [EF1-cyc+2H]$^{2+}$ and [EF1-R-cyc+2H]$^{2+}$. **(B)** Mobilograms of [EF1-cyc+Eu−H]$^{2+}$ and [EF1-R-cyc+Eu−H]$^{2+}$.

4.6 Supplementary MD Data

Table S32 Amino acid residues involved in the coordination of La^{3+}, Eu^{3+} and Lu^{3+} in the peptide EF1-cyc as well residual coordinating water molecules from 500 ns MD trajectories (without initial restraints); mo=monodentate, bi=bidentate, bb = backbone oxygen.

Metal	Water	Coordinating Residues	Ln^{3+} potential
La^{3+}	7	Asp^3(bi), Asp^9(mo)	Hay and co-workers[17]
	6	Asp^1(bb), Asp^3(mo), Asp^3(bb), Glu^{12}(mo)	Merz and co-workers[14–16]
Eu^{3+}	7	Asp^3(mo), Asp^3(bb), Asp^5(mo)	Hay and co-workers[17]
	3	Asp^1(mo), Asp^1(bb), Asp^3(mo), Asp^3(bb), Glu^{12}(mo)	Merz and co-workers[14–16]
Lu^{3+}	4	Asp^3(mo),Asp^3(bb), Asp^5(mo), Asp^9(mo)	Hay and co-workers[17]

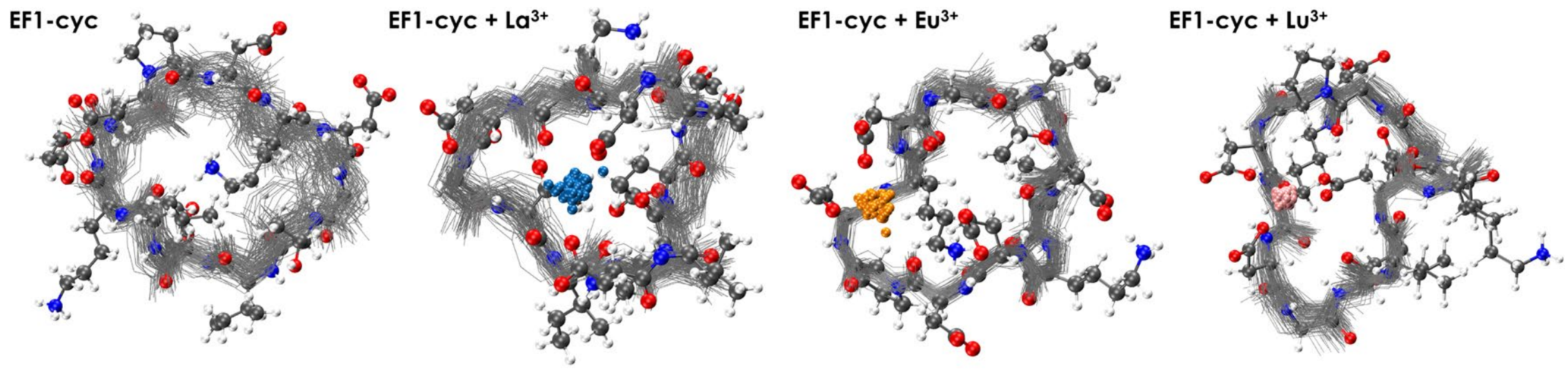

Figure S86 Molecular structures of EF1-cyc from left to right: without metal; with La^{3+}; with Eu^{3+} and with Lu^{3+} corresponding to the MD results shown in Table S32 using the Ln^{3+} potential of Hay and co-workers.[17] The static image from the MD trajectory (used) is depicted in ball-and-stick form, with 100 snapshots of the backbone (grey) and metal atoms superimposed.

Table S33 Amino acid residues involved in the coordination of La^{3+} and Eu^{3+} in the peptide EF1-G-cyc as well residual coordinating water molecules from 500 ns MD trajectories (without initial restraints); mo=monodentate, bi=bidentate, bb = backbone oxygen.

Metal	Water	Coordinating Residues	Ln^{3+} potential
La^{3+}	5	Asp^1(mo), Asp^3(mo), Asp^5(bi), Asp^9(mo)	Hay and co-workers[17]
Eu^{3+}	6	Asp^1(mo), Asp^3(mo), Asp^5(mo)	Hay and co-workers[17]

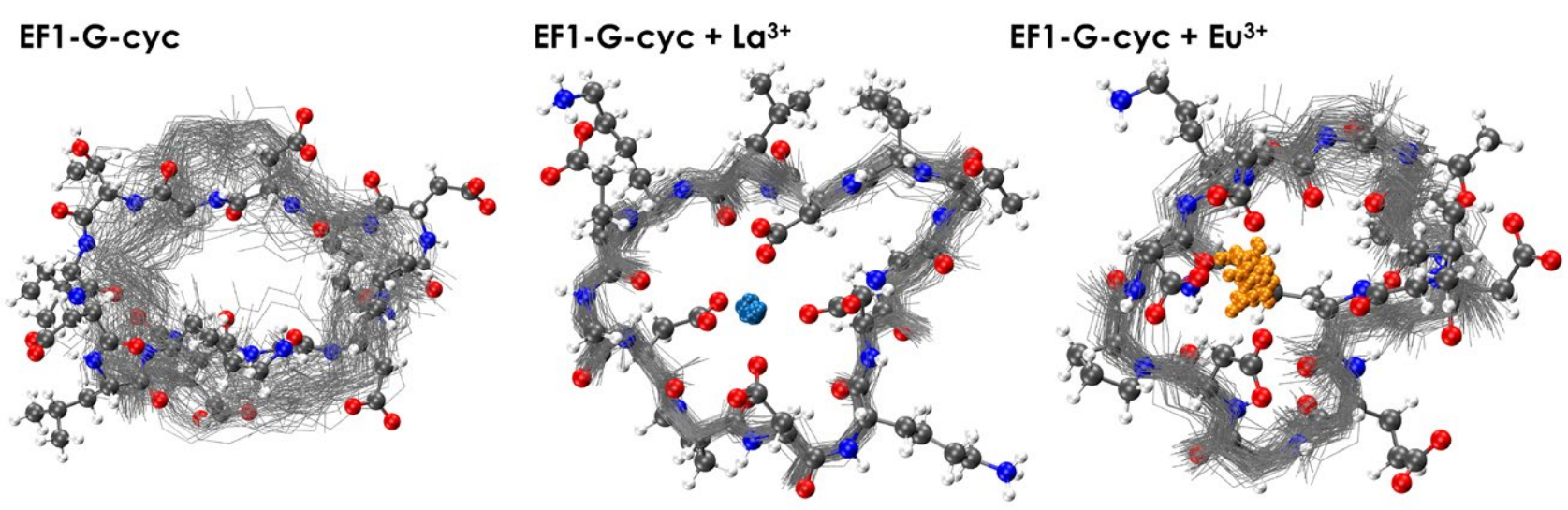

Figure S87 Molecular structures of EF1-G-cyc from left to right: without metal; with La^{3+} and with Eu^{3+} corresponding to the MD results shown in Table S33. The static image from the MD trajectory is depicted in ball-and-stick form, with 100 snapshots of the backbone (grey) and metal atoms superimposed.

5 Data Availability

Raw data files from NMR (.fid), TRLFS (.sif), ITC (.itc), CD (.jws) and MS (.raw) experiments, along with .xyz and .pdb files of the molecular dynamics simulations are available in the RODARE repository (DOI: 10.14278/rodare.4287).

6 References

[1] S. M. Gutenthaler, S. Tsushima, R. Steudtner, M. Gailer, A. Hoffmann-Röder, B. Drobot, L. J. Daumann, "Lanmodulin peptides – unravelling the binding of the EF-Hand loop sequences stripped from the structural corset" *Inorg. Chem. Front.* **2022**, *9*, 4009–4021.

[2] F. Taube, B. Drobot, A. Rossberg, H. Foerstendorf, M. Acker, M. Patzschke, M. Trumm, S. Taut, T. Stumpf, "Thermodynamic and Structural Studies on the Ln(III)/An(III) Malate Complexation" *Inorg. Chem.* **2019**, *58*, 368–381.

[3] B. Drobot, M. Schmidt, Y. Mochizuki, T. Abe, K. Okuwaki, F. Brulfert, S. Falke, S. A. Samsonov, Y. Komeiji, C. Betzel, T. Stumpf, J. Raff, S. Tsushima, "Cm3+/Eu3+ induced structural, mechanistic and functional implications for calmodulin" *Phys. Chem. Chem. Phys.* **2019**, *21*, 21213–21222.

[4] M. Leutenegger, Andor SIF image reader, MATLAB Central File Exchange, **2021**, https://www.mathworks.com/matlabcentral/fileexchange/11224-andor-sif-image-reader.

[5] C. A. Andersson, R. Bro, "The N-way Toolbox for MATLAB" *Chemom. Intell. Lab. Syst.* **2000**, *52*, 1–4.

[6] B. Drobot, R. Steudtner, J. Raff, G. Geipel, V. Brendler, S. Tsushima, "Combining luminescence spectroscopy, parallel factor analysis and quantum chemistry to reveal metal speciation – a case study of uranyl(VI) hydrolysis" *Chem. Sci.* **2015**, *6*, 964–972.

[7] B. Drobot, A. Bauer, R. Steudtner, S. Tsushima, F. Bok, M. Patzschke, J. Raff, V. Brendler, "Speciation Studies of Metals in Trace Concentrations: The Mononuclear Uranyl(VI) Hydroxo Complexes" *Anal. Chem.* **2016**, *88*, 3548–3555.

[8] D. S. Smith, "Solution of Simultaneous Chemical Equilibria in Heterogeneous Systems: Implementation in Matlab" *Chemistry Faculty Publications* **2019**. https://scholars.wlu.ca/chem_faculty/14

[9] F. Menges, Spectragraphy-optical spectroscopy software (version 1.2).

[10] S. M. Gutenthaler-Tietze, J. Kretzschmar, S. Tsushima, R. Steudtner, B. Drobot, L. J. Daumann, "Reversing Lanmodulin's Metal-Binding Sequence in Short Peptides Surprisingly Increases the Lanthanide Affinity" *Angew. Chem. Int. Ed.* **2025**, *64*, e202510453.

[11] S. M. Stow, T. J. Causon, X. Zheng, R. T. Kurulugama, T. Mairinger, J. C. May, E. E. Rennie, E. S. Baker, R. D. Smith, J. A. McLean, S. Hann, J. C. Fjeldsted, "An Interlaboratory Evaluation of Drift Tube Ion Mobility-Mass Spectrometry Collision Cross Section Measurements" *Anal. Chem.* **2017**, *89*, 9048–9055.

[12] F. Hennrich, S. Ito, P. Weis, M. Neumaier, S. Takano, T. Tsukuda, M. M. Kappes, "Cyclic ion mobility of doped [MAu24L18]2− superatoms and their fragments (M = Ni, Pd and Pt; L = alkynyl)" *Phys. Chem. Chem. Phys.* **2024**, *26*, 8408–8418.

[13] D. A. Case, J. T. Berryman, R.M. Betz, D.S. Cerutti, T.E. Cheatham, T.A. Darden, R.E. Duke, T.J. Giese, H. Gohlke, A.W. Goetz, N. Homeyer, S. Izadi, P. Janowski, J. Kaus, A. Kovalenko, T.S. Lee, S. LeGrand, P. Li, T. Luchko, R. Luo, B. Madej, K.M. Merz, G. Monard, P. Needham, H. Nguyen, H.T. Nguyen, I. Omelyan, A. Onufriev, D.R. Roe, A. Roitberg, R. Salomon-Ferrer, C.L. Simmerling, W. Smith, J. Swails, R.C. Walker, J. Wang, R.M. Wolf, X. Wu, D.M. York, P.A. Kollman **2015**.

[14] P. Li, K. M. Merz, "Taking into Account the Ion-induced Dipole Interaction in the Nonbonded Model of Ions" *J. Chem. Theory Comput.* **2014**, *10*, 289–297.

[15] P. Li, L. F. Song, K. M. Merz, "Parameterization of Highly Charged Metal Ions Using the 12-6-4 LJ-Type Nonbonded Model in Explicit Water" *J. Phys. Chem. B* **2015**, *119*, 883–895.

[16] Z. Li, L. F. Song, P. Li, K. M. Jr. Merz, "Parametrization of Trivalent and Tetravalent Metal Ions for the OPC3, OPC, TIP3P-FB, and TIP4P-FB Water Models" *J. Chem. Theory Comput.* **2021**, *17*, 2342–2354.

[17] P. Kantakevičius, C. Mathiah, L. O. Johannissen, S. Hay, "Chelator-Based Parameterization of the 12-6-4 Lennard-Jones Molecular Mechanics Potential for More Realistic Metal Ion–Protein Interactions" *J. Chem. Theory Comput.* **2022**, *18*, 2367–2374.

[18] D. S. Wishart, "Interpreting protein chemical shift data" *Prog. Nucl. Magn. Reson. Spectrosc.* **2011**, *58*, 62–87.

[19] P. Roepstorff, J. Fohlman, "Proposal for a common nomenclature for sequence ions in mass spectra of peptides" *Biomed. Mass Spectrom.* **1984**, *11*, 601.

[20] T. H. J. Niedermeyer, M. Strohalm, "mMass as a Software Tool for the Annotation of Cyclic Peptide Tandem Mass Spectra" *PLOS ONE* **2012**, *7*, e44913.
